# Molecular signatures define alopecia areata subtypes and transcriptional biomarkers

**DOI:** 10.1016/j.ebiom.2016.03.036

**Published:** 2016-03-31

**Authors:** Ali Jabbari, Jane E. Cerise, James C. Chen, Julian Mackay-Wiggan, Madeleine Duvic, Vera Price, Maria Hordinsky, David Norris, Raphael Clynes, Angela M. Christiano

**Affiliations:** a Department of Dermatology, Columbia University, New York, NY, USA; b Department of Systems Biology, Columbia University, New York, USA.; c Department of Dermatology, M.D. Anderson Cancer Center, Houston, TX, USA; d Department of Dermatology, UCSF, San Francisco, CA, USA; e Department of Dermatology, University of Minnesota, Minneapolis, MN, USA; f Department of Dermatology, University of Colorado, Denver, CO, USA; g Department of Genetics & Development, Columbia University, New York, NY, USA

**Keywords:** Alopecia areata, Biomarkers, Autoimmune

## Abstract

Alopecia areata (AA) is an autoimmune disease typified by nonscarring hair loss with a variable clinical course. In this study, we conducted whole genome gene expression analysis of 96 human scalp skin biopsy specimens from AA or normal control subjects. Based on gene expression profiling, samples formed distinct clusters based on the presence or absence of disease as well as disease phenotype (patchy disease compared with alopecia totalis or universalis). Differential gene expression analysis allowed us to robustly demonstrate graded immune activity in samples of increasing phenotypic severity and generate a quantitative gene expression scoring system that classified samples based on interferon and cytotoxic T lymphocyte immune signatures critical for disease pathogenesis.

## Highlights

• Gene expression analysis of 96 scalp biopsies from patients with alopecia areata (AA) and healthy controls was performed.
• Samples from AA patchy, alopecia universalis/totalis and control patients formed distinct clusters by gene expression.
• A set of gene expression biomarkers, the Alopecia Areata Disease Activity Index (ALADIN), was formulated.
• ALADIN distinguished AA phenotypes and normal controls.
• ALADIN may have utility in clinical trials of AA.

Alopecia areata is a disease characterized by autoimmune attack of the hair follicle. A complete understanding of the signaling pathways involved in the disease is lacking. Based on gene expression profiling of skin samples from 96 patients and controls, a set of biomarkers, termed the Alopecia Areata Disease Activity Index, or ALADIN, was formulated. ALADIN was able to distinguish samples from patients with patchy disease from samples from patients with the more extensive forms of disease. The usefulness of this biomarker tool is ready to be assessed in clinical trials of therapeutics for alopecia areata.

## 1 Introduction

Alopecia areata (AA) is an autoimmune skin disease in which the hair follicle is the target of immune attack. Patients characteristically present with round or ovoid patches of hair loss usually on the scalp that can spontaneously resolve, persist, or progress to involve the scalp or the entire body (Gilhar et al., 2012). The three major phenotypic variants of the disease are patchy-type AA (AAP), which is often localized to small areas on the scalp or in the beard area, alopecia totalis (AT), which involves the entire scalp, and alopecia universalis (AU), which involves the entire body surface area. There are currently no FDA approved drugs for AA. Treatment is often empiric and typically involves observation, intralesional steroids, topical immunotherapy or broad immunosuppressive treatments of variable efficacy. The more severe forms of the disease, AU and AT, are often recalcitrant to treatment. Despite its high prevalence and the need for effective treatments, the molecular and cellular effectors of AA have not been well studied. It is currently unclear if distinct pathogenic mechanisms drive these more severe forms of the disease, or whether those disease mechanisms are exacerbated in AU and AT compared to AAP.

Histologically, AA is characterized by an immune infiltrate centered around the hair bulb. This infiltrate is made up of predominantly CD4 and CD8 T cells (Ito et al., 2008), although other cell types, including natural killer cells (Ito et al., 2008, Kaufman et al., 2010), macrophages (Castellana et al., 2014), mast cells (Bertolini et al., 2014) and eosinophils (Elston et al., 1997) may also be present. Substantial differences in histological appearance have not been described when comparing AAP, AT, and AU samples, although others have cited that disease duration may impact the amount of peribulbar infiltrate, with more acute cases being reported as having relatively more robust inflammation and chronic cases having less (Whiting, 2003a).

Recent strides in the field have transformed our understanding of disease pathogenesis, drug targets, and potential therapeutic solutions. The results of our initial genome wide association study (GWAS) (Petukhova et al., 2010) and, more recently, of a large GWAS meta-analysis (Betz et al., 2015) have identified numerous loci that imply a strong role for variants in genes that direct and influence immune responses. Interestingly, almost all of the implicated immune genes have been associated with other autoimmune diseases, including type 1 diabetes, rheumatoid arthritis, and celiac disease, lending further support for the common-cause hypothesis of autoimmune diseases (Gregersen and Olsson, 2009). Of particular note, single nucleotide polymorphisms in the ULBP3 and ULBP6 genes confer an increased risk for developing the disease and are uniquely associated with AA. The ULBP family of genes encodes proteins that serve as ligands for NKG2D and, when expressed, mark a cell for immune targeting by natural killer cells or NKG2D-expressing CD8 T cells. These data led to the recognition of NKG2D-bearing CD8 T cells in the peribulbar infiltrate in skin sections of lesional scalp biopsy specimens of patients with AA as well as in affected skin and skin-draining lymph nodes from the C3H/HeJ mouse model of spontaneous AA (Petukhova et al., 2010, Xing et al., 2014). Adoptive transfer of this population of cells from C3H/HeJ mice with alopecia into unaffected C3H/HeJ mice led to the induction of alopecia, substantiating a pivotal role for these effector cells in the mouse AA model (Xing et al., 2014).

We previously identified prominent interferon (IFN) and common gamma chain cytokine (γc) signatures in AA, both of which we postulated contributed to disease pathogenesis (Xing et al., 2014). Based on these findings, a therapeutic strategy based on inhibition of critical members of a family of signaling molecules, Janus kinases (JAKs), was found to be effective at treating AA in a mouse model of disease and a small series of human patients. Gene expression profiling played a critical role in our selection of small molecule JAK inhibitors for AA, and we reasoned that gene expression studies that include the different AA phenotypes have the potential to provide additional insights into novel therapeutic solutions as well as pathogenic mechanisms.

Here, we profiled scalp biopsy samples collected from a total of 96 patients with a range of AA phenotypes and normal control patients. Patient samples were collected from the National Alopecia Areata Registry sites across the United States after phenotypic classification by dermatologists who specialize in hair disorders. Skin biopsy samples were then interrogated using microarray-based gene expression analysis to identify the AA-specific gene expression signature. We found a striking level of immune activity in AT/AU samples by gene expression analysis. Despite the lack of consistently effective treatments in AT and AU, these data suggest that drugs that disrupt this immune activity may be useful for therapeutic purposes. Furthermore, based on our data, we created an Alopecia Areata Disease Severity Index (ALADIN), a gene expression metric that effectively distinguishes AT/AU samples, AAP samples, and normal control (NC) samples from each other. ALADIN may be used to accurately track disease activity in patients undergoing conventional or experimental treatments.

## 2 Materials and Methods

### 2.1 Experimental Design

The objective of this study was to identify immune and nonimmune signaling pathways as well as biomarkers in the affected skin from patients with AA. The overall design was to use whole genome based gene expression techniques on skin samples from patients with AA of variable severity and compare those with skin samples from healthy controls. Sample collection, sample processing and data analysis are described below.

### 2.2 Human Patient Demographics

Two independent datasets were collected from four National Alopecia Areata Foundation (NAAF) registry sites. Our discovery dataset consisted of samples from 63 patients (20 AAP, 20 AT/AU, and 23 normal controls). Our validation dataset was comprised of samples from 33 patients (8 AAP, 12 AT/AU, and 13 Normal controls). A more complete description of the datasets broken down by disease status, gender, age, and NAAF registry site is provided in Supplemental Table 1.

### 2.3 Ethics Statement

All studies have been approved by the Institutional Review Boards at the Columbia University Medical Center, the University of Minnesota, the University of California, San Francisco, and the M.D. Anderson Cancer Center and were conducted under the Declaration of Helsinki principles. Informed written consent was received from participants prior to inclusion in the study.

### 2.4 Human Tissue Sampling and Processing

Skin punch biopsy specimens were fixed in the PAXgene Tissue Containers and shipped overnight to Columbia University. Samples were bisected, with one half of the sample processed using the PAXgene tissue miRNA kit to extract RNA. Library prep was performed for microarray analysis using Ovation RNA Amplification System V2 and Biotin Encore kits (NuGen Technologies, Inc., San Carlos, CA). Samples were subsequently hybridized to Human Genome U133 Plus 2.0 chips (Affymetrix, Santa Clara, CA) and scanned at the Columbia University Pathology Core or the Yale Center for Genome Analysis.

Microarray data were deposited in Gene Expression Omnibus, accession GSE68801.

### 2.5 Analysis Packages

Quality control of microarrays was performed using the affyAnalysisQC package from http://arrayanalysis.org/. Differential expression in these studies was defined by an absolute fold change threshold of 1.5 with a Benjamini–Hochberg-corrected significance threshold of 0.05. Clustering and principal component analysis was done using the modules provided in the Bioconductor R package. Network images were generated with Cytoscape.

### 2.6 Microarray Preprocessing and Quality Control

Microarray preprocessing was performed using BioConductor in R. Preprocessing of the two datasets, discovery dataset (63 samples) and the validation dataset (33 samples), were performed separately using the same pipeline. Quality control was performed using the affyanalysisQC package from http://arrayanalysis.org/. The discovery dataset and the validation dataset were normalized separately using GCRMA and MAS5. The Affymetrix HGU-133Plus2 array contains 54675 probe sets (PSIDs). Filtering was performed so that PSIDs that were on the X or Y chromosome, that were Affymetrix control probe sets, or that did not have Gene Symbol annotation were removed from all arrays for further downstream analysis. For the 3D plot of the ALADIN scores, all 96 samples from both datasets were combined before performing GCRMA normalization and correcting for batch effects.

### 2.7 Sample Filtering and Batch Correction

In order to perform analysis on the 63 AA lesional (both AT/AU and AAP) and NC samples in the discovery data set, PSIDs were further filtered to remove PSIDs that had not been called present on at least one of the 63 arrays resulting in 36954 PSIDs. Correction for batch effects was performed using the implementation of the function ComBat available in the sva package with gender and AA group (AT/AU, AAP, and normal) used as covariates. No batch correction was required for the validation set.

### 2.8 Differential Expression Analysis

Differential analysis was performed on the batch corrected discovery data set using linear models as implemented in the limma package in Bioconductor (Smyth, 2004). Two-sample comparisons were performed separately to identify PSIDs differentially expressed in AA patients versus normal controls, in AAP patients versus normal controls, and in AT/AU patients versus normal controls treating gender as a fixed factor.

### 2.9 Principal Component Analysis

Principal component analysis was performed on all 36954 PSIDs that were used to perform differential expression analysis. The probability density of the first two principal components was estimated for each group (AT/AU, AAP, and NC) assuming a bivariate distribution.

### 2.10 Calculation of ALADIN Scores

The CTL, IFN and KRT ALADIN scores were calculated for each sample as described previously (Xing et al., 2014). Briefly, z-scores are calculated for each PSID relative to the mean and standard deviation of normal controls. Z-scores for each gene are obtained by averaging z-scores of PSIDs mapping to that gene. Signature scores are then calculated averages of the z-scores for genes belonging to the corresponding signature.

### 2.11 Funding

This work was supported in part by US Public Health Service National Institutes of Health NIAMS grants R01AR056016 (to AMC), R21AR061881 (to AMC and RC), U01AR067173 (to AMC) and P30AR044535 (the Columbia University Skin Disease Research Center), as well as the Locks of Love Foundation and the Alopecia Areata Initiative. JEC and JCC are supported by the T32GM082771 Medical Genetics Training Grant (issued to AMC). AJ is supported by a NIAMS grant (K08AR069111), a Physician Scientist Career Development Award from the Dermatology Foundation, the Louis V. Gerstner Jr Scholars Program, and the Irving Scholars Program from the Irving Institute for Clinical and Translational Research at the Columbia University Medical Center.

Additional Materials and Methods are presented in the Supplemental Materials.

## 3 Results

### 3.1 AA Gene Expression Signatures

Gene expression profiling was performed on samples from 96 patients, divided into a discovery dataset of 63 patients and an external validation dataset of 33 patients (for a more complete description refer to Methods section and Supplemental Table 1). Microarray-based gene expression analysis was conducted on the discovery dataset, consisting of 20 AAP, 20 AT/AU, and 23 normal control scalp skin biopsy specimens. Differentially expressed genes were identified based on the comparison of AA samples versus normal controls. From this set of analyses, a disease specific gene expression profile was generated, based on differentially expressed genes selected with an absolute fold change (FC) > 1.5 and false discovery rate (FDR) < 0.05. The AA-specific disease signature was comprised of 1083 Affymetrix probes that showed increased expression and 919 Affymetrix probes that showed decreased expression in AA (Supplemental Table 2). In order to ensure the robustness of the data from this initial set of samples, external validation was performed using an additional 8 AAP, 12 AT/AU, and 13 normal control scalp skin biopsy specimens as a validation set.

**Fig. 1:**
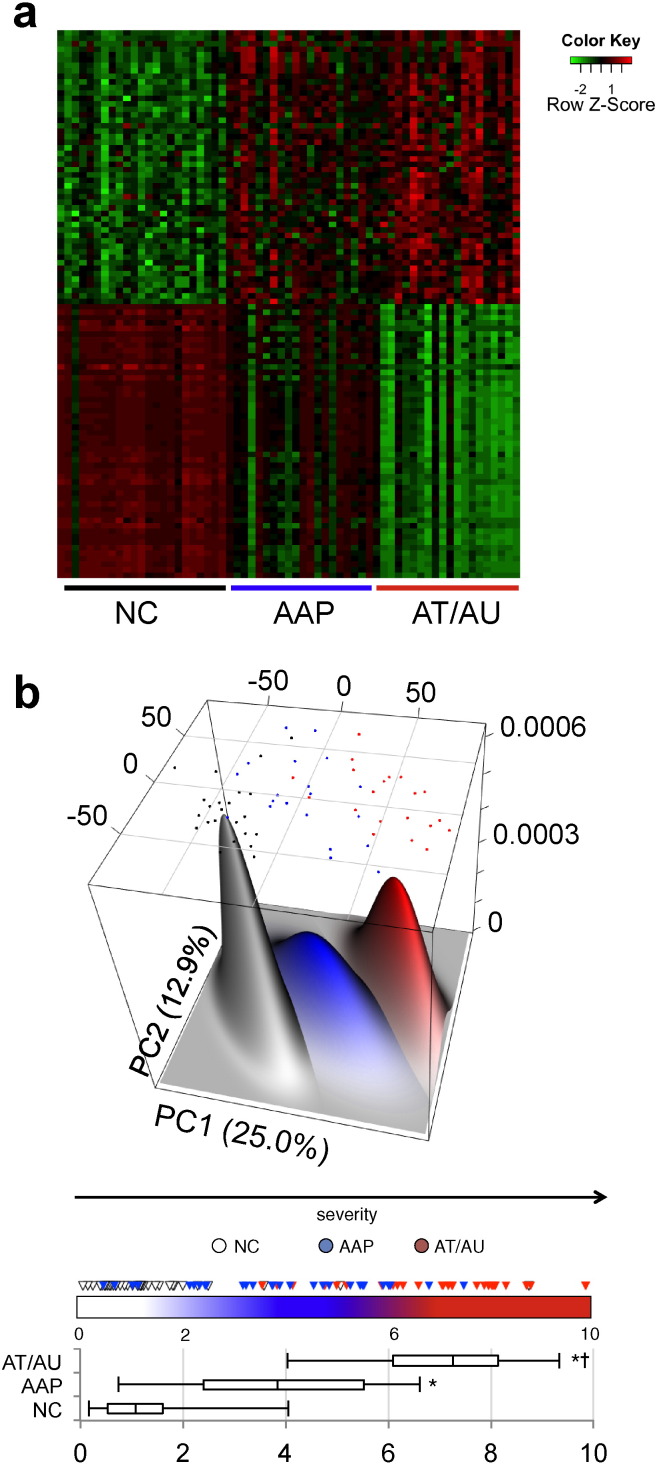
Alopecia areata disease-specific signature. (a) Heat map of the 50 most differentially expressed genes with increased expression and 50 most differentially expressed genes with decreased expression within the AA-specific disease signature among AT/AU, AAP, and NC samples in the training set. (b) Expression terrain map of samples arrayed along the principal components of differential gene expression. The dots represent the location of each sample in the expression space (black = NC, blue = AAP, red = AT/AU), and the size of the peaks are generated based on the number of samples in the region (more juxtaposed samples produce higher, wider peaks). The principal component space can be condensed into a single numeric score reflecting the risk of a sample being a control, AAP, or AT/AU based on its location in the terrain space. This consensus score provides statistically significant separation control, AAP, and AT/AU sample cohorts (box-and-whiskers plot). Box denotes the interquartile range and median, whiskers denote the 5th and 95th percentiles, * indicates statistical significance against NC, † indicates statistical significance versus AAP.

Of note, genes associated with cell mediated cytotoxicity, including PRF1 and several granzymes, as well as immune cell trafficking chemokine genes were among the top genes listed as showing increased expression, while hair keratin associated genes and developmental genes, such as DSMG4, FGF18, and GPRC5D, were among those genes showing decreased expression. Patterns of gene expression distinguished the phenotypic groups from each other, with normal controls and AT/AU samples showing the greatest disparity (Fig. 1a). Plotting the samples in a terrain expression map revealed three clusters corresponding to healthy controls, AAP patients, and AT/AU patients (Fig. 1b, Supplemental Table 3). These patient groups fell along a near-linear path through the terrain map.

To more concisely represent this multidimensional data, we generated a single score evaluating the relative risk of any given sample being AAP or AT/AU based on its location in this terrain. This score is a scalar representation of normalized deviation that any given patient has from an “unaffected” molecular state, based on a consensus of all differentially expressed genes between AA and healthy controls (see Materials and Methods section). The resulting score is bounded between 0–10 (represented as the color bar in Fig. 1b), 10 representing risk of maximal severity (AT/AU, red), and 0 representing minimal risk of disease (healthy controls, white). AAP samples on the whole fell in between these two extremes (blue). Control samples in the dataset had a cohort median score of 1.08; AAP, a median of 3.83; and AT/AU, a median of 7.26 (Fig. 1b box-and-whiskers plot). Both disease groups were statistically significant from unaffected controls by nonparametric statistics (p < 0.05), and AT/AU patients were additionally separable from AAP patients (p < 0.05). The differentially expressed genes from the discovery data set were able to distinguish the AA samples from normal samples by hierarchical clustering in our validation set (Supplemental Fig. 1). These data suggest the pathology of AA can be expressed at the level of molecular gene expression, and that AAP samples exhibit an AA-specific molecular state that is intermediate between AT/AU and normal controls.

### 3.2 AT/AU Skin Samples Are Immunologically Active

**Fig. 2:**
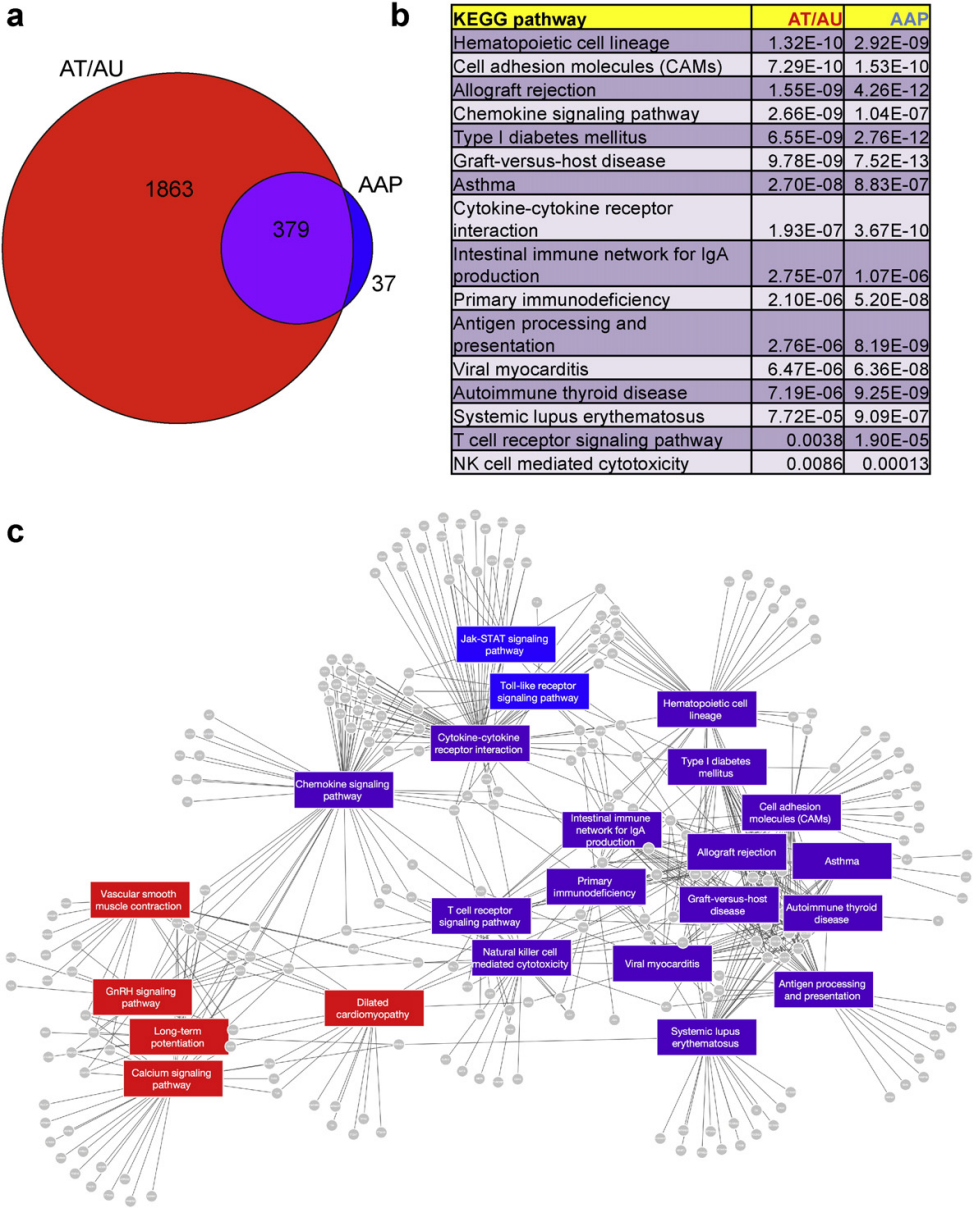
Increased gene expression complexity and sustained inflammation in alopecia totalis and universalis. (a) Venn diagram of differentially expressed gene probesets in AT/AU compared with normal (“AT/AU”) and AAP compared with normal (“AAP”). Shown are the numbers of differentially expressed genes within each section of the Venn diagram. (b) List of KEGG pathways shared between AT/AU versus normal controls and AAP versus normal controls. (c) Network map of KEGG pathways upregulated in AT/AU versus normal controls (red), AAP versus normal controls (blue), or shared pathways in both AT/AU versus normal and AAP versus normal controls.

The linear presentation of molecular classification between controls, AAP, and AT/AU in global gene expression analyses, in combination with the presence of immune-related genes in the disease signature, led us to question whether the AT/AU samples were immunologically active. Since AT/AU samples seemed to exhibit a more severe AA-specific signature than those of AAP based on both the level of differential expression and the number of differentially expressed genes, we separately examined the gene expression profiles of AT/AU compared with normal as well as that for AAP compared with healthy controls.The AT/AU-specific disease signature, based on FC > 1.5 and FDR < 0.05, was comprised of 2242 probesets with increased expression and 1651 probesets with decreased expression (Supplemental Table 4). The AAP-specific disease signature, based on similar thresholds, exhibited much lower numbers of differentially expressed genes, with only 416 probesets with increased expression and 550 probesets with decreased expression (Supplemental Table 5). Comparison of the AT/AU- and AAP-specific genes lists showed overlap of AAP-specific genes among the two lists, with few AAP-specific genes not contained within the AT/AU-specific gene list (Fig. 2a). These data indicate that the gene expression perturbations in AT/AU are more complex and more severe than the AAP form of the disease.

Pathway analysis was performed for signatures that were upregulated in either AAP or AT/AU samples (Supplemental Tables 6 and 7). Interestingly, the shared set of pathways that were upregulated in both AAP and AU/AT (Fig. 2b, c), including “Graft-versus-host disease,” “Type I diabetes mellitus,” “Allograft rejection,” “Cell adhesion molecules,” and “Antigen processing and presentation,” were made up of antigen presentation genes, supporting the pathogenic theme of loss of immune privilege of the hair follicle microenvironment and immune activation (Chen et al., 2015) in AA. Interestingly, the “Chemokine signaling pathway” was also found to be significantly upregulated, raising the possibility of targeting these intercellular trafficking molecules for therapeutic purposes, as has been proposed for other autoimmune skin diseases (Rashighi et al., 2014). These results indicate that the majority of the active immune pathways in AA are the same in the milder as well as the more severe forms of the disease.

### 3.3 Infiltrate Gene Expression Signatures Correlate With AA Phenotype

**Fig. 3:**
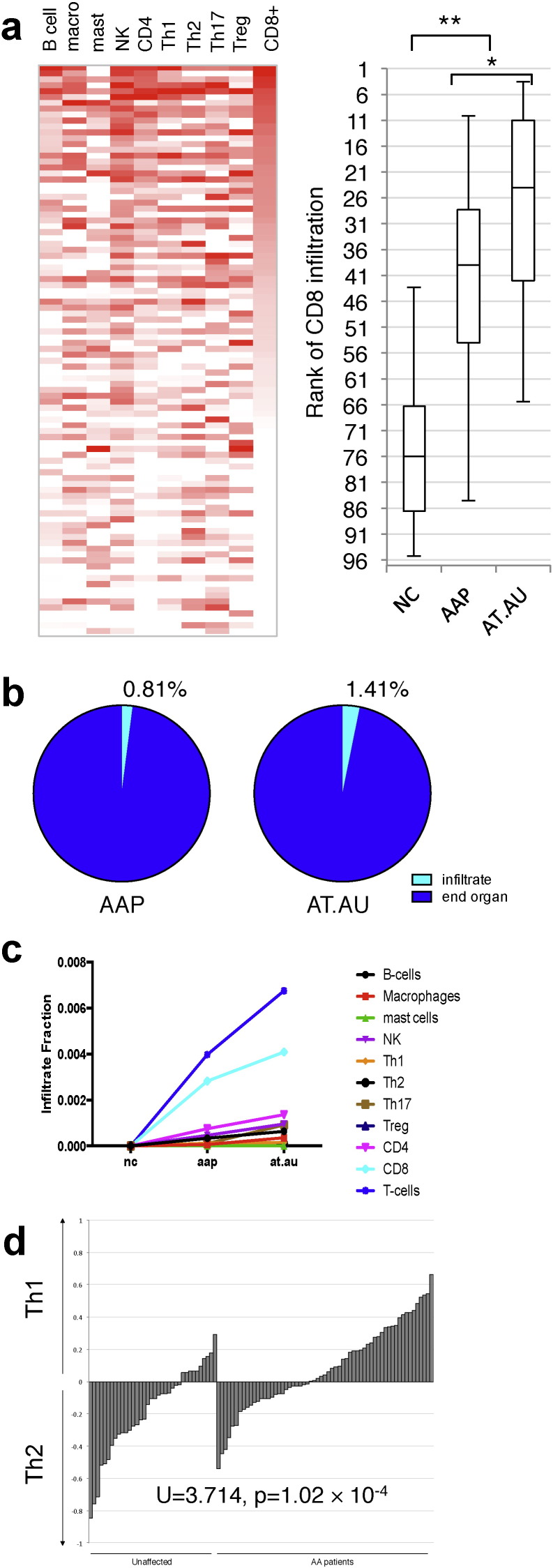
Immune cell infiltrate gene expression signatures correlate with AA phenotype. (a), Relative estimates of the indicated infiltrating immune cells on a patient-by-patient basis based on consensus expression of corresponding immune markers (heatmap, right). More intense red indicates increasing amounts of infiltrate. Patients are ranked by CD8 infiltration. The box-and-whiskers plot reflects the distribution of the indicated clinical presentations according to CD8 infiltration rank (lower rank indicates higher levels of infiltration). Box denotes the interquartile range and median, whiskers denote the 5th and 95th percentiles, * indicates statistical significance p = 0.005, ** indicates p < 1 × 10^− 5^. (b) Using the consensus expression, infiltration burden of the biopsy samples is estimated for each presentation cohort, AAP = patchy, (left) AT/AU = totalis/universalis (right), as well as the relative share of each immune tissue type in the total infiltration density compared to unaffected controls (line chart). (c) Differences in estimated infiltration of each indicated immune type expressed as a fraction of total sample signal across NC, AAP, and AT/AU. (d) Using these derived immune scores, the relative load of Th1 vs Th2 cells infiltrating in each patient sample biopsy can be measured as a log-ratio (Th1:Th2). Positive values indicate greater levels of Th1, and negative values indicate greater levels of Th2 signal. On the whole, AA patients (AA or AT/AU) exhibit an overall increase in the Th1:Th2 log-ratio compared to unaffected controls, p = 1.02 × 10^− 4^ by U-test.

The presence of immune-related marker genes in our gene expression array cohorts led us to interrogate whether or not these infiltrates could be detected directly in microarray analysis. The ability to detect infiltrating populations would prove informative to understanding the pathogenesis and characterization of AA. To identify infiltrating immune cell signatures, we adopted unique gene expression signatures defining each of several infiltrating immune cells from a work studying infiltration in cancers (Bindea et al., 2013) and used this as our Immune Gene Signature (IGS). This approach yielded gene markers for several immune types including but not limited to B-cells, T-cells, macrophages, natural killers, and mast cells. We built numeric relative measurements of the relative infiltration of each of these tissue types as a function of the expression of the corresponding IGS (Fig. 3a). Using this metric, we were able to quantitate the relative infiltration of each immune tissue on a patient-by-patient basis and test them for correlation with AA severity (Supplemental Fig. 2). Of the infiltrates tested, only ranking of CD8 T-cells and natural killer cells had sufficient power to segregate NC from AAP or AT/AU. Ranking by CD8 activity produced a dose-dependent separation between the three clinical presentations, significantly separating the three populations (hashes represent the medians of each cohort). NK-specific markers did not mirror the power of CD8 T cell-specific markers, indicating that the correlation is not likely the result of NK infiltrates or shared NK/CD8 T cell genes.

Using the IGS metrics, we also estimated the overall infiltrate signal within the AAP samples (Fig. 3b, left), and the AT/AU samples (Fig. 3b, right). The overall estimated changes in infiltration of each immune tissue type are also presented (Fig. 3b, chart). From the gene expression data, we observed an estimated immune infiltrate burden of 0.8–1.4%, correlating with increased clinical severity of AA. Concordantly, CD8^+^ infiltrates consisted of greater than 65% of the total infiltrate density only in samples from AAP or AT/AU patients. The absolute change in each immune tissue infiltrate across the three presentations is also shown (Fig. 3c), indicating that only CD8^+^ infiltrates change significantly across the three populations. These results indicate that although there is some expression-based evidence for multiple infiltrating tissue types, the most significantly abundant cell type associated with AA is non-NK, CD8^+^ cells. In addition, we detected elevated levels of markers associated with macrophages, total CD4^+^ T cells, CD4^+^ T cell subsets, NK cells and B cells, though these represented minor fractions compared to the CD8 T cell fraction.

Furthermore, we used the IGS scores to estimate the relative Th1 and Th2 fractions detected in patient samples (Fig. 3d). For each patient (AA or unaffected control), we represented the Th load within the sample biopsy as a ratio of Th1:Th2 signal, and observed that AA patient samples exhibit a shift to higher Th1 ratios compared to normal controls. The rank shift of Th1:Th2 associated with AA presentation was statistically significant by the Mann–Whitney U-test (p = 1.02 × 10^− 4^) indicating that, on the whole, skin from AA patients contains elevated levels of Th1 signatures relative to Th2 signatures as compared with unaffected patients, though there are AA patients with both Th1 and Th2 signatures.

### 3.4 ALADIN Scores Correlate With Disease Phenotype

**Fig. 4:**
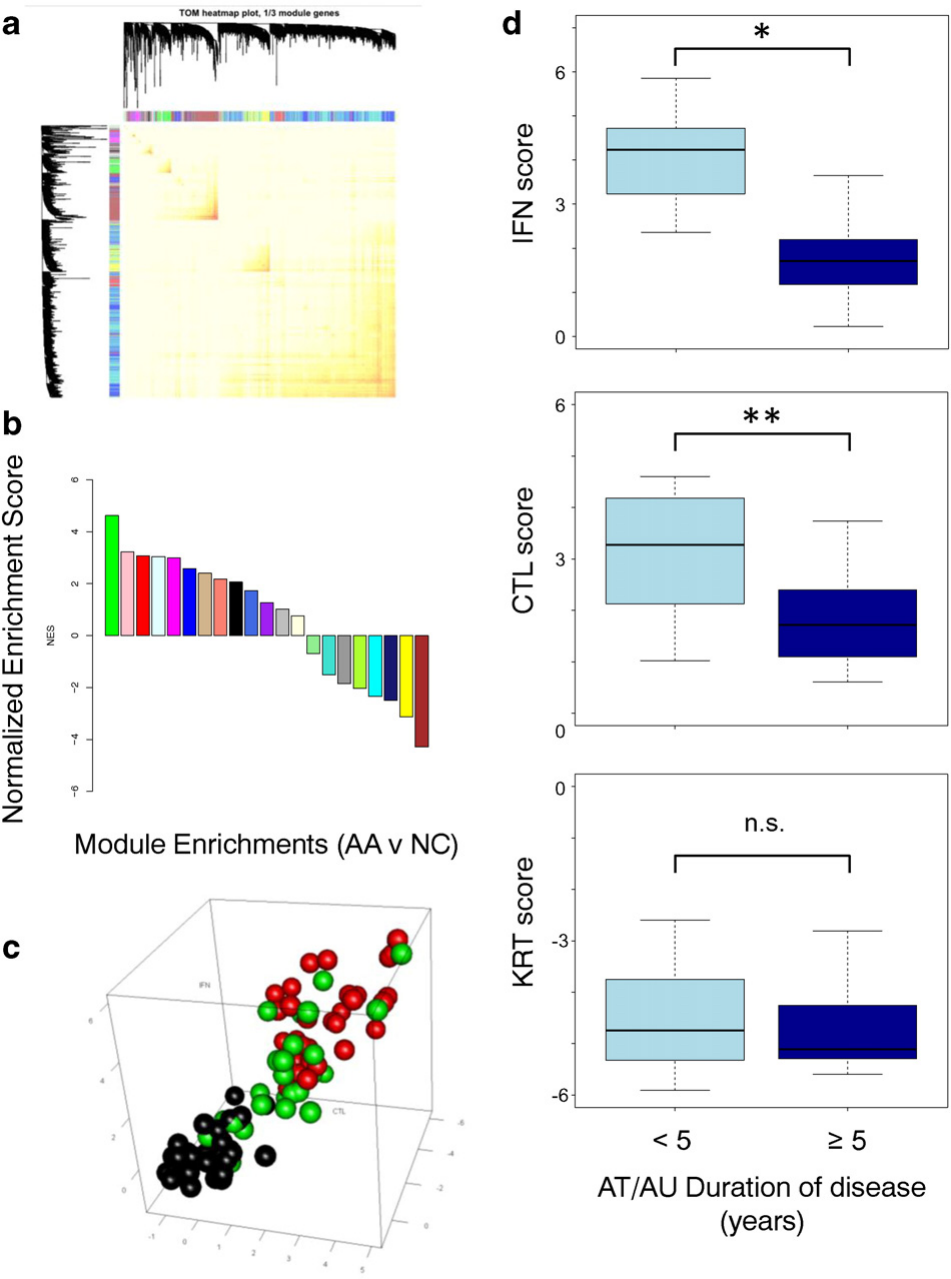
ALADIN scores parallels disease phenotype. (a) Co-expression analysis of the genes differentially expressed between AA and healthy controls reveals 20 modules of genes. (b) GSEA of all 20 genes modules for enrichment in significant differential expression between AA and controls reveals that the green and brown modules are most highly enriched in comparisons. (c) The ALADIN score classifies patient samples in three dimensions integrating immune infiltration and structural changes reflected by gene expression to identify relative risk of AA severity in patients (Black: NC, Green: AAP, Red: AT/AU). (d) CTL (top panel), IFN (middle panel), and KRT (bottom panel) signature scores from patients with AU/AT with respect to disease duration.

One of the goals of this study was to generate a metric that identified the most prominent features of the AA disease signature that would allow for a quantitative assessment of disease status. Weighted gene co-expression analysis (WGCNA) of the genes differentially expressed between AA and healthy controls revealed 20 clusters of co-expressed genes (Fig. 4a). These gene sets represent co-expressed modules and indicate the possibility of co-regulation, shared biological function, and/or shared pathways. For each of these modules we defined color-coded eigengenes, or metagenes, using the first principal component of the gene expression signature derived from the genes within each module. Gene set enrichment analysis (GSEA) of these modules with ranked lists of genes that were differentially expressed between AA and NC cohorts, as well as tests of association between module metagenes and disease phenotype revealed that the green and brown modules are the most significantly associated with disease phenotype (Fig. 4b, Supplemental Fig. 3a, b). These contain immune and immune response signatures (green) and structural keratins (brown).

In our earlier work, we developed an original scoring system, the Alopecia Areata Disease Activity Index (ALADIN), a three-dimensional quantitative composite gene expression score, for potential use as a biomarker for tracking disease severity and response to treatment (Xing et al., 2014). The metric scores patients along a combination of cytotoxic T lymphocyte infiltration (CTL), IFN-associated markers (IFN), and a hair keratin panel (KRT). Interestingly, the CTL signature contains the two genes, CD8A and PRF1, which are found in the CD8 T-cell signature referenced above (Fig. 3). Inspection of the components of the green module revealed the presence of genes contained in both the ALADIN CTL and IFN signatures, and the brown signature contained the genes that made up the ALADIN KRT signature (Supplemental Table 8). Statistically significant differences were found between AU/AT, AAP, and normal control sample groups for all three scores (Supplemental Fig. 4). A three-dimensional plot of the ALADIN scores for the combined discovery and validation dataset of 96 AT/AU, AAP, and NC samples showed that AT/AU samples clustered farthest away from NC samples, with AAP samples positioned in an intermediate position between both of these sets (Fig. 4c). A subsequent GSEA showed statistically significant enrichment of the original ALADIN gene sets in both AAP and AT/AU cohorts samples compared with normal controls (Supplemental Fig. 3c).

Spurred by previous work that described decreased immune infiltrates among skin samples from patients with chronic disease when compared with those from patients with acute disease (Whiting, 2003a), we assessed whether or not the duration of disease influenced the ALADIN score. Skin samples from AT/AU patients with 5 or more years of disease exhibited statistically significant decreases in IFN and CTL scores when compared with samples from AT/AU patients of shorter duration (Fig. 4d). This relationship was not seen between long- and short-duration AAP samples (Supplemental Fig. 5). These data indicate that the ALADIN score may distinguish AA forms that differ in severity, and, further, that inflammatory and immune infiltrate scores diminish among the more severe forms of AA over time.

## 4 Discussion

Here, we have utilized microarray based whole genome gene expression assays to generate new fundamental insights into the biology of AA. Our work here includes the use of scalp skin biopsy specimens from 60 patients with AA and 36 healthy controls. While other prior studies using microarrays in human AA have been published by our group and others (Xing et al., 2014, Carroll et al., 2002, Subramanya et al., 2010, Suárez-Fariñas et al., 2015), here we studied a larger cohort of subject samples and identify several critical features of disease pathogenesis.

First, AT/AU exhibits a high level of immune activity compared with normal controls and AAP samples. The notion held by some dermatologists that patients with longstanding AT/AU have lost the ability to regrow hair likely stems from a historical difficulty in treating these patients with previously available topical and oral medications (Alkhalifah et al., 2010) and difficulty in identifying appreciable numbers of rudimentary hairs in skin biopsy specimens of patients with severe disease. However, our data challenge this idea by providing evidence for sustained high levels of immunological activity in AT/AU samples that is greater than that seen in AAP. This immune activity in patients with AT/AU implies that a sufficiently strong immunosuppressant or treatment targeting a pathway necessary for the maintenance of the immune response may indeed be efficacious for these types of patients. Indeed, reports exist of use of deep penetrating ultraviolet light A treatment in combination with a photosensitizing agent can be effective in severe forms of AA (Claudy and Gagnaire, 1983, Ito et al., 2009). Furthermore, as supported by others (Whiting, 2003b), the immune activity in AU/AT samples diminishes over time, although we did not observe the same relationship among AAP samples. Additionally, our recent mechanistic data have supported a role for Janus kinase-mediated pathways in AA (Xing et al., 2014, Jabbari et al., 2015), and several additional case reports have corroborated that small molecule JAK inhibitors appear to be a promising class of drugs for AA, even in cases of severe or widespread disease (Higgins et al., 2015, Pieri et al., 2015, Craiglow and King, 2014). Of note, our analysis identified upregulation of the Jak-STAT pathway in AAP but not AU/AT samples, which may be due to gene expression variance observed within the AU/AT sample set.

Second, the molecular definition of AA supports a prominent role for CD8 T cells in the pathogenesis of the human disease. Progressively increasing gene expression signatures for CD8 T cells is seen when comparing NC, AAP and AT/AU samples. Previous studies by our group (Xing et al., 2014) and others (McElwee et al., 2005) have shown that CD8 T cells are necessary and sufficient in a mouse model of AA, and our prior GWAS study (Petukhova et al., 2010) implicated a role for CD8 T cells, by virtue of expression of NKG2D and the association found between AA and NKG2DL, in AA pathogenesis. The role of other cell types, including NK cells that may play a regulatory role in AA, has recently been assessed (Kaufman et al., 2010), and our data confirm that NK cells are indeed associated with the presence of disease in AA. Our data not only corroborate a role for CD8 T cells in the pathogenesis of disease, but further draws a correlation between the level of CD8 T cell density and disease severity/phenotype.

Third, the relative contribution of Th1 cells to disease pathogenesis in AA appears to be greater than that for other T-helper subtypes. Our data as well as work by others (Suárez-Fariñas et al., 2015) support the notion that the T cell infiltrate is composed of a mixed population of T helper cells. In fact, some samples in our set showed upregulation of Th1, Th2 and Th17 signatures simultaneously. However, our analysis here indicates that a predominant Th1 signature is more often seen among AA samples, and a Th1 signature is seen at a higher frequency in AA skin samples than in the normal control samples. The interactions and contributions of these separate T helper cell populations has yet to be fully investigated, although our data would indicate that targeting Th1 cells or cell signatures would likely be useful to a larger proportion of AA patients.

Finally, our analysis of gene expression among samples from a range of AA phenotypes led to the development of the ALADIN metric. We have previously shown use of this tool in case reports describing treatment of AA patients with JAK inhibitors (Xing et al., 2014, Jabbari et al., 2015), and we have shown here the ability of this multifactorial tool at distinguishing the milder AAP patient samples from the more severe AU/AT phenotype. Future work will examine the utility of ALADIN in the context of clinical trials for AA. For example, a set of biomarkers from a baseline skin biopsy or a biopsy early after the initiation of a particular treatment that could predict treatment response or, possibly more importantly, failure, preventing unnecessary exposure to potential side effects would indeed be a useful tool.

This study establishes a molecular definition of the AA specific disease process in the skin and may be interrogated for signatures corresponding to signaling mediators or cellular participants. These data serve as a rich resource for investigators pursuing pathogenic disease mechanisms and therapeutic targets in AA.

## Author Contributions

AJ, JEC, and JCC were responsible in large part for performing the studies reported herein and participated in the design, execution, and interpretation of the data. JMW, MD, VP, MH and DN were responsible for the recruitment and classification of study subjects as well as the procurement of tissue samples. AJ, RC, AMC were responsible for conception, design, oversight, execution, and interpretation of the data for this study. All authors contributed to the drafts, writing, figure preparation and editing of the final manuscript.

## Disclosures

Conflict of interests: none

## Appendix A Supplementary data

Supplementary material. Image 1
