# Supplementary material for: Molecular signatures define alopecia areata subtypes and transcriptional biomarkers
Source: eBioMedicine. 2016 Mar 31;7:240–7. doi: 10.1016/j.ebiom.2016.03.036 (PMC4909368; doi:10.1016/j.ebiom.2016.03.036)
Supplement: Supplementary file 1 — Supplementary material. [file mmc1.pdf]

Supplemental Materials

Supplemental Materials and Methods

Generating a linear Euclidean classifier for AA severity

Principal component analysis and terrain mapping of the AA disease signature revealed a near-linear dependency between NC, AAP, and AT/AU patients in an expression space defined by the first two principal components (PCs). The expression terrain map was generated with the MeV software suite using Euclidean distance as a metric and 10 nearest-neighbors as a clustering parameter. In order to convert this into a more intuitive, numeric score predictive of severity, we generated a list of genes that significantly contributed to PC1 and PC2. This was done by rank-sorting the genes' weighted contributions to each PC and selecting the set of genes before the inflection point of the weight distribution. The expression vectors of these genes were then z-score transformed and rank-normalized to generate non-zero, statistically comparable expression values. On a patient-by-patient basis, all genes in each normalized vector were used to construct centroid values in the appropriate PC vector. Each centroid value subsequently corresponds to a cardinal point in a grid defined as PC1xPC2 for each patient. The linear distance between each centroid (each patient's molecular state regarding AA) and the origin $PC1_{min}$ x $PC2_{min}$ was obtained by Euclidean distance to created a single scalar representation of that sample's deviation from an unaffected molecular state within the AA signature space. The vector was then quantile-normalized to bind the values between 0 and 10 for presentation.

Generating the consensus Immune Gene Signatures

Unique signature genes for each of the infiltrate populations were adopted from a previous work characterizing infiltration in cancers based on expression profiling (1). To generate relative classifiers ranking infiltration, each group of mutually exclusive genes were tested for co-segregation and classification power independently (Supplemental Fig 4) before being integrated into a consensus score for each individual patient. Integration was done in the following steps: z-score transforming all gene vectors to obtain scale-comparable expression values; rank-transforming these vectors to obtain ordered, non-negative values for each gene signal; and finally averaging over the ranks to create a consensus value of the rank-ordered expression for each infiltrating tissue. For the estimation of total infiltrate load per sample, the consensus z-score was transformed back into expression space for each individual gene and normalized to the consensus of housekeeping genes with the minimum coefficient of variation across the population, in a patient-by-patient basis.

The following table shows the signatures for each cell type:

Cell	Symbol	Entrez	Probe
aDCs	CCL1	6346	207533_at
aDCs	CD83	9308	204440_at
aDCs	LAMP3	27074	205569_at
B-cells	BLK	640	210934_at
B-cells	CD19	930	206398_s_at
B-cells	MS4A1	931	228599_at
DCs	CCL13	6357	216714_at
DCs	CCL17	6361	207900_at

DCs	CCL22	6367	207861_at
DCs	CD209	30835	207278_s_at
Eosinophils	CCR3	1232	208304_at
Eosinophils	GPR44	11251	216464_x_at
Eosinophils	IL5RA	3568	211517_s_at
iDCs	CD1A	909	210325_at
iDCs	CD1E	913	215784_at
Macrophages	CCL7	6354	208075_s_at
Macrophages	CXCL5	6374	215101_s_at
Macrophages	FN1	2335	216442_x_at
Macrophages	MSR1	4481	214770_at
Macrophages	PPBP	5473	214146_s_at
mast cells	CMA1	1215	214533_at
mast cells	MS4A2	2206	207497_s_at
mast cells	TPSAB1	7177	216485_s_at
Neutrophils	FPRL1	2358	210773_s_at
Neutrophils	IL8RA	3577	207094_at
Neutrophils	IL8RB	3579	207008_at
NK cells	NCR1	9437	217095_x_at
NK cells	XCL1	6375	206366_x_at
Normal	DCN	1634	242605_at
pDC	CLEC4C	170482	1555687_a_at
T-cells	CD2	914	205831_at
T-cells	CD247	919	210031_at
T-cells	CD28	940	211861_x_at
T-cells	CD3E	916	205456_at
T-cells	CD3G	917	206804_at
T-cells	CD6	923	213958_at
T-cells	IL2RB	3560	205291_at
T-cells	ZAP70	7535	214032_at
CD8 T-cells	CD8A	925	205758_at
CD8 T-cells	PRF1	5551	214617_at
Cytotoxic cells	KLRF1	51348	220646_s_at
Cytotoxic cells	GNLY	10578	37145_at
Cytotoxic cells	GZMA	3001	205488_at
Cytotoxic cells	GZMH	2999	210321_at
Cytotoxic cells	GZMK	3003	206666_at
Tem	LTK	4058	217184_s_at
Tem	NFATC4	4776	236270_at

Th	CD4	920	216424_at
Th1	CD38	952	205692_s_at
Th1	CSF2	1437	210229_s_at
Th1	CTLA4	1493	236341_at
Th1	IFNG	3458	210354_at
Th1	IL12RB2	3595	206999_at
Th1	LTA	4049	206975_at
Th1	STAT4	6775	206118_at
Th1	TBX21	30009	220684_at
Th17	IL17A	3605	216876_s_at
Th17	RORC	6097	228806_at
Th2	CXCR6	10663	211469_s_at
Th2	GATA3	2625	209604_s_at
Th2	IL26	55801	221111_at
Th2	LAIR2	3904	207509_s_at
Th2	PMCH	5367	206942_s_at
Th2	SMAD2	4087	239271_at
Tfh	BLR1	643	216734_s_at
Tfh	CXCL13	10563	205242_at
Tfh/Th2	MAF	4094	209348_s_at
Tfh	PDCD1	5133	207634_at
Treg	FOXP3	50943	224211_at

Unsupervised Machine Learning, Weighted Gene Co-Expression Analysis

Weighted Gene Co-expression Analysis (WGCNA) (2) was performed on the PSIDs in the ComBat adjusted expression set whose variance exceeded the median of the variances of all the PSIDs. Adjacency between PSIDs was defined as the Pearson correlation of the expression profiles across samples raised to a soft-thresholding power equal which was set to 10. The resulting adjacency matrix was transformed into a Topological Overlap matrix (TOM) from which the dissimilarity matrix was calculated. Hierarchical clustering was performed on the

dissimilarity matrix and modules were identified from the resulting branches of the dendrogram (2). The coexpression heatmap and dendrogram in Fig 5 were created from stratified sampling of 1/3 of the PSIDs assigned to the 20 modules using TOM as the adjacency measure. Kruskal-Wallis tests were performed to test for association between module eigengenes and the categorical traits disease phenotype, gender, and originating NAAF site. Pearson's correlation coefficient and p-values were estimated between each module eigengene and subject age. Gene Set Enrichment Analysis (GSEA) (3, 4) was used to further test for overrepresentation of genes over/under expressed in AA with respect to Normal controls in the different WGCNA modules. The normalized enrichment score (NES) reflects the degree to which a gene set is overrepresented at the top or bottom of a ranked list of genes taking into account differences in module size. Preranked gene lists were created with the t-statistic for ranking.

Supplemental Methods References

1. Bindea G et al. (2013) Spatiotemporal dynamics of intratumoral immune cells reveal the immune landscape in human cancer. *Immunity* 39:782–795.

2. Langfelder P, Horvath S (2008) WGCNA: an R package for weighted correlation network analysis. *BMC Bioinformatics* 9:559.

3. Subramanian A et al. (2005) Gene set enrichment analysis: a knowledge-based approach for interpreting genome-wide expression profiles. *Proc Natl Acad Sci USA* 102:15545–15550.

4. Mootha VK et al. (2003) PGC-1α-responsive genes involved in oxidative phosphorylation are coordinately downregulated in human diabetes. *Nat Genet* 34:267–273.

Supplemental Fig 1

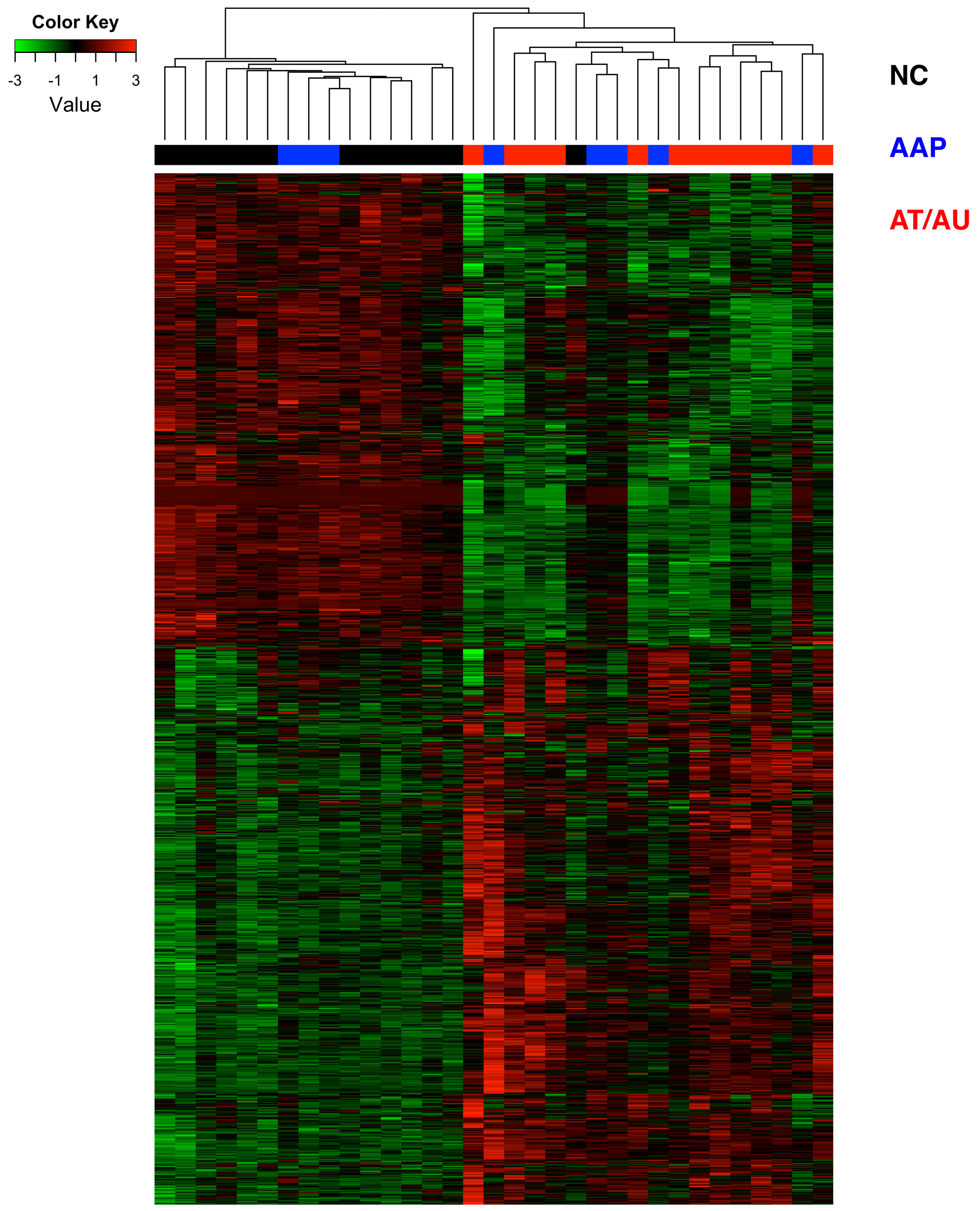

Supplemental Fig 1. AA Validation Set. Dendrogram and heatmap of the 33 samples in the validation dataset. Hierarchical clustering using Euclidean distance and average linkage was performed using the 2002 Affymetrix PSIDs that were identified as differentially expressed between AA patients and normal controls in the Discovery dataset were used to cluster the samples.

Supplemental Fig 2

a

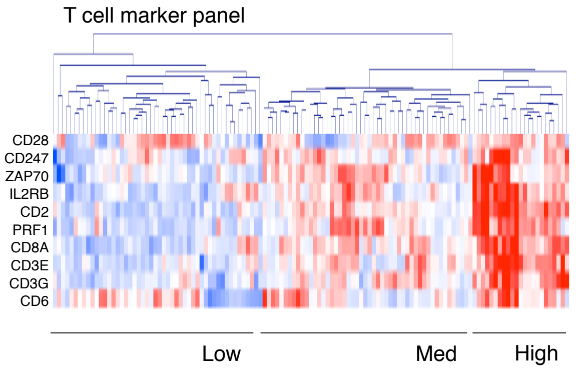

b

%	Low	Med	High
N	72	26	2
AAP	41	47	12
AT/AU	22	33	45

$\chi^2 = 33.36$
$p = 1x10^{-6}$

Supplemental Fig 2. T cell immune gene signature among AA samples. (**a**) Unsupervised consensus clustering of AA patients and unaffected controls using signature genes unique to each infiltrating immune tissue allows for the relative quantification of infiltrates in each sample. In this heatmap, red indicates higher expression and white indicates lower expression. Three main superclusters are demarcated as Low, Medium (Med), and High relative levels of infiltration based on marker expression. (**b**) The three infiltration superclusters are statistically significantly correlated with prognosis. A 3x3 chi-squared test reveals that the severity of infiltration is predictive of the severity of the AA phenotype across these patients. The numbers displayed in each cell represents the percentage of each clinical presentation that is found in the accompanying supercluster, e.g., 72% of NC samples were found in the Low cluster. The chi-squared statistic and accompanying p-value are provided.

Supplemental Fig 3

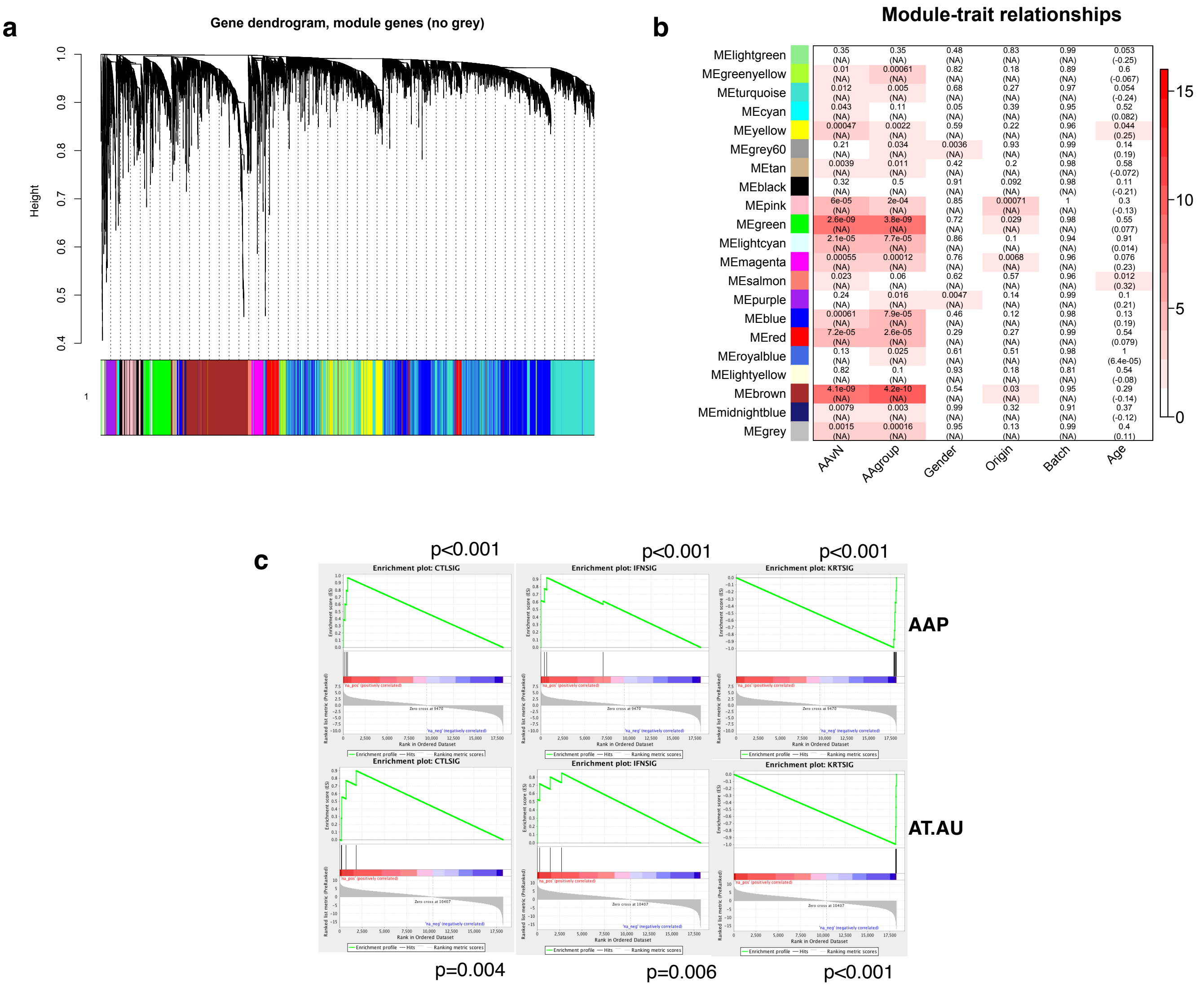

Supplemental Fig 3. Modules in AA disease specific signature define ALADIN components. (**a**) A dendrogram reflecting the gene co-expression clustering results. Along the bottom the colored barcode indicates the divisions that identified the 20 co-expressed modules used in this work. (**b**) A table of results when testing several clinical traits for association with the twenty modules. Displayed in each cell are the p-values for association between the corresponding module and trait. Cells are colored in increasing red to correspond to the significance of the association. (**c**) Gene Set Enrichment Analyses testing for statistical enrichment of each of the original ALADIn pathways in the AA cohort. In all comparisons against unaffected controls, there was statistical enrichment of the genes in the IFN, CTL, and KRT pathways in the direction expected (IFN and CTL are positively enriched, KRT is negatively enriched).

Supplemental Fig 4

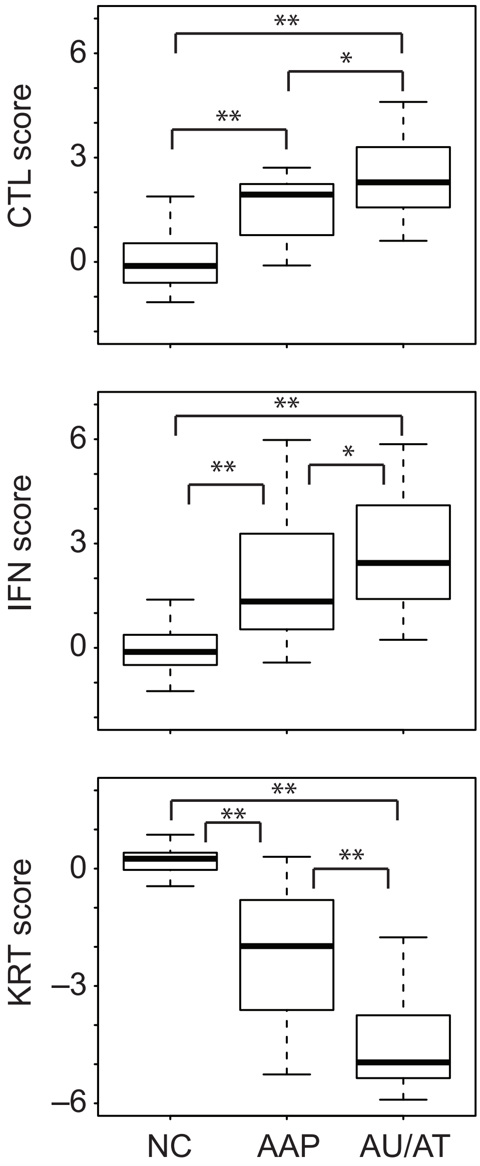

Supplemental Fig 4. ALADIN components differentiate AA phenotypes and normal controls. CTL (top panel), IFN (middle panel), and KRT (bottom panel) components of ALADIN were compared among normal control, AAP, and AU/AT samples. * $p < 0.05$; ** $p < 0.0001$

Supplemental Fig 5

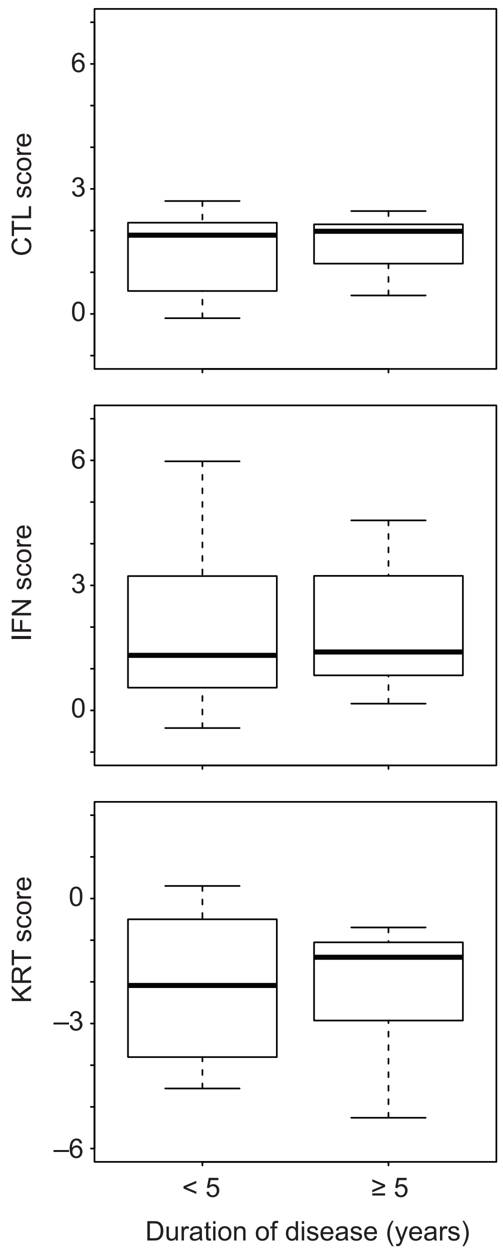

Supplemental Fig 5. Duration does not significantly influence ALADIN component scores among AAP patients. CTL (top panel), IFN (middle panel), and KRT (bottom panel) components of ALADIN were compared among AAP patients with less than 5 years duration or at least 5 years duration. No significant differences were found.

Supplemental Tables

Supplemental Table 1. Patient Demographics

Demographics for AT/AU, AAP/AAT, Normal samples

		Discovery Set	Validation Set	Total
Number (Proportion) of Patients by phenotype	AT/AU	20 (0.317)	12 (0.364)	32 (0.333)
	AAP/AAT (perilesional)	20 (0.317)	8 (0.242)	28 (0.292)
	Normal Controls	23 (0.365)	13 (0394)	36 (0.375)
	Total	63	33	96
Number (Proportion) of Females by Phenotype	AT/AU	14 (0.7)	10 (0.833)	24 (0.75)
	AAP/AAT (perilesional)	11 (0.55)	6 (0.75)	17 (0.607)
	Normal Controls	14 (0.61)	9 (0.692)	23 (0.639)
	Total	39 (0.619)	25 (0.758)	64 (0.667)
Number (Proportion) of Samples by Center	Columbia	37 (0.587)	15 (0.455)	52 (0.542)
	Houston	17 (0.270)	5 (0.152)	22 (0.229)
	Minnesota	7 (0.111)	3 (0.0909)	10 (0.104)
	San Francisco	2 (0.0317)	10 (0.303)	12 (0.125)
	Total	63	33	96
Mean Age in yrs [range] by phenotype	AT/AU	42.1 [18,67]	45.6 [22,66]	43.4 [18,67]
	AAP/AAT	37.3 [26,55]	41.9 [16,66]	38.6 [16,66]
	Normal Controls	38.5 [23,61]	36.3 [23,53]	37.7 [23,61]
	All samples	39.3 [18,67]	41.0 [16,66]	39.89 [16,67]
Mean Lesion Duration in yrs [range] by phenotype	AT/AU	10.30 [0.083,37]	14.17 [1,37]	11.75 [0.083,37]
	AAP/AAT	8.53 [0.019,39]	5.27 [0.167,20]	7.60 [0.019,39]
	All AA samples	9.42 [0.019,39]	10.61 [0.167,37]	9.81 [0.019,39]

Supplemental Table 2. AA v NC Differential Expression

ProbeSetID	GeneSymbol	logFC	F	p.value	BH_fdr
203915_at	CXCL9	3.57	11.9	3.40E-12	8.26E-10
204533_at	CXCL10	3.08	8.48	1.50E-10	2.01E-08
209728_at	HLA-DRB4	3.01	8.04	5.24E-04	3.92E-03
204580_at	MMP12	2.86	7.27	1.92E-08	1.13E-06
209924_at	CCL18 /// LOC101060271	2.77	6.82	5.73E-09	4.06E-07
32128_at	CCL18 /// LOC101060271	2.72	6.59	1.89E-08	1.11E-06
206407_s_at	CCL13	2.13	4.37	7.86E-11	1.21E-08
216714_at	CCL13	2.04	4.11	2.54E-10	3.12E-08
205758_at	CD8A	1.88	3.68	7.27E-11	1.14E-08
219386_s_at	SLAMF8	1.82	3.54	2.55E-12	6.55E-10
1405_i_at	CCL5	1.72	3.3	2.81E-12	7.07E-10
205821_at	KLRC4-KLRK1 /// KLRK1	1.7	3.26	1.58E-12	4.39E-10
213537_at	HLA-DPA1	1.69	3.23	6.01E-08	2.95E-06
204655_at	CCL5	1.68	3.21	1.04E-11	2.25E-09
205987_at	CD1C	1.66	3.17	1.33E-13	5.23E-11
206749_at	CD1B	1.64	3.12	3.14E-07	1.10E-05
205831_at	CD2	1.54	2.91	5.15E-10	5.64E-08
215666_at	HLA-DRB4	1.53	2.89	3.93E-03	1.82E-02
205488_at	GZMA	1.52	2.86	3.88E-08	2.02E-06
217147_s_at	TRAT1	1.51	2.85	1.92E-07	7.35E-06
1558972_s_at	THEMIS	1.46	2.75	1.49E-08	9.14E-07
229641_at	CCBE1	1.43	2.69	6.99E-10	7.22E-08
216935_at	LINC00302	1.43	2.69	1.37E-06	3.62E-05
229437_at	MIR155 /// MIR155HG	1.43	2.69	1.42E-08	8.78E-07
216718_at	LINC00302	1.42	2.67	4.19E-06	8.99E-05
206666_at	GZMK	1.4	2.65	7.06E-07	2.12E-05
206715_at	TFEC	1.4	2.64	1.38E-08	8.54E-07
213475_s_at	ITGAL	1.4	2.63	1.48E-09	1.32E-07
214617_at	PRF1	1.38	2.61	2.04E-07	7.69E-06
1559263_s_at	ZC3H12D	1.38	2.6	1.13E-11	2.37E-09
228592_at	MS4A1	1.37	2.59	1.16E-03	7.27E-03
220005_at	P2RY13	1.37	2.59	1.19E-10	1.69E-08
235221_at	CBLN3	1.36	2.57	1.76E-06	4.40E-05
1552870_s_at	AXDND1	1.36	2.56	1.66E-05	2.71E-04
238725_at	IRF1	1.34	2.54	1.86E-09	1.59E-07
210247_at	SYN2	1.33	2.51	6.59E-08	3.17E-06
206637_at	P2RY14	1.32	2.5	2.61E-12	6.64E-10
211657_at	CEACAM6	1.32	2.5	8.84E-06	1.63E-04
216920_s_at	TARP /// TRGC2	1.32	2.5	1.23E-06	3.32E-05
210146_x_at	LILRB2	1.31	2.48	1.17E-06	3.20E-05

204891_s_at	LCK	1.31	2.48	9.61E-10	9.50E-08
1552584_at	IL12RB1	1.31	2.48	5.29E-09	3.82E-07
214321_at	NOV	1.29	2.44	2.23E-08	1.28E-06
226218_at	IL7R	1.29	2.44	1.73E-07	6.73E-06
215049_x_at	CD163	1.29	2.44	3.98E-09	3.03E-07
210915_x_at	TRBC1	1.29	2.44	1.34E-09	1.23E-07
211796_s_at	TRBC1	1.29	2.44	8.37E-08	3.80E-06
219607_s_at	MS4A4A	1.28	2.44	8.69E-08	3.92E-06
211656_x_at	HLA-DQB1 /// LOC101060835	1.28	2.43	2.47E-09	2.01E-07
205890_s_at	GABBR1 /// UBD	1.28	2.43	1.60E-06	4.10E-05
216233_at	CD163	1.28	2.42	5.86E-08	2.89E-06
227346_at	IKZF1	1.27	2.42	5.64E-10	6.02E-08
206134_at	ADAMDEC1	1.27	2.41	1.86E-07	7.16E-06
236295_s_at	NLRC3	1.27	2.41	6.69E-09	4.62E-07
224555_x_at	IL37	1.27	2.41	1.97E-06	4.82E-05
204714_s_at	F5	1.26	2.4	1.44E-06	3.74E-05
213193_x_at	TRBC1	1.26	2.4	5.66E-09	4.02E-07
211339_s_at	ITK	1.26	2.4	2.97E-07	1.05E-05
210176_at	TLR1	1.26	2.4	9.76E-11	1.45E-08
228381_at	ATF7IP2 /// LOC100287628	1.26	2.4	1.37E-06	3.61E-05
213240_s_at	KRT4	1.26	2.39	1.63E-06	4.16E-05
227553_at	PIK3R5	1.25	2.38	4.09E-09	3.10E-07
244413_at	CLECL1	1.25	2.37	2.45E-08	1.39E-06
206545_at	CD28	1.23	2.35	1.96E-06	4.81E-05
203645_s_at	CD163	1.23	2.35	2.18E-08	1.26E-06
213539_at	CD3D	1.23	2.35	1.81E-10	2.34E-08
219890_at	CLEC5A	1.22	2.33	1.41E-07	5.75E-06
204205_at	APOBEC3G	1.22	2.33	2.85E-10	3.44E-08
231776_at	EOMES	1.22	2.33	7.97E-06	1.51E-04
227194_at	FAM3B	1.22	2.32	3.21E-07	1.12E-05
214038_at	CCL8	1.21	2.31	9.48E-07	2.68E-05
215806_x_at	TARP /// TRGC2	1.2	2.3	3.16E-06	7.11E-05
204869_at	PCSK2	1.2	2.3	7.12E-07	2.14E-05
204446_s_at	ALOX5	1.19	2.28	2.57E-07	9.26E-06
228362_s_at	FAM26F	1.19	2.28	3.98E-07	1.32E-05
1557570_a_at	LOC285084	1.19	2.28	3.34E-05	4.67E-04
206296_x_at	MAP4K1	1.18	2.27	6.23E-10	6.54E-08
215228_at	NHLH2	1.18	2.26	1.75E-05	2.82E-04
214219_x_at	MAP4K1	1.17	2.25	4.12E-10	4.69E-08
203757_s_at	CEACAM6	1.17	2.25	1.69E-05	2.75E-04
209823_x_at	HLA-DQB1 /// LOC101060835	1.17	2.25	8.27E-08	3.78E-06
236028_at	IBSP	1.17	2.25	5.52E-06	1.12E-04

228869_at	SNX20	1.16	2.24	3.28E-09	2.56E-07
205419_at	GPR183	1.16	2.23	2.96E-08	1.64E-06
206082_at	HCP5	1.16	2.23	1.44E-10	1.94E-08
230983_at	FAM129C	1.15	2.23	4.44E-05	5.80E-04
209606_at	CYTIP	1.15	2.22	7.01E-09	4.81E-07
235080_at	CLUAP1	1.15	2.22	3.07E-07	1.08E-05
221080_s_at	DENND1C	1.15	2.22	1.32E-07	5.46E-06
203416_at	CD53	1.15	2.22	4.54E-10	5.04E-08
211144_x_at	TARP /// TRGC2	1.15	2.21	2.48E-06	5.84E-05
210164_at	GZMB	1.14	2.21	8.32E-07	2.42E-05
227645_at	PIK3R5	1.14	2.21	1.18E-09	1.11E-07
236285_at	KLHDC7B	1.14	2.2	9.81E-06	1.77E-04
203471_s_at	PLEK	1.14	2.2	6.13E-09	4.29E-07
229779_at	COL4A4	1.14	2.2	5.20E-06	1.07E-04
214339_s_at	MAP4K1	1.13	2.19	1.40E-09	1.27E-07
230753_at	PATL2	1.13	2.19	3.51E-08	1.87E-06
205043_at	CFTR	1.13	2.19	1.72E-04	1.67E-03
206227_at	CILP	1.13	2.18	4.12E-05	5.47E-04
236175_at	TRIM55	1.12	2.18	1.82E-05	2.90E-04
221470_s_at	IL37	1.12	2.18	9.79E-05	1.07E-03
44673_at	SIGLEC1	1.12	2.18	1.53E-08	9.29E-07
228094_at	AMICA1	1.12	2.18	1.81E-10	2.34E-08
229391_s_at	FAM26F	1.12	2.17	5.03E-07	1.60E-05
1554748_at	CLCNKB	1.12	2.17	8.13E-05	9.37E-04
239529_at	C5orf20 /// TIFAB	1.12	2.17	1.32E-08	8.23E-07
236782_at	SAMD3	1.12	2.17	5.77E-06	1.16E-04
242641_at	COL6A6	1.12	2.17	4.11E-05	5.46E-04
220507_s_at	UPB1	1.12	2.17	2.15E-03	1.16E-02
209813_x_at	TARP	1.11	2.16	1.89E-06	4.66E-05
1564474_at	ZBED3-AS1	1.11	2.16	2.52E-05	3.76E-04
206761_at	CD96	1.11	2.15	6.85E-08	3.26E-06
214567_s_at	XCL1 /// XCL2	1.1	2.15	1.48E-07	5.93E-06
1555993_at	CACNA1D	1.1	2.14	1.52E-08	9.28E-07
229383_at	1-Mar	1.1	2.14	3.30E-09	2.57E-07
203761_at	SLA	1.1	2.14	4.62E-11	7.68E-09
214450_at	CTSW	1.1	2.14	1.53E-06	3.94E-05
214399_s_at	KRT4	1.1	2.14	5.25E-06	1.08E-04
1562364_at	GVINP1	1.1	2.14	1.12E-07	4.76E-06
205180_s_at	ADAM8	1.09	2.14	6.64E-08	3.18E-06
219666_at	MS4A6A	1.09	2.13	5.29E-09	3.82E-07
205364_at	ACOX2	1.09	2.13	2.93E-05	4.24E-04
213920_at	CUX2	1.09	2.13	3.89E-04	3.13E-03
230391_at	CD84	1.09	2.13	9.18E-08	4.08E-06

220485_s_at	SIRPG	1.09	2.13	4.34E-07	1.42E-05
205030_at	FABP7	1.09	2.13	1.38E-02	4.67E-02
207277_at	CD209	1.09	2.13	1.71E-08	1.03E-06
220330_s_at	SAMSN1	1.09	2.13	2.43E-05	3.66E-04
229151_at	SLC14A1	1.09	2.13	7.00E-03	2.81E-02
223710_at	CCL26	1.09	2.13	6.53E-05	7.85E-04
227266_s_at	FYB	1.09	2.12	2.67E-09	2.15E-07
207957_s_at	PRKCB	1.09	2.12	4.89E-07	1.57E-05
231124_x_at	LY9	1.08	2.12	8.53E-07	2.46E-05
207981_s_at	ESRRG	1.08	2.12	1.53E-05	2.53E-04
226136_at	GLIPR1	1.08	2.12	3.12E-09	2.46E-07
204440_at	CD83	1.08	2.12	8.25E-09	5.49E-07
210038_at	PRKCQ	1.08	2.12	8.08E-05	9.32E-04
206914_at	CRTAM	1.08	2.11	1.10E-07	4.70E-06
219385_at	SLAMF8	1.08	2.11	3.43E-08	1.84E-06
219820_at	SLC6A16	1.08	2.11	3.23E-07	1.12E-05
207651_at	GPR171	1.07	2.1	4.68E-06	9.80E-05
1553081_at	WFDC12	1.07	2.09	1.41E-03	8.44E-03
229510_at	MS4A14	1.06	2.09	6.04E-07	1.87E-05
220358_at	BATF3	1.06	2.09	1.83E-08	1.09E-06
205804_s_at	TRAF3IP3	1.06	2.09	2.50E-06	5.89E-05
214081_at	PLXDC1	1.06	2.08	2.83E-05	4.11E-04
230937_at	LOC285835	1.06	2.08	1.14E-05	2.00E-04
1556472_s_at	SCML4	1.05	2.08	7.81E-07	2.30E-05
232081_at	ABCG1	1.05	2.07	5.31E-09	3.82E-07
214913_at	ADAMTS3	1.05	2.07	7.57E-08	3.52E-06
228943_at	MAP6	1.05	2.07	1.23E-06	3.33E-05
227353_at	TMC8	1.05	2.06	2.77E-07	9.85E-06
241981_at	FAM20A	1.04	2.05	3.60E-06	7.94E-05
233052_at	DNAH8	1.03	2.05	1.73E-04	1.68E-03
243585_at	ATP13A5	1.03	2.05	3.03E-06	6.91E-05
206247_at	MICB	1.03	2.04	2.69E-07	9.66E-06
230110_at	MCOLN2	1.03	2.04	4.41E-06	9.37E-05
1564786_at	LOC338667	1.03	2.04	1.15E-03	7.22E-03
229390_at	FAM26F	1.03	2.04	7.53E-07	2.24E-05
206366_x_at	XCL1	1.03	2.04	2.86E-07	1.01E-05
1553062_at	MOGAT1	1.03	2.04	8.40E-05	9.62E-04
221978_at	HLA-F	1.03	2.04	1.11E-09	1.06E-07
240197_at	SYN2	1.02	2.03	4.60E-07	1.49E-05
1557474_at	LOC284578	1.02	2.03	9.53E-06	1.73E-04
242488_at	CHRM3	1.02	2.03	7.29E-04	5.07E-03
205269_at	LCP2	1.02	2.03	6.27E-08	3.04E-06

1559194_a_at	CLEC4GP1	1.02	2.03	4.11E-06	8.86E-05
242268_at	CELF2	1.02	2.03	2.02E-07	7.65E-06
214203_s_at	PRODH	1.02	2.03	1.42E-07	5.79E-06
213960_at	NTRK3	1.02	2.02	5.19E-05	6.58E-04
230418_s_at	GALNT16	1.02	2.02	6.98E-06	1.36E-04
219519_s_at	SIGLEC1	1.02	2.02	2.85E-07	1.01E-05
227742_at	CLIC6	1.01	2.02	1.91E-05	3.01E-04
204912_at	IL10RA	1.01	2.02	8.42E-12	1.86E-09
1560396_at	KLHL6	1.01	2.02	2.70E-05	3.97E-04
228532_at	C1orf162	1.01	2.02	1.76E-10	2.30E-08
227677_at	JAK3	1.01	2.01	3.81E-07	1.27E-05
208161_s_at	ABCC3	1.01	2.01	3.16E-10	3.72E-08
223343_at	MS4A7	1	2	1.72E-05	2.79E-04
209879_at	SELPLG	0.999	2	8.94E-09	5.85E-07
210184_at	ITGAX	0.998	2	1.95E-07	7.46E-06
231029_at	F5	0.995	1.99	7.31E-04	5.08E-03
202953_at	C1QB	0.994	1.99	4.46E-11	7.49E-09
209969_s_at	STAT1	0.991	1.99	5.19E-07	1.64E-05
205270_s_at	LCP2	0.991	1.99	9.12E-09	5.95E-07
217087_at	C1orf68	0.989	1.99	9.79E-04	6.41E-03
207802_at	CRISP3	0.989	1.99	5.17E-03	2.24E-02
205786_s_at	ITGAM	0.989	1.99	2.16E-07	8.07E-06
229241_at	LDHD	0.989	1.99	2.90E-05	4.20E-04
220560_at	C11orf21	0.989	1.98	3.29E-07	1.14E-05
209498_at	CEACAM1	0.988	1.98	8.73E-06	1.62E-04
244363_at	ROS1	0.986	1.98	2.98E-04	2.56E-03
211991_s_at	HLA-DPA1	0.985	1.98	4.04E-10	4.63E-08
206785_s_at	KLRC1 /// KLRC2	0.985	1.98	1.94E-07	7.43E-06
1566671_a_at	PDXK	0.984	1.98	2.35E-04	2.13E-03
204279_at	PSMB9	0.984	1.98	1.54E-09	1.36E-07
230550_at	MS4A6A	0.984	1.98	4.71E-07	1.52E-05
205979_at	SCGB2A1	0.983	1.98	1.30E-02	4.47E-02
205291_at	IL2RB	0.982	1.98	8.56E-07	2.47E-05
227142_at	PLEKHG5	0.981	1.97	2.36E-06	5.61E-05
204411_at	KIF21B	0.981	1.97	1.04E-05	1.86E-04
204994_at	MX2	0.98	1.97	3.17E-08	1.73E-06
208438_s_at	FGR	0.98	1.97	5.93E-09	4.17E-07
212671_s_at	HLA-DQA1 /// HLA-DQA2 /// LOC100509457	0.98	1.97	7.94E-05	9.20E-04
229499_at	CAPN13	0.98	1.97	4.26E-04	3.36E-03
207908_at	KRT2	0.978	1.97	3.66E-06	8.05E-05
204959_at	MNDA	0.978	1.97	2.37E-07	8.67E-06
239427_at	SLAMF1	0.977	1.97	1.72E-07	6.70E-06

244572_at	KY	0.976	1.97	5.04E-07	1.60E-05
226142_at	GLIPR1	0.973	1.96	5.00E-09	3.64E-07
209734_at	NCKAP1L	0.973	1.96	3.44E-11	5.97E-09
218795_at	ACP6	0.964	1.95	3.55E-07	1.20E-05
214873_at	LRP5L	0.964	1.95	1.54E-04	1.53E-03
235291_s_at	FLJ32255	0.963	1.95	6.61E-06	1.29E-04
209975_at	CYP2E1	0.962	1.95	8.47E-08	3.84E-06
204377_s_at	VPRBP	0.962	1.95	1.51E-06	3.89E-05
220801_s_at	HAO2	0.96	1.94	9.49E-04	6.26E-03
207861_at	CCL22	0.958	1.94	4.93E-06	1.03E-04
202524_s_at	SPOCK2	0.958	1.94	4.10E-06	8.85E-05
232428_at	MOGAT2	0.957	1.94	2.66E-04	2.35E-03
204777_s_at	MAL	0.957	1.94	1.71E-06	4.32E-05
214945_at	FAM153A /// FAM153B /// FAM153C /// LOC100507387	0.957	1.94	1.10E-03	6.96E-03
206069_s_at	ACADL	0.956	1.94	1.36E-04	1.39E-03
205101_at	CIITA	0.955	1.94	7.33E-09	5.01E-07
210889_s_at	FCGR2B	0.954	1.94	6.79E-10	7.02E-08
215133_s_at	FAM153A /// FAM153B /// LOC100507387 /// LOC100507427	0.953	1.94	2.52E-04	2.25E-03
219045_at	RHOF	0.952	1.94	9.58E-11	1.43E-08
206513_at	AIM2	0.952	1.93	9.37E-06	1.71E-04
208885_at	LCP1	0.951	1.93	4.28E-09	3.20E-07
235276_at	EPSTI1	0.946	1.93	5.74E-07	1.79E-05
236270_at	NFATC4	0.945	1.93	1.88E-07	7.25E-06
205280_at	GLRB	0.943	1.92	7.39E-07	2.21E-05
228573_at	ANTXR2	0.942	1.92	2.00E-06	4.90E-05
229723_at	TAGAP	0.942	1.92	1.90E-05	3.00E-04
202957_at	HCLS1	0.942	1.92	1.58E-08	9.50E-07
232581_x_at	HIVEP3	0.94	1.92	2.67E-05	3.93E-04
206978_at	CCR2	0.939	1.92	5.73E-06	1.15E-04
241418_at	LOC344887	0.939	1.92	3.88E-04	3.13E-03
207710_at	LCE2B	0.936	1.91	1.15E-04	1.22E-03
215784_at	CD1E	0.933	1.91	7.63E-05	8.93E-04
206214_at	PLA2G7	0.931	1.91	1.44E-05	2.42E-04
212975_at	DENND3	0.931	1.91	3.06E-06	6.95E-05
228836_at	SLC25A35	0.93	1.91	5.86E-05	7.21E-04
228298_at	PCED1B	0.929	1.9	6.12E-06	1.21E-04
206143_at	SLC26A3	0.928	1.9	1.41E-03	8.45E-03
206181_at	SLAMF1	0.928	1.9	3.59E-07	1.21E-05
227168_at	MIAT	0.927	1.9	4.33E-07	1.42E-05
223605_at	SLC25A18	0.926	1.9	4.14E-04	3.28E-03
201884_at	CEACAM5	0.925	1.9	7.40E-05	8.69E-04

235175_at	GBP4	0.923	1.9	3.65E-07	1.23E-05
204122_at	TYROBP	0.922	1.89	6.45E-09	4.48E-07
236081_at	SNCA	0.921	1.89	1.80E-05	2.87E-04
227210_at	SFMBT2	0.921	1.89	1.31E-08	8.18E-07
219452_at	DPEP2	0.916	1.89	2.65E-07	9.55E-06
230560_at	STXBP6	0.911	1.88	1.84E-06	4.56E-05
204160_s_at	ENPP4	0.91	1.88	2.37E-05	3.59E-04
229634_at	TMEM139	0.91	1.88	1.04E-05	1.87E-04
214012_at	ERAP1	0.909	1.88	1.67E-04	1.63E-03
223562_at	PARVG	0.909	1.88	8.36E-08	3.80E-06
226603_at	SAMD9L	0.908	1.88	1.54E-07	6.11E-06
229534_at	ACOT4	0.907	1.87	3.72E-06	8.17E-05
31637_s_at	NR1D1 /// THRA	0.905	1.87	8.14E-06	1.53E-04
212873_at	HMHA1	0.905	1.87	5.93E-08	2.91E-06
211200_s_at	EFCAB2	0.903	1.87	6.87E-04	4.83E-03
214044_at	RYR2	0.902	1.87	1.18E-05	2.05E-04
205686_s_at	CD86	0.902	1.87	1.57E-10	2.09E-08
218627_at	DRAM1	0.9	1.87	2.04E-08	1.19E-06
219319_at	HIF3A	0.899	1.86	1.02E-05	1.83E-04
219594_at	NINJ2	0.899	1.86	1.10E-09	1.06E-07
1554360_at	FCHSD2	0.897	1.86	3.05E-05	4.36E-04
210607_at	FLT3LG	0.897	1.86	7.44E-08	3.49E-06
212613_at	BTN3A2	0.895	1.86	7.95E-06	1.50E-04
224451_x_at	ARHGAP9	0.895	1.86	1.05E-06	2.92E-05
207466_at	GAL	0.894	1.86	1.32E-03	8.03E-03
206118_at	STAT4	0.892	1.86	4.12E-06	8.89E-05
217478_s_at	HLA-DMA	0.89	1.85	1.29E-11	2.63E-09
205098_at	CCR1	0.889	1.85	3.81E-08	2.00E-06
220577_at	GVINP1	0.889	1.85	6.62E-07	2.01E-05
205295_at	CKMT2	0.888	1.85	1.05E-06	2.92E-05
205432_at	OVGP1	0.887	1.85	1.07E-06	2.96E-05
210163_at	CXCL11	0.885	1.85	2.29E-04	2.09E-03
1556474_a_at	FLJ38379	0.884	1.85	5.80E-06	1.16E-04
227609_at	EPSTI1	0.884	1.85	1.70E-04	1.65E-03
229597_s_at	WDFY4	0.88	1.84	3.34E-07	1.15E-05
207430_s_at	MSMB	0.879	1.84	2.65E-03	1.36E-02
204760_s_at	NR1D1 /// THRA	0.877	1.84	4.49E-05	5.85E-04
219616_at	ACSS3	0.877	1.84	9.28E-07	2.64E-05
228057_at	DDIT4L	0.876	1.84	1.50E-05	2.50E-04
203892_at	WFDC2	0.876	1.84	6.60E-06	1.29E-04
206623_at	PDE6A	0.875	1.83	4.30E-04	3.38E-03
244385_at	KDM4C	0.875	1.83	8.88E-06	1.64E-04

236331_at	CDKL2	0.874	1.83	5.60E-04	4.13E-03
210538_s_at	BIRC3	0.874	1.83	4.71E-05	6.07E-04
204197_s_at	RUNX3	0.873	1.83	3.46E-11	5.97E-09
1561306_s_at	STRC	0.869	1.83	7.75E-05	9.03E-04
203485_at	RTN1	0.869	1.83	3.12E-06	7.06E-05
205768_s_at	SLC27A2	0.869	1.83	3.09E-04	2.63E-03
210439_at	ICOS	0.868	1.83	2.89E-05	4.19E-04
211734_s_at	FCER1A	0.868	1.82	8.20E-09	5.47E-07
230422_at	FPR3	0.868	1.82	6.40E-06	1.26E-04
213830_at	YME1L1	0.867	1.82	1.81E-03	1.01E-02
235486_at	KIAA1549L	0.865	1.82	2.70E-05	3.97E-04
206199_at	CEACAM7	0.865	1.82	1.40E-03	8.41E-03
204153_s_at	MFNG	0.864	1.82	9.19E-08	4.08E-06
209905_at	HOXA10-HOXA9 /// HOXA9 /// MIR196B	0.858	1.81	4.04E-06	8.75E-05
220780_at	PLA2G3	0.857	1.81	1.93E-05	3.03E-04
244598_at	LCP2	0.854	1.81	8.64E-07	2.49E-05
210026_s_at	CARD10	0.854	1.81	9.23E-06	1.69E-04
229497_at	ANKDD1A	0.853	1.81	1.65E-09	1.43E-07
231930_at	ELMOD1 /// LOC643923	0.853	1.81	5.43E-05	6.81E-04
208396_s_at	PDE1A	0.853	1.81	1.09E-05	1.93E-04
203828_s_at	IL32	0.853	1.81	2.24E-07	8.31E-06
244033_at	CEP128	0.851	1.8	6.60E-07	2.00E-05
212587_s_at	PTPRC	0.851	1.8	7.90E-06	1.50E-04
230025_at	GJD3	0.85	1.8	5.03E-07	1.60E-05
226474_at	NLRC5	0.85	1.8	9.72E-08	4.27E-06
205213_at	ACAP1	0.848	1.8	4.00E-08	2.08E-06
231093_at	FCRL3	0.848	1.8	1.18E-04	1.24E-03
226878_at	HLA-DOA	0.848	1.8	1.19E-06	3.23E-05
202531_at	IRF1	0.848	1.8	2.00E-07	7.63E-06
230773_at	ZNF385D	0.846	1.8	3.17E-05	4.49E-04
207955_at	CCL27	0.845	1.8	3.44E-04	2.85E-03
235343_at	VASH2	0.845	1.8	4.16E-06	8.93E-05
216701_at	C1orf68	0.844	1.79	1.65E-03	9.48E-03
209795_at	CD69	0.844	1.79	6.11E-03	2.54E-02
204259_at	MMP7	0.844	1.79	1.86E-06	4.60E-05
205456_at	CD3E	0.842	1.79	1.64E-07	6.44E-06
208121_s_at	PTPRO	0.842	1.79	5.27E-08	2.64E-06
226311_at	ADAMTS2	0.841	1.79	4.34E-05	5.70E-04
236234_at	PDE1A	0.841	1.79	4.00E-06	8.67E-05
220003_at	LRRC36	0.84	1.79	8.99E-05	1.01E-03
207901_at	IL12B	0.84	1.79	5.74E-05	7.10E-04
1553311_at	C20orf197	0.839	1.79	6.47E-07	1.98E-05

226818_at	MPEG1	0.838	1.79	8.14E-09	5.45E-07
205997_at	ADAM28	0.837	1.79	8.85E-08	3.97E-06
41577_at	PPP1R16B	0.837	1.79	1.13E-06	3.12E-05
215116_s_at	DNM1	0.837	1.79	7.30E-07	2.18E-05
1294_at	MIR5193 /// UBA7	0.836	1.79	4.70E-08	2.38E-06
208146_s_at	CPVL	0.836	1.78	1.35E-07	5.54E-06
210619_s_at	HYAL1	0.834	1.78	5.15E-04	3.86E-03
213723_s_at	IDUA	0.834	1.78	9.34E-05	1.04E-03
226248_s_at	KIAA1324	0.833	1.78	1.19E-04	1.25E-03
230799_at	LOC100134259	0.832	1.78	3.30E-05	4.62E-04
231015_at	KLF15	0.831	1.78	2.82E-06	6.48E-05
229354_at	AHRR	0.83	1.78	1.93E-05	3.04E-04
213888_s_at	TRAF3IP3	0.83	1.78	2.60E-05	3.85E-04
219992_at	TAC3	0.829	1.78	4.71E-04	3.62E-03
1552398_a_at	CLEC12A	0.829	1.78	9.88E-05	1.08E-03
241440_at	ZIC1	0.828	1.78	2.54E-04	2.26E-03
1561394_s_at	KIAA1755	0.827	1.77	2.32E-05	3.53E-04
235719_at	CYP4V2	0.827	1.77	2.23E-04	2.05E-03
209901_x_at	AIF1	0.827	1.77	3.04E-08	1.68E-06
242286_at	GRIN2A	0.827	1.77	7.87E-07	2.32E-05
1564475_s_at	ZBED3-AS1	0.825	1.77	6.77E-04	4.77E-03
226612_at	UBE2QL1	0.825	1.77	1.20E-07	5.06E-06
213845_at	GRIK2	0.824	1.77	2.43E-04	2.19E-03
204663_at	ME3	0.824	1.77	6.79E-06	1.32E-04
223691_at	RGS22	0.824	1.77	6.86E-05	8.19E-04
237870_at	NQO2	0.823	1.77	7.25E-04	5.05E-03
235574_at	GBP4	0.823	1.77	4.61E-07	1.50E-05
205859_at	LY86	0.823	1.77	1.72E-06	4.32E-05
215332_s_at	CD8B /// LOC100996919	0.823	1.77	1.27E-08	8.00E-07
220783_at	MMP27	0.82	1.77	6.14E-06	1.22E-04
233907_s_at	SERTAD4	0.82	1.77	2.55E-06	5.97E-05
208964_s_at	FADS1 /// MIR1908	0.819	1.76	3.32E-03	1.61E-02
225598_at	SLC45A4	0.819	1.76	1.23E-06	3.31E-05
238039_at	LOC728769	0.818	1.76	1.12E-07	4.77E-06
209785_s_at	PLA2G4C	0.818	1.76	4.95E-05	6.32E-04
239237_at	LOC100506776	0.817	1.76	1.28E-04	1.33E-03
207072_at	IL18RAP	0.816	1.76	2.18E-05	3.35E-04
206186_at	MPP3	0.815	1.76	4.95E-06	1.03E-04
228258_at	TBC1D10C	0.813	1.76	1.25E-07	5.20E-06
239787_at	KCTD4	0.812	1.76	3.48E-03	1.67E-02
223280_x_at	MS4A6A	0.812	1.76	8.71E-08	3.93E-06
226436_at	RASSF4	0.812	1.76	2.46E-07	8.91E-06

213603_s_at	RAC2	0.811	1.75	2.12E-08	1.23E-06
238983_at	NSUN7	0.81	1.75	1.95E-04	1.85E-03
227183_at	MIR143HG /// MIR145	0.81	1.75	1.06E-03	6.81E-03
238229_at	TMEM67	0.81	1.75	6.94E-04	4.87E-03
212444_at	GPRC5A	0.809	1.75	7.73E-05	9.01E-04
228821_at	ST6GAL2	0.808	1.75	2.05E-04	1.92E-03
229088_at	ENPP1	0.807	1.75	1.19E-05	2.07E-04
227647_at	KCNE3	0.807	1.75	1.00E-04	1.09E-03
205769_at	SLC27A2	0.806	1.75	8.07E-04	5.48E-03
210665_at	TFPI	0.806	1.75	2.02E-07	7.65E-06
231549_at	C1orf158	0.805	1.75	1.26E-02	4.35E-02
236995_x_at	TFEC	0.805	1.75	1.53E-07	6.11E-06
205992_s_at	IL15	0.805	1.75	3.40E-08	1.83E-06
244297_at	ANKRD18A	0.804	1.75	2.23E-04	2.05E-03
243601_at	LOC285957	0.803	1.74	7.67E-08	3.55E-06
219704_at	YBX2	0.803	1.74	1.31E-04	1.35E-03
232311_at	B2M	0.801	1.74	1.24E-07	5.16E-06
224356_x_at	MS4A6A	0.8	1.74	9.48E-08	4.18E-06
211138_s_at	KMO	0.799	1.74	6.03E-08	2.95E-06
209488_s_at	RBPMS	0.799	1.74	3.45E-05	4.79E-04
212970_at	APBB2	0.799	1.74	1.74E-06	4.36E-05
222088_s_at	SLC2A14 /// SLC2A3	0.797	1.74	4.45E-05	5.80E-04
236253_at	ZNF546	0.797	1.74	3.11E-06	7.04E-05
203895_at	PLCB4	0.797	1.74	1.60E-04	1.58E-03
214981_at	POSTN	0.795	1.74	2.22E-06	5.32E-05
236886_at	LOC100049716	0.795	1.73	2.68E-05	3.94E-04
204114_at	NID2	0.794	1.73	7.70E-06	1.47E-04
236150_at	HYKK	0.794	1.73	6.48E-06	1.27E-04
224357_s_at	MS4A4A	0.793	1.73	3.55E-07	1.20E-05
1568794_at	LOC643355	0.793	1.73	1.24E-04	1.30E-03
229619_at	FOLR2	0.792	1.73	4.44E-06	9.40E-05
1554906_a_at	MPHOSPH6	0.792	1.73	4.18E-04	3.31E-03
235742_at	RHOC	0.791	1.73	3.48E-08	1.86E-06
221142_s_at	PECR	0.791	1.73	2.08E-03	1.13E-02
228342_s_at	ALPK3	0.791	1.73	3.30E-03	1.60E-02
206932_at	CH25H	0.79	1.73	1.01E-04	1.10E-03
235533_at	COX19	0.789	1.73	9.11E-05	1.02E-03
219183_s_at	CYTH4	0.789	1.73	1.38E-06	3.64E-05
234722_x_at	OBP2B	0.788	1.73	1.78E-04	1.72E-03
232617_at	CTSS	0.788	1.73	1.81E-07	6.99E-06
201743_at	CD14	0.788	1.73	8.99E-08	4.02E-06
230782_at	SORD	0.788	1.73	4.02E-08	2.09E-06

1553772_at	GK5	0.786	1.72	2.65E-06	6.16E-05
220428_at	CD207	0.786	1.72	1.21E-05	2.11E-04
239272_at	MMP28	0.786	1.72	9.37E-05	1.04E-03
239380_at	C5orf27	0.785	1.72	7.77E-05	9.05E-04
235557_at	GPAT2	0.785	1.72	4.52E-04	3.52E-03
204939_s_at	PLN	0.784	1.72	1.31E-02	4.49E-02
204502_at	SAMHD1	0.783	1.72	3.46E-08	1.85E-06
1560531_at	LCE1B	0.783	1.72	3.02E-06	6.90E-05
201506_at	TGFBI	0.783	1.72	1.84E-08	1.09E-06
1556427_s_at	LRRN4CL	0.782	1.72	3.09E-04	2.62E-03
230962_at	DCLK1	0.782	1.72	2.28E-04	2.08E-03
214087_s_at	MYBPC1	0.782	1.72	5.05E-04	3.81E-03
209842_at	SOX10	0.782	1.72	2.56E-05	3.80E-04
214596_at	CHRM3	0.782	1.72	2.52E-03	1.31E-02
203498_at	RCAN2	0.781	1.72	1.97E-06	4.82E-05
203104_at	CSF1R	0.781	1.72	8.37E-10	8.40E-08
204852_s_at	PTPN7	0.781	1.72	1.60E-07	6.30E-06
225701_at	AKNA	0.781	1.72	8.74E-09	5.73E-07
220559_at	EN1	0.781	1.72	5.68E-07	1.77E-05
206625_at	PRPH2	0.78	1.72	1.36E-04	1.39E-03
241739_at	OGFOD1	0.78	1.72	2.49E-04	2.22E-03
244276_at	KLB	0.78	1.72	1.11E-04	1.18E-03
1556096_s_at	UNC13C	0.778	1.72	1.63E-03	9.42E-03
225491_at	SLC1A2	0.778	1.72	3.41E-05	4.74E-04
1556839_s_at	LOC100289090	0.778	1.72	1.53E-03	8.95E-03
205599_at	TRAF1	0.777	1.71	7.64E-07	2.26E-05
232737_s_at	ENPP3	0.775	1.71	3.26E-04	2.73E-03
204424_s_at	LMO3	0.775	1.71	3.82E-03	1.79E-02
204919_at	PRH1-PRR4 /// PRR4	0.775	1.71	1.67E-03	9.57E-03
219716_at	APOL6	0.775	1.71	2.15E-07	8.04E-06
231773_at	ANGPTL1	0.775	1.71	5.05E-06	1.05E-04
219574_at	1-Mar	0.774	1.71	1.75E-07	6.78E-06
1557558_s_at	MATN1-AS1	0.774	1.71	3.14E-06	7.08E-05
234787_at	DKFZp564H213	0.773	1.71	5.85E-06	1.17E-04
236261_at	OSBPL6	0.771	1.71	1.27E-05	2.18E-04
204575_s_at	MMP19	0.771	1.71	8.50E-07	2.46E-05
211005_at	LAT	0.771	1.71	8.77E-07	2.51E-05
229254_at	MFSD4	0.771	1.71	2.93E-04	2.53E-03
209994_s_at	ABCB1 /// ABCB4	0.77	1.71	2.75E-06	6.36E-05
206601_s_at	HOXD3	0.77	1.7	9.67E-06	1.75E-04
243526_at	WDR86	0.769	1.7	1.36E-03	8.24E-03
205403_at	IL1R2	0.769	1.7	3.18E-08	1.73E-06

213418_at	HSPA6	0.768	1.7	5.65E-06	1.14E-04
236064_at	SLC25A35	0.767	1.7	2.74E-04	2.40E-03
218345_at	TMEM176A	0.767	1.7	9.63E-06	1.75E-04
236539_at	PTPN22	0.767	1.7	2.12E-06	5.14E-05
1563369_at	LINC00173	0.766	1.7	1.14E-04	1.21E-03
211742_s_at	EVI2B	0.766	1.7	6.96E-07	2.10E-05
209827_s_at	IL16	0.766	1.7	6.11E-07	1.89E-05
213095_x_at	AIF1	0.765	1.7	3.17E-08	1.73E-06
203547_at	CD4	0.765	1.7	3.21E-10	3.77E-08
204519_s_at	PLLP	0.765	1.7	2.63E-04	2.32E-03
235418_at	FAHD2A	0.764	1.7	9.15E-06	1.68E-04
230925_at	APBB1IP	0.764	1.7	7.66E-04	5.26E-03
229430_at	C8orf46	0.764	1.7	2.97E-05	4.28E-04
223655_at	CD163L1	0.763	1.7	1.25E-10	1.76E-08
207757_at	ZFP2	0.762	1.7	4.36E-06	9.28E-05
1564276_at	C5orf56	0.762	1.7	1.25E-06	3.36E-05
239183_at	ANGPTL1	0.761	1.69	4.42E-07	1.44E-05
1555890_at	OR2A20P /// OR2A9P	0.761	1.69	3.34E-04	2.78E-03
204670_x_at	HLA-DRB1 /// HLA-DRB4 /// LOC100507709 /// LOC100507714	0.76	1.69	6.92E-08	3.29E-06
230563_at	RASGEF1A	0.76	1.69	2.31E-04	2.10E-03
212588_at	PTPRC	0.759	1.69	1.18E-06	3.22E-05
232543_x_at	ARHGAP9	0.757	1.69	1.16E-06	3.18E-05
205691_at	SYNGR3	0.757	1.69	1.88E-05	2.97E-04
210895_s_at	CD86	0.757	1.69	2.86E-08	1.60E-06
205059_s_at	IDUA	0.756	1.69	3.69E-05	5.03E-04
204501_at	NOV	0.755	1.69	4.06E-07	1.34E-05
206707_x_at	FAM65B	0.755	1.69	8.09E-08	3.72E-06
230388_s_at	KANSL1-AS1	0.754	1.69	3.55E-03	1.69E-02
229902_at	FLT4	0.754	1.69	3.02E-05	4.33E-04
214243_s_at	SERHL /// SERHL2	0.754	1.69	1.23E-02	4.29E-02
205455_at	MST1R	0.753	1.69	5.10E-05	6.49E-04
213849_s_at	PPP2R2B	0.753	1.69	3.83E-07	1.28E-05
242988_at	DCST2	0.753	1.69	1.59E-05	2.62E-04
205299_s_at	BTN2A2	0.752	1.68	2.86E-05	4.15E-04
215051_x_at	AIF1	0.752	1.68	7.84E-09	5.28E-07
204352_at	TRAF5	0.752	1.68	5.37E-07	1.69E-05
205067_at	IL1B	0.752	1.68	2.88E-04	2.50E-03
206576_s_at	CEACAM1	0.751	1.68	1.14E-04	1.21E-03
214240_at	GAL	0.751	1.68	8.91E-03	3.35E-02
237291_at	PRORSD1P	0.751	1.68	1.33E-04	1.36E-03
207777_s_at	SP140	0.751	1.68	7.25E-07	2.17E-05
210385_s_at	ERAP1	0.75	1.68	1.35E-04	1.38E-03

205843_x_at	CRAT	0.75	1.68	2.74E-03	1.40E-02
219999_at	MAN2A2	0.749	1.68	4.89E-07	1.57E-05
204057_at	IRF8	0.748	1.68	8.90E-05	1.00E-03
242557_at	ZNRD1-AS1	0.747	1.68	8.33E-03	3.19E-02
230480_at	PIWIL4	0.747	1.68	1.10E-05	1.94E-04
222134_at	DDO	0.747	1.68	1.32E-05	2.25E-04
211372_s_at	IL1R2	0.746	1.68	7.23E-05	8.52E-04
228060_at	SLC35F1	0.746	1.68	1.05E-04	1.14E-03
238575_at	OSBPL6	0.746	1.68	1.28E-04	1.33E-03
1556666_a_at	LOC101059957 /// TTC6	0.745	1.68	3.71E-03	1.75E-02
204774_at	EVI2A	0.745	1.68	1.06E-05	1.88E-04
229800_at	DCLK1	0.745	1.68	3.18E-05	4.50E-04
210140_at	CST7	0.744	1.67	7.04E-04	4.93E-03
202307_s_at	TAP1	0.743	1.67	1.95E-09	1.65E-07
203708_at	PDE4B	0.743	1.67	1.02E-05	1.84E-04
214434_at	HSPA12A	0.743	1.67	5.44E-06	1.11E-04
204614_at	SERPINB2	0.743	1.67	4.33E-05	5.69E-04
204179_at	MB	0.743	1.67	9.48E-05	1.05E-03
210108_at	CACNA1D	0.742	1.67	5.63E-05	7.02E-04
232234_at	SLA2	0.742	1.67	1.62E-06	4.14E-05
228391_at	CYP4V2	0.741	1.67	7.31E-05	8.60E-04
211990_at	HLA-DPA1	0.74	1.67	1.50E-04	1.50E-03
59697_at	RAB15	0.74	1.67	1.09E-06	3.02E-05
223122_s_at	SFRP2	0.74	1.67	4.07E-03	1.87E-02
207623_at	ABCF2	0.74	1.67	5.33E-06	1.09E-04
213752_at	KAZN	0.739	1.67	2.51E-07	9.06E-06
228367_at	ALPK2	0.739	1.67	5.13E-05	6.52E-04
1554406_a_at	CLEC7A	0.738	1.67	2.10E-04	1.95E-03
229400_at	HOXD10	0.738	1.67	2.03E-04	1.91E-03
213369_at	CDHR1	0.737	1.67	1.02E-02	3.73E-02
206104_at	ISL1	0.737	1.67	4.64E-06	9.75E-05
210644_s_at	LAIR1	0.737	1.67	3.10E-03	1.53E-02
1560697_at	TRHDE-AS1	0.736	1.67	3.61E-04	2.95E-03
210380_s_at	CACNA1G	0.736	1.67	3.03E-06	6.91E-05
220615_s_at	FAR2	0.735	1.66	2.64E-03	1.36E-02
237974_at	ABHD12B /// MIR4454	0.735	1.66	1.55E-08	9.38E-07
221087_s_at	APOL3	0.735	1.66	7.50E-08	3.51E-06
214651_s_at	HOXA10-HOXA9 /// HOXA9 /// MIR196B	0.734	1.66	2.39E-05	3.61E-04
1552316_a_at	GIMAP1	0.734	1.66	2.19E-04	2.02E-03
208450_at	LGALS2	0.733	1.66	1.50E-03	8.84E-03
231175_at	BEND6	0.732	1.66	3.66E-05	5.01E-04
203932_at	HLA-DMB	0.732	1.66	4.19E-12	9.98E-10

229348_at	UBIAD1	0.732	1.66	4.88E-04	3.71E-03
211012_s_at	PML	0.731	1.66	5.00E-07	1.60E-05
218322_s_at	ACSL5	0.731	1.66	1.74E-06	4.37E-05
238581_at	GBP5	0.731	1.66	2.97E-04	2.55E-03
219836_at	ZBED2	0.731	1.66	3.24E-04	2.72E-03
209976_s_at	CYP2E1	0.731	1.66	3.02E-06	6.88E-05
207522_s_at	ATP2A3	0.731	1.66	1.42E-03	8.51E-03
230040_at	ADAMTS18	0.73	1.66	1.73E-03	9.82E-03
231348_s_at	LMO3	0.73	1.66	9.02E-03	3.39E-02
236514_at	ACOT8	0.73	1.66	6.49E-09	4.50E-07
211062_s_at	CPZ /// GPR78	0.73	1.66	1.29E-06	3.44E-05
226439_s_at	NBEA	0.729	1.66	3.02E-05	4.33E-04
238114_at	PCMTD1	0.729	1.66	8.91E-07	2.55E-05
208131_s_at	PTGIS	0.729	1.66	7.91E-05	9.18E-04
231384_at	GRIN2A	0.729	1.66	9.34E-04	6.18E-03
215967_s_at	LY9	0.729	1.66	8.44E-08	3.83E-06
228724_at	TTLL7	0.728	1.66	2.26E-04	2.07E-03
230888_at	WDR91	0.728	1.66	5.85E-06	1.17E-04
230143_at	RNF165	0.728	1.66	6.36E-07	1.95E-05
226627_at	8-Sep	0.728	1.66	1.03E-06	2.88E-05
1554240_a_at	ITGAL	0.727	1.66	1.60E-06	4.10E-05
231323_at	PSMB2	0.727	1.66	1.74E-04	1.69E-03
210262_at	CRISP2	0.726	1.65	2.73E-03	1.39E-02
1554704_at	ATP8B3	0.726	1.65	8.91E-07	2.55E-05
205660_at	OASL	0.726	1.65	1.48E-05	2.47E-04
228658_at	MIAT	0.726	1.65	2.61E-04	2.31E-03
219505_at	CECR1	0.725	1.65	3.76E-04	3.05E-03
207381_at	ALOX12B	0.725	1.65	1.81E-03	1.01E-02
240409_at	TPTE2P5	0.725	1.65	8.98E-04	5.99E-03
219799_s_at	DHRS9	0.725	1.65	8.94E-05	1.01E-03
230805_at	MIR142	0.724	1.65	4.36E-08	2.23E-06
206587_at	CCT6B	0.724	1.65	5.32E-05	6.70E-04
206204_at	GRB14	0.724	1.65	1.06E-02	3.83E-02
206337_at	CCR7	0.723	1.65	5.51E-06	1.12E-04
225033_at	ST3GAL1	0.723	1.65	2.15E-07	8.04E-06
202982_s_at	ACOT1 /// ACOT2	0.722	1.65	2.35E-03	1.24E-02
207547_s_at	FAM107A /// LOC101059937	0.722	1.65	1.72E-06	4.33E-05
210215_at	TFR2	0.721	1.65	6.49E-07	1.98E-05
1561226_at	XCR1	0.721	1.65	3.00E-05	4.31E-04
204529_s_at	TOX	0.72	1.65	1.06E-05	1.89E-04
238018_at	FAM150B	0.72	1.65	3.91E-04	3.15E-03
230036_at	SAMD9L	0.719	1.65	1.61E-05	2.64E-04

209685_s_at	PRKCB	0.719	1.65	1.67E-06	4.23E-05
232027_at	SYNE1	0.719	1.65	6.40E-05	7.73E-04
229857_s_at	KANSL1-AS1	0.719	1.65	9.15E-03	3.42E-02
223816_at	SLC46A2	0.719	1.65	5.71E-05	7.08E-04
208733_at	RAB2A	0.718	1.65	2.72E-03	1.39E-02
202156_s_at	CELF2	0.718	1.64	9.52E-07	2.69E-05
208018_s_at	HCK	0.718	1.64	7.56E-08	3.52E-06
229092_at	NR2F2	0.717	1.64	4.24E-05	5.60E-04
203766_s_at	LMOD1	0.717	1.64	4.25E-04	3.35E-03
1557283_a_at	ZNF519	0.717	1.64	1.44E-05	2.42E-04
205749_at	CYP1A1	0.715	1.64	1.03E-02	3.74E-02
37966_at	PARVB	0.715	1.64	1.09E-05	1.93E-04
206856_at	LILRB5	0.715	1.64	2.50E-05	3.74E-04
223946_at	MED23	0.715	1.64	3.53E-05	4.86E-04
230873_at	TTLL3	0.715	1.64	7.18E-04	5.01E-03
35974_at	LRMP	0.714	1.64	4.99E-06	1.04E-04
202075_s_at	PLTP	0.714	1.64	6.46E-10	6.75E-08
201037_at	PFKP	0.713	1.64	1.01E-09	9.83E-08
230417_at	GALNT16	0.713	1.64	3.25E-05	4.57E-04
218066_at	SLC12A7	0.713	1.64	1.11E-06	3.06E-05
229105_at	GPR39	0.712	1.64	9.52E-05	1.05E-03
1555728_a_at	MS4A4A	0.712	1.64	3.93E-08	2.05E-06
232083_at	KIF16B	0.711	1.64	5.82E-04	4.26E-03
214889_at	FAM149A	0.711	1.64	1.60E-04	1.58E-03
209374_s_at	IGHM	0.711	1.64	3.58E-03	1.70E-02
205221_at	HGD	0.711	1.64	7.28E-03	2.89E-02
210321_at	GZMH	0.711	1.64	7.47E-06	1.44E-04
1556136_at	MYLK4	0.71	1.64	4.86E-04	3.70E-03
207100_s_at	VAMP1	0.71	1.64	1.76E-05	2.84E-04
226811_at	FAM46C	0.709	1.63	2.99E-05	4.30E-04
231911_at	ERMN	0.709	1.63	3.21E-08	1.75E-06
203281_s_at	MIR5193 /// UBA7	0.709	1.63	5.53E-07	1.74E-05
214307_at	HGD	0.707	1.63	7.24E-04	5.04E-03
229802_at	WISP1	0.707	1.63	1.55E-04	1.54E-03
232300_at	ADIRF-AS1	0.707	1.63	1.85E-04	1.77E-03
210718_s_at	ARL17A /// ARL17B /// LOC100294341 /// LOC100996709	0.706	1.63	2.78E-03	1.41E-02
204674_at	LRMP	0.706	1.63	3.61E-06	7.97E-05
1559584_a_at	C16orf54	0.706	1.63	4.46E-04	3.48E-03
229649_at	NRXN3	0.706	1.63	3.12E-06	7.06E-05
213733_at	MYO1F	0.706	1.63	9.19E-08	4.08E-06
219213_at	JAM2	0.705	1.63	1.76E-05	2.84E-04

231195_at	KLRG2	0.705	1.63	4.56E-04	3.53E-03
223922_x_at	MS4A6A	0.704	1.63	1.63E-06	4.15E-05
230616_at	LAMB2P1	0.703	1.63	2.31E-06	5.52E-05
204735_at	PDE4A	0.703	1.63	5.83E-10	6.19E-08
220595_at	PDZRN4	0.703	1.63	2.15E-04	2.00E-03
230405_at	C5orf56	0.702	1.63	2.42E-06	5.73E-05
204220_at	GMFG	0.702	1.63	5.65E-07	1.77E-05
236583_at	GIMAP1	0.7	1.62	6.58E-05	7.91E-04
211909_x_at	PTGER3	0.7	1.62	1.18E-04	1.24E-03
232570_s_at	ADAM33	0.699	1.62	8.29E-05	9.52E-04
210297_s_at	MSMB	0.698	1.62	6.78E-03	2.74E-02
227048_at	LAMA1	0.698	1.62	1.30E-03	7.96E-03
228111_s_at	DNAH1	0.698	1.62	3.24E-05	4.56E-04
204014_at	DUSP4	0.698	1.62	2.89E-05	4.18E-04
222592_s_at	ACSL5	0.698	1.62	9.79E-05	1.07E-03
1562247_at	LOC286058	0.698	1.62	1.77E-05	2.84E-04
212187_x_at	PTGDS	0.697	1.62	6.68E-05	8.00E-04
1553212_at	KRT78	0.697	1.62	2.69E-03	1.38E-02
219690_at	IGFLR1	0.697	1.62	5.22E-06	1.07E-04
228132_at	ABLIM2	0.697	1.62	2.36E-07	8.65E-06
217557_s_at	CPM	0.697	1.62	8.74E-06	1.62E-04
207561_s_at	ABCB8 /// ASIC3	0.696	1.62	6.71E-04	4.74E-03
219723_x_at	AGPAT3	0.696	1.62	1.60E-05	2.63E-04
230974_at	DDX19B	0.695	1.62	2.64E-06	6.15E-05
205619_s_at	MEOX1	0.695	1.62	3.08E-07	1.08E-05
227563_at	FAM27E3	0.695	1.62	2.16E-04	2.00E-03
230453_s_at	ATP2A3	0.694	1.62	1.10E-03	6.97E-03
215082_at	ELOVL5	0.694	1.62	1.34E-02	4.58E-02
1553890_s_at	NTN5	0.694	1.62	8.63E-04	5.80E-03
1560011_at	JRK	0.694	1.62	1.15E-05	2.00E-04
239273_s_at	MMP28	0.694	1.62	1.10E-04	1.18E-03
226972_s_at	CCDC136	0.693	1.62	4.22E-05	5.57E-04
225353_s_at	C1QC	0.692	1.62	1.59E-07	6.27E-06
44790_s_at	KIAA0226L	0.691	1.61	4.47E-05	5.83E-04
206060_s_at	PTPN22	0.691	1.61	7.24E-06	1.40E-04
214963_at	NUP160	0.691	1.61	7.44E-05	8.73E-04
206539_s_at	CYP4F12	0.691	1.61	2.12E-04	1.97E-03
205225_at	ESR1	0.69	1.61	2.70E-05	3.97E-04
213942_at	MEGF6	0.69	1.61	2.32E-06	5.52E-05
219959_at	MOCOS	0.689	1.61	9.81E-03	3.61E-02
210432_s_at	SCN3A	0.689	1.61	3.28E-03	1.60E-02
228150_at	SEC16B	0.689	1.61	1.26E-07	5.22E-06

228607_at	OAS2	0.688	1.61	4.54E-06	9.59E-05
219211_at	USP18	0.688	1.61	8.11E-06	1.53E-04
202510_s_at	TNFAIP2	0.688	1.61	8.25E-08	3.77E-06
201422_at	IFI30 /// PIK3R2	0.688	1.61	4.25E-08	2.19E-06
204829_s_at	FOLR2	0.687	1.61	7.94E-06	1.50E-04
236055_at	DQX1	0.687	1.61	2.05E-04	1.92E-03
205066_s_at	ENPP1	0.687	1.61	6.53E-05	7.85E-04
218400_at	OAS3	0.687	1.61	2.96E-05	4.26E-04
235056_at	ETV6	0.686	1.61	3.27E-06	7.32E-05
223950_s_at	FLYWCH1	0.686	1.61	1.07E-03	6.83E-03
244070_at	SYNE1	0.686	1.61	1.86E-05	2.96E-04
235122_at	HIVEP3	0.685	1.61	2.18E-04	2.02E-03
214746_s_at	ZNF467	0.685	1.61	1.68E-03	9.61E-03
228182_at	ADCY5	0.685	1.61	2.73E-04	2.39E-03
230488_s_at	DBH-AS1	0.685	1.61	1.44E-05	2.42E-04
238422_at	LBX2-AS1	0.684	1.61	2.44E-04	2.19E-03
205896_at	SLC22A4	0.684	1.61	1.25E-02	4.34E-02
210982_s_at	HLA-DRA	0.684	1.61	4.52E-05	5.88E-04
223800_s_at	LIMS3 /// LIMS3L	0.683	1.61	1.20E-05	2.08E-04
1569886_a_at	GLB1L3	0.683	1.61	2.53E-05	3.78E-04
219562_at	RAB26	0.682	1.6	2.21E-04	2.04E-03
244251_at	LCP2	0.682	1.6	1.06E-06	2.95E-05
1562573_at	CYP17A1	0.682	1.6	4.30E-04	3.38E-03
219471_at	KIAA0226L	0.682	1.6	4.01E-05	5.37E-04
234994_at	TMEM200A	0.682	1.6	2.04E-04	1.91E-03
205073_at	CYP2J2	0.682	1.6	2.69E-04	2.37E-03
213160_at	DOCK2	0.681	1.6	9.57E-07	2.70E-05
229222_at	ACSS3	0.681	1.6	3.59E-05	4.92E-04
208189_s_at	MYO7A	0.681	1.6	9.60E-06	1.74E-04
206030_at	ASPA	0.68	1.6	1.18E-04	1.25E-03
209695_at	PTP4A3	0.68	1.6	1.15E-05	2.00E-04
1553213_a_at	KRT78	0.68	1.6	1.33E-03	8.09E-03
1568638_a_at	IDO2	0.679	1.6	2.72E-06	6.31E-05
210253_at	HTATIP2	0.679	1.6	3.18E-05	4.50E-04
209948_at	KCNMB1	0.679	1.6	2.27E-04	2.07E-03
203673_at	TG	0.678	1.6	1.18E-03	7.37E-03
214210_at	SLC25A17	0.678	1.6	1.73E-07	6.73E-06
211740_at	ICA1	0.678	1.6	6.79E-03	2.75E-02
204563_at	SELL	0.678	1.6	2.43E-03	1.27E-02
214719_at	SLC46A3	0.677	1.6	1.58E-07	6.27E-06
229947_at	PI15	0.677	1.6	1.58E-04	1.57E-03
1559050_at	HCG27 /// LOC100996357	0.677	1.6	1.28E-05	2.20E-04

244444_at	PKD1L2	0.677	1.6	7.06E-05	8.36E-04
239345_at	SLC19A3	0.676	1.6	3.07E-04	2.61E-03
204914_s_at	SOX11	0.676	1.6	6.54E-03	2.67E-02
205306_x_at	KMO	0.676	1.6	5.69E-05	7.06E-04
232024_at	GIMAP2	0.676	1.6	1.47E-07	5.92E-06
237411_at	ADAMTS6	0.676	1.6	2.26E-04	2.07E-03
205593_s_at	PDE9A	0.676	1.6	3.44E-04	2.85E-03
211685_s_at	NCALD	0.676	1.6	2.83E-03	1.43E-02
219429_at	FA2H	0.675	1.6	5.81E-05	7.16E-04
205898_at	CX3CR1	0.675	1.6	5.12E-06	1.06E-04
230425_at	EPHB1	0.675	1.6	4.81E-05	6.17E-04
227867_at	TRABD2A	0.675	1.6	5.30E-05	6.70E-04
239582_at	PML	0.675	1.6	6.35E-08	3.07E-06
229015_at	LOC286367	0.674	1.6	2.70E-03	1.38E-02
1570020_at	AACSP1	0.674	1.6	8.07E-04	5.48E-03
209670_at	TRAC	0.673	1.59	4.82E-07	1.55E-05
209883_at	COLGALT2	0.673	1.59	2.70E-04	2.37E-03
206502_s_at	INSM1	0.673	1.59	1.03E-04	1.12E-03
239178_at	FGF9	0.673	1.59	9.57E-05	1.05E-03
1556063_s_at	RPP30	0.672	1.59	3.53E-06	7.81E-05
224225_s_at	ETV7	0.672	1.59	8.89E-05	1.00E-03
228780_at	POU3F3	0.671	1.59	6.48E-05	7.80E-04
37145_at	GNLY	0.671	1.59	2.47E-04	2.21E-03
235780_at	PRKACB	0.671	1.59	4.62E-06	9.72E-05
230631_s_at	IL10RB-AS1	0.671	1.59	2.00E-04	1.89E-03
208606_s_at	WNT4	0.67	1.59	2.53E-04	2.25E-03
213309_at	PLCL2	0.67	1.59	5.77E-06	1.16E-04
1556471_at	SCML4	0.67	1.59	1.82E-05	2.90E-04
232794_at	LOC153682	0.669	1.59	6.96E-06	1.36E-04
203402_at	KCNAB2	0.668	1.59	5.67E-07	1.77E-05
237753_at	IL21R	0.668	1.59	3.44E-04	2.85E-03
207900_at	CCL17	0.667	1.59	1.35E-04	1.39E-03
206552_s_at	TAC1	0.667	1.59	2.24E-03	1.20E-02
210794_s_at	MEG3	0.667	1.59	2.71E-05	3.97E-04
230169_at	THAP6	0.667	1.59	7.26E-07	2.17E-05
40420_at	STK10	0.667	1.59	1.36E-09	1.24E-07
1554057_at	ASH1L-AS1	0.667	1.59	3.09E-04	2.63E-03
203559_s_at	ABP1	0.667	1.59	3.66E-06	8.07E-05
202952_s_at	ADAM12	0.666	1.59	1.12E-04	1.20E-03
211748_x_at	PTGDS	0.666	1.59	1.56E-05	2.57E-04
206655_s_at	GP1BB /// SEPT5 /// SEPT5-GP1BB	0.666	1.59	1.89E-03	1.05E-02
207376_at	VENTX	0.666	1.59	5.26E-06	1.08E-04

219232_s_at	EGLN3	0.666	1.59	2.18E-06	5.25E-05
226777_at	ADAM12 /// ADAM12-OT1	0.665	1.59	2.09E-04	1.95E-03
242450_at	RGMB	0.665	1.59	2.02E-04	1.90E-03
208195_at	TTN	0.665	1.59	7.64E-06	1.46E-04
204940_at	PLN	0.664	1.58	9.83E-03	3.62E-02
226906_s_at	ARHGAP9	0.664	1.58	7.69E-07	2.27E-05
204265_s_at	GPSM3	0.663	1.58	3.41E-07	1.17E-05
228647_at	LOC100049716	0.663	1.58	8.58E-07	2.48E-05
235050_at	SLC2A12	0.662	1.58	6.29E-05	7.64E-04
213693_s_at	MUC1	0.661	1.58	7.24E-03	2.88E-02
204717_s_at	SLC29A2	0.661	1.58	5.55E-06	1.12E-04
224932_at	CHCHD10	0.661	1.58	1.49E-06	3.86E-05
204198_s_at	RUNX3	0.661	1.58	4.86E-07	1.56E-05
1559839_at	TBX18	0.66	1.58	8.82E-04	5.90E-03
229019_at	ZNF385B	0.66	1.58	2.70E-03	1.38E-02
206600_s_at	SLC16A5	0.66	1.58	3.50E-05	4.83E-04
227201_at	LOC643837	0.66	1.58	1.17E-04	1.24E-03
232792_at	TRIM69	0.66	1.58	2.00E-05	3.12E-04
237493_at	IL22RA2	0.659	1.58	9.89E-08	4.33E-06
205522_at	HOXD4	0.659	1.58	5.56E-05	6.94E-04
1559144_x_at	LINC00910	0.659	1.58	1.56E-05	2.58E-04
226743_at	SLFN11	0.658	1.58	4.62E-08	2.35E-06
214828_s_at	RRP7A /// RRP7B	0.658	1.58	9.35E-03	3.48E-02
230756_at	ZNF683	0.658	1.58	1.54E-09	1.36E-07
230440_at	ZNF469	0.658	1.58	1.25E-06	3.36E-05
204438_at	MRC1	0.658	1.58	1.76E-06	4.40E-05
204499_at	AGTPBP1	0.657	1.58	9.87E-06	1.78E-04
243606_at	NXPE3	0.657	1.58	1.69E-05	2.74E-04
206170_at	ADRB2	0.657	1.58	8.27E-10	8.35E-08
242204_at	WFDC5	0.656	1.58	2.92E-04	2.53E-03
204301_at	KBTBD11	0.655	1.58	1.93E-06	4.75E-05
1554003_at	ARHGEF28	0.655	1.58	3.67E-05	5.01E-04
206710_s_at	EPB41L3	0.655	1.57	1.46E-07	5.90E-06
207238_s_at	PTPRC	0.655	1.57	1.47E-04	1.49E-03
242388_x_at	TAGAP	0.655	1.57	1.42E-04	1.44E-03
230145_at	DUS3L	0.655	1.57	2.72E-04	2.39E-03
227794_at	GLYATL1 /// LOC100287413	0.654	1.57	3.43E-03	1.65E-02
234312_s_at	ACSS2	0.654	1.57	1.03E-04	1.11E-03
236673_at	TIFAB	0.654	1.57	1.11E-06	3.06E-05
206219_s_at	VAV1	0.653	1.57	5.37E-07	1.69E-05
209083_at	CORO1A	0.653	1.57	2.12E-06	5.13E-05
239694_at	TRIM7	0.653	1.57	3.67E-05	5.01E-04

225285_at	BCAT1	0.653	1.57	3.02E-04	2.58E-03
212750_at	PPP1R16B	0.652	1.57	3.06E-07	1.07E-05
207328_at	ALOX15	0.652	1.57	6.42E-04	4.59E-03
208056_s_at	CBFA2T3	0.651	1.57	2.64E-04	2.33E-03
235891_at	DNM3OS /// MIR214 /// MIR3120	0.651	1.57	3.53E-05	4.86E-04
203561_at	FCGR2A	0.651	1.57	6.11E-06	1.21E-04
219610_at	ARHGEF28	0.651	1.57	2.35E-07	8.63E-06
223741_s_at	TTYH2	0.65	1.57	4.03E-04	3.22E-03
231600_at	CLEC12B	0.65	1.57	8.90E-03	3.35E-02
36030_at	IFFO1	0.649	1.57	8.95E-06	1.65E-04
222124_at	HIF3A	0.648	1.57	7.21E-03	2.87E-02
204629_at	PARVB	0.648	1.57	2.92E-05	4.22E-04
226841_at	MPEG1	0.648	1.57	1.37E-06	3.61E-05
206726_at	HPGDS	0.647	1.57	7.13E-03	2.85E-02
226611_s_at	CENPV	0.647	1.57	4.45E-05	5.80E-04
233510_s_at	PARVG	0.647	1.57	2.27E-05	3.46E-04
206693_at	IL7	0.646	1.57	8.50E-06	1.58E-04
242414_at	QPRT	0.646	1.57	5.04E-05	6.43E-04
213489_at	MAPRE2	0.646	1.57	2.81E-06	6.46E-05
336_at	TBXA2R	0.646	1.56	1.83E-03	1.03E-02
202803_s_at	ITGB2	0.646	1.56	4.02E-05	5.37E-04
223534_s_at	RPS6KL1	0.646	1.56	4.55E-05	5.91E-04
237316_at	ANKDD1A	0.646	1.56	4.43E-03	2.00E-02
235597_s_at	RGPD1 /// RGPD2	0.646	1.56	2.30E-03	1.22E-02
205880_at	PRKD1	0.646	1.56	8.44E-07	2.45E-05
235708_at	KLB	0.646	1.56	1.50E-02	4.97E-02
241765_at	CPM	0.645	1.56	1.25E-05	2.16E-04
213213_at	DIDO1	0.645	1.56	4.05E-05	5.40E-04
217525_at	OLFML1	0.645	1.56	2.48E-05	3.72E-04
204118_at	CD48	0.644	1.56	9.86E-04	6.44E-03
230360_at	GLDN	0.644	1.56	6.95E-07	2.10E-05
222218_s_at	PILRA	0.644	1.56	1.55E-05	2.57E-04
235634_at	PURG	0.644	1.56	1.92E-03	1.06E-02
244680_at	GLRB	0.644	1.56	2.74E-04	2.40E-03
211795_s_at	FYB	0.643	1.56	4.75E-05	6.11E-04
239675_at	LINC00900	0.643	1.56	4.58E-04	3.54E-03
224027_at	CCL28	0.643	1.56	5.04E-06	1.05E-04
1554248_at	ZNF638	0.643	1.56	5.93E-04	4.31E-03
227412_at	PPP1R3E	0.643	1.56	6.38E-06	1.26E-04
235292_at	FLJ32255	0.643	1.56	1.52E-05	2.52E-04
239130_at	MIR101-1 /// MIR3671	0.642	1.56	2.09E-04	1.95E-03
220073_s_at	PLEKHG6	0.642	1.56	9.75E-06	1.76E-04
1560698_a_a	TRHDE-AS1	0.642	1.56	1.28E-03	7.87E-03

t					
214053_at	ERBB4	0.642	1.56	1.40E-02	4.72E-02
244779_at	ZDHHC2	0.642	1.56	1.14E-05	2.00E-04
204336_s_at	RGS19	0.642	1.56	3.47E-05	4.80E-04
242013_at	BCL2L15	0.641	1.56	5.81E-05	7.17E-04
223533_at	LRRC8C	0.641	1.56	7.71E-04	5.28E-03
225973_at	TAP2	0.641	1.56	9.34E-06	1.71E-04
219195_at	PPARGC1A	0.641	1.56	1.22E-03	7.57E-03
1552497_a_at	SLAMF6	0.641	1.56	3.68E-05	5.01E-04
207351_s_at	SH2D2A	0.641	1.56	2.18E-06	5.25E-05
226955_at	AFAP1L1	0.64	1.56	3.82E-04	3.09E-03
205337_at	DCT	0.64	1.56	4.53E-04	3.52E-03
1552634_a_at	ZNF101	0.64	1.56	3.85E-04	3.11E-03
242940_x_at	DLX6	0.639	1.56	3.00E-04	2.57E-03
240509_s_at	GREM2	0.639	1.56	1.94E-03	1.07E-02
222101_s_at	DCHS1	0.639	1.56	5.64E-04	4.15E-03
205671_s_at	HLA-DOB	0.639	1.56	7.30E-03	2.90E-02
225909_at	LOC728743 /// ZNF775	0.639	1.56	6.79E-05	8.11E-04
240429_at	ZNF546	0.639	1.56	3.59E-05	4.92E-04
229052_at	ANKRD23 /// ANKRD39	0.639	1.56	9.20E-05	1.03E-03
220384_at	NME8	0.638	1.56	1.44E-03	8.58E-03
1555854_at	AKR1C1 /// AKR1C2 /// LOC101060798	0.638	1.56	9.23E-03	3.44E-02
209546_s_at	APOL1	0.638	1.56	5.18E-06	1.07E-04
210576_at	CYP4F8	0.638	1.56	2.90E-03	1.46E-02
205433_at	BCHE	0.637	1.56	1.38E-04	1.41E-03
215446_s_at	LOX	0.637	1.56	2.99E-05	4.30E-04
221916_at	NEFL	0.637	1.55	3.00E-03	1.49E-02
39402_at	IL1B	0.636	1.55	5.43E-04	4.03E-03
228448_at	MAP6	0.636	1.55	9.37E-06	1.71E-04
211743_s_at	PRG2	0.636	1.55	3.33E-03	1.61E-02
210451_at	PKLR	0.635	1.55	4.52E-03	2.03E-02
224456_s_at	FOXD2-AS1	0.635	1.55	3.43E-04	2.84E-03
204785_x_at	IFNAR2	0.635	1.55	1.45E-04	1.47E-03
229797_at	MCOLN3	0.634	1.55	1.34E-03	8.12E-03
215303_at	DCLK1	0.634	1.55	6.49E-04	4.63E-03
231980_at	DOK6	0.634	1.55	1.09E-03	6.92E-03
1553454_at	RPTN	0.634	1.55	6.20E-03	2.57E-02
224463_s_at	C11orf70	0.634	1.55	9.45E-05	1.05E-03
228160_at	LINC00667	0.633	1.55	2.09E-04	1.95E-03
236436_at	SLC25A45	0.633	1.55	2.00E-05	3.12E-04
238025_at	MLKL	0.633	1.55	2.37E-05	3.58E-04
222905_s_at	TMEM143	0.633	1.55	5.48E-06	1.11E-04

206421_s_at	SERPINB7	0.633	1.55	7.51E-03	2.96E-02
220435_at	SLC30A10	0.633	1.55	5.53E-03	2.37E-02
239846_at	MTHFD1	0.632	1.55	9.08E-05	1.02E-03
209765_at	ADAM19	0.631	1.55	1.74E-05	2.81E-04
223699_at	CNDP1	0.631	1.55	1.33E-02	4.55E-02
203508_at	TNFRSF1B	0.631	1.55	1.07E-07	4.58E-06
212276_at	LPIN1	0.631	1.55	4.27E-05	5.63E-04
211343_s_at	COL13A1	0.63	1.55	1.01E-06	2.85E-05
204908_s_at	BCL3	0.63	1.55	6.99E-05	8.31E-04
214186_s_at	HCG26	0.63	1.55	1.52E-04	1.52E-03
213566_at	RNASE6	0.63	1.55	4.34E-06	9.24E-05
202888_s_at	ANPEP	0.63	1.55	1.53E-08	9.29E-07
209375_at	XPC	0.629	1.55	4.04E-05	5.39E-04
228847_at	EXOC3	0.629	1.55	1.98E-04	1.87E-03
201137_s_at	HLA-DPB1	0.629	1.55	1.51E-05	2.51E-04
208335_s_at	DARC	0.628	1.55	3.07E-06	6.96E-05
212611_at	DTX4	0.628	1.55	5.64E-05	7.02E-04
219734_at	SIDT1	0.628	1.55	6.58E-06	1.29E-04
202465_at	PCOLCE	0.628	1.55	5.83E-07	1.81E-05
212451_at	SECISBP2L	0.628	1.54	2.79E-05	4.07E-04
203523_at	LSP1	0.627	1.54	1.67E-05	2.72E-04
236480_at	MIR210HG	0.627	1.54	3.87E-04	3.12E-03
229734_at	MIR4697HG	0.627	1.54	2.73E-07	9.75E-06
206345_s_at	PON1	0.627	1.54	1.48E-03	8.74E-03
210664_s_at	TFPI	0.626	1.54	4.02E-05	5.37E-04
206974_at	CXCR6	0.626	1.54	5.72E-06	1.15E-04
227482_at	ADCK1	0.626	1.54	1.78E-04	1.71E-03
210194_at	PLA2R1	0.626	1.54	2.67E-05	3.94E-04
242207_at	CENPP /// LOC100128361	0.626	1.54	1.04E-04	1.12E-03
1552410_at	CLEC4F	0.626	1.54	4.85E-04	3.70E-03
239683_at	CLYBL	0.626	1.54	2.17E-05	3.34E-04
213326_at	VAMP1	0.625	1.54	2.82E-05	4.11E-04
234946_at	ENTPD6	0.625	1.54	1.35E-03	8.19E-03
204820_s_at	BTN3A2 /// BTN3A3	0.625	1.54	1.76E-07	6.81E-06
230626_at	TSPAN12	0.624	1.54	8.43E-03	3.22E-02
205399_at	DCLK1	0.624	1.54	4.38E-06	9.30E-05
208894_at	HLA-DRA	0.624	1.54	5.23E-04	3.91E-03
239260_at	CORIN	0.624	1.54	3.64E-04	2.97E-03
236154_at	QKI	0.624	1.54	9.83E-05	1.08E-03
238121_at	GK5	0.624	1.54	1.01E-05	1.82E-04
214032_at	ZAP70	0.624	1.54	1.20E-08	7.57E-07
213820_s_at	STARD5	0.623	1.54	2.62E-06	6.11E-05
228888_at	STAC2	0.623	1.54	4.39E-03	1.98E-02

226047_at	MRVI1	0.623	1.54	7.35E-04	5.09E-03
1555865_at	TOLLIP-AS1	0.623	1.54	9.22E-04	6.11E-03
1555579_s_a t	PTPRM	0.622	1.54	2.42E-06	5.73E-05
228167_at	KLHL6	0.622	1.54	9.50E-05	1.05E-03
233880_at	RNF213	0.621	1.54	1.48E-05	2.47E-04
213559_s_at	ZNF467	0.621	1.54	1.56E-03	9.09E-03
238423_at	SYTL3	0.621	1.54	1.55E-03	9.07E-03
219056_at	RNASEH2B	0.621	1.54	6.42E-05	7.74E-04
220351_at	CCRL1	0.621	1.54	4.25E-03	1.94E-02
210325_at	CD1A	0.62	1.54	1.33E-04	1.37E-03
244468_at	PDZK1P1	0.62	1.54	7.80E-05	9.08E-04
1554824_at	ZNF585A	0.62	1.54	2.71E-04	2.38E-03
202499_s_at	SLC2A3	0.62	1.54	6.09E-03	2.53E-02
203332_s_at	INPP5D	0.619	1.54	5.04E-07	1.60E-05
227285_at	C1orf51	0.619	1.54	1.04E-04	1.13E-03
235414_at	ZNF383	0.619	1.54	3.02E-05	4.33E-04
223423_at	GPR160	0.619	1.54	1.02E-04	1.11E-03
219525_at	SLC47A1	0.619	1.54	8.62E-04	5.80E-03
219131_at	UBIAD1	0.619	1.54	3.06E-03	1.52E-02
235593_at	ZEB2	0.619	1.54	4.49E-04	3.50E-03
202032_s_at	MAN2A2	0.618	1.54	9.41E-08	4.16E-06
227877_at	ANXA2R	0.618	1.53	1.18E-06	3.22E-05
214735_at	IPCEF1	0.618	1.53	1.07E-05	1.90E-04
213245_at	ADCY1	0.618	1.53	7.82E-04	5.34E-03
229631_at	DNHD1	0.618	1.53	1.31E-04	1.35E-03
233083_at	MTHFD2L	0.618	1.53	1.01E-05	1.81E-04
229450_at	IFIT3	0.618	1.53	1.13E-04	1.20E-03
238454_at	ZNF540	0.617	1.53	6.51E-06	1.28E-04
204685_s_at	ATP2B2	0.617	1.53	3.89E-04	3.13E-03
219033_at	PARP8	0.617	1.53	9.41E-08	4.16E-06
211883_x_at	CEACAM1	0.617	1.53	4.34E-06	9.24E-05
208268_at	ADAM28	0.616	1.53	1.35E-05	2.29E-04
235639_at	CDH19	0.616	1.53	3.43E-04	2.84E-03
209245_s_at	KIF1C	0.616	1.53	2.67E-05	3.93E-04
239349_at	C1QTNF7	0.615	1.53	4.30E-03	1.95E-02
227733_at	TMEM63C	0.615	1.53	3.64E-04	2.97E-03
38149_at	ARHGAP25	0.615	1.53	5.80E-08	2.87E-06
89977_at	ACSM5	0.615	1.53	1.70E-03	9.70E-03
224583_at	COTL1	0.615	1.53	2.50E-04	2.23E-03
217630_at	ANGEL2	0.614	1.53	8.28E-04	5.60E-03
204821_at	BTN3A3	0.614	1.53	2.69E-06	6.24E-05
231261_at	CATSPERG	0.614	1.53	4.51E-05	5.87E-04

235515_at	SYNE4	0.614	1.53	1.01E-03	6.58E-03
230673_at	PKHD1L1	0.613	1.53	4.73E-05	6.10E-04
206991_s_at	CCR5	0.613	1.53	4.16E-06	8.93E-05
235518_at	SLC8A1	0.613	1.53	6.34E-05	7.67E-04
34210_at	CD52	0.613	1.53	2.73E-04	2.39E-03
229687_s_at	PRDM11	0.613	1.53	2.53E-04	2.26E-03
240806_at	RPL15	0.613	1.53	6.80E-07	2.06E-05
236360_at	FLJ42875	0.613	1.53	4.47E-04	3.48E-03
206120_at	CD33	0.613	1.53	2.19E-05	3.35E-04
229175_at	SMYD4	0.612	1.53	4.32E-07	1.42E-05
236465_at	RNF175	0.612	1.53	5.27E-05	6.66E-04
235199_at	RNF125	0.612	1.53	1.64E-10	2.17E-08
219773_at	NOX4	0.611	1.53	3.91E-04	3.15E-03
229002_at	FAM69B	0.61	1.53	1.47E-02	4.89E-02
227015_at	ASPHD2	0.61	1.53	2.98E-04	2.56E-03
227344_at	IKZF1	0.61	1.53	2.89E-05	4.18E-04
223694_at	TRIM7	0.61	1.53	2.51E-04	2.24E-03
226745_at	CYP4V2	0.61	1.53	1.57E-04	1.55E-03
227196_at	RHPN2	0.609	1.53	1.24E-02	4.31E-02
202192_s_at	GAS7	0.609	1.53	5.06E-11	8.27E-09
1558177_at	TMEM229B	0.609	1.53	4.37E-06	9.29E-05
214147_at	MROH7	0.609	1.53	2.18E-03	1.18E-02
243403_x_at	CPM	0.609	1.53	2.87E-06	6.59E-05
226610_at	CENPV	0.609	1.52	9.30E-05	1.03E-03
227610_at	TSPAN11	0.609	1.52	6.72E-04	4.75E-03
213822_s_at	UBE3B	0.608	1.52	3.52E-03	1.68E-02
222123_s_at	HIF3A	0.608	1.52	1.71E-04	1.66E-03
207067_s_at	HDC	0.608	1.52	5.61E-03	2.39E-02
1556346_at	COTL1	0.608	1.52	8.71E-06	1.61E-04
230836_at	ST8SIA4	0.608	1.52	1.33E-06	3.54E-05
226553_at	TMPRSS2	0.607	1.52	8.71E-03	3.30E-02
206157_at	PTX3	0.607	1.52	2.21E-04	2.03E-03
205447_s_at	MAP3K12	0.607	1.52	7.83E-05	9.11E-04
205904_at	MICA	0.607	1.52	7.73E-06	1.47E-04
209816_at	PTCH1	0.606	1.52	4.00E-03	1.85E-02
207443_at	NR2E1	0.606	1.52	3.75E-04	3.05E-03
238545_at	BRD7	0.606	1.52	2.32E-03	1.23E-02
239929_at	PM20D1	0.606	1.52	2.23E-04	2.05E-03
243602_at	MGC40069	0.605	1.52	3.82E-05	5.18E-04
241456_at	FAM78B	0.605	1.52	2.92E-04	2.52E-03
227178_at	CELF2	0.604	1.52	1.68E-05	2.74E-04
205518_s_at	CMAHP	0.604	1.52	2.02E-04	1.90E-03
233111_at	PTCSC1	0.604	1.52	5.53E-06	1.12E-04

227702_at	CYP4X1	0.604	1.52	1.48E-05	2.47E-04
211776_s_at	EPB41L3	0.604	1.52	2.02E-06	4.93E-05
216237_s_at	MCM5	0.604	1.52	2.19E-04	2.02E-03
240512_x_at	KCTD4	0.603	1.52	1.42E-03	8.51E-03
220646_s_at	KLRF1	0.603	1.52	6.49E-04	4.63E-03
1558934_a_at	GTF2H5	0.603	1.52	5.19E-04	3.89E-03
221218_s_at	TPK1	0.603	1.52	1.44E-07	5.83E-06
227803_at	ENPP5	0.603	1.52	3.60E-03	1.71E-02
202638_s_at	ICAM1	0.603	1.52	1.49E-04	1.49E-03
205484_at	SIT1	0.602	1.52	3.24E-05	4.55E-04
242957_at	VWCE	0.601	1.52	5.32E-04	3.96E-03
206695_x_at	ZNF43	0.6	1.52	1.72E-06	4.32E-05
228177_at	CREBBP	0.6	1.52	1.54E-04	1.53E-03
202637_s_at	ICAM1	0.6	1.52	7.46E-07	2.22E-05
204639_at	ADA	0.6	1.52	3.57E-04	2.93E-03
1569225_a_at	SCML4	0.599	1.52	1.96E-04	1.86E-03
243027_at	IGSF5	0.599	1.51	1.49E-03	8.80E-03
240430_at	KCMF1	0.598	1.51	2.88E-05	4.17E-04
202357_s_at	CFB	0.598	1.51	7.71E-05	9.00E-04
228802_at	RBPMS2	0.598	1.51	2.36E-03	1.24E-02
223211_at	HACL1	0.598	1.51	8.39E-05	9.61E-04
232001_at	PRKCQ-AS1	0.598	1.51	5.47E-03	2.34E-02
234841_x_at	OBP2A	0.597	1.51	3.65E-05	4.99E-04
230457_at	TMEM242	0.597	1.51	1.64E-04	1.61E-03
207317_s_at	CASQ2	0.597	1.51	1.24E-02	4.30E-02
213750_at	RSL1D1	0.596	1.51	2.42E-06	5.73E-05
230751_at	WNT4	0.596	1.51	1.27E-03	7.82E-03
238977_at	MCM6	0.596	1.51	1.44E-05	2.42E-04
235173_at	MBNL1-AS1	0.595	1.51	5.06E-03	2.21E-02
205081_at	CRIP1	0.595	1.51	4.45E-05	5.80E-04
209933_s_at	CD300A	0.595	1.51	2.55E-05	3.79E-04
218126_at	RMDN3	0.595	1.51	4.75E-06	9.92E-05
229039_at	SYN2	0.595	1.51	9.15E-04	6.08E-03
227138_at	CRTAP	0.594	1.51	2.19E-07	8.16E-06
60471_at	RIN3	0.594	1.51	2.57E-08	1.45E-06
221805_at	NEFL	0.594	1.51	4.46E-04	3.48E-03
227606_s_at	STAMBPL1	0.594	1.51	1.02E-06	2.86E-05
239425_at	DCUN1D5	0.594	1.51	2.48E-05	3.72E-04
232662_x_at	FAM213A	0.594	1.51	3.75E-08	1.97E-06
231562_at	APOC2	0.594	1.51	4.95E-06	1.03E-04
227753_at	TMEM139	0.593	1.51	1.01E-03	6.59E-03
203320_at	SH2B3	0.593	1.51	2.92E-06	6.69E-05

243099_at	NFAM1	0.593	1.51	2.45E-08	1.39E-06
216250_s_at	LPXN	0.592	1.51	1.94E-08	1.13E-06
226805_at	FITM2	0.592	1.51	1.46E-05	2.45E-04
224162_s_at	FBXO31	0.592	1.51	4.12E-06	8.88E-05
212827_at	IGHM	0.592	1.51	5.06E-03	2.21E-02
236309_x_at	ZMIZ2	0.592	1.51	4.32E-06	9.21E-05
213837_at	L3MBTL1	0.592	1.51	4.64E-06	9.75E-05
244629_s_at	PDPK1	0.592	1.51	1.98E-03	1.09E-02
217521_at	HAL	0.592	1.51	7.39E-03	2.93E-02
226992_at	NOSTRIN	0.592	1.51	4.33E-03	1.96E-02
1561589_a_at	NBEAL1	0.592	1.51	3.12E-04	2.64E-03
230802_at	ARHGAP24	0.591	1.51	1.67E-03	9.58E-03
230780_at	LINC00886	0.591	1.51	7.95E-04	5.41E-03
1563088_a_at	LOC284837	0.591	1.51	2.88E-04	2.50E-03
205114_s_at	CCL3 /// CCL3L1 /// CCL3L3 /// LOC101060267	0.59	1.5	2.93E-04	2.53E-03
212091_s_at	COL6A1	0.589	1.5	4.84E-06	1.01E-04
237748_at	SCGB2B2	0.589	1.5	1.15E-02	4.07E-02
206804_at	CD3G	0.589	1.5	1.11E-04	1.19E-03
231577_s_at	GBP1	0.589	1.5	1.14E-03	7.17E-03
212580_at	CAST	0.589	1.5	3.09E-04	2.62E-03
1560762_at	LOC285972	0.588	1.5	1.24E-03	7.66E-03
203260_at	HDDC2	0.588	1.5	1.45E-03	8.63E-03
223522_at	MIR600 /// MIR600HG	0.588	1.5	1.32E-03	8.03E-03
232155_at	RNF213	0.588	1.5	6.41E-05	7.74E-04
224856_at	FKBP5	0.587	1.5	3.95E-04	3.17E-03
1558770_a_at	PIK3R6	0.587	1.5	2.93E-03	1.47E-02
227396_at	PTPRJ	0.587	1.5	1.98E-06	4.84E-05
239074_at	GRAPL	0.586	1.5	7.47E-04	5.16E-03
220326_s_at	ARHGEF40	0.586	1.5	2.98E-05	4.30E-04
226702_at	CMPK2	0.586	1.5	4.00E-04	3.21E-03
205841_at	JAK2	0.586	1.5	2.55E-06	5.97E-05
228677_s_at	RASAL3	0.585	1.5	4.16E-06	8.93E-05
227749_at	POU2F2	0.585	1.5	1.60E-05	2.63E-04
228984_at	CARNS1	0.585	1.5	3.45E-07	1.18E-05
225478_at	MFHAS1	-0.585	-1.5	2.01E-08	1.17E-06
209496_at	RARRES2	-0.586	-1.5	1.95E-04	1.85E-03
1556204_a_at	ZNF814	-0.586	-1.5	3.51E-04	2.89E-03
210074_at	CTSL2	-0.587	-1.5	1.49E-03	8.80E-03
218929_at	CDKN2AIP	-0.587	-1.5	6.17E-12	1.39E-09
228284_at	TLE1	-0.587	-1.5	2.05E-05	3.18E-04
209457_at	DUSP5	-0.587	-1.5	2.55E-05	3.79E-04

234923_at	RALGAPA1	-0.587	-1.5	9.12E-04	6.06E-03
232579_at	LOC100134229	-0.588	-1.5	2.84E-06	6.53E-05
219687_at	HHAT	-0.588	-1.5	2.89E-08	1.61E-06
232304_at	PELI1	-0.588	-1.5	3.58E-03	1.70E-02
1569126_at	CCNC	-0.588	-1.5	8.21E-04	5.56E-03
212686_at	PPM1H	-0.588	-1.5	2.62E-06	6.12E-05
214036_at	EFNA5	-0.588	-1.5	4.81E-08	2.43E-06
226541_at	FBXO30	-0.589	-1.5	1.68E-07	6.56E-06
202458_at	PRSS23	-0.589	-1.5	2.42E-06	5.73E-05
220431_at	TMPRSS11E	-0.589	-1.5	6.82E-03	2.76E-02
205138_s_at	UST	-0.589	-1.5	6.55E-09	4.53E-07
217787_s_at	GALNT2	-0.589	-1.5	2.30E-09	1.90E-07
242786_at	SBF2-AS1	-0.59	-1.51	5.36E-06	1.09E-04
223539_s_at	SERF1A /// SERF1B	-0.59	-1.51	8.49E-05	9.67E-04
219271_at	GALNT14	-0.59	-1.51	1.47E-07	5.92E-06
205586_x_at	VGF	-0.591	-1.51	2.01E-10	2.54E-08
236371_s_at	TGS1	-0.591	-1.51	2.63E-03	1.36E-02
202272_s_at	FBXO28	-0.592	-1.51	1.65E-07	6.48E-06
232322_x_at	STARD10	-0.592	-1.51	7.55E-07	2.24E-05
226077_at	RNF145	-0.592	-1.51	1.21E-08	7.65E-07
229093_at	NOS3	-0.592	-1.51	1.53E-06	3.94E-05
1553672_at	ENAH	-0.593	-1.51	4.30E-09	3.21E-07
208141_s_at	DOHH	-0.593	-1.51	2.03E-06	4.96E-05
210716_s_at	CLIP1	-0.593	-1.51	1.93E-04	1.83E-03
201167_x_at	ARHGDIA	-0.593	-1.51	1.00E-04	1.09E-03
209162_s_at	PRPF4	-0.594	-1.51	5.99E-06	1.19E-04
1553185_at	RASEF	-0.594	-1.51	2.09E-05	3.23E-04
1553425_at	WDR65	-0.594	-1.51	2.02E-10	2.54E-08
209118_s_at	TUBA1A	-0.594	-1.51	1.16E-09	1.10E-07
202290_at	PDAP1	-0.594	-1.51	7.82E-07	2.31E-05
226071_at	ADAMTSL4	-0.595	-1.51	4.78E-04	3.66E-03
1558540_s_at	SLC2A11	-0.595	-1.51	5.67E-08	2.82E-06
219775_s_at	CPLX3	-0.595	-1.51	2.03E-07	7.68E-06
212570_at	ENDOD1	-0.595	-1.51	7.26E-05	8.56E-04
214697_s_at	PTBP3	-0.596	-1.51	7.81E-05	9.08E-04
202207_at	ARL4C	-0.596	-1.51	1.24E-07	5.16E-06
226590_at	ZNF618	-0.598	-1.51	7.02E-05	8.34E-04
227032_at	PLXNA2	-0.598	-1.51	6.93E-08	3.29E-06
213999_at	YIPF4	-0.598	-1.51	1.63E-05	2.67E-04
228485_s_at	SLC44A1	-0.598	-1.51	1.79E-06	4.46E-05
213087_s_at	EEF1D	-0.599	-1.51	6.20E-04	4.47E-03
206116_s_at	TPM1	-0.599	-1.51	9.40E-04	6.20E-03

213096_at	TMCC2	-0.599	-1.51	1.62E-09	1.42E-07
243910_x_at	CAND1	-0.599	-1.52	4.55E-03	2.04E-02
206196_s_at	RUNDC3A	-0.6	-1.52	1.18E-05	2.05E-04
1568126_at	ANXA2	-0.602	-1.52	2.27E-04	2.07E-03
226592_at	ZNF618	-0.602	-1.52	1.03E-07	4.47E-06
228360_at	LYPD6B	-0.602	-1.52	1.57E-09	1.37E-07
218113_at	TMEM2	-0.603	-1.52	7.17E-08	3.39E-06
205157_s_at	JUP /// KRT17	-0.603	-1.52	5.25E-05	6.64E-04
213476_x_at	TUBB3	-0.604	-1.52	8.60E-10	8.57E-08
239355_at	GMCL1	-0.605	-1.52	2.12E-11	3.96E-09
210954_s_at	TSC22D2	-0.605	-1.52	4.12E-08	2.13E-06
224559_at	MALAT1	-0.605	-1.52	1.97E-04	1.87E-03
203692_s_at	E2F3	-0.605	-1.52	5.31E-05	6.70E-04
202461_at	EIF2B2	-0.605	-1.52	2.14E-09	1.78E-07
232249_at	FMNL3	-0.607	-1.52	5.55E-04	4.10E-03
241245_at	SRSF4	-0.607	-1.52	4.80E-03	2.12E-02
217080_s_at	HOMER2	-0.607	-1.52	2.90E-09	2.32E-07
203707_at	LINC00921 /// ZNF263	-0.608	-1.52	4.31E-10	4.84E-08
232168_x_at	MACF1	-0.608	-1.52	2.33E-04	2.12E-03
213344_s_at	H2AFX	-0.608	-1.52	9.32E-08	4.13E-06
212098_at	MGAT5	-0.609	-1.52	3.27E-13	1.11E-10
207511_s_at	CNPPD1	-0.609	-1.53	2.11E-07	7.92E-06
213887_s_at	POLR2E	-0.609	-1.53	1.74E-06	4.36E-05
201848_s_at	BNIP3	-0.609	-1.53	1.66E-10	2.17E-08
1554572_a_at	SUV39H2	-0.61	-1.53	4.11E-08	2.13E-06
212662_at	PVR	-0.61	-1.53	1.06E-05	1.89E-04
225830_at	PDZD8	-0.61	-1.53	2.33E-07	8.56E-06
206543_at	SMARCA2	-0.61	-1.53	2.02E-06	4.92E-05
228553_at	ENAH	-0.61	-1.53	5.66E-05	7.03E-04
207038_at	SLC16A6	-0.61	-1.53	2.70E-05	3.97E-04
211571_s_at	VCAN	-0.611	-1.53	2.91E-04	2.52E-03
1567107_s_at	TPM4	-0.611	-1.53	8.73E-06	1.62E-04
200664_s_at	DNAJB1	-0.612	-1.53	1.37E-08	8.52E-07
203705_s_at	FZD7	-0.612	-1.53	8.83E-05	9.97E-04
228699_at	NRP2	-0.613	-1.53	5.94E-05	7.29E-04
214359_s_at	HSP90AB1	-0.613	-1.53	4.95E-03	2.17E-02
224036_s_at	LMBR1	-0.614	-1.53	3.55E-05	4.88E-04
211793_s_at	ABI2	-0.614	-1.53	7.15E-10	7.34E-08
210419_at	BARX2	-0.614	-1.53	9.53E-09	6.16E-07
220456_at	SPTLC3	-0.614	-1.53	3.31E-08	1.79E-06
218861_at	RNF25	-0.614	-1.53	2.24E-07	8.31E-06
201939_at	PLK2	-0.615	-1.53	1.10E-05	1.94E-04

227461_at	STON2	-0.616	-1.53	1.10E-09	1.06E-07
244682_at	CAMSAP1	-0.616	-1.53	7.40E-04	5.12E-03
205195_at	AP1S1	-0.616	-1.53	1.20E-04	1.26E-03
240144_at	DNASE1	-0.616	-1.53	1.68E-04	1.63E-03
207112_s_at	GAB1	-0.617	-1.53	1.89E-07	7.27E-06
222164_at	FGFR1	-0.618	-1.53	8.62E-07	2.48E-05
219648_at	MREG	-0.618	-1.53	1.35E-17	3.85E-14
238513_at	PRRG4	-0.618	-1.53	5.43E-05	6.81E-04
242592_at	GPR137C	-0.618	-1.53	4.08E-06	8.83E-05
1558577_at	LOC148709	-0.618	-1.53	5.40E-10	5.84E-08
208908_s_at	CAST	-0.619	-1.54	8.62E-08	3.90E-06
208002_s_at	ACOT7	-0.619	-1.54	1.04E-10	1.53E-08
207764_s_at	HIPK3	-0.619	-1.54	2.02E-07	7.65E-06
215066_at	PTPRF	-0.62	-1.54	1.66E-03	9.51E-03
229290_at	DAPL1	-0.62	-1.54	8.83E-10	8.75E-08
203018_s_at	SSX2IP	-0.622	-1.54	6.07E-06	1.21E-04
203790_s_at	HRSP12	-0.623	-1.54	1.33E-06	3.54E-05
212365_at	MYO1B	-0.623	-1.54	7.69E-08	3.56E-06
1565939_at	C5orf22	-0.624	-1.54	4.43E-04	3.46E-03
52651_at	COL8A2	-0.625	-1.54	8.69E-07	2.50E-05
202345_s_at	FABP5 /// LOC101060453	-0.625	-1.54	1.13E-08	7.19E-07
1558732_at	MAP4K4	-0.628	-1.54	4.28E-03	1.95E-02
210417_s_at	PI4KB	-0.628	-1.55	9.25E-05	1.03E-03
206156_at	GJB5	-0.629	-1.55	2.93E-04	2.53E-03
1569519_at	LOC101059961 /// LOC101060202 /// LOC101060226 /// LOC101060362 /// LOC101060684 /// NBPF1 /// NBPF10 /// NBPF11 /// NBPF12 /// NBPF24 /// NBPF7 /// NBPF8 /// NBPF9	-0.63	-1.55	1.77E-05	2.84E-04
204117_at	PREP	-0.631	-1.55	9.28E-09	6.02E-07
226193_x_at	CBWD1 /// CBWD3 /// CBWD5 /// CBWD6 /// CBWD7 /// LOC100653334 /// LOC101060578	-0.631	-1.55	3.31E-06	7.39E-05
216554_s_at	ENO1	-0.631	-1.55	1.49E-03	8.79E-03
222757_s_at	ZAK	-0.632	-1.55	4.28E-04	3.37E-03
1555225_at	C1orf43	-0.632	-1.55	9.60E-06	1.74E-04
209882_at	RIT1	-0.633	-1.55	1.28E-06	3.42E-05
221664_s_at	F11R	-0.633	-1.55	1.65E-06	4.20E-05
37408_at	MRC2	-0.634	-1.55	1.50E-09	1.34E-07
214877_at	CDKAL1	-0.634	-1.55	1.53E-04	1.52E-03
222701_s_at	CHCHD7	-0.634	-1.55	1.64E-09	1.42E-07
220085_at	HELLS	-0.634	-1.55	3.66E-03	1.73E-02
206367_at	REN	-0.634	-1.55	1.29E-05	2.22E-04

224940_s_at	PAPPA	-0.635	-1.55	2.64E-06	6.15E-05
215646_s_at	VCAN	-0.635	-1.55	1.42E-03	8.51E-03
223925_s_at	MTPN	-0.635	-1.55	1.24E-03	7.68E-03
1553186_x_at	RASEF	-0.635	-1.55	2.49E-05	3.74E-04
238621_at	FMN1	-0.635	-1.55	1.12E-05	1.97E-04
226499_at	NRARP	-0.635	-1.55	7.15E-08	3.38E-06
203980_at	FABP4	-0.636	-1.55	1.10E-02	3.93E-02
227602_at	RAB7A	-0.636	-1.55	1.99E-03	1.09E-02
215028_at	SEMA6A	-0.637	-1.56	4.43E-04	3.46E-03
203243_s_at	PDLIM5	-0.637	-1.56	8.58E-03	3.26E-02
215807_s_at	PLXNB1	-0.637	-1.56	5.38E-05	6.76E-04
231259_s_at	CCND2	-0.639	-1.56	2.68E-04	2.36E-03
203722_at	ALDH4A1	-0.64	-1.56	4.08E-07	1.35E-05
244755_at	XPR1	-0.64	-1.56	8.68E-05	9.84E-04
208653_s_at	CD164	-0.64	-1.56	1.49E-06	3.85E-05
218192_at	IP6K2	-0.641	-1.56	4.60E-06	9.68E-05
208719_s_at	DDX17	-0.641	-1.56	1.26E-06	3.38E-05
205117_at	FGF1	-0.641	-1.56	3.36E-03	1.62E-02
222348_at	MAST4	-0.641	-1.56	7.71E-06	1.47E-04
213577_at	SQLE	-0.641	-1.56	3.54E-05	4.87E-04
1555401_at	SOHLH2	-0.641	-1.56	1.30E-09	1.20E-07
1559658_at	KATNBL1	-0.642	-1.56	2.69E-05	3.96E-04
224762_at	SERINC2	-0.643	-1.56	1.74E-09	1.49E-07
229817_at	ZNF608	-0.643	-1.56	1.30E-07	5.38E-06
238028_at	C6orf132	-0.643	-1.56	4.10E-07	1.35E-05
212081_x_at	PRRC2A	-0.643	-1.56	2.01E-07	7.64E-06
204557_s_at	DZIP1	-0.643	-1.56	8.65E-05	9.81E-04
244118_at	GABRA1	-0.643	-1.56	8.97E-09	5.87E-07
202089_s_at	SLC39A6	-0.643	-1.56	1.10E-05	1.94E-04
222364_at	SLC44A1	-0.643	-1.56	4.47E-04	3.48E-03
1564307_a_at	A2ML1	-0.644	-1.56	9.49E-03	3.52E-02
216086_at	SV2C	-0.645	-1.56	3.68E-07	1.24E-05
219671_at	HPCAL4	-0.646	-1.56	4.30E-10	4.84E-08
203016_s_at	SSX2IP	-0.646	-1.56	6.54E-10	6.79E-08
219340_s_at	CLN8	-0.646	-1.56	1.15E-09	1.09E-07
1553226_at	LINC00052	-0.646	-1.57	1.23E-06	3.32E-05
221455_s_at	WNT3	-0.646	-1.57	2.72E-15	2.01E-12
218484_at	NDUFA4L2	-0.647	-1.57	2.31E-04	2.10E-03
227313_at	CNPY4	-0.648	-1.57	1.25E-09	1.16E-07
209198_s_at	SYT11	-0.648	-1.57	1.00E-04	1.10E-03
1555151_s_at	TDH	-0.648	-1.57	1.47E-07	5.92E-06
1565717_s_at	FUS	-0.649	-1.57	1.81E-04	1.74E-03

224325_at	FZD8	-0.65	-1.57	1.16E-03	7.26E-03
218834_s_at	TMEM132A	-0.65	-1.57	6.14E-08	2.99E-06
204148_s_at	POMZP3 /// ZP3	-0.651	-1.57	9.14E-04	6.07E-03
203221_at	TLE1	-0.651	-1.57	2.49E-10	3.07E-08
202341_s_at	TRIM2	-0.651	-1.57	5.52E-04	4.08E-03
226069_at	PRICKLE1	-0.651	-1.57	6.16E-07	1.89E-05
217109_at	MUC4	-0.652	-1.57	1.82E-03	1.02E-02
220816_at	LPAR3	-0.652	-1.57	5.92E-07	1.84E-05
232865_at	AFF4	-0.653	-1.57	1.26E-04	1.32E-03
204903_x_at	ATG4B	-0.654	-1.57	1.57E-09	1.37E-07
207826_s_at	ID3	-0.655	-1.57	3.05E-10	3.63E-08
228293_at	DEPDC7	-0.655	-1.57	7.99E-09	5.36E-07
236402_at	BRAF	-0.655	-1.57	1.04E-06	2.91E-05
229929_at	SPSB4	-0.655	-1.58	6.37E-08	3.08E-06
226237_at	COL8A1	-0.656	-1.58	6.28E-03	2.59E-02
212144_at	SUN2	-0.656	-1.58	2.98E-08	1.65E-06
210929_s_at	AHSG	-0.656	-1.58	2.35E-07	8.61E-06
236241_at	MED31	-0.656	-1.58	7.58E-03	2.98E-02
208650_s_at	CD24	-0.657	-1.58	7.26E-06	1.41E-04
220317_at	LRAT	-0.657	-1.58	2.05E-08	1.19E-06
243496_at	RAB18	-0.657	-1.58	6.42E-05	7.74E-04
1563900_at	FAM83B	-0.657	-1.58	5.90E-03	2.47E-02
212841_s_at	PPFIBP2	-0.658	-1.58	1.04E-10	1.54E-08
202744_at	SLC20A2	-0.658	-1.58	3.04E-10	3.63E-08
206605_at	ENDOU	-0.659	-1.58	7.36E-09	5.02E-07
218916_at	ZNF768	-0.66	-1.58	3.09E-07	1.08E-05
204173_at	MYL6B	-0.66	-1.58	9.98E-08	4.36E-06
214446_at	ELL2	-0.66	-1.58	2.26E-07	8.34E-06
205007_s_at	CIB2	-0.661	-1.58	6.81E-09	4.69E-07
201229_s_at	ARIH2	-0.661	-1.58	7.84E-05	9.11E-04
242660_at	C10orf112	-0.661	-1.58	4.26E-03	1.94E-02
220482_s_at	SERGEF	-0.662	-1.58	1.18E-05	2.06E-04
206315_at	CRLF1	-0.662	-1.58	7.72E-09	5.21E-07
217897_at	FXYD6	-0.663	-1.58	6.59E-05	7.92E-04
41856_at	UNC5B	-0.665	-1.59	6.45E-07	1.97E-05
222833_at	LPCAT2	-0.665	-1.59	1.30E-05	2.22E-04
212983_at	HRAS	-0.666	-1.59	9.12E-08	4.06E-06
213486_at	COPG2IT1	-0.667	-1.59	1.42E-03	8.49E-03
204750_s_at	DSC2	-0.667	-1.59	2.18E-05	3.35E-04
208932_at	PPP4C	-0.668	-1.59	7.48E-04	5.16E-03
203634_s_at	CPT1A	-0.668	-1.59	4.66E-05	6.02E-04
229518_at	FAM46B	-0.668	-1.59	5.72E-11	9.27E-09
1552365_at	SCIN	-0.668	-1.59	5.21E-04	3.90E-03

213713_s_at	GLB1L2	-0.669	-1.59	5.14E-08	2.58E-06
228126_x_at	CTXN1	-0.67	-1.59	1.16E-08	7.40E-07
230976_at	AK8	-0.671	-1.59	2.98E-04	2.56E-03
200622_x_at	CALM1 /// CALM2 /// CALM3	-0.671	-1.59	1.36E-06	3.60E-05
223468_s_at	RGMA	-0.672	-1.59	2.48E-05	3.72E-04
228329_at	DAB1	-0.673	-1.59	5.13E-03	2.23E-02
214168_s_at	TJP1	-0.673	-1.59	3.23E-07	1.12E-05
201418_s_at	SOX4	-0.673	-1.59	2.36E-04	2.13E-03
230748_at	SLC16A6	-0.673	-1.59	2.21E-07	8.22E-06
230369_at	GPR161	-0.674	-1.59	2.68E-09	2.15E-07
226027_at	SWI5	-0.674	-1.6	5.54E-09	3.96E-07
215817_at	SERPINB13	-0.674	-1.6	8.79E-10	8.73E-08
219229_at	SLCO3A1	-0.674	-1.6	2.61E-11	4.71E-09
206109_at	FUT1	-0.675	-1.6	1.15E-10	1.65E-08
238775_at	ITFG1	-0.676	-1.6	3.10E-04	2.63E-03
233203_at	ROPN1	-0.676	-1.6	5.82E-04	4.26E-03
213069_at	HEG1	-0.677	-1.6	4.01E-09	3.05E-07
213038_at	RNF19B	-0.677	-1.6	3.50E-10	4.09E-08
1559433_at	APCDD1L-AS1	-0.678	-1.6	2.56E-04	2.27E-03
220021_at	TMC7	-0.678	-1.6	9.44E-09	6.11E-07
215374_at	PAPOLA	-0.679	-1.6	3.91E-03	1.82E-02
205348_s_at	DYNC1I1	-0.679	-1.6	2.42E-03	1.27E-02
236700_at	EIF3C	-0.679	-1.6	8.10E-03	3.12E-02
219806_s_at	SMCO4	-0.681	-1.6	1.50E-07	6.01E-06
242906_at	SESN3	-0.681	-1.6	9.15E-06	1.68E-04
205732_s_at	NCOA2	-0.681	-1.6	1.97E-07	7.53E-06
226313_at	C10orf35	-0.683	-1.61	9.07E-11	1.37E-08
232348_at	C18orf8	-0.683	-1.61	4.51E-11	7.54E-09
239899_at	RNF145	-0.683	-1.61	8.83E-07	2.53E-05
1554804_a_at	CLDN19	-0.684	-1.61	7.89E-06	1.50E-04
207011_s_at	PTK7	-0.684	-1.61	7.06E-07	2.12E-05
204181_s_at	ZBTB43	-0.684	-1.61	6.19E-03	2.56E-02
223599_at	TRIM6	-0.684	-1.61	1.13E-04	1.21E-03
235057_at	ITCH	-0.684	-1.61	4.96E-03	2.18E-02
228636_at	BHLHE22	-0.685	-1.61	2.57E-04	2.28E-03
1561030_at	TMC7	-0.686	-1.61	6.67E-06	1.30E-04
1559256_at	MAGI1	-0.687	-1.61	5.20E-09	3.78E-07
225105_at	C12orf75	-0.687	-1.61	3.22E-06	7.22E-05
228121_at	TGFB2	-0.688	-1.61	1.08E-05	1.92E-04
222927_s_at	CPLX3	-0.689	-1.61	1.28E-10	1.78E-08
228977_at	LOC729680	-0.689	-1.61	6.12E-06	1.21E-04
223478_at	TIMM8B	-0.69	-1.61	5.90E-06	1.18E-04

204556_s_at	DZIP1	-0.69	-1.61	4.17E-05	5.51E-04
225718_at	KIAA1715	-0.691	-1.61	9.55E-03	3.53E-02
229581_at	ELFN1	-0.691	-1.61	2.34E-06	5.58E-05
210868_s_at	ELOVL6	-0.691	-1.61	4.55E-09	3.35E-07
242881_x_at	LOC100506303 /// LOC100653149 /// LOC101060483 /// LOC400879 /// LOC440157	-0.691	-1.61	5.02E-03	2.20E-02
221539_at	EIF4EBP1	-0.691	-1.61	4.98E-12	1.15E-09
1565525_a_at	TCP11L2	-0.691	-1.61	3.37E-08	1.82E-06
203779_s_at	MPZL2	-0.692	-1.62	4.88E-07	1.57E-05
1553352_x_at	ERVW-1	-0.692	-1.62	1.41E-11	2.85E-09
223103_at	STARD10	-0.692	-1.62	2.31E-08	1.32E-06
213100_at	UNC5B	-0.692	-1.62	7.17E-07	2.15E-05
226026_at	DIRC2	-0.693	-1.62	1.86E-11	3.59E-09
201266_at	TXNRD1	-0.693	-1.62	2.74E-14	1.45E-11
215199_at	CALD1	-0.695	-1.62	8.70E-06	1.61E-04
239062_at	TNRC6C-AS1	-0.695	-1.62	2.74E-07	9.76E-06
230254_at	FAM26E	-0.696	-1.62	5.10E-03	2.22E-02
232914_s_at	SYTL2	-0.696	-1.62	3.63E-06	8.01E-05
222528_s_at	SLC25A37	-0.696	-1.62	4.14E-07	1.37E-05
217238_s_at	ALDOB	-0.696	-1.62	2.22E-07	8.23E-06
231771_at	GJB6	-0.699	-1.62	1.19E-09	1.12E-07
217871_s_at	MIF	-0.7	-1.62	8.27E-06	1.55E-04
204761_at	USP6NL	-0.701	-1.63	9.29E-12	2.04E-09
211672_s_at	ARPC4	-0.702	-1.63	8.17E-04	5.54E-03
202436_s_at	CYP1B1	-0.702	-1.63	4.56E-04	3.53E-03
36564_at	RNF19B	-0.702	-1.63	8.44E-10	8.45E-08
1557034_s_at	LOC100134445 /// LOC100288778 /// WASH1 /// WASH2P /// WASH3P /// WASH5P /// WASH7P	-0.702	-1.63	1.81E-04	1.74E-03
242583_at	STON2	-0.703	-1.63	1.07E-08	6.88E-07
209588_at	EPHB2	-0.703	-1.63	2.25E-06	5.39E-05
222506_at	LMBR1	-0.704	-1.63	3.71E-05	5.04E-04
202695_s_at	STK17A	-0.705	-1.63	1.66E-07	6.49E-06
205918_at	SLC4A3	-0.705	-1.63	8.40E-06	1.57E-04
219869_s_at	SLC39A8	-0.705	-1.63	5.30E-05	6.70E-04
219976_at	HOOK1	-0.705	-1.63	1.44E-06	3.74E-05
1558173_a_at	LUZP1	-0.705	-1.63	4.64E-06	9.75E-05
214972_at	MGEA5	-0.706	-1.63	2.64E-04	2.33E-03
215547_at	TSC22D2	-0.706	-1.63	6.66E-12	1.49E-09
220011_at	AUNIP	-0.707	-1.63	3.05E-06	6.94E-05
208978_at	CRIP2	-0.707	-1.63	6.20E-08	3.02E-06
222420_s_at	UBE2H	-0.707	-1.63	1.04E-09	1.02E-07

37425_g_at	CCHCR1	-0.707	-1.63	1.56E-09	1.37E-07
201860_s_at	PLAT	-0.707	-1.63	9.29E-07	2.64E-05
209873_s_at	PKP3	-0.708	-1.63	7.60E-08	3.53E-06
235367_at	MYPN	-0.708	-1.63	3.90E-13	1.27E-10
242163_at	THRAP3	-0.709	-1.63	1.19E-03	7.40E-03
209280_at	MRC2	-0.71	-1.64	5.42E-11	8.82E-09
215268_at	KIAA0754	-0.71	-1.64	1.43E-06	3.73E-05
213439_x_at	RUNDC3A	-0.71	-1.64	4.43E-10	4.95E-08
235684_s_at	SESN3	-0.71	-1.64	2.10E-07	7.88E-06
223044_at	SLC40A1	-0.711	-1.64	5.90E-12	1.34E-09
238001_at	KCTD6	-0.712	-1.64	5.54E-10	5.94E-08
235977_at	LONRF2	-0.713	-1.64	8.07E-07	2.36E-05
209078_s_at	TXN2	-0.713	-1.64	1.48E-05	2.46E-04
203021_at	SLPI	-0.713	-1.64	2.84E-07	1.01E-05
224397_s_at	TMTC1	-0.714	-1.64	2.98E-06	6.81E-05
213606_s_at	ARHGDIA	-0.715	-1.64	2.14E-05	3.30E-04
234925_at	LINC00917	-0.715	-1.64	1.88E-08	1.11E-06
202208_s_at	ARL4C	-0.716	-1.64	3.53E-11	6.06E-09
200907_s_at	PALLD	-0.716	-1.64	4.55E-08	2.32E-06
230622_at	MLLT4	-0.717	-1.64	1.76E-11	3.42E-09
1557944_s_at	CTNND1 /// TMX2-CTNND1	-0.717	-1.64	2.61E-04	2.31E-03
222162_s_at	ADAMTS1	-0.717	-1.64	1.42E-06	3.72E-05
204141_at	TUBB2A	-0.718	-1.65	3.13E-14	1.60E-11
201183_s_at	CHD4	-0.719	-1.65	5.91E-06	1.18E-04
227317_at	LMCD1	-0.719	-1.65	6.74E-06	1.32E-04
223278_at	GJB2	-0.721	-1.65	2.26E-09	1.88E-07
225950_at	SAMD8	-0.721	-1.65	7.26E-13	2.27E-10
226494_at	CAMSAP3	-0.721	-1.65	1.01E-07	4.42E-06
228527_s_at	SLC25A37	-0.722	-1.65	2.18E-08	1.26E-06
200835_s_at	MAP4	-0.722	-1.65	5.59E-04	4.12E-03
214698_at	PTBP3	-0.722	-1.65	2.86E-05	4.15E-04
230464_at	S1PR5	-0.722	-1.65	8.24E-06	1.54E-04
225119_at	CHMP4B	-0.722	-1.65	8.05E-07	2.36E-05
242899_at	SESN3	-0.722	-1.65	4.28E-06	9.14E-05
227443_at	LURAP1L	-0.722	-1.65	5.49E-09	3.93E-07
1565759_at	RPL13	-0.724	-1.65	3.35E-08	1.81E-06
1558387_at	UG0898H09	-0.725	-1.65	3.09E-04	2.62E-03
210655_s_at	FOXO3 /// FOXO3B	-0.725	-1.65	3.88E-07	1.29E-05
1555007_s_at	WDR66	-0.725	-1.65	2.90E-08	1.61E-06
210393_at	LGR5	-0.726	-1.65	4.40E-07	1.44E-05
235388_at	CHD9	-0.727	-1.65	3.84E-04	3.11E-03
201981_at	PAPPA	-0.727	-1.65	2.01E-07	7.63E-06

210338_s_at	HSPA8 /// SNORD14C /// SNORD14D	-0.729	-1.66	5.85E-04	4.27E-03
1552496_a_at	COBL	-0.729	-1.66	1.33E-06	3.52E-05
205422_s_at	ITGBL1	-0.73	-1.66	9.26E-06	1.69E-04
218888_s_at	NETO2	-0.73	-1.66	3.90E-06	8.51E-05
230068_s_at	PEG3-AS1	-0.73	-1.66	2.43E-07	8.82E-06
209591_s_at	BMP7	-0.731	-1.66	1.16E-06	3.18E-05
210910_s_at	POMZP3	-0.731	-1.66	1.33E-03	8.11E-03
230830_at	SLC51B	-0.732	-1.66	5.07E-10	5.56E-08
208621_s_at	EZR	-0.734	-1.66	3.24E-03	1.58E-02
227269_s_at	RAB40C	-0.734	-1.66	1.89E-06	4.66E-05
226876_at	FAM101B	-0.736	-1.66	6.86E-07	2.08E-05
224218_s_at	TRPS1	-0.736	-1.67	1.38E-05	2.32E-04
218368_s_at	TNFRSF12A	-0.736	-1.67	1.96E-09	1.66E-07
238789_at	KANK1	-0.736	-1.67	5.05E-06	1.05E-04
1570511_at	ARHGEF10L	-0.737	-1.67	9.20E-06	1.69E-04
1556037_s_at	HHIP	-0.739	-1.67	3.66E-08	1.94E-06
204736_s_at	CSPG4	-0.739	-1.67	9.30E-06	1.70E-04
205635_at	KALRN	-0.74	-1.67	3.91E-06	8.52E-05
239136_at	UNC5B-AS1	-0.74	-1.67	4.62E-04	3.57E-03
207008_at	CXCR2	-0.743	-1.67	3.98E-05	5.33E-04
224595_at	SLC44A1	-0.743	-1.67	6.46E-05	7.79E-04
222651_s_at	TRPS1	-0.743	-1.67	2.49E-05	3.73E-04
203904_x_at	CD82	-0.746	-1.68	1.50E-08	9.18E-07
204657_s_at	SHB	-0.746	-1.68	4.85E-09	3.54E-07
219225_at	PGBD5	-0.747	-1.68	1.28E-06	3.42E-05
1555730_a_at	CFL1	-0.747	-1.68	4.72E-04	3.62E-03
220203_at	BMP8A	-0.749	-1.68	3.25E-12	7.96E-10
212157_at	SDC2	-0.75	-1.68	1.66E-06	4.21E-05
220244_at	LINC00312	-0.751	-1.68	2.35E-06	5.60E-05
207192_at	DNASE1L2	-0.753	-1.69	5.70E-06	1.15E-04
224526_at	LOC100132319	-0.754	-1.69	9.54E-03	3.53E-02
219367_s_at	NRP2	-0.754	-1.69	2.96E-05	4.27E-04
220067_at	SPTBN5	-0.754	-1.69	5.28E-05	6.67E-04
226065_at	PRICKLE1	-0.755	-1.69	1.11E-08	7.11E-07
211362_s_at	SERPINB13	-0.755	-1.69	4.66E-10	5.14E-08
212154_at	SDC2	-0.756	-1.69	2.38E-05	3.59E-04
1555226_s_at	C1orf43	-0.757	-1.69	4.16E-05	5.50E-04
228786_at	PTCHD3P1	-0.757	-1.69	5.61E-07	1.76E-05
238593_at	C11orf80	-0.757	-1.69	6.42E-03	2.64E-02
220489_s_at	SERINC2	-0.758	-1.69	1.34E-07	5.54E-06
219660_s_at	ATP8A2	-0.758	-1.69	2.47E-06	5.82E-05

1568954_s_at	C16orf72	-0.759	-1.69	7.49E-10	7.60E-08
241771_at	RIMBP2	-0.759	-1.69	1.02E-07	4.44E-06
214433_s_at	SELENBP1	-0.759	-1.69	1.95E-05	3.06E-04
220625_s_at	ELF5	-0.76	-1.69	5.64E-06	1.14E-04
202416_at	DNAJC7	-0.761	-1.69	1.83E-10	2.34E-08
229900_at	CD109	-0.761	-1.69	5.49E-09	3.93E-07
207268_x_at	ABI2	-0.761	-1.7	1.76E-13	6.70E-11
216629_at	SRRM2	-0.762	-1.7	1.19E-06	3.23E-05
220962_s_at	PADI1	-0.764	-1.7	2.16E-11	4.02E-09
1553288_a_at	NYAP1	-0.764	-1.7	3.43E-07	1.17E-05
1564274_at	C9orf47	-0.768	-1.7	1.34E-06	3.54E-05
1557910_at	HSP90AB1	-0.769	-1.7	1.63E-03	9.41E-03
220407_s_at	TGFB2	-0.769	-1.7	1.81E-05	2.89E-04
226599_at	FHDC1	-0.771	-1.71	1.17E-11	2.44E-09
244758_at	SCAND3	-0.772	-1.71	5.33E-08	2.67E-06
206561_s_at	AKR1B10	-0.772	-1.71	1.50E-02	4.98E-02
200824_at	GSTP1	-0.772	-1.71	5.34E-05	6.73E-04
220022_at	ZNF334	-0.772	-1.71	4.14E-04	3.28E-03
223690_at	LTBP2	-0.773	-1.71	5.17E-07	1.64E-05
207233_s_at	MITF	-0.774	-1.71	3.76E-07	1.26E-05
231230_at	KCNK10	-0.774	-1.71	7.40E-08	3.47E-06
204268_at	S100A2	-0.775	-1.71	5.65E-07	1.77E-05
215198_s_at	CALD1	-0.776	-1.71	5.54E-08	2.76E-06
216222_s_at	MYO10	-0.776	-1.71	4.30E-05	5.66E-04
231935_at	ARPP21	-0.778	-1.71	1.94E-11	3.66E-09
226187_at	CDS1	-0.778	-1.71	6.57E-15	4.11E-12
236140_at	GCLM	-0.778	-1.71	4.28E-04	3.37E-03
243611_at	MICALCL	-0.778	-1.71	2.82E-10	3.42E-08
217230_at	EZR	-0.778	-1.71	3.83E-04	3.09E-03
241990_at	RHOV	-0.779	-1.72	3.82E-08	2.00E-06
218677_at	S100A14	-0.779	-1.72	3.66E-07	1.23E-05
217234_s_at	EZR	-0.779	-1.72	3.37E-04	2.80E-03
1555074_a_at	KCNH5	-0.779	-1.72	1.29E-05	2.22E-04
217294_s_at	ENO1	-0.78	-1.72	5.14E-04	3.86E-03
210372_s_at	TPD52L1	-0.782	-1.72	3.87E-06	8.45E-05
221920_s_at	SLC25A37	-0.782	-1.72	1.21E-07	5.07E-06
1553691_at	B3GALNT2	-0.782	-1.72	1.79E-12	4.82E-10
206137_at	RIMS2	-0.783	-1.72	1.00E-09	9.81E-08
1558388_a_at	UG0898H09	-0.783	-1.72	7.41E-04	5.13E-03
206375_s_at	HSPB3	-0.785	-1.72	2.33E-06	5.55E-05
218574_s_at	LMCD1	-0.785	-1.72	1.51E-07	6.02E-06
227524_at	FZD3	-0.786	-1.72	6.96E-08	3.30E-06

213358_at	SOGA2	-0.789	-1.73	6.89E-06	1.34E-04
203184_at	FBN2	-0.79	-1.73	7.60E-04	5.22E-03
213456_at	SOSTDC1	-0.79	-1.73	3.34E-07	1.15E-05
201105_at	LGALS1	-0.79	-1.73	3.96E-05	5.32E-04
201148_s_at	TIMP3	-0.791	-1.73	2.92E-07	1.03E-05
201559_s_at	CLIC4	-0.792	-1.73	8.06E-07	2.36E-05
220710_at	ANP32A-IT1	-0.794	-1.73	1.71E-05	2.78E-04
1566465_at	KCNK1	-0.795	-1.73	3.29E-04	2.75E-03
201846_s_at	RYBP	-0.795	-1.73	2.39E-06	5.68E-05
242141_at	HDAC2	-0.795	-1.73	1.74E-07	6.75E-06
209679_s_at	SMAGP	-0.797	-1.74	6.67E-09	4.60E-07
209243_s_at	PEG3	-0.798	-1.74	5.29E-07	1.67E-05
235095_at	CCDC64B	-0.799	-1.74	1.34E-09	1.23E-07
220518_at	ABI3BP	-0.8	-1.74	3.36E-05	4.69E-04
1556316_s_at	LOC284889	-0.802	-1.74	2.28E-06	5.46E-05
227499_at	FZD3	-0.803	-1.74	4.66E-08	2.37E-06
217893_s_at	AKIRIN1	-0.803	-1.74	1.58E-04	1.56E-03
216591_s_at	SDHC	-0.804	-1.75	2.35E-03	1.24E-02
203797_at	VSNL1	-0.805	-1.75	1.43E-08	8.79E-07
221803_s_at	NRBF2	-0.806	-1.75	3.01E-12	7.47E-10
219956_at	GALNT6	-0.806	-1.75	5.66E-06	1.14E-04
226390_at	STARD4	-0.807	-1.75	1.83E-06	4.56E-05
204733_at	KLK6	-0.807	-1.75	4.65E-04	3.59E-03
220527_at	MRPL20	-0.808	-1.75	8.32E-09	5.52E-07
205255_x_at	TCF7	-0.808	-1.75	2.43E-13	8.63E-11
206777_s_at	CRYBB2 /// CRYBB2P1	-0.81	-1.75	4.80E-03	2.12E-02
222784_at	SMOC1	-0.811	-1.76	1.92E-05	3.03E-04
237466_s_at	HHIP	-0.813	-1.76	1.02E-09	9.94E-08
1552487_a_at	BNC1	-0.813	-1.76	8.30E-08	3.79E-06
210978_s_at	TAGLN2	-0.813	-1.76	1.62E-04	1.59E-03
217185_s_at	ZNF259 /// ZNF259P1	-0.813	-1.76	8.53E-10	8.52E-08
1553711_a_at	FAM218A	-0.814	-1.76	4.72E-07	1.52E-05
211002_s_at	TRIM29	-0.815	-1.76	3.88E-05	5.23E-04
226968_at	KIF1B	-0.815	-1.76	6.37E-05	7.70E-04
229097_at	DIAPH3	-0.819	-1.76	5.28E-06	1.08E-04
204730_at	RIMS3	-0.82	-1.77	7.68E-14	3.26E-11
230498_at	MCHR1	-0.822	-1.77	4.68E-06	9.80E-05
201445_at	CNN3	-0.822	-1.77	5.39E-06	1.10E-04
235275_at	BMP8B	-0.826	-1.77	2.80E-08	1.57E-06
208055_s_at	HERC4	-0.827	-1.77	1.10E-05	1.94E-04
210276_s_at	TRIOBP	-0.829	-1.78	3.62E-11	6.17E-09
240024_at	SEC14L2	-0.829	-1.78	2.90E-05	4.20E-04

224941_at	PAPPA	-0.832	-1.78	2.50E-06	5.89E-05
1554524_a_at	OLFM3	-0.832	-1.78	1.05E-07	4.53E-06
202693_s_at	STK17A	-0.832	-1.78	1.64E-09	1.42E-07
1556186_s_at	EMC1	-0.833	-1.78	4.44E-04	3.47E-03
205899_at	CCNA1	-0.835	-1.78	1.60E-04	1.58E-03
203499_at	EPHA2	-0.838	-1.79	1.51E-10	2.02E-08
239155_at	CXADR	-0.839	-1.79	1.67E-06	4.23E-05
239913_at	SLC10A4	-0.839	-1.79	3.88E-09	2.96E-07
200644_at	MARCKSL1	-0.839	-1.79	8.62E-07	2.48E-05
201267_s_at	PSMC3	-0.84	-1.79	4.06E-04	3.24E-03
239707_at	SLC5A10	-0.84	-1.79	3.77E-16	4.49E-13
219382_at	SERTAD3	-0.84	-1.79	1.24E-07	5.17E-06
213836_s_at	WIPI1	-0.842	-1.79	2.91E-12	7.25E-10
219529_at	CLIC3	-0.843	-1.79	6.85E-08	3.26E-06
227069_at	CUX1	-0.844	-1.79	1.13E-06	3.10E-05
203742_s_at	LOC732360 /// TDG	-0.845	-1.8	7.47E-11	1.16E-08
223541_at	HAS3	-0.847	-1.8	1.33E-05	2.26E-04
1568617_a_at	CAMSAP3	-0.847	-1.8	3.44E-07	1.17E-05
229065_at	SLC35F3	-0.849	-1.8	1.34E-05	2.28E-04
208637_x_at	ACTN1	-0.852	-1.8	6.04E-06	1.20E-04
211367_s_at	CASP1	-0.853	-1.81	1.78E-07	6.91E-06
209273_s_at	ISCA1	-0.853	-1.81	7.65E-14	3.26E-11
231875_at	KIF21A	-0.854	-1.81	1.91E-10	2.42E-08
232069_at	KIF26A	-0.854	-1.81	2.33E-09	1.92E-07
206409_at	TIAM1	-0.854	-1.81	2.96E-04	2.55E-03
225496_s_at	SYTL2	-0.855	-1.81	5.56E-08	2.77E-06
226952_at	EAF1	-0.855	-1.81	7.83E-14	3.29E-11
222874_s_at	CLN8	-0.856	-1.81	9.71E-13	2.94E-10
206044_s_at	BRAF	-0.857	-1.81	5.77E-08	2.86E-06
221795_at	NTRK2	-0.86	-1.82	7.03E-05	8.34E-04
203354_s_at	PSD3	-0.861	-1.82	4.08E-03	1.88E-02
226637_at	UBE2H	-0.862	-1.82	3.16E-10	3.72E-08
208613_s_at	FLNB	-0.866	-1.82	6.85E-08	3.26E-06
206448_at	ZNF365	-0.868	-1.82	3.94E-05	5.29E-04
201615_x_at	CALD1	-0.868	-1.83	1.05E-05	1.87E-04
210948_s_at	LEF1	-0.869	-1.83	2.86E-11	5.07E-09
211071_s_at	MLLT11	-0.869	-1.83	2.82E-10	3.42E-08
231858_x_at	AP5B1	-0.87	-1.83	1.49E-04	1.50E-03
202206_at	ARL4C	-0.873	-1.83	1.64E-08	9.81E-07
208555_x_at	CST2	-0.873	-1.83	1.49E-15	1.28E-12
221114_at	AMBN	-0.874	-1.83	8.67E-06	1.61E-04
226623_at	PHYHIPL	-0.876	-1.83	4.15E-09	3.13E-07

228720_at	SORCS2	-0.876	-1.84	3.84E-07	1.28E-05
238460_at	FAM83A	-0.877	-1.84	2.44E-09	1.99E-07
219369_s_at	OTUB2	-0.878	-1.84	4.41E-09	3.26E-07
201616_s_at	CALD1	-0.881	-1.84	5.44E-10	5.86E-08
201341_at	ENC1	-0.883	-1.84	2.16E-06	5.22E-05
204132_s_at	FOXO3 /// FOXO3B	-0.883	-1.84	4.46E-06	9.43E-05
228303_at	GALNT6	-0.884	-1.85	2.09E-09	1.76E-07
231192_at	LPAR3	-0.888	-1.85	4.47E-19	3.35E-15
235022_at	FAM210A	-0.889	-1.85	2.17E-12	5.69E-10
209772_s_at	CD24	-0.889	-1.85	6.23E-07	1.91E-05
209098_s_at	JAG1	-0.897	-1.86	3.03E-09	2.40E-07
220245_at	SLC45A2	-0.9	-1.87	1.19E-08	7.50E-07
212531_at	LCN2	-0.901	-1.87	6.37E-04	4.56E-03
34478_at	RAB11B	-0.903	-1.87	8.99E-05	1.01E-03
239719_at	CD109	-0.904	-1.87	5.49E-10	5.90E-08
241854_at	DNASE1	-0.904	-1.87	1.33E-06	3.54E-05
209442_x_at	ANK3	-0.908	-1.88	4.58E-05	5.94E-04
226829_at	AFAP1L2	-0.908	-1.88	1.47E-16	2.02E-13
215271_at	TNN	-0.909	-1.88	4.03E-03	1.86E-02
216641_s_at	LAD1	-0.909	-1.88	3.57E-07	1.21E-05
1553915_at	C10orf126	-0.91	-1.88	1.44E-10	1.94E-08
233971_at	FAM166A	-0.914	-1.88	1.86E-06	4.60E-05
228900_at	SPECC1	-0.914	-1.88	1.18E-09	1.11E-07
244533_at	PTPN14	-0.914	-1.88	5.59E-04	4.12E-03
1552626_a_at	TMEM163	-0.916	-1.89	1.54E-05	2.55E-04
222774_s_at	NETO2	-0.918	-1.89	9.69E-08	4.26E-06
226767_s_at	FAHD1	-0.919	-1.89	5.77E-06	1.16E-04
1554334_a_at	DNAJA4	-0.92	-1.89	2.34E-13	8.39E-11
235075_at	DSG3	-0.922	-1.89	2.25E-10	2.80E-08
206758_at	EDN2	-0.927	-1.9	1.40E-09	1.27E-07
205534_at	PCDH7	-0.927	-1.9	7.14E-07	2.14E-05
206004_at	TGM3	-0.927	-1.9	3.27E-08	1.77E-06
214104_at	GPR161	-0.928	-1.9	2.47E-11	4.48E-09
211361_s_at	SERPINB13	-0.93	-1.91	6.63E-11	1.05E-08
226535_at	ITGB6	-0.932	-1.91	1.14E-09	1.08E-07
222783_s_at	SMOC1	-0.934	-1.91	3.07E-07	1.08E-05
211219_s_at	LHX2	-0.935	-1.91	3.32E-06	7.41E-05
203423_at	RBP1	-0.935	-1.91	7.94E-06	1.50E-04
202147_s_at	IFRD1	-0.936	-1.91	4.86E-12	1.13E-09
243541_at	IL31RA	-0.938	-1.92	3.02E-03	1.50E-02
209242_at	PEG3	-0.939	-1.92	6.16E-07	1.89E-05
203367_at	DUSP14	-0.941	-1.92	8.31E-16	8.30E-13

200906_s_at	PALLD	-0.945	-1.92	1.26E-07	5.24E-06
214939_x_at	MLLT4	-0.945	-1.93	2.89E-17	6.29E-14
205525_at	CALD1	-0.949	-1.93	5.92E-08	2.91E-06
228915_at	DACH1	-0.949	-1.93	2.44E-05	3.67E-04
200052_s_at	ILF2	-0.95	-1.93	6.16E-07	1.89E-05
219612_s_at	FGG	-0.956	-1.94	5.69E-07	1.78E-05
1555299_s_at	ERVW-1	-0.957	-1.94	4.15E-10	4.70E-08
211538_s_at	HSPA2	-0.957	-1.94	1.71E-12	4.69E-10
222853_at	FLRT3	-0.96	-1.95	5.62E-09	4.01E-07
231395_at	ATP8A2	-0.961	-1.95	6.83E-09	4.69E-07
212942_s_at	KIAA1199	-0.962	-1.95	8.46E-11	1.29E-08
207367_at	ATP12A	-0.962	-1.95	1.37E-03	8.26E-03
223078_s_at	TMOD3	-0.963	-1.95	3.86E-12	9.29E-10
239586_at	FAM83A	-0.966	-1.95	7.78E-09	5.25E-07
1552685_a_at	GRHL1	-0.968	-1.96	5.69E-09	4.04E-07
204351_at	S100P	-0.972	-1.96	1.92E-05	3.03E-04
233555_s_at	SULF2	-0.974	-1.96	4.56E-08	2.32E-06
1552511_a_at	CPA6	-0.976	-1.97	1.63E-13	6.28E-11
205676_at	CYP27B1	-0.977	-1.97	1.59E-04	1.57E-03
204378_at	BCAS1	-0.982	-1.98	2.36E-10	2.93E-08
232149_s_at	NSMAF	-0.985	-1.98	3.11E-09	2.46E-07
223503_at	TMEM163	-0.988	-1.98	4.35E-05	5.70E-04
203798_s_at	VSNL1	-0.988	-1.98	1.51E-08	9.24E-07
216504_s_at	SLC39A8	-0.989	-1.98	2.00E-05	3.12E-04
211764_s_at	UBE2D1	-0.99	-1.99	9.05E-14	3.76E-11
205334_at	S100A1	-0.993	-1.99	1.39E-06	3.65E-05
212807_s_at	SORT1	-0.996	-1.99	2.86E-10	3.44E-08
203222_s_at	TLE1	-0.997	-2	1.94E-09	1.65E-07
213759_at	ARL4C	-0.998	-2	2.76E-13	9.45E-11
223879_s_at	OXR1	-0.999	-2	3.50E-09	2.70E-07
204751_x_at	DSC2	-0.999	-2	3.89E-09	2.96E-07
205595_at	DSG3	-1	-2	1.63E-07	6.42E-06
1553493_a_at	TDH	-1	-2	2.51E-09	2.03E-07
216604_s_at	SLC7A8	-1	-2	1.21E-07	5.08E-06
205125_at	PLCD1	-1.01	-2.01	8.91E-15	5.22E-12
221102_s_at	TRPM6	-1.01	-2.01	1.32E-11	2.67E-09
214417_s_at	FETUB	-1.01	-2.01	1.70E-06	4.29E-05
200730_s_at	PTP4A1	-1.01	-2.01	2.68E-06	6.23E-05
205592_at	SLC4A1	-1.01	-2.01	1.77E-12	4.81E-10
204705_x_at	ALDOB	-1.02	-2.02	7.46E-07	2.22E-05
207195_at	CNTN6	-1.02	-2.02	9.46E-06	1.72E-04
205709_s_at	CDS1	-1.02	-2.02	1.50E-09	1.33E-07

201147_s_at	TIMP3	-1.02	-2.03	5.35E-08	2.68E-06
202435_s_at	CYP1B1	-1.02	-2.03	2.49E-04	2.22E-03
215150_at	YOD1	-1.02	-2.03	1.81E-10	2.34E-08
239726_at	ANK3	-1.03	-2.04	6.12E-05	7.46E-04
1553710_at	FAM218A	-1.03	-2.04	1.15E-07	4.88E-06
207291_at	PRRG4	-1.03	-2.04	1.06E-06	2.94E-05
202883_s_at	PPP2R1B	-1.03	-2.04	4.36E-15	2.88E-12
206140_at	LHX2	-1.03	-2.04	1.57E-03	9.13E-03
241412_at	BTC	-1.03	-2.04	2.93E-07	1.03E-05
223679_at	CTNNB1	-1.03	-2.05	5.74E-07	1.79E-05
206774_at	FRMPD1	-1.03	-2.05	5.75E-11	9.28E-09
229228_at	CREB5 /// LOC401317	-1.04	-2.05	2.07E-07	7.79E-06
229856_s_at	PITHD1	-1.04	-2.05	9.34E-11	1.40E-08
210387_at	NCALD	-1.04	-2.06	1.47E-07	5.92E-06
1555462_at	PPP1R1C	-1.04	-2.06	4.34E-07	1.42E-05
220999_s_at	CYFIP2	-1.04	-2.06	1.31E-08	8.20E-07
217924_at	C6orf106	-1.04	-2.06	7.11E-10	7.32E-08
203535_at	S100A9	-1.05	-2.07	6.26E-03	2.58E-02
226681_at	UBE2H	-1.05	-2.07	2.32E-11	4.30E-09
228910_at	CD82	-1.05	-2.07	1.29E-10	1.79E-08
212822_at	HEG1	-1.05	-2.07	6.60E-08	3.17E-06
228605_at	UBXN2A	-1.05	-2.08	9.88E-08	4.33E-06
205637_s_at	SH3GL3	-1.06	-2.08	1.05E-08	6.73E-07
218980_at	FHOD3	-1.06	-2.08	8.96E-07	2.56E-05
1554333_at	DNAJA4	-1.06	-2.08	1.90E-11	3.66E-09
223650_s_at	NRBF2	-1.06	-2.08	1.46E-12	4.12E-10
205941_s_at	COL10A1	-1.06	-2.09	5.84E-12	1.33E-09
202016_at	MEST	-1.06	-2.09	2.40E-07	8.76E-06
1557122_s_at	GABRB2	-1.07	-2.09	2.41E-07	8.77E-06
209875_s_at	SPP1	-1.07	-2.1	4.67E-04	3.60E-03
210335_at	RASSF9	-1.08	-2.11	6.18E-09	4.31E-07
212915_at	PDZRN3	-1.08	-2.11	2.07E-08	1.20E-06
205535_s_at	PCDH7	-1.08	-2.11	3.16E-09	2.49E-07
209126_x_at	KRT6B	-1.08	-2.11	1.46E-06	3.79E-05
224724_at	SULF2	-1.08	-2.11	3.35E-11	5.84E-09
224277_at	MOP-1	-1.08	-2.12	2.70E-15	2.01E-12
221389_at	PLA2G2E	-1.08	-2.12	4.53E-19	3.35E-15
224520_s_at	BEST3	-1.09	-2.12	1.08E-07	4.63E-06
200744_s_at	GNB1	-1.09	-2.12	6.45E-16	6.81E-13
204790_at	SMAD7	-1.09	-2.13	2.64E-13	9.19E-11
221558_s_at	LEF1	-1.09	-2.13	1.13E-10	1.63E-08
206504_at	CYP24A1	-1.1	-2.14	1.58E-07	6.27E-06

229942_at	BNC2	-1.1	-2.14	1.55E-08	9.38E-07
235591_at	SSTR1	-1.1	-2.14	1.05E-07	4.53E-06
206376_at	SLC6A15	-1.1	-2.14	1.58E-11	3.15E-09
219768_at	VTCN1	-1.1	-2.14	9.80E-05	1.07E-03
201043_s_at	ANP32A	-1.1	-2.15	4.32E-09	3.21E-07
228922_at	SHF	-1.1	-2.15	1.24E-10	1.75E-08
235479_at	CPEB2	-1.11	-2.15	1.08E-10	1.58E-08
238741_at	FAM83A	-1.11	-2.15	7.94E-09	5.33E-07
206089_at	NELL1	-1.11	-2.15	3.79E-04	3.07E-03
241985_at	JMY	-1.11	-2.16	3.33E-11	5.83E-09
227410_at	FAM43A	-1.11	-2.16	3.54E-14	1.75E-11
1556421_at	LOC286189	-1.12	-2.17	2.04E-08	1.19E-06
242344_at	GABRB2	-1.12	-2.17	2.73E-07	9.75E-06
232361_s_at	EHF	-1.12	-2.17	1.36E-04	1.39E-03
218384_at	CARHSP1	-1.13	-2.18	2.66E-09	2.15E-07
203827_at	WIPI1	-1.13	-2.19	5.45E-15	3.54E-12
219842_at	ARL15	-1.13	-2.19	2.71E-11	4.87E-09
232378_at	SLC5A9	-1.13	-2.19	1.79E-06	4.45E-05
201110_s_at	THBS1	-1.13	-2.19	9.43E-07	2.67E-05
226698_at	FCHSD1	-1.14	-2.2	2.87E-11	5.07E-09
213385_at	CHN2	-1.14	-2.2	1.25E-09	1.16E-07
220782_x_at	KLK12	-1.15	-2.21	1.36E-06	3.60E-05
231867_at	TENM2	-1.15	-2.22	1.22E-07	5.09E-06
213457_at	MFHAS1	-1.15	-2.22	1.39E-08	8.62E-07
222015_at	CSNK1E	-1.15	-2.22	3.83E-13	1.26E-10
216258_s_at	SERPINB13	-1.15	-2.22	7.41E-11	1.16E-08
210521_s_at	FETUB	-1.15	-2.22	6.06E-08	2.96E-06
220800_s_at	TMOD3	-1.16	-2.23	5.49E-12	1.26E-09
237981_at	CMYA5	-1.16	-2.23	3.58E-09	2.76E-07
227955_s_at	EFNA5	-1.16	-2.23	4.79E-12	1.13E-09
217933_s_at	LAP3	-1.16	-2.24	2.97E-14	1.54E-11
220129_at	CCDC169-SOHLH2 /// SOHLH2	-1.17	-2.25	2.28E-08	1.30E-06
223427_s_at	EPB41L4B	-1.17	-2.25	2.43E-10	3.00E-08
236651_at	KALRN	-1.17	-2.25	3.50E-05	4.83E-04
228653_at	SAMD5	-1.17	-2.25	1.14E-06	3.12E-05
217799_x_at	UBE2H	-1.17	-2.25	1.69E-11	3.34E-09
230138_at	NCS1	-1.18	-2.27	1.08E-13	4.41E-11
220038_at	C8orf44-SGK3 /// SGK3	-1.19	-2.28	1.97E-06	4.82E-05
233340_at	SPINK13	-1.19	-2.29	4.64E-09	3.42E-07
227961_at	CTSB	-1.19	-2.29	5.93E-06	1.18E-04
229084_at	CNTN4	-1.2	-2.29	3.95E-05	5.31E-04
239028_at	LYPD6	-1.2	-2.29	1.78E-13	6.73E-11
208216_at	DLX4	-1.2	-2.29	3.27E-09	2.55E-07

239082_at	FZD3	-1.2	-2.3	7.72E-08	3.57E-06
228068_at	GOLGA7B	-1.2	-2.3	5.58E-14	2.46E-11
236255_at	PLEKHG4B	-1.2	-2.3	3.55E-10	4.14E-08
220272_at	BNC2	-1.2	-2.3	4.33E-08	2.23E-06
209343_at	EFHD1	-1.21	-2.32	1.69E-05	2.75E-04
217395_at	MT4	-1.21	-2.32	1.21E-12	3.52E-10
203650_at	PROCR	-1.22	-2.32	8.64E-16	8.40E-13
201149_s_at	TIMP3	-1.22	-2.33	1.46E-07	5.90E-06
221425_s_at	ISCA1	-1.23	-2.34	1.77E-17	4.67E-14
203413_at	NELL2	-1.23	-2.34	1.25E-06	3.36E-05
208708_x_at	EIF5	-1.23	-2.35	2.30E-18	8.50E-15
229163_at	CAMK2N1	-1.24	-2.35	2.74E-06	6.33E-05
1553539_at	KRT74	-1.24	-2.36	6.52E-10	6.79E-08
226122_at	PLEKHG1	-1.25	-2.38	5.22E-14	2.35E-11
215034_s_at	TM4SF1	-1.25	-2.39	8.63E-09	5.69E-07
230135_at	HHIP	-1.26	-2.39	5.38E-09	3.86E-07
231743_at	WNT3	-1.26	-2.39	8.39E-18	2.58E-14
230469_at	RTKN2	-1.26	-2.39	3.70E-07	1.24E-05
234316_x_at	KLK12	-1.26	-2.39	1.43E-08	8.82E-07
206935_at	PCDH8	-1.26	-2.39	6.61E-05	7.93E-04
242329_at	CREB5 /// LOC401317	-1.26	-2.4	3.15E-10	3.72E-08
219250_s_at	FLRT3	-1.26	-2.4	2.45E-12	6.34E-10
209387_s_at	TM4SF1	-1.26	-2.4	7.57E-09	5.13E-07
205471_s_at	DACH1	-1.26	-2.4	1.02E-07	4.43E-06
213793_s_at	HOMER1	-1.27	-2.41	1.18E-08	7.49E-07
214636_at	CALCB	-1.27	-2.41	2.02E-12	5.38E-10
214226_at	PRSS53	-1.27	-2.42	9.69E-15	5.60E-12
1552807_a_at	SIGLEC10	-1.28	-2.43	6.06E-11	9.73E-09
225295_at	SLC39A10	-1.28	-2.43	1.14E-12	3.33E-10
215779_s_at	HIST1H2BC /// HIST1H2BE /// HIST1H2BF /// HIST1H2BG /// HIST1H2BI /// NCALD	-1.28	-2.43	3.56E-09	2.74E-07
228996_at	RC3H1	-1.29	-2.44	1.53E-13	5.95E-11
206912_at	FOXE1	-1.29	-2.44	7.04E-08	3.34E-06
239203_at	LSMEM1	-1.3	-2.46	1.37E-09	1.25E-07
236263_at	SHH	-1.3	-2.46	2.48E-08	1.40E-06
227950_at	UBE2H	-1.3	-2.46	1.36E-14	7.74E-12
212478_at	RMND5A	-1.3	-2.47	1.51E-18	6.95E-15
232555_at	CREB5	-1.31	-2.48	6.00E-09	4.21E-07
209372_x_at	TUBB2A /// TUBB2B	-1.31	-2.48	3.08E-12	7.59E-10
230740_at	EHD3	-1.32	-2.49	7.24E-09	4.95E-07
222871_at	KLHDC8A	-1.32	-2.49	1.44E-11	2.88E-09
213880_at	LGR5	-1.32	-2.5	1.22E-05	2.12E-04

235683_at	SESN3	-1.33	-2.51	1.11E-11	2.35E-09
1554097_a_at	MIR31HG	-1.34	-2.53	8.50E-17	1.43E-13
243478_at	CHST2	-1.34	-2.53	1.05E-09	1.02E-07
1556533_at	LINC00868	-1.34	-2.54	1.76E-18	7.21E-15
214590_s_at	UBE2D1	-1.34	-2.54	4.00E-15	2.68E-12
219683_at	FZD3	-1.35	-2.54	4.36E-09	3.24E-07
235723_at	BNC2	-1.35	-2.54	1.11E-07	4.73E-06
233586_s_at	KLK12	-1.35	-2.55	1.92E-07	7.36E-06
230276_at	FAM49A	-1.35	-2.55	1.17E-10	1.66E-08
210689_at	CLDN14	-1.36	-2.57	4.23E-12	1.00E-09
237905_at	KRT25	-1.37	-2.58	4.38E-04	3.43E-03
216092_s_at	SLC7A8	-1.37	-2.58	5.82E-10	6.19E-08
242138_at	DLX1	-1.37	-2.59	3.69E-08	1.95E-06
220984_s_at	SLCO5A1	-1.38	-2.6	6.42E-11	1.03E-08
1569433_at	SAMD5	-1.39	-2.62	8.30E-10	8.36E-08
219301_s_at	CNTNAP2	-1.39	-2.62	1.73E-08	1.04E-06
218458_at	GMCL1	-1.41	-2.65	5.68E-15	3.62E-12
1558687_a_at	FOXN1	-1.41	-2.66	1.69E-16	2.23E-13
212479_s_at	RMND5A	-1.42	-2.67	8.05E-15	4.80E-12
240389_at	TRPM6	-1.42	-2.67	5.87E-08	2.89E-06
1569852_at	LSMEM1	-1.42	-2.68	3.90E-14	1.87E-11
206326_at	GRP	-1.43	-2.69	1.15E-03	7.22E-03
227650_at	HSPA14	-1.43	-2.7	1.00E-11	2.18E-09
234488_s_at	GMCL1 /// GMCL1P1	-1.43	-2.7	3.96E-15	2.68E-12
232568_at	MGC24103	-1.43	-2.7	1.24E-08	7.82E-07
221416_at	PLA2G2F	-1.44	-2.7	7.93E-17	1.40E-13
203130_s_at	KIF5C	-1.44	-2.71	8.99E-08	4.02E-06
200790_at	ODC1	-1.44	-2.72	1.87E-15	1.57E-12
219463_at	LAMP5	-1.45	-2.73	1.84E-13	6.86E-11
202752_x_at	SLC7A8	-1.45	-2.74	4.23E-10	4.78E-08
205931_s_at	CREB5 /// LOC401317	-1.46	-2.75	1.61E-08	9.66E-07
238568_s_at	NPC1	-1.46	-2.75	1.75E-14	9.64E-12
215785_s_at	CYFIP2	-1.47	-2.76	1.29E-13	5.18E-11
206882_at	SLC1A6	-1.47	-2.77	8.35E-13	2.55E-10
226887_at	HSPA14	-1.47	-2.78	9.41E-19	4.97E-15
219554_at	RHCG	-1.48	-2.78	2.52E-09	2.03E-07
225809_at	PARM1	-1.48	-2.79	1.83E-10	2.34E-08
231778_at	DLX3	-1.48	-2.79	3.06E-16	3.89E-13
229030_at	CAPN8	-1.52	-2.88	1.30E-06	3.46E-05
206604_at	OVOL1	-1.53	-2.89	1.07E-15	9.88E-13
233814_at	EFNA5	-1.53	-2.9	1.26E-11	2.59E-09
242062_at	SAMD8	-1.54	-2.91	2.46E-13	8.67E-11

1553494_at	TDH	-1.54	-2.91	4.82E-09	3.53E-07
213780_at	TCHH	-1.55	-2.92	3.17E-04	2.67E-03
217428_s_at	COL10A1	-1.55	-2.92	7.97E-11	1.22E-08
217031_at	KRT84	-1.55	-2.93	1.61E-09	1.40E-07
217678_at	SLC7A11	-1.57	-2.96	1.20E-10	1.70E-08
1553388_at	FAM26D	-1.58	-2.99	5.50E-14	2.45E-11
1553574_at	IFNE	-1.58	-3	4.45E-14	2.08E-11
1555774_at	ZAR1	-1.59	-3.02	2.56E-17	5.91E-14
208290_s_at	EIF5	-1.61	-3.05	5.75E-18	1.93E-14
237003_at	BEST3	-1.61	-3.06	1.05E-11	2.25E-09
223739_at	PADI1	-1.62	-3.07	3.91E-10	4.50E-08
243445_at	BNC2	-1.62	-3.07	2.46E-11	4.48E-09
205290_s_at	BMP2	-1.63	-3.1	3.70E-14	1.80E-11
209683_at	FAM49A	-1.63	-3.1	1.99E-13	7.29E-11
208250_s_at	DMBT1	-1.64	-3.11	5.17E-10	5.64E-08
1553829_at	CYP1B1-AS1	-1.64	-3.12	4.56E-10	5.04E-08
209618_at	CTNND2	-1.64	-3.13	7.66E-06	1.46E-04
220624_s_at	ELF5	-1.67	-3.19	2.22E-17	5.47E-14
224412_s_at	TRPM6	-1.67	-3.19	7.45E-09	5.06E-07
213774_s_at	PPP1R2	-1.68	-3.21	3.52E-16	4.34E-13
231461_at	KRT71	-1.7	-3.24	6.36E-05	7.70E-04
230722_at	BNC2	-1.7	-3.26	5.26E-09	3.81E-07
1557321_a_at	CAPN14	-1.71	-3.27	1.13E-11	2.37E-09
228975_at	SP6	-1.71	-3.27	6.16E-19	3.80E-15
203921_at	CHST2	-1.71	-3.27	2.80E-09	2.25E-07
209617_s_at	CTNND2	-1.72	-3.29	4.72E-08	2.39E-06
214576_at	KRT36	-1.72	-3.29	1.48E-15	1.28E-12
213909_at	LRRC15	-1.73	-3.31	3.87E-07	1.29E-05
1554592_a_at	SLC1A6	-1.73	-3.31	8.02E-13	2.47E-10
202884_s_at	PPP2R1B	-1.73	-3.31	5.01E-14	2.28E-11
232682_at	MREG	-1.73	-3.32	5.01E-16	5.61E-13
223720_at	SPINK7	-1.75	-3.36	2.65E-07	9.54E-06
213107_at	TNIK	-1.75	-3.37	2.29E-13	8.30E-11
238625_at	C1orf168	-1.76	-3.38	1.01E-13	4.14E-11
1554593_s_at	SLC1A6	-1.76	-3.39	4.32E-10	4.84E-08
233123_at	SLC40A1	-1.76	-3.39	6.97E-11	1.10E-08
1553861_at	TCP11L2	-1.77	-3.41	3.40E-17	6.98E-14
202886_s_at	PPP2R1B	-1.78	-3.43	1.31E-13	5.22E-11
209800_at	KRT16	-1.78	-3.43	6.08E-10	6.42E-08
209859_at	TRIM9	-1.78	-3.44	9.69E-10	9.55E-08
205724_at	PKP1	-1.78	-3.44	2.63E-15	2.01E-12
209757_s_at	MYCN	-1.79	-3.46	1.42E-09	1.28E-07

221962_s_at	UBE2H	-1.8	-3.48	4.53E-14	2.09E-11
210082_at	ABCA4	-1.8	-3.48	2.10E-12	5.53E-10
241014_at	FLG-AS1	-1.82	-3.52	9.23E-11	1.39E-08
233301_at	OXCT2	-1.82	-3.54	5.09E-17	9.58E-14
227506_at	SLC16A9	-1.83	-3.56	3.54E-14	1.75E-11
202376_at	SERPINA3	-1.85	-3.61	1.12E-07	4.76E-06
218935_at	EHD3	-1.86	-3.62	1.06E-12	3.18E-10
205289_at	BMP2	-1.86	-3.63	3.87E-12	9.29E-10
230493_at	SHISA2	-1.87	-3.65	3.25E-06	7.28E-05
228646_at	PPP1R1C	-1.93	-3.82	1.92E-11	3.66E-09
227763_at	LYPD6	-1.94	-3.84	4.41E-09	3.26E-07
238478_at	BNC2	-1.96	-3.88	2.11E-09	1.76E-07
219932_at	SLC27A6	-1.97	-3.92	4.64E-11	7.68E-09
221627_at	TRIM10	-1.99	-3.97	2.05E-10	2.57E-08
210319_x_at	MSX2	-2	-4.01	2.70E-13	9.34E-11
218266_s_at	NCS1	-2	-4.01	5.57E-16	6.05E-13
237732_at	PRR9	-2	-4.01	3.26E-05	4.59E-04
210311_at	FGF5	-2.01	-4.03	1.87E-14	1.01E-11
205555_s_at	MSX2	-2.02	-4.04	7.76E-16	7.97E-13
209981_at	CSDC2	-2.03	-4.09	2.01E-15	1.65E-12
229823_at	RIMS2	-2.04	-4.11	3.16E-10	3.72E-08
209921_at	SLC7A11	-2.04	-4.12	3.47E-13	1.17E-10
211357_s_at	ALDOB	-2.07	-4.19	2.82E-08	1.58E-06
222351_at	PPP2R1B	-2.07	-4.21	9.10E-11	1.37E-08
205374_at	SLN	-2.09	-4.25	3.11E-11	5.48E-09
204687_at	PARM1	-2.1	-4.29	3.65E-13	1.22E-10
211485_s_at	FGF18	-2.13	-4.38	4.01E-14	1.90E-11
229158_at	WNK4	-2.14	-4.41	3.05E-08	1.68E-06
202166_s_at	PPP1R2	-2.16	-4.48	1.73E-21	6.41E-17
224262_at	IL1F10	-2.16	-4.48	1.93E-11	3.66E-09
239572_at	GJA3	-2.2	-4.6	1.34E-12	3.81E-10
207065_at	KRT75	-2.23	-4.7	2.39E-09	1.96E-07
220090_at	CRNN	-2.26	-4.79	1.42E-10	1.93E-08
237507_at	KRT73	-2.27	-4.82	4.18E-09	3.15E-07
207147_at	DLX2	-2.27	-4.84	3.62E-10	4.21E-08
231583_at	KRT74	-2.29	-4.9	5.49E-06	1.12E-04
211429_s_at	SERPINA1	-2.32	-4.98	1.56E-09	1.37E-07
240388_at	KRT27	-2.34	-5.06	3.14E-06	7.08E-05
1564435_a_at	KRT72	-2.36	-5.12	1.90E-10	2.41E-08
228705_at	CAPN12	-2.37	-5.17	4.27E-16	4.93E-13
219832_s_at	HOXC13	-2.37	-5.18	1.35E-16	1.99E-13
1553537_at	KRT73	-2.38	-5.21	7.09E-15	4.30E-12

202833_s_at	SERPINA1	-2.42	-5.35	2.03E-08	1.18E-06
203304_at	BAMBI	-2.43	-5.38	7.76E-12	1.73E-09
219659_at	ATP8A2	-2.43	-5.4	1.30E-10	1.80E-08
229730_at	SMTNL2	-2.53	-5.76	6.86E-11	1.08E-08
204720_s_at	DNAJC6	-2.57	-5.95	1.26E-15	1.13E-12
1562628_at	KRT40	-2.58	-5.98	7.63E-13	2.37E-10
227764_at	LYPD6	-2.62	-6.14	3.82E-11	6.44E-09
206987_x_at	FGF18	-2.72	-6.58	1.08E-12	3.19E-10
211029_x_at	FGF18	-2.74	-6.69	1.07E-12	3.18E-10
1555775_a_at	ZAR1	-2.78	-6.89	5.18E-17	9.58E-14
220256_s_at	OXCT2	-2.9	-7.44	1.60E-20	2.96E-16
220635_at	PSORS1C2	-2.92	-7.57	2.49E-15	1.96E-12
207716_at	KRT38	-3.04	-8.24	2.76E-08	1.56E-06
206994_at	CST4	-3.07	-8.4	8.98E-17	1.44E-13
205713_s_at	COMP	-3.07	-8.42	7.70E-11	1.19E-08
208092_s_at	FAM49A	-3.08	-8.44	9.00E-16	8.53E-13
214284_s_at	FGF18	-3.12	-8.7	2.09E-11	3.92E-09
1564921_at	KRTAP13-1	-3.13	-8.78	2.26E-07	8.34E-06
206423_at	ANGPTL7	-3.21	-9.25	3.23E-08	1.76E-06
231382_at	FGF18	-3.24	-9.42	1.59E-11	3.16E-09
232887_at	PIRT	-3.27	-9.65	3.28E-15	2.33E-12
206027_at	S100A3	-3.32	-10	3.39E-14	1.72E-11
1554398_at	LYG2	-3.36	-10.2	7.00E-15	4.30E-12
230720_at	RNF182	-3.38	-10.4	2.42E-15	1.94E-12
214517_at	KRTAP5-9	-3.44	-10.9	2.98E-15	2.16E-12
242301_at	CBLN2	-3.53	-11.5	3.26E-07	1.13E-05
220779_at	PADI3	-3.54	-11.6	1.17E-10	1.66E-08
215189_at	KRT86 /// LOC100509764	-3.65	-12.5	8.47E-09	5.60E-07
1562629_a_at	KRT40	-3.77	-13.7	1.22E-11	2.51E-09
206677_at	KRT31	-3.95	-15.5	3.62E-11	6.17E-09
234685_x_at	KRTAP4-9	-4.01	-16.1	6.50E-07	1.98E-05
207146_at	KRT32	-4.01	-16.2	1.06E-11	2.27E-09
233631_x_at	KRTAP9-2 /// KRTAP9-8 /// LOC100996762	-4.03	-16.4	1.12E-06	3.08E-05
240967_at	KRTAP19-3	-4.08	-16.9	1.16E-08	7.40E-07
237853_x_at	KRTAP10-12	-4.14	-17.7	1.47E-16	2.02E-13
233681_at	KRTAP3-3	-4.15	-17.7	4.14E-07	1.36E-05
207670_at	KRT85	-4.22	-18.6	1.22E-09	1.13E-07
1556410_a_at	KRTAP19-1	-4.4	-21.1	3.16E-08	1.73E-06
234680_at	KRTAP17-1	-4.4	-21.1	1.30E-09	1.20E-07
216921_s_at	KRT35	-4.45	-21.8	2.55E-08	1.44E-06
233534_at	KRTAP3-2	-4.49	-22.5	2.26E-07	8.34E-06
206224_at	CST1	-4.5	-22.6	1.26E-19	1.56E-15

216810_at	KRTAP4-7	-4.5	-22.7	1.44E-07	5.83E-06
233158_at	KRT82	-4.52	-22.9	1.23E-12	3.55E-10
234772_s_at	KRTAP2-1 /// KRTAP2-2	-4.54	-23.2	1.31E-07	5.42E-06
206969_at	KRT34 /// LOC100653049	-4.61	-24.5	1.87E-08	1.11E-06
234633_at	KRTAP4-11	-4.66	-25.3	3.85E-10	4.44E-08
220978_at	KRTAP1-3	-4.69	-25.8	1.21E-07	5.08E-06
233533_at	KRTAP1-5	-4.71	-26.1	1.44E-07	5.83E-06
233537_at	KRTAP3-1	-4.71	-26.2	6.76E-08	3.24E-06
220970_s_at	KRTAP2-3 /// KRTAP2-4	-4.71	-26.2	7.63E-08	3.54E-06
234635_at	KRTAP4-1	-4.78	-27.4	1.01E-07	4.41E-06
234639_x_at	KRTAP9-8	-4.84	-28.7	3.68E-08	1.95E-06
220976_s_at	KRTAP1-1	-4.87	-29.2	4.64E-08	2.36E-06
233640_x_at	KRTAP9-4	-4.9	-29.8	4.09E-08	2.12E-06
234683_at	KRTAP4-11 /// KRTAP4-6 /// LOC100134267	-4.9	-29.9	1.09E-07	4.67E-06
234678_at	KRTAP4-3	-4.91	-30	4.25E-09	3.18E-07
1564803_at	KRTAP11-1	-4.93	-30.4	1.14E-09	1.08E-07
1560897_a_at	KRTAP10-11	-4.95	-30.9	2.82E-11	5.03E-09
224269_at	KRTAP4-12	-4.97	-31.4	4.38E-08	2.24E-06
234631_at	KRTAP4-8	-4.99	-31.7	1.31E-10	1.81E-08
208532_x_at	KRTAP5-8	-5.02	-32.4	6.02E-14	2.62E-11
219270_at	CHAC1	-5.04	-32.8	1.08E-16	1.66E-13
234679_at	KRTAP9-3	-5.07	-33.6	4.80E-09	3.52E-07
234691_at	KRTAP2-1	-5.16	-35.6	2.39E-09	1.96E-07
234637_at	KRTAP4-5	-5.17	-36	8.68E-09	5.70E-07
213711_at	KRT81	-5.2	-36.9	7.15E-13	2.26E-10
207787_at	KRT33B	-5.35	-40.7	1.40E-09	1.27E-07
220972_s_at	KRTAP9-9	-5.36	-41.1	5.76E-09	4.07E-07
1564960_at	KRTAP7-1	-5.46	-43.9	9.52E-12	2.08E-09
1561330_at	DSG4	-5.47	-44.4	1.87E-13	6.92E-11
1555673_at	KRTAP2-3 /// KRTAP2-4	-5.48	-44.5	1.64E-09	1.42E-07
234671_at	KRTAP4-2	-5.51	-45.5	1.76E-09	1.51E-07
234684_s_at	KRTAP4-4	-5.53	-46.2	3.03E-09	2.40E-07
207669_at	KRT83	-5.55	-46.7	1.50E-14	8.40E-12
207457_s_at	LY6G6D	-5.69	-51.6	3.74E-15	2.61E-12
208483_x_at	KRT33A	-5.75	-53.8	6.89E-13	2.19E-10
234880_x_at	KRTAP1-3	-5.89	-59.4	4.77E-13	1.54E-10
221297_at	GPRC5D	-6.01	-64.5	2.34E-14	1.25E-11
1564974_at	KRTAP8-1	-6.14	-70.5	1.12E-10	1.62E-08

Supplemental Table 3. Loading of Principal Components for Figure 1

Gene.Symbol	Entrez.Gene	PC1	PC2
KCNH8	131096	-0.001524302	-0.023413066
WFDC12	128488	0.01146765	0.02231481
TDH	157739	-0.011392322	-0.024839346
KRT73	319101	-0.021396358	-0.027002813
CYP1B1-AS1	285154	-0.012629837	-0.026268239
LYG2	254773	-0.038555675	-0.032552768
SLC1A6	6511	-0.020013964	-0.016243067
SLC1A6	6511	-0.01871392	-0.022948265
KRTAP2-3 /// KRTAP2-4	85294 /// 730755	-0.075419295	-0.059160096
ZAR1	326340	-0.024883389	-0.031093448
SRGAP2	23380	-0.009106197	0.021028605
KRTAP19-1	337882	-0.061268298	-0.049776857
CAPN14	440854	-0.017259484	-0.021555483
AHNAK2	113146	-0.013218277	0.021658322
KRTAP10-11	386678	-0.058488143	-0.063546521
DSG4	147409	-0.058597448	-0.064012895
KRT40	125115	-0.024677624	-0.030420554
KRT40	125115	-0.040095144	-0.052399425
RORA	6095	-0.003867528	0.022164673
KRT72	140807	-0.022043745	-0.036257658
KRTAP11-1	337880	-0.062015898	-0.057124623
KRTAP13-1	140258	-0.039256621	-0.043037888
KRTAP7-1	337878	-0.067303815	-0.043264077
KRTAP8-1	337879	-0.081012666	-0.046766017
LMF1	64788	0.014147629	-0.02325881
PPP1R2	5504	-0.019301048	-0.020359084
SERPINA3	12	-0.02537318	-0.022605859
SLC7A8	23428	-0.020453972	-0.009182535
SERPINA1	5265	-0.034168309	-0.020917568
PPP2R1B	5519	-0.01675194	-0.020598669
KIF5C	3800	-0.008461938	-0.028906367
BAMBI	25805	-0.019955658	-0.03741659
PSD3	23362	-0.020073362	0.015331869
S100A9	6280	-0.020940142	0.007951259
CXCL9	4283	0.034345955	0.018789279
CHST2	9435	-0.016240437	-0.03018589
COL11A1	1301	-0.006803536	-0.024465594
CXCL10	3627	0.032287696	0.013639861

MMP12	4321	0.022996553	0.019529245
CGA	1081	-0.007244098	-0.020866818
PARM1	25849	-0.026627347	-0.017838298
DNAJC6	9829	-0.023424736	-0.031224967
FGF1	2246	-0.002604998	-0.020120939
BMP2	650	-0.019339218	-0.024206278
BARD1	580	-0.007780222	0.020202192
DYNC1I1	1780	0.00128182	-0.021924673
SLN	6588	-0.021930804	-0.028013021
MSX2	4488	-0.016977738	-0.024204364
CYP27B1	1594	-0.01099425	-0.022430045
COMP	1311	-0.038958359	-0.034692982
PKP1	5317	-0.02185594	-0.008561197
ZNF264	9422	-0.011609698	0.021084477
CREB5 /// LOC401317	9586 /// 401317	-0.022026584	-0.01079736
MSX1	4487	0.002062228	-0.020661939
S100A3	6274	-0.036937821	-0.038266925
LHX2	9355	-0.008682195	-0.032763002
CST1	1469	-0.043708155	-0.041514303
RYR3	6263	0.006034532	-0.020813145
GRP	2922	-0.016207068	-0.031358896
CCL13	6357	0.020546228	0.014080331
ANGPTL7	10218	-0.042373608	-0.043950673
KRT31	3881	-0.044027617	-0.050991734
PCDH8	5100	-0.014749951	-0.025090169
KRT34 /// LOC100653049	3885 /// 100653049	-0.062675407	-0.057654477
FGF18	8817	-0.029676535	-0.034776424
CST4	1472	-0.027938737	-0.0327645
KRT75	9119	-0.025948441	-0.033408393
KRT32	3882	-0.043882591	-0.051803451
DLX2	1746	-0.018502521	-0.035240062
CNTN6	27255	-0.007855892	-0.02033424
LY6G6D	58530	-0.063324004	-0.060091514
KRT83	3889	-0.058142508	-0.064965094
KRT85	3891	-0.048283049	-0.055779878
KRT38	8687	-0.038379079	-0.036767604
KRT33B	3884	-0.067598942	-0.067484545
FAM49A	81553	-0.027388738	-0.040154985
DMBT1	1755	-0.013859746	-0.021298329
KRT33A	3883	-0.070423155	-0.056443699
KRTAP5-8	57830	-0.059175909	-0.051708999

KLF5	688	-0.005236923	0.021771197
EFHD1	80303	-0.004377294	-0.031619771
CTNND2	1501	-0.022736122	-0.018969404
CTNND2	1501	-0.01691292	-0.033317475
FAM49A	81553	-0.012972315	-0.020576476
HLA-DRB4	3126	0.0276375	0.019119355
MYCN	4613	-0.018360194	-0.025064914
GRIA1	2890	-0.003318441	-0.023634529
KRT16	3868	-0.025175952	-0.013922708
TRIM9	114088	-0.017206037	-0.02864486
TGFB2	7042	0.001962285	-0.020612141
SLC7A11	23657	-0.018728664	-0.024176405
CCL18 /// LOC101060271	6362 /// 101060271	0.021625793	0.027901451
CSDC2	27254	-0.01608149	-0.023881064
LOC101060589 /// SMG1	23049 /// 101060589	-0.014852727	0.02075315
ABCA4	24	-0.019620523	-0.022701089
LILRB2	10288	0.02024238	-0.001386092
FGF5	2250	-0.02018816	-0.02249281
MSX2	4488	-0.018387771	-0.024252611
FGF18	8817	-0.03099121	-0.033570249
ALDOB	229	-0.025718172	-0.024302997
SERPINA1	5265	-0.030107653	-0.02236958
FGF18	8817	-0.021598311	-0.025293331
SERPINB4	6318	-0.023499228	0.015210265
SPAG9	9043	-0.014159715	0.020809126
TNIK	23043	-0.017777551	-0.020191408
SEZ6L	23544	0.000776125	-0.021630037
COL9A2	1298	0.009869533	-0.022479887
KRT81	3887	-0.058355525	-0.063873539
TCHH	7062	-0.022558577	-0.031682057
HOMER1	9456	-0.020859152	0.001183053
LGR5	8549	-0.016773289	-0.02490914
LRRC15	131578	-0.023479012	-0.024422221
TBC1D30	23329	-0.001905264	-0.023687819
SST	6750	-0.003064783	-0.020361433
FGF18	8817	-0.036167421	-0.040571063
KRTAP5-9	3846	-0.034560332	-0.038824181
SLURP1	57152	-0.00053953	0.029507977
PRF1	5551	0.020077874	-0.003343446
EXPH5	23086	-0.007839454	0.020979568

KRT86 /// LOC100509764	3892 /// 100509764	-0.047555026	-0.045208406
TNN	63923	-0.005358564	-0.02926051
POU2F3	25833	-0.005964562	0.020341199
HCRP1	387535	-0.008744807	0.020895345
C1orf68	100129271	0.006643736	0.021009736
LINC00302	388699	0.016468567	0.020405039
KRTAP4-7	100132476	-0.065953833	-0.051697004
KRT35	3886	-0.052376865	-0.061537121
C1orf68	100129271	0.007575664	0.023588484
NCS1	23413	-0.021287628	-0.01360443
EHD3	30845	-0.017521973	-0.025356732
FHOD3	80206	-0.004552575	-0.022331992
CHAC1	79094	-0.05438228	-0.053414932
SLAMF8	56833	0.022460559	0.003984615
ZMAT3	64393	-0.014353186	0.022367161
ATP8A2	51761	-0.030687878	-0.025993912
VTCN1	79679	-0.007625127	-0.028997432
HOXC13	3229	-0.026155423	-0.023889012
SLC16A10	117247	-0.003132058	0.021703678
SLC27A6	28965	-0.024579762	-0.021042283
CRNN	49860	-0.017744509	-0.033241074
OXCT2	64064	-0.026894356	-0.029272282
PSORS1C2	170680	-0.031960638	-0.027697342
PADI3	51702	-0.039588825	-0.050505891
KRTAP2-3 /// KRTAP2-4	85294 /// 730755	-0.068002243	-0.053600632
KRTAP9-9	81870	-0.074055494	-0.061738501
KRTAP1-1	81851	-0.067118878	-0.061774608
KRTAP1-3	81850	-0.065911576	-0.058625484
GPRC5D	55507	-0.069910412	-0.062645489
UBE2H	7328	-0.02146762	-0.014479847
PPP2R1B	5519	-0.012702404	-0.028786372
IL1F10	84639	-0.022762133	-0.012482346
KRTAP4-12	83755	-0.069700736	-0.060746462
TRPM6	140803	-0.018385832	-0.026554921
LOC100132319	100132319	-0.00240889	-0.023776712
COL8A1	1295	-8.13E-05	-0.020593872
MIR612 /// NEAT1	283131 /// 693197	-0.011556263	0.021479359
SLC16A9	220963	-0.019779434	-0.02044738
LYPD6	130574	-0.022630992	-0.02857373
LYPD6	130574	-0.032980448	-0.029951741
CTSB	1508	-0.021543508	0.012203865

LMF1	64788	0.013991826	-0.024267742
MS4A1	931	0.021237065	-0.002813991
PPP1R1C	151242	-0.021564889	-0.018707331
SAMD5	389432	-0.012777582	-0.023252289
CAPN12	147968	-0.020346243	-0.03086003
CAPN8	388743	-0.013964521	-0.031832182
SLC25A13	10165	-0.00532952	0.020271778
CNTN4	152330	-0.01077022	-0.028823482
LIMS3 /// LIMS3-LOC440895 /// LIMS3L /// LOC100288570 /// LOC100507334 /// LOC440895	96626 /// 440895 /// 100271835 /// 100288570 /// 100288695 /// 100507334	0.00843126	-0.023865569
WNK4	65266	-0.019881364	-0.041605467
COL11A1	1301	-0.012881869	-0.023963247
SMTNL2	342527	-0.022550796	-0.038879456
RIMS2	9699	-0.019344738	-0.033093731
GFRA3	2676	0.000797875	-0.021746508
DDX17	10521	-0.012222068	0.020970614
HAPLN1	1404	-0.007359373	-0.023049162
FAM49A	81553	-0.009677161	-0.020937046
RTKN2	219790	-0.008863433	-0.024012323
SHISA2	387914	-0.023863057	-0.03264598
RNF182	221687	-0.034382137	-0.038812401
BNC2	54796	-0.025038914	-0.009851629
EHD3	30845	-0.010711936	-0.024029415
FGF18	8817	-0.035229164	-0.046576458
KRT71	112802	-0.020832202	-0.035673271
KRT74	121391	-0.027211587	-0.043891906
SERPINB6	5269	0.012148399	0.020038006
ARG1	383	-0.008033876	0.027614963
EHF	26298	-0.014393016	0.020930983
EHF	26298	-0.022105522	0.018042243
SLC5A9	200010	-0.008374956	-0.026309335
MGC24103	158295	-0.020807784	-0.011341111
PIRT	644139	-0.033757063	-0.039273987
SLC40A1	30061	-0.024343347	-0.006819826
KRT82	3888	-0.049407628	-0.056238822
OXCT2	64064	-0.014987322	-0.02107257
KRTAP1-5	83895	-0.066980639	-0.058734502
KRTAP3-2	83897	-0.064485202	-0.0561138

KRTAP3-1	83896	-0.062577842	-0.066428164
KRTAP9-2 /// KRTAP9-8 /// LOC100996762	83899 /// 83901 /// 100996762	-0.060721729	-0.048465992
KRTAP9-4	85280	-0.070314294	-0.055562615
KRTAP3-3	85293	-0.057289679	-0.057391815
EFNA5	1946	-0.020162894	-0.008717154
KRTAP4-8	728224	-0.065494204	-0.053353125
KRTAP4-11	653240	-0.062055983	-0.051065988
KRTAP4-1	85285	-0.068403994	-0.058284468
KRTAP4-5	85289	-0.069048246	-0.066137825
KRTAP9-8	83901	-0.06853258	-0.052281377
KRTAP4-2	85291	-0.075110951	-0.060129488
KRTAP4-3	85290	-0.068599769	-0.052302485
KRTAP9-3	83900	-0.069807278	-0.058151301
KRTAP17-1	83902	-0.055823734	-0.057383792
KRTAP4-11 /// KRTAP4-6 /// LOC100134267	81871 /// 653240 /// 100134267	-0.07041952	-0.059124013
KRTAP4-4	84616	-0.078455783	-0.055682129
KRTAP4-9	100132386	-0.059851237	-0.046259252
KRTAP2-1	81872	-0.070614316	-0.056041067
KRTAP2-1 /// KRTAP2-2	81872 /// 728279	-0.06319279	-0.059336389
KRTAP1-3	81850	-0.073427665	-0.057122807
CBLN3	643866	0.023624616	-0.004853244
SSTR1	6751	-0.006971892	-0.020167143
KALRN	8997	-0.011450717	-0.028797227
BEST3	144453	-0.012747434	-0.022991113
KRT73	319101	-0.017149675	-0.040864827
PRR9	574414	-0.026633486	-0.037848339
KRTAP10-12	386685	-0.046207678	-0.039577255
KRT25	147183	-0.016845684	-0.031593713
BNC2	54796	-0.029829109	-0.005143697
C1orf168	199920	-0.017236303	-0.020723632
SH3RF2	153769	-0.008912792	0.020790525
LSMEM1	286006	-0.010087775	-0.02233185
GJA3	2700	-0.023819691	-0.025964851
ANK3	288	-0.020781147	0.010047587
KRT27	342574	-0.031342423	-0.037072157
TRPM6	140803	-0.013860716	-0.024859436
KRTAP19-3	337970	-0.055646898	-0.038031819
FLG-AS1	339400	-0.015693709	-0.024175446
DLX1	1745	-0.009564261	-0.023425273

CBLN2	147381	-0.047098379	-0.050645357
BNC2	54796	-0.017066407	-0.021032853
CHST2	9435	-0.012286278	-0.020809599
IL31RA	133396	-0.00981094	-0.028147793
KANSL1	284058	-0.014162994	0.021145815
SHANK2	22941	0.012030759	-0.022181703
SCAF4	57466	-0.015796668	0.020025721
CCL18 /// LOC101060271	6362 /// 101060271	0.021138556	0.027739241
COL11A1	1301	-0.007141394	-0.02958902

Supplemental Table 4. AT/AU vs NC Differential Expression

ProbeSetID	GeneSymbol	logFC	F	p.value	BH_fdr
209728_at	HLA-DRB4	4.19	18.3	1.73E-05	1.78E-04
203915_at	CXCL9	3.95	15.5	1.10E-11	1.61E-09
204533_at	CXCL10	3.44	10.8	4.61E-11	5.31E-09
209924_at	CCL18 /// LOC101060271	2.94	7.66	1.36E-09	8.88E-08
32128_at	CCL18 /// LOC101060271	2.93	7.62	4.44E-09	2.36E-07
204580_at	MMP12	2.63	6.18	1.58E-06	2.59E-05
206407_s_at	CCL13	2.35	5.1	2.11E-10	1.91E-08
216714_at	CCL13	2.26	4.81	5.20E-10	4.08E-08
219386_s_at	SLAMF8	2.19	4.55	2.54E-12	4.59E-10
215666_at	HLA-DRB4	2.17	4.49	3.45E-04	1.94E-03
205758_at	CD8A	2.1	4.3	7.85E-09	3.74E-07
206749_at	CD1B	1.94	3.85	3.01E-07	6.98E-06
204869_at	PCSK2	1.93	3.82	1.12E-13	3.13E-11
228592_at	MS4A1	1.91	3.75	1.36E-04	9.31E-04
205821_at	KLRC4-KLRK1 /// KLRK1	1.89	3.7	8.79E-11	9.28E-09
235221_at	CBLN3	1.88	3.68	8.12E-09	3.85E-07
204655_at	CCL5	1.87	3.66	6.95E-10	5.19E-08
217147_s_at	TRAT1	1.86	3.62	3.18E-07	7.23E-06
1405_i_at	CCL5	1.85	3.6	7.49E-10	5.47E-08
227194_at	FAM3B	1.84	3.58	9.19E-12	1.39E-09
205987_at	CD1C	1.8	3.49	2.63E-11	3.31E-09
205831_at	CD2	1.8	3.48	3.80E-09	2.09E-07
216718_at	LINC00302	1.78	3.43	2.40E-06	3.64E-05
229641_at	CCBE1	1.78	3.43	3.82E-10	3.15E-08
213537_at	HLA-DPA1	1.77	3.41	9.18E-06	1.07E-04
216935_at	LINC00302	1.77	3.4	1.05E-06	1.86E-05
213475_s_at	ITGAL	1.76	3.38	1.71E-10	1.60E-08
228381_at	ATF7IP2 /// LOC100287628	1.74	3.34	4.88E-08	1.64E-06
214617_at	PRF1	1.74	3.33	1.67E-08	6.83E-07
210176_at	TLR1	1.71	3.28	2.15E-14	8.31E-12
206715_at	TFEC	1.71	3.27	2.71E-08	1.03E-06
1554748_at	CLCNKB	1.7	3.24	8.79E-08	2.62E-06
210247_at	SYN2	1.69	3.23	9.84E-09	4.45E-07
229437_at	MIR155 /// MIR155HG	1.69	3.22	3.50E-08	1.26E-06
229151_at	SLC14A1	1.69	3.22	2.30E-04	1.41E-03
205488_at	GZMA	1.69	3.22	3.96E-07	8.53E-06
205043_at	CFTR	1.68	3.22	8.12E-07	1.51E-05
210146_x_at	LILRB2	1.68	3.21	5.13E-08	1.71E-06
236285_at	KLHDC7B	1.66	3.17	2.95E-08	1.11E-06
216920_s_at	TARP /// TRGC2	1.66	3.16	2.71E-07	6.41E-06
219890_at	CLEC5A	1.65	3.15	4.92E-12	8.12E-10

204714_s_at	F5	1.64	3.11	2.37E-08	9.22E-07
229779_at	COL4A4	1.63	3.09	2.42E-08	9.39E-07
214321_at	NOV	1.62	3.08	1.95E-09	1.21E-07
215228_at	NHLH2	1.62	3.08	1.03E-07	2.95E-06
206069_s_at	ACADL	1.61	3.06	6.72E-10	5.09E-08
219607_s_at	MS4A4A	1.61	3.06	7.34E-09	3.54E-07
1552870_s_at	AXDND1	1.61	3.05	3.83E-05	3.35E-04
1559263_s_at	ZC3H12D	1.61	3.05	4.95E-11	5.56E-09
211657_at	CEACAM6	1.6	3.03	6.37E-06	7.90E-05
204891_s_at	LCK	1.59	3.02	3.34E-10	2.83E-08
216233_at	CD163	1.59	3.02	1.25E-08	5.37E-07
215049_x_at	CD163	1.59	3	7.05E-10	5.22E-08
238725_at	IRF1	1.58	3	4.54E-10	3.64E-08
220005_at	P2RY13	1.58	2.99	1.98E-10	1.82E-08
206545_at	CD28	1.57	2.96	8.29E-07	1.54E-05
1557570_a_at	LOC285084	1.56	2.96	1.41E-06	2.38E-05
211656_x_at	HLA-DQB1 /// LOC101060835	1.56	2.95	5.00E-10	3.95E-08
203645_s_at	CD163	1.55	2.93	1.44E-09	9.34E-08
211339_s_at	ITK	1.55	2.93	2.47E-07	5.96E-06
236295_s_at	NLRC3	1.55	2.93	6.43E-09	3.18E-07
230753_at	PATL2	1.54	2.9	1.02E-10	1.05E-08
244413_at	CLECL1	1.54	2.9	9.46E-09	4.32E-07
243585_at	ATP13A5	1.52	2.87	4.63E-10	3.71E-08
213240_s_at	KRT4	1.52	2.86	1.45E-06	2.43E-05
224555_x_at	IL37	1.51	2.85	5.84E-06	7.41E-05
242488_at	CHRM3	1.51	2.85	1.81E-06	2.89E-05
206637_at	P2RY14	1.51	2.85	1.44E-11	2.01E-09
214219_x_at	MAP4K1	1.51	2.84	2.50E-11	3.20E-09
210915_x_at	TRBC1	1.5	2.82	8.38E-09	3.93E-07
228943_at	MAP6	1.5	2.82	6.99E-10	5.20E-08
1558972_s_at	THEMIS	1.49	2.82	9.40E-07	1.70E-05
235080_at	CLUAP1	1.49	2.81	1.70E-08	6.91E-07
215806_x_at	TARP /// TRGC2	1.49	2.81	1.06E-06	1.87E-05
231776_at	EOMES	1.49	2.8	4.02E-06	5.51E-05
230983_at	FAM129C	1.48	2.8	9.43E-06	1.09E-04
219820_at	SLC6A16	1.48	2.8	6.55E-11	7.05E-09
213193_x_at	TRBC1	1.48	2.79	2.65E-08	1.01E-06
206134_at	ADAMDEC1	1.48	2.78	1.46E-06	2.44E-05
1555993_at	CACNA1D	1.47	2.78	8.96E-14	2.59E-11
227553_at	PIK3R5	1.47	2.78	3.11E-09	1.77E-07
203757_s_at	CEACAM6	1.47	2.78	4.38E-06	5.90E-05
227142_at	PLEKHG5	1.47	2.78	1.06E-10	1.08E-08
214038_at	CCL8	1.46	2.76	2.06E-07	5.19E-06

206296_x_at	MAP4K1	1.46	2.76	1.97E-10	1.81E-08
211796_s_at	TRBC1	1.46	2.75	1.21E-06	2.10E-05
211144_x_at	TARP /// TRGC2	1.46	2.74	5.36E-07	1.07E-05
221080_s_at	DENND1C	1.46	2.74	2.17E-09	1.31E-07
221470_s_at	IL37	1.45	2.74	3.26E-05	2.94E-04
204377_s_at	VPRBP	1.45	2.74	1.24E-11	1.79E-09
206666_at	GZMK	1.45	2.73	2.35E-05	2.28E-04
230391_at	CD84	1.45	2.73	5.74E-09	2.93E-07
228869_at	SNX20	1.44	2.72	3.89E-10	3.19E-08
204205_at	APOBEC3G	1.44	2.72	6.40E-10	4.88E-08
207981_s_at	ESRRG	1.44	2.72	1.49E-07	4.02E-06
227742_at	CLIC6	1.44	2.71	7.36E-08	2.30E-06
207957_s_at	PRKCB	1.44	2.71	8.89E-09	4.11E-07
227346_at	IKZF1	1.43	2.7	3.10E-09	1.76E-07
204446_s_at	ALOX5	1.43	2.7	2.87E-07	6.71E-06
231124_x_at	LY9	1.43	2.7	3.12E-08	1.16E-06
213539_at	CD3D	1.43	2.69	3.86E-10	3.18E-08
205890_s_at	GABBR1 /// UBD	1.43	2.69	5.05E-06	6.60E-05
239529_at	C5orf20 /// TIFAB	1.42	2.68	4.38E-09	2.33E-07
206761_at	CD96	1.42	2.67	8.64E-09	4.01E-07
230110_at	MCOLN2	1.42	2.67	1.34E-07	3.66E-06
213920_at	CUX2	1.41	2.66	1.76E-04	1.14E-03
1553081_at	WFDC12	1.41	2.65	4.56E-04	2.43E-03
226136_at	GLIPR1	1.41	2.65	2.40E-10	2.14E-08
207802_at	CRISP3	1.41	2.65	4.81E-04	2.52E-03
229499_at	CAPN13	1.41	2.65	1.06E-05	1.20E-04
214339_s_at	MAP4K1	1.41	2.65	8.39E-10	6.00E-08
230418_s_at	GALNT16	1.4	2.64	1.49E-08	6.22E-07
233052_at	DNAH8	1.4	2.64	6.13E-06	7.69E-05
209813_x_at	TARP	1.4	2.63	2.79E-07	6.56E-06
209606_at	CYTIP	1.39	2.63	2.25E-09	1.35E-07
206914_at	CRTAM	1.39	2.63	1.21E-08	5.23E-07
206082_at	HCP5	1.39	2.63	2.32E-11	3.00E-09
203471_s_at	PLEK	1.39	2.62	3.01E-09	1.73E-07
228362_s_at	FAM26F	1.39	2.62	2.05E-06	3.21E-05
236028_at	IBSP	1.39	2.62	6.33E-06	7.86E-05
227645_at	PIK3R5	1.39	2.62	1.37E-10	1.33E-08
205280_at	GLRB	1.39	2.61	1.52E-09	9.71E-08
238229_at	TMEM67	1.39	2.61	8.87E-09	4.11E-07
205804_s_at	TRAF3IP3	1.38	2.61	2.61E-07	6.23E-06
228094_at	AMICA1	1.38	2.6	1.32E-10	1.29E-08
226218_at	IL7R	1.37	2.59	7.80E-06	9.32E-05
231029_at	F5	1.37	2.59	1.12E-05	1.25E-04

232081_at	ABCG1	1.37	2.58	1.32E-11	1.88E-09
206227_at	CILP	1.37	2.58	5.53E-05	4.49E-04
1556472_s_at	SCML4	1.36	2.57	1.48E-07	3.99E-06
1552584_at	IL12RB1	1.36	2.57	2.51E-07	6.03E-06
210038_at	PRKCQ	1.36	2.56	2.42E-05	2.33E-04
205180_s_at	ADAM8	1.36	2.56	9.35E-09	4.28E-07
227353_at	TMC8	1.35	2.56	1.82E-08	7.32E-07
229383_at	1-Mar	1.35	2.55	6.01E-10	4.62E-08
214081_at	PLXDC1	1.34	2.54	2.82E-06	4.15E-05
204424_s_at	LMO3	1.34	2.54	1.09E-05	1.22E-04
241418_at	LOC344887	1.34	2.53	2.83E-05	2.64E-04
203416_at	CD53	1.34	2.53	4.96E-09	2.59E-07
1556666_a_at	LOC101059957 /// TTC6	1.34	2.53	2.73E-06	4.06E-05
214044_at	RYR2	1.34	2.52	1.61E-08	6.62E-07
214203_s_at	PRODH	1.34	2.52	4.65E-09	2.44E-07
1560396_at	KLHL6	1.33	2.51	4.01E-06	5.51E-05
1557474_at	LOC284578	1.33	2.51	7.28E-07	1.39E-05
231348_s_at	LMO3	1.33	2.51	1.97E-05	1.97E-04
214399_s_at	KRT4	1.33	2.51	4.75E-06	6.28E-05
210164_at	GZMB	1.33	2.51	5.32E-07	1.07E-05
235276_at	EPSTI1	1.32	2.5	2.71E-10	2.36E-08
1566671_a_at	PDXK	1.32	2.5	1.56E-05	1.63E-04
1561306_s_at	STRC	1.32	2.5	1.18E-08	5.16E-07
220485_s_at	SIRPG	1.32	2.5	2.27E-07	5.59E-06
207908_at	KRT2	1.32	2.5	1.17E-06	2.04E-05
236782_at	SAMD3	1.32	2.5	4.17E-06	5.67E-05
44673_at	SIGLEC1	1.32	2.49	4.43E-08	1.51E-06
230799_at	LOC100134259	1.32	2.49	7.54E-09	3.63E-07
214567_s_at	XCL1 /// XCL2	1.31	2.48	3.03E-07	7.00E-06
219385_at	SLAMF8	1.31	2.48	1.69E-09	1.07E-07
241981_at	FAM20A	1.31	2.48	8.54E-07	1.57E-05
1562364_at	GVINP1	1.31	2.48	2.27E-07	5.59E-06
214873_at	LRP5L	1.31	2.47	2.99E-06	4.35E-05
203761_at	SLA	1.3	2.47	9.13E-11	9.55E-09
204160_s_at	ENPP4	1.3	2.46	3.04E-07	7.01E-06
220780_at	PLA2G3	1.3	2.46	1.80E-08	7.25E-07
204777_s_at	MAL	1.3	2.46	2.03E-09	1.24E-07
212975_at	DENND3	1.29	2.45	5.70E-09	2.91E-07
205364_at	ACOX2	1.29	2.44	2.54E-05	2.42E-04
204411_at	KIF21B	1.29	2.44	1.39E-07	3.80E-06
236175_at	TRIM55	1.29	2.44	4.26E-05	3.66E-04
238983_at	NSUN7	1.28	2.43	2.09E-07	5.24E-06
228298_at	PCED1B	1.28	2.43	1.31E-07	3.61E-06

201884_at	CEACAM5	1.28	2.42	1.04E-06	1.84E-05
235291_s_at	FLJ32255	1.28	2.42	6.70E-07	1.29E-05
1559194_a_at	CLEC4GP1	1.28	2.42	1.90E-06	3.01E-05
236270_at	NFATC4	1.27	2.42	1.61E-10	1.52E-08
204994_at	MX2	1.27	2.41	8.36E-10	5.99E-08
229391_s_at	FAM26F	1.27	2.41	4.17E-06	5.67E-05
229510_at	MS4A14	1.26	2.4	7.52E-07	1.42E-05
242268_at	CELF2	1.26	2.4	9.64E-08	2.81E-06
209969_s_at	STAT1	1.26	2.39	1.69E-08	6.90E-07
236081_at	SNCA	1.26	2.39	7.47E-07	1.42E-05
202524_s_at	SPOCK2	1.26	2.39	9.43E-08	2.76E-06
230560_at	STXBP6	1.26	2.39	2.17E-08	8.58E-07
1564474_at	ZBED3-AS1	1.25	2.38	1.36E-04	9.29E-04
204959_at	MNDA	1.25	2.37	2.01E-10	1.83E-08
244297_at	ANKRD18A	1.24	2.37	1.62E-06	2.64E-05
226311_at	ADAMTS2	1.24	2.37	9.35E-09	4.28E-07
206366_x_at	XCL1	1.24	2.36	1.60E-07	4.25E-06
240197_at	SYN2	1.23	2.35	5.33E-07	1.07E-05
220330_s_at	SAMSN1	1.23	2.35	6.36E-05	5.02E-04
214450_at	CTSW	1.23	2.35	1.28E-06	2.19E-05
213830_at	YME1L1	1.23	2.34	3.50E-05	3.11E-04
211200_s_at	EFCAB2	1.22	2.34	2.76E-04	1.63E-03
213960_at	NTRK3	1.22	2.33	1.98E-05	1.98E-04
204440_at	CD83	1.22	2.33	3.45E-09	1.92E-07
227647_at	KCNE3	1.22	2.33	3.91E-07	8.45E-06
212671_s_at	HLA-DQA1 /// HLA-DQA2 /// LOC100509457	1.22	2.33	2.55E-05	2.43E-04
1556474_a_at	FLJ38379	1.22	2.33	5.34E-07	1.07E-05
217059_at	MUC7	1.22	2.33	6.48E-03	2.03E-02
218795_at	ACP6	1.22	2.32	5.63E-08	1.86E-06
207277_at	CD209	1.22	2.32	7.87E-08	2.42E-06
206513_at	AIM2	1.21	2.32	3.36E-06	4.78E-05
229254_at	MFSD4	1.21	2.32	7.32E-08	2.29E-06
215784_at	CD1E	1.21	2.32	9.30E-06	1.08E-04
242641_at	COL6A6	1.21	2.31	1.55E-04	1.04E-03
230550_at	MS4A6A	1.21	2.31	2.45E-07	5.93E-06
209823_x_at	HLA-DQB1 /// LOC101060835	1.21	2.31	5.36E-07	1.07E-05
221978_at	HLA-F	1.2	2.31	7.72E-11	8.22E-09
213723_s_at	IDUA	1.2	2.3	1.71E-07	4.44E-06
220577_at	GVINP1	1.2	2.3	3.26E-08	1.19E-06
227210_at	SFMBT2	1.2	2.3	4.08E-10	3.31E-08
206118_at	STAT4	1.2	2.3	1.91E-07	4.86E-06
211743_s_at	PRG2	1.2	2.3	6.01E-08	1.95E-06
229723_at	TAGAP	1.2	2.3	9.77E-07	1.75E-05

209785_s_at	PLA2G4C	1.2	2.3	3.78E-07	8.24E-06
204279_at	PSMB9	1.2	2.29	1.07E-10	1.09E-08
218627_at	DRAM1	1.2	2.29	3.44E-12	6.05E-10
223122_s_at	SFRP2	1.2	2.29	3.30E-06	4.70E-05
206143_at	SLC26A3	1.19	2.29	5.24E-04	2.70E-03
220003_at	LRRC36	1.19	2.29	3.40E-07	7.58E-06
207710_at	LCE2B	1.19	2.29	5.73E-05	4.61E-04
208438_s_at	FGR	1.19	2.28	1.35E-09	8.85E-08
205269_at	LCP2	1.19	2.27	2.52E-08	9.70E-07
209879_at	SELPLG	1.18	2.27	1.30E-09	8.58E-08
214913_at	ADAMTS3	1.18	2.27	1.47E-09	9.50E-08
205270_s_at	LCP2	1.18	2.27	6.20E-09	3.09E-07
219704_at	YBX2	1.18	2.27	1.58E-06	2.60E-05
244572_at	KY	1.18	2.26	2.10E-07	5.25E-06
206587_at	CCT6B	1.18	2.26	1.05E-09	7.19E-08
206785_s_at	KLRC1 /// KLRC2	1.18	2.26	3.22E-08	1.18E-06
219666_at	MS4A6A	1.18	2.26	3.07E-07	7.07E-06
227183_at	MIR143HG /// MIR145	1.17	2.26	1.27E-05	1.38E-04
204663_at	ME3	1.17	2.26	4.69E-08	1.59E-06
214596_at	CHRM3	1.17	2.26	2.46E-05	2.37E-04
226248_s_at	KIAA1324	1.17	2.25	5.44E-07	1.09E-05
220507_s_at	UPB1	1.17	2.25	6.27E-03	1.98E-02
210026_s_at	CARD10	1.17	2.25	1.58E-07	4.18E-06
209842_at	SOX10	1.17	2.25	1.28E-11	1.83E-09
227266_s_at	FYB	1.17	2.24	4.06E-07	8.68E-06
209498_at	CEACAM1	1.17	2.24	1.13E-05	1.26E-04
230937_at	LOC285835	1.17	2.24	1.14E-04	8.05E-04
231600_at	CLEC12B	1.17	2.24	4.89E-05	4.08E-04
213888_s_at	TRAF3IP3	1.16	2.24	8.07E-07	1.51E-05
203895_at	PLCB4	1.16	2.24	3.73E-07	8.17E-06
229088_at	ENPP1	1.16	2.24	6.55E-08	2.09E-06
219452_at	DPEP2	1.16	2.23	9.21E-08	2.71E-06
230925_at	APBB1IP	1.16	2.23	4.36E-06	5.89E-05
202957_at	HCLS1	1.16	2.23	4.87E-09	2.55E-07
220358_at	BATF3	1.16	2.23	7.47E-07	1.42E-05
228060_at	SLC35F1	1.16	2.23	2.45E-08	9.48E-07
1564475_s_at	ZBED3-AS1	1.16	2.23	9.75E-05	7.09E-04
206181_at	SLAMF1	1.16	2.23	3.20E-08	1.18E-06
1554360_at	FCHSD2	1.16	2.23	1.13E-05	1.26E-04
223343_at	MS4A7	1.16	2.23	2.36E-05	2.29E-04
228532_at	C1orf162	1.16	2.23	2.73E-09	1.58E-07
205432_at	OVGP1	1.15	2.22	8.21E-10	5.91E-08
233907_s_at	SERTAD4	1.15	2.22	2.42E-08	9.39E-07

205291_at	IL2RB	1.15	2.22	1.14E-06	1.98E-05
214243_s_at	SERHL /// SERHL2	1.15	2.22	4.94E-04	2.58E-03
226436_at	RASSF4	1.15	2.22	1.23E-09	8.22E-08
232737_s_at	ENPP3	1.15	2.21	9.14E-06	1.06E-04
220560_at	C11orf21	1.14	2.21	2.31E-07	5.64E-06
230025_at	GJD3	1.14	2.21	5.80E-09	2.93E-07
219519_s_at	SIGLEC1	1.14	2.21	1.33E-06	2.26E-05
206204_at	GRB14	1.14	2.21	3.50E-04	1.96E-03
209734_at	NCKAP1L	1.14	2.21	9.90E-11	1.02E-08
217087_at	C1orf68	1.14	2.21	1.54E-03	6.35E-03
227677_at	JAK3	1.14	2.2	2.07E-06	3.23E-05
203892_at	WFDC2	1.14	2.2	2.19E-08	8.64E-07
214053_at	ERBB4	1.14	2.2	1.25E-05	1.37E-04
208161_s_at	ABCC3	1.14	2.2	5.86E-08	1.92E-06
211991_s_at	HLA-DPA1	1.13	2.2	3.21E-09	1.80E-07
235056_at	ETV6	1.13	2.2	3.24E-13	8.02E-11
221916_at	NEFL	1.13	2.19	7.54E-06	9.08E-05
206247_at	MICB	1.13	2.19	7.21E-07	1.38E-05
235486_at	KIAA1549L	1.13	2.19	3.39E-06	4.82E-05
202953_at	C1QB	1.13	2.19	9.62E-12	1.43E-09
208396_s_at	PDE1A	1.13	2.19	1.03E-08	4.63E-07
1294_at	MIR5193 /// UBA7	1.13	2.19	1.22E-10	1.20E-08
204919_at	PRH1-PRR4 /// PRR4	1.13	2.19	1.12E-04	7.94E-04
208885_at	LCP1	1.13	2.19	1.23E-08	5.29E-07
232581_x_at	HIVEP3	1.13	2.19	9.19E-06	1.07E-04
210607_at	FLT3LG	1.13	2.18	3.63E-09	2.01E-07
209975_at	CYP2E1	1.13	2.18	1.76E-07	4.54E-06
1555854_at	AKR1C1 /// AKR1C2 /// LOC101060798	1.13	2.18	4.72E-05	3.97E-04
205786_s_at	ITGAM	1.13	2.18	9.86E-07	1.77E-05
219045_at	RHOF	1.13	2.18	2.99E-10	2.59E-08
228057_at	DDIT4L	1.13	2.18	3.11E-07	7.13E-06
229634_at	TMEM139	1.13	2.18	1.55E-06	2.55E-05
203498_at	RCAN2	1.12	2.18	2.99E-10	2.59E-08
205768_s_at	SLC27A2	1.12	2.18	5.39E-05	4.40E-04
228836_at	SLC25A35	1.12	2.18	1.71E-05	1.76E-04
1556136_at	MYLK4	1.12	2.18	1.44E-06	2.41E-05
232027_at	SYNE1	1.12	2.17	1.61E-07	4.25E-06
239427_at	SLAMF1	1.12	2.17	5.62E-07	1.11E-05
204122_at	TYROBP	1.12	2.17	3.23E-09	1.81E-07
223691_at	RGS22	1.12	2.17	1.55E-06	2.55E-05
204912_at	IL10RA	1.12	2.17	6.81E-10	5.11E-08
235708_at	KLB	1.12	2.17	3.04E-04	1.76E-03
229797_at	MCOLN3	1.12	2.17	4.64E-07	9.64E-06

239272_at	MMP28	1.12	2.17	6.32E-07	1.23E-05
214079_at	DHRS2	1.11	2.16	6.22E-03	1.96E-02
210184_at	ITGAX	1.11	2.16	1.34E-06	2.29E-05
206978_at	CCR2	1.11	2.16	2.22E-06	3.43E-05
231015_at	KLF15	1.11	2.16	1.97E-07	4.98E-06
212873_at	HMHA1	1.11	2.16	4.12E-08	1.42E-06
205419_at	GPR183	1.11	2.16	3.74E-07	8.19E-06
229015_at	LOC286367	1.11	2.16	4.30E-06	5.82E-05
205098_at	CCR1	1.11	2.16	8.44E-09	3.95E-07
229390_at	FAM26F	1.11	2.16	2.51E-05	2.40E-04
228573_at	ANTXR2	1.11	2.16	1.96E-06	3.08E-05
1554406_a_at	CLEC7A	1.11	2.15	5.79E-06	7.36E-05
210108_at	CACNA1D	1.11	2.15	5.01E-08	1.68E-06
243601_at	LOC285957	1.11	2.15	8.55E-10	6.10E-08
1557558_s_at	MATN1-AS1	1.1	2.15	4.12E-09	2.23E-07
205295_at	CKMT2	1.1	2.15	2.63E-07	6.24E-06
214434_at	HSPA12A	1.1	2.15	3.01E-09	1.73E-07
226878_at	HLA-DOA	1.1	2.15	4.50E-08	1.53E-06
206655_s_at	GP1BB /// SEPT5 /// SEPT5-GP1BB	1.1	2.15	2.33E-06	3.56E-05
201743_at	CD14	1.1	2.15	4.06E-11	4.76E-09
212592_at	IGJ	1.1	2.14	1.77E-02	4.53E-02
220783_at	MMP27	1.1	2.14	4.01E-07	8.60E-06
219999_at	MAN2A2	1.1	2.14	3.25E-12	5.77E-10
240409_at	TPTE2P5	1.1	2.14	2.17E-07	5.40E-06
213369_at	CDHR1	1.1	2.14	6.93E-04	3.37E-03
219319_at	HIF3A	1.1	2.14	3.10E-06	4.47E-05
205059_s_at	IDUA	1.09	2.13	3.36E-08	1.22E-06
238423_at	SYTL3	1.09	2.13	3.16E-07	7.22E-06
1561394_s_at	KIAA1755	1.09	2.13	3.18E-07	7.23E-06
235343_at	VASH2	1.09	2.13	5.60E-08	1.85E-06
219716_at	APOL6	1.09	2.13	5.52E-11	6.07E-09
228342_s_at	ALPK3	1.09	2.13	2.80E-04	1.65E-03
210665_at	TFPI	1.09	2.13	1.88E-08	7.53E-07
238039_at	LOC728769	1.09	2.12	3.90E-10	3.19E-08
229534_at	ACOT4	1.09	2.12	2.40E-06	3.64E-05
223741_s_at	TTYH2	1.08	2.12	6.25E-09	3.10E-07
244598_at	LCP2	1.08	2.12	5.94E-08	1.94E-06
226142_at	GLIPR1	1.08	2.12	7.64E-08	2.36E-06
235175_at	GBP4	1.08	2.12	6.74E-07	1.30E-05
204197_s_at	RUNX3	1.08	2.12	3.06E-11	3.78E-09
226603_at	SAMD9L	1.08	2.12	4.19E-08	1.44E-06
41577_at	PPP1R16B	1.08	2.11	5.10E-08	1.70E-06
205073_at	CYP2J2	1.08	2.11	2.20E-07	5.47E-06

217621_at	SLC6A2	1.08	2.11	7.43E-06	8.96E-05
228367_at	ALPK2	1.08	2.11	8.82E-08	2.62E-06
235533_at	COX19	1.08	2.11	2.28E-06	3.50E-05
237291_at	PRORSD1P	1.08	2.11	1.61E-06	2.63E-05
229354_at	AHRR	1.08	2.11	6.15E-07	1.20E-05
236261_at	OSBPL6	1.08	2.11	9.99E-08	2.89E-06
204914_s_at	SOX11	1.08	2.11	5.48E-05	4.46E-04
205599_at	TRAF1	1.08	2.11	1.31E-09	8.64E-08
217478_s_at	HLA-DMA	1.08	2.11	2.14E-11	2.84E-09
1553835_a_at	COL6A5	1.08	2.11	6.27E-03	1.98E-02
203485_at	RTN1	1.07	2.11	1.63E-07	4.29E-06
205992_s_at	IL15	1.07	2.11	2.02E-09	1.24E-07
219616_at	ACSS3	1.07	2.1	2.41E-07	5.85E-06
211138_s_at	KMO	1.07	2.1	1.50E-09	9.61E-08
1553062_at	MOGAT1	1.07	2.1	2.76E-04	1.63E-03
210619_s_at	HYAL1	1.07	2.1	2.14E-04	1.33E-03
215133_s_at	FAM153A /// FAM153B /// LOC100507387 /// LOC100507427	1.07	2.1	2.41E-04	1.46E-03
238575_at	OSBPL6	1.07	2.1	4.16E-06	5.66E-05
210432_s_at	SCN3A	1.07	2.1	1.45E-05	1.53E-04
212970_at	APBB2	1.07	2.1	9.18E-09	4.23E-07
242450_at	RGMB	1.07	2.09	3.26E-07	7.35E-06
207522_s_at	ATP2A3	1.07	2.09	8.90E-05	6.60E-04
1553834_at	COL6A5	1.06	2.09	5.57E-03	1.80E-02
1554248_at	ZNF638	1.06	2.09	1.19E-06	2.07E-05
226474_at	NLRC5	1.06	2.09	8.28E-10	5.95E-08
243526_at	WDR86	1.06	2.09	7.03E-05	5.45E-04
228752_at	EFCAB4B	1.06	2.09	6.63E-04	3.25E-03
229619_at	FOLR2	1.06	2.08	2.74E-07	6.47E-06
209901_x_at	AIF1	1.06	2.08	6.23E-09	3.10E-07
236918_s_at	LRRC34	1.06	2.08	3.16E-06	4.54E-05
210644_s_at	LAIR1	1.06	2.08	1.55E-04	1.03E-03
207302_at	SGCG	1.06	2.08	2.28E-05	2.22E-04
230888_at	WDR91	1.06	2.08	7.19E-09	3.49E-07
207651_at	GPR171	1.06	2.08	1.09E-04	7.77E-04
214889_at	FAM149A	1.06	2.08	2.47E-07	5.95E-06
206623_at	PDE6A	1.06	2.08	8.02E-04	3.78E-03
241739_at	OGFOD1	1.06	2.08	2.14E-05	2.11E-04
219183_s_at	CYTH4	1.06	2.08	8.39E-08	2.54E-06
209827_s_at	IL16	1.06	2.08	2.69E-09	1.57E-07
204352_at	TRAF5	1.06	2.08	8.79E-10	6.22E-08
204501_at	NOV	1.06	2.08	8.27E-09	3.89E-07
205896_at	SLC22A4	1.06	2.08	9.35E-05	6.86E-04

204153_s_at	MFNG	1.06	2.08	4.16E-09	2.24E-07
203708_at	PDE4B	1.05	2.08	1.95E-07	4.95E-06
213752_at	KAZN	1.05	2.07	6.39E-12	1.01E-09
206030_at	ASPA	1.05	2.07	1.55E-07	4.12E-06
205101_at	CIITA	1.05	2.07	5.31E-08	1.76E-06
1559950_at	FAM66C /// FAM66D	1.05	2.07	2.04E-04	1.28E-03
229820_at	LINC00969	1.05	2.07	4.86E-04	2.55E-03
210889_s_at	FCGR2B	1.05	2.07	9.96E-09	4.49E-07
206186_at	MPP3	1.05	2.07	3.09E-07	7.09E-06
224451_x_at	ARHGAP9	1.05	2.07	1.49E-06	2.47E-05
208733_at	RAB2A	1.05	2.07	9.74E-05	7.08E-04
223605_at	SLC25A18	1.05	2.07	5.15E-04	2.66E-03
207100_s_at	VAMP1	1.05	2.07	1.17E-07	3.31E-06
215116_s_at	DNM1	1.05	2.07	3.87E-09	2.12E-07
205337_at	DCT	1.05	2.07	1.97E-06	3.10E-05
1555890_at	OR2A20P /// OR2A9P	1.05	2.07	2.65E-06	3.96E-05
230563_at	RASGEF1A	1.05	2.07	2.50E-05	2.40E-04
1562573_at	CYP17A1	1.05	2.06	4.72E-07	9.75E-06
228258_at	TBC1D10C	1.05	2.06	3.59E-09	2.00E-07
1564786_at	LOC338667	1.04	2.06	6.61E-03	2.06E-02
212613_at	BTN3A2	1.04	2.06	1.89E-06	3.00E-05
209905_at	HOXA10-HOXA9 /// HOXA9 /// MIR196B	1.04	2.06	5.88E-06	7.45E-05
205299_s_at	BTN2A2	1.04	2.06	2.61E-07	6.23E-06
212444_at	GPRC5A	1.04	2.06	1.04E-05	1.18E-04
216701_at	C1orf68	1.04	2.06	1.56E-03	6.44E-03
227201_at	LOC643837	1.04	2.06	2.62E-07	6.23E-06
205843_x_at	CRAT	1.04	2.06	9.05E-04	4.17E-03
235418_at	FAHD2A	1.04	2.06	1.98E-08	7.89E-07
205213_at	ACAP1	1.04	2.05	1.06E-08	4.71E-07
235574_at	GBP4	1.04	2.05	5.88E-08	1.92E-06
213845_at	GRIK2	1.04	2.05	2.17E-06	3.35E-05
232617_at	CTSS	1.04	2.05	9.83E-09	4.45E-07
229430_at	C8orf46	1.04	2.05	2.35E-06	3.59E-05
1552398_a_at	CLEC12A	1.04	2.05	4.12E-05	3.56E-04
242940_x_at	DLX6	1.04	2.05	4.33E-07	9.09E-06
220801_s_at	HAO2	1.04	2.05	3.76E-03	1.31E-02
206214_at	PLA2G7	1.04	2.05	4.87E-05	4.07E-04
207623_at	ABCF2	1.03	2.05	6.86E-09	3.35E-07
223946_at	MED23	1.03	2.05	1.93E-07	4.89E-06
210439_at	ICOS	1.03	2.05	3.19E-05	2.89E-04
222317_at	PDE3B	1.03	2.04	8.88E-03	2.61E-02
207072_at	IL18RAP	1.03	2.04	8.04E-06	9.57E-05
209488_s_at	RBPMS	1.03	2.04	6.27E-06	7.81E-05

205455_at	MST1R	1.03	2.04	3.00E-06	4.36E-05
236150_at	HYKK	1.03	2.04	1.66E-07	4.36E-06
211734_s_at	FCER1A	1.03	2.04	6.30E-09	3.12E-07
204760_s_at	NR1D1 /// THRA	1.03	2.04	8.34E-05	6.27E-04
236064_at	SLC25A35	1.03	2.04	1.63E-05	1.69E-04
31637_s_at	NR1D1 /// THRA	1.03	2.04	1.92E-05	1.94E-04
219992_at	TAC3	1.03	2.04	2.36E-04	1.44E-03
1555756_a_at	CLEC7A	1.03	2.04	3.93E-04	2.15E-03
205769_at	SLC27A2	1.02	2.03	3.15E-04	1.81E-03
211062_s_at	CPZ /// GPR78	1.02	2.03	6.29E-11	6.82E-09
227168_at	MIAT	1.02	2.03	1.12E-06	1.96E-05
229497_at	ANKDD1A	1.02	2.03	4.32E-10	3.49E-08
1554057_at	ASH1L-AS1	1.02	2.03	7.04E-08	2.23E-06
229241_at	LDHD	1.02	2.03	4.30E-04	2.31E-03
240183_at	TMEM213	1.02	2.03	2.62E-03	9.79E-03
1568638_a_at	IDO2	1.02	2.03	2.74E-09	1.59E-07
230220_at	UNC80	1.02	2.03	1.26E-07	3.52E-06
209994_s_at	ABCB1 /// ABCB4	1.02	2.03	2.70E-09	1.57E-07
241440_at	ZIC1	1.02	2.02	1.29E-04	8.92E-04
207561_s_at	ABCB8 /// ASIC3	1.02	2.02	4.04E-06	5.53E-05
204259_at	MMP7	1.02	2.02	6.28E-06	7.81E-05
1558770_a_at	PIK3R6	1.02	2.02	2.60E-06	3.90E-05
223562_at	PARVG	1.02	2.02	9.00E-07	1.64E-05
235719_at	CYP4V2	1.02	2.02	2.85E-05	2.65E-04
229902_at	FLT4	1.02	2.02	3.05E-06	4.42E-05
227803_at	ENPP5	1.01	2.02	8.36E-06	9.88E-05
211909_x_at	PTGER3	1.01	2.02	6.23E-07	1.22E-05
204179_at	MB	1.01	2.02	2.18E-06	3.37E-05
214828_s_at	RRP7A /// RRP7B	1.01	2.02	3.33E-04	1.89E-03
1560011_at	JRK	1.01	2.02	4.34E-08	1.49E-06
229597_s_at	WDFY4	1.01	2.02	6.63E-07	1.28E-05
204519_s_at	PLLP	1.01	2.02	5.75E-05	4.62E-04
204939_s_at	PLN	1.01	2.02	6.20E-03	1.96E-02
1553772_at	GK5	1.01	2.02	9.49E-08	2.77E-06
236360_at	FLJ42875	1.01	2.01	9.01E-07	1.64E-05
211685_s_at	NCALD	1.01	2.01	5.98E-05	4.77E-04
229092_at	NR2F2	1.01	2.01	8.80E-08	2.62E-06
223121_s_at	SFRP2	1.01	2.01	2.39E-04	1.46E-03
230417_at	GALNT16	1.01	2.01	4.60E-08	1.56E-06
209883_at	COLGALT2	1.01	2.01	3.25E-07	7.35E-06
225491_at	SLC1A2	1.01	2.01	3.68E-06	5.12E-05
1553890_s_at	NTN5	1.01	2.01	1.83E-05	1.86E-04
238581_at	GBP5	1.01	2.01	3.33E-05	2.99E-04

227609_at	EPSTI1	1.01	2.01	1.44E-04	9.74E-04
201506_at	TGFBI	1.01	2.01	2.24E-10	2.01E-08
205671_s_at	HLA-DOB	1	2	1.18E-04	8.29E-04
238018_at	FAM150B	1	2	1.10E-05	1.23E-04
208146_s_at	CPVL	1	2	8.83E-08	2.62E-06
219562_at	RAB26	1	2	4.85E-08	1.63E-06
238114_at	PCMTD1	0.998	2	3.17E-09	1.79E-07
202531_at	IRF1	0.998	2	1.43E-07	3.89E-06
204852_s_at	PTPN7	0.997	2	3.76E-08	1.33E-06
226627_at	8-Sep	0.995	1.99	1.67E-08	6.83E-07
230169_at	THAP6	0.994	1.99	8.99E-12	1.37E-09
236539_at	PTPN22	0.993	1.99	3.09E-07	7.09E-06
219959_at	MOCOS	0.993	1.99	2.29E-03	8.76E-03
1556427_s_at	LRRN4CL	0.993	1.99	4.61E-05	3.89E-04
223534_s_at	RPS6KL1	0.993	1.99	3.38E-08	1.22E-06
239593_at	TMEM213	0.993	1.99	2.86E-03	1.05E-02
1559952_x_at	FAM66C /// FAM66D	0.993	1.99	3.05E-04	1.76E-03
217557_s_at	CPM	0.993	1.99	8.55E-08	2.57E-06
206601_s_at	HOXD3	0.991	1.99	4.17E-07	8.86E-06
213095_x_at	AIF1	0.99	1.99	3.63E-09	2.01E-07
223950_s_at	FLYWCH1	0.989	1.99	4.05E-05	3.51E-04
1559050_at	HCG27 /// LOC100996357	0.988	1.98	1.18E-08	5.16E-07
225598_at	SLC45A4	0.988	1.98	1.50E-07	4.04E-06
242988_at	DCST2	0.988	1.98	6.73E-08	2.14E-06
210163_at	CXCL11	0.986	1.98	2.36E-04	1.44E-03
238545_at	BRD7	0.985	1.98	3.36E-06	4.78E-05
236480_at	MIR210HG	0.985	1.98	2.94E-07	6.84E-06
228780_at	POU3F3	0.984	1.98	1.02E-07	2.95E-06
232428_at	MOGAT2	0.984	1.98	2.31E-03	8.84E-03
220559_at	EN1	0.984	1.98	1.31E-08	5.56E-07
244033_at	CEP128	0.984	1.98	1.31E-06	2.23E-05
205306_x_at	KMO	0.983	1.98	5.73E-07	1.13E-05
206707_x_at	FAM65B	0.983	1.98	5.80E-09	2.93E-07
228182_at	ADCY5	0.983	1.98	1.39E-05	1.48E-04
230974_at	DDX19B	0.982	1.98	1.30E-08	5.54E-07

222938_x_at	ENPP3	0.982	1.98	1.78E-07	4.59E-06
213822_s_at	UBE3B	0.982	1.98	3.82E-05	3.34E-04
226439_s_at	NBEA	0.981	1.97	1.05E-06	1.86E-05
1556096_s_at	UNC13C	0.981	1.97	6.61E-04	3.25E-03
234787_at	DKFZp564H213	0.98	1.97	1.96E-06	3.09E-05
202510_s_at	TNFAIP2	0.98	1.97	1.54E-10	1.46E-08
228888_at	STAC2	0.979	1.97	4.98E-05	4.14E-04
234722_x_at	OBP2B	0.979	1.97	2.85E-05	2.65E-04
236583_at	GIMAP1	0.979	1.97	8.80E-07	1.61E-05
231384_at	GRIN2A	0.978	1.97	4.07E-05	3.52E-04
212187_x_at	PTGDS	0.977	1.97	1.79E-08	7.23E-07
228391_at	CYP4V2	0.977	1.97	5.64E-06	7.20E-05
231323_at	PSMB2	0.977	1.97	4.10E-06	5.61E-05
238877_at	EYA4	0.977	1.97	2.07E-04	1.30E-03
236886_at	LOC100049716	0.976	1.97	1.31E-05	1.41E-04
231549_at	C1orf158	0.976	1.97	1.09E-02	3.08E-02
236995_x_at	TFEC	0.976	1.97	5.16E-07	1.04E-05
1561226_at	XCR1	0.975	1.97	7.01E-06	8.55E-05
237870_at	NQO2	0.975	1.97	2.01E-04	1.27E-03
226818_at	MPEG1	0.975	1.97	8.48E-08	2.56E-06
220276_at	RERGL	0.973	1.96	1.75E-03	7.07E-03
230036_at	SAMD9L	0.973	1.96	2.78E-07	6.53E-06
228111_s_at	DNAH1	0.973	1.96	5.02E-07	1.02E-05
204114_at	NID2	0.972	1.96	3.88E-06	5.36E-05
203673_at	TG	0.971	1.96	2.89E-05	2.67E-04
213217_at	ADCY2	0.971	1.96	3.23E-07	7.31E-06
219211_at	USP18	0.971	1.96	2.44E-07	5.90E-06
211005_at	LAT	0.969	1.96	7.14E-08	2.25E-06
230405_at	C5orf56	0.968	1.96	2.45E-09	1.45E-07
1570020_at	AACSP1	0.968	1.96	1.57E-05	1.64E-04
207777_s_at	SP140	0.967	1.96	3.56E-08	1.27E-06
1556063_s_at	RPP30	0.967	1.95	4.00E-09	2.18E-07
239380_at	C5orf27	0.967	1.95	2.67E-05	2.52E-04
219574_at	1-Mar	0.966	1.95	1.73E-08	7.02E-07

230453_s_at	ATP2A3	0.966	1.95	1.45E-04	9.79E-04
205066_s_at	ENPP1	0.966	1.95	7.31E-07	1.39E-05
242286_at	GRIN2A	0.965	1.95	2.10E-07	5.25E-06
59697_at	RAB15	0.964	1.95	1.06E-07	3.01E-06
228160_at	LINC00667	0.964	1.95	4.22E-08	1.45E-06
205997_at	ADAM28	0.963	1.95	5.10E-08	1.70E-06
202307_s_at	TAP1	0.963	1.95	5.45E-12	8.91E-10
223280_x_at	MS4A6A	0.962	1.95	3.06E-07	7.06E-06
205433_at	BCHE	0.962	1.95	1.55E-07	4.12E-06
205221_at	HGD	0.96	1.95	2.24E-03	8.62E-03
204301_at	KBTBD11	0.96	1.95	1.22E-08	5.28E-07
228494_at	PPP1R9A	0.959	1.94	3.65E-04	2.03E-03
205691_at	SYNGR3	0.959	1.94	1.28E-06	2.19E-05
240509_s_at	GREM2	0.957	1.94	5.61E-05	4.54E-04
242414_at	QPRT	0.957	1.94	5.49E-09	2.83E-07
220646_s_at	KLRF1	0.956	1.94	8.55E-06	1.00E-04
231773_at	ANGPTL1	0.956	1.94	5.55E-07	1.10E-05
227048_at	LAMA1	0.955	1.94	7.20E-05	5.55E-04
214746_s_at	ZNF467	0.954	1.94	4.34E-05	3.70E-04
206726_at	HPGDS	0.954	1.94	3.20E-04	1.83E-03
1555728_a_at	MS4A4A	0.954	1.94	3.17E-09	1.79E-07
207955_at	CCL27	0.953	1.94	4.39E-04	2.35E-03
206199_at	CEACAM7	0.952	1.93	1.62E-03	6.64E-03
225285_at	BCAT1	0.951	1.93	1.01E-05	1.15E-04
210140_at	CST7	0.951	1.93	2.52E-04	1.52E-03
235050_at	SLC2A12	0.95	1.93	2.14E-06	3.32E-05
211012_s_at	PML	0.95	1.93	2.05E-09	1.25E-07
235742_at	RHOC	0.949	1.93	3.84E-08	1.35E-06
213603_s_at	RAC2	0.949	1.93	4.49E-08	1.53E-06
212587_s_at	PTPRC	0.949	1.93	1.26E-05	1.37E-04
227196_at	RHPN2	0.949	1.93	3.10E-04	1.79E-03
227867_at	TRABD2A	0.948	1.93	6.07E-06	7.64E-05
231093_at	FCRL3	0.947	1.93	2.45E-04	1.48E-03
218322_s_at	ACSL5	0.947	1.93	3.31E-07	7.43E-06

1552634_a_at	ZNF101	0.947	1.93	1.95E-06	3.07E-05
207376_at	VENTX	0.946	1.93	1.72E-07	4.46E-06
204499_at	AGTPBP1	0.945	1.93	2.31E-08	9.04E-07
229002_at	FAM69B	0.945	1.93	4.04E-04	2.20E-03
239349_at	C1QTNF7	0.944	1.92	1.66E-04	1.09E-03
1568794_at	LOC643355	0.944	1.92	3.04E-04	1.76E-03
206060_s_at	PTPN22	0.943	1.92	7.42E-07	1.41E-05
229866_at	STK32A	0.942	1.92	2.70E-04	1.60E-03
236253_at	ZNF546	0.941	1.92	9.05E-07	1.65E-05
230143_at	RNF165	0.94	1.92	2.41E-07	5.85E-06
219056_at	RNASEH2B	0.94	1.92	3.66E-08	1.30E-06
211748_x_at	PTGDS	0.939	1.92	3.75E-10	3.13E-08
226702_at	CMPK2	0.939	1.92	3.24E-07	7.32E-06
214087_s_at	MYBPC1	0.939	1.92	7.10E-04	3.43E-03
232195_at	GPR158	0.939	1.92	9.17E-05	6.76E-04
236436_at	SLC25A45	0.939	1.92	2.61E-09	1.53E-07
239183_at	ANGPTL1	0.938	1.92	7.53E-08	2.33E-06
228821_at	ST6GAL2	0.937	1.91	9.92E-05	7.19E-04
219054_at	NPR3	0.937	1.91	5.62E-06	7.18E-05
1553311_at	C20orf197	0.936	1.91	2.56E-06	3.84E-05
203828_s_at	IL32	0.936	1.91	6.36E-06	7.89E-05
203104_at	CSF1R	0.936	1.91	2.69E-09	1.57E-07
1554906_a_at	MPHOSPH6	0.936	1.91	3.36E-04	1.90E-03
208450_at	LGALS2	0.935	1.91	4.68E-04	2.48E-03
1558934_a_at	GTF2H5	0.935	1.91	3.02E-06	4.38E-05
203281_s_at	MIR5193 /// UBA7	0.934	1.91	8.04E-09	3.82E-07
219213_at	JAM2	0.934	1.91	1.71E-07	4.45E-06
220428_at	CD207	0.933	1.91	3.27E-06	4.66E-05
206539_s_at	CYP4F12	0.933	1.91	3.15E-05	2.86E-04
208189_s_at	MYO7A	0.933	1.91	1.04E-07	2.98E-06
236331_at	CDKL2	0.932	1.91	1.52E-03	6.31E-03
1555786_s_at	LINC00520	0.932	1.91	7.68E-03	2.33E-02
226553_at	TMPRSS2	0.932	1.91	7.88E-04	3.73E-03
203402_at	KCNAB2	0.932	1.91	6.79E-10	5.11E-08

232234_at	SLA2	0.932	1.91	5.51E-07	1.10E-05
205403_at	IL1R2	0.931	1.91	4.61E-09	2.43E-07
1559590_at	CHDH	0.931	1.91	9.21E-10	6.43E-08
226612_at	UBE2QL1	0.93	1.91	4.00E-07	8.60E-06
230873_at	TTLL3	0.93	1.9	1.72E-04	1.12E-03
239273_s_at	MMP28	0.929	1.9	3.92E-06	5.40E-05
239785_at	DZIP1L	0.928	1.9	1.84E-09	1.15E-07
228847_at	EXOC3	0.927	1.9	3.05E-07	7.04E-06
229222_at	ACSS3	0.927	1.9	1.49E-06	2.48E-05
1554122_a_at	HSD17B12	0.926	1.9	3.04E-03	1.10E-02
211742_s_at	EVI2B	0.926	1.9	4.17E-07	8.86E-06
1556300_s_at	SIM1	0.925	1.9	7.60E-07	1.43E-05
1564276_at	C5orf56	0.925	1.9	8.03E-07	1.50E-05
208195_at	TTN	0.925	1.9	9.00E-07	1.64E-05
236154_at	QKI	0.923	1.9	8.43E-07	1.56E-05
214012_at	ERAP1	0.923	1.9	8.81E-04	4.08E-03
204220_at	GMFG	0.923	1.9	3.38E-08	1.22E-06
229052_at	ANKRD23 /// ANKRD39	0.923	1.9	1.10E-06	1.93E-05
218066_at	SLC12A7	0.923	1.9	6.58E-08	2.10E-06
208121_s_at	PTPRO	0.922	1.89	1.67E-07	4.37E-06
230847_at	WRNIP1	0.921	1.89	2.49E-06	3.75E-05
213160_at	DOCK2	0.921	1.89	4.41E-08	1.51E-06
244363_at	ROS1	0.921	1.89	2.59E-03	9.67E-03
206026_s_at	TNFAIP6	0.921	1.89	1.50E-05	1.58E-04
204130_at	HSD11B2	0.921	1.89	3.61E-07	7.94E-06
230616_at	LAMB2P1	0.92	1.89	2.47E-08	9.53E-07
243027_at	IGSF5	0.92	1.89	1.09E-05	1.23E-04
226955_at	AFAP1L1	0.92	1.89	5.88E-06	7.45E-05
205593_s_at	PDE9A	0.92	1.89	5.81E-05	4.66E-04
1568696_at	CDNF	0.918	1.89	5.58E-06	7.13E-05
205225_at	ESR1	0.918	1.89	1.63E-07	4.30E-06
209976_s_at	CYP2E1	0.917	1.89	9.60E-07	1.73E-05
223655_at	CD163L1	0.917	1.89	2.48E-10	2.20E-08
210215_at	TFR2	0.917	1.89	5.85E-08	1.92E-06
213418_at	HSPA6	0.917	1.89	6.15E-06	7.71E-05

230962_at	DCLK1	0.917	1.89	8.96E-05	6.64E-04
210253_at	HTATIP2	0.917	1.89	1.73E-06	2.78E-05
234994_at	TMEM200A	0.916	1.89	3.25E-05	2.93E-04
1553454_at	RPTN	0.915	1.89	5.28E-05	4.34E-04
202357_s_at	CFB	0.915	1.89	1.54E-07	4.12E-06
204774_at	EVI2A	0.915	1.89	1.00E-05	1.15E-04
213309_at	PLCL2	0.915	1.89	3.42E-07	7.61E-06
230941_at	LOC728537	0.915	1.89	1.53E-05	1.61E-04
226611_s_at	CENPV	0.915	1.89	4.85E-07	9.99E-06
210538_s_at	BIRC3	0.915	1.89	4.84E-04	2.54E-03
207067_s_at	HDC	0.915	1.89	2.63E-04	1.57E-03
214186_s_at	HCG26	0.915	1.89	1.95E-06	3.07E-05
205859_at	LY86	0.914	1.88	1.90E-05	1.92E-04
231195_at	KLRG2	0.914	1.88	3.50E-05	3.11E-04
205660_at	OASL	0.914	1.88	1.23E-06	2.13E-05
228724_at	TTLL7	0.914	1.88	1.92E-05	1.93E-04
227174_at	WDR72	0.914	1.88	2.50E-03	9.39E-03
211740_at	ICA1	0.913	1.88	2.27E-03	8.70E-03
232300_at	ADIRF-AS1	0.913	1.88	3.26E-05	2.94E-04
204575_s_at	MMP19	0.911	1.88	1.83E-07	4.69E-06
1552316_a_at	GIMAP1	0.911	1.88	5.38E-05	4.39E-04
232083_at	KIF16B	0.909	1.88	1.75E-04	1.14E-03
205619_s_at	MEOX1	0.909	1.88	8.84E-08	2.63E-06
219531_at	CEP72	0.909	1.88	3.22E-05	2.91E-04
230636_s_at	KLF9	0.908	1.88	9.69E-08	2.82E-06
231930_at	ELMOD1 /// LOC643923	0.908	1.88	4.43E-04	2.37E-03
242392_at	UBE2U	0.907	1.88	6.79E-03	2.10E-02
226500_at	ZBTB47	0.906	1.87	3.02E-10	2.60E-08
206552_s_at	TAC1	0.906	1.87	6.14E-04	3.07E-03
229631_at	DNHD1	0.905	1.87	8.40E-07	1.55E-05
215967_s_at	LY9	0.905	1.87	7.18E-08	2.26E-06
236950_s_at	LINC00964	0.905	1.87	1.44E-05	1.52E-04
204829_s_at	FOLR2	0.905	1.87	1.92E-06	3.03E-05

89977_at	ACSM5	0.905	1.87	8.49E-05	6.36E-04
219610_at	ARHGEF28	0.904	1.87	1.50E-09	9.61E-08
239846_at	MTHFD1	0.904	1.87	1.02E-06	1.81E-05
236565_s_at	LARP6	0.903	1.87	3.47E-04	1.94E-03
1556839_s_at	LOC100289090	0.903	1.87	1.13E-03	4.98E-03
244385_at	KDM4C	0.903	1.87	2.00E-05	2.00E-04
219594_at	NINJ2	0.902	1.87	3.80E-07	8.28E-06
215051_x_at	AIF1	0.902	1.87	7.33E-09	3.54E-07
1555865_at	TOLLIP-AS1	0.902	1.87	1.65E-06	2.69E-05
229019_at	ZNF385B	0.901	1.87	3.57E-04	1.99E-03
221087_s_at	APOL3	0.901	1.87	1.03E-08	4.61E-07
1560697_at	TRHDE-AS1	0.901	1.87	1.64E-04	1.08E-03
207547_s_at	FAM107A /// LOC101059937	0.9	1.87	2.55E-07	6.10E-06
213489_at	MAPRE2	0.9	1.87	3.88E-08	1.36E-06
214945_at	FAM153A /// FAM153B /// FAM153C /// LOC100507387	0.899	1.86	9.89E-03	2.85E-02
242656_at	GTF2H1	0.899	1.86	4.20E-06	5.71E-05
226811_at	FAM46C	0.898	1.86	1.29E-05	1.40E-04
229537_at	LMO4	0.898	1.86	5.43E-07	1.09E-05
228150_at	SEC16B	0.898	1.86	1.71E-08	6.94E-07
230277_at	LOC100289187	0.897	1.86	6.93E-08	2.20E-06
209685_s_at	PRKCB	0.897	1.86	3.30E-08	1.20E-06
215446_s_at	LOX	0.897	1.86	5.09E-07	1.03E-05
239007_at	ZNF616	0.896	1.86	9.78E-06	1.12E-04
204717_s_at	SLC29A2	0.896	1.86	1.55E-07	4.12E-06
208018_s_at	HCK	0.896	1.86	1.36E-08	5.70E-07
232543_x_at	ARHGAP9	0.896	1.86	2.61E-07	6.22E-06
206856_at	LILRB5	0.896	1.86	9.34E-06	1.08E-04
213326_at	VAMP1	0.895	1.86	4.18E-07	8.87E-06
205518_s_at	CMAHP	0.895	1.86	9.64E-06	1.11E-04
224356_x_at	MS4A6A	0.895	1.86	1.62E-06	2.65E-05
225701_at	AKNA	0.895	1.86	3.01E-08	1.13E-06
230766_at	GART	0.895	1.86	2.37E-06	3.61E-05
213733_at	MYO1F	0.894	1.86	1.49E-08	6.22E-07
219505_at	CECR1	0.894	1.86	1.87E-04	1.20E-03

229400_at	HOXD10	0.894	1.86	6.08E-05	4.84E-04
243690_at	TRIOBP	0.894	1.86	1.47E-08	6.16E-07
226992_at	NOSTRIN	0.894	1.86	1.26E-04	8.78E-04
208072_s_at	DGKD	0.893	1.86	5.29E-11	5.91E-09
242557_at	ZNRD1-AS1	0.893	1.86	3.54E-03	1.25E-02
214307_at	HGD	0.892	1.86	3.39E-04	1.91E-03
244276_at	KLB	0.891	1.85	1.86E-04	1.19E-03
227412_at	PPP1R3E	0.891	1.85	1.05E-08	4.70E-07
236518_at	KIAA1984	0.891	1.85	7.76E-08	2.39E-06
228132_at	ABLIM2	0.89	1.85	3.19E-08	1.17E-06
211372_s_at	IL1R2	0.89	1.85	2.29E-05	2.23E-04
201037_at	PFKP	0.89	1.85	9.02E-10	6.34E-08
220073_s_at	PLEKHG6	0.89	1.85	2.69E-07	6.38E-06
243606_at	NXPE3	0.89	1.85	1.91E-07	4.87E-06
228218_at	LSAMP	0.89	1.85	2.29E-05	2.23E-04
230360_at	GLDN	0.889	1.85	6.71E-09	3.29E-07
215332_s_at	CD8B /// LOC100996919	0.889	1.85	1.99E-06	3.12E-05
224357_s_at	MS4A4A	0.889	1.85	8.22E-07	1.53E-05
225379_at	MAPT	0.888	1.85	4.61E-08	1.57E-06
1552302_at	TMEM106A	0.888	1.85	2.92E-06	4.27E-05
233880_at	RNF213	0.888	1.85	8.29E-08	2.51E-06
204735_at	PDE4A	0.887	1.85	3.79E-10	3.14E-08
206710_s_at	EPB41L3	0.886	1.85	7.27E-09	3.52E-07
204057_at	IRF8	0.886	1.85	1.19E-04	8.39E-04
210674_s_at	PCDHA1 /// PCDHA10 /// PCDHA11 /// PCDHA12 /// PCDHA13 /// PCDHA2 /// PCDHA3 /// PCDHA4 /// PCDHA5 /// PCDHA6 /// PCDHA7 /// PCDHA8 /// PCDHA9 /// PCDHAC1 /// PCDHAC2	0.885	1.85	3.13E-05	2.85E-04
205860_x_at	FOLH1 /// FOLH1B	0.885	1.85	3.32E-05	2.98E-04
202952_s_at	ADAM12	0.884	1.85	2.36E-05	2.29E-04
211776_s_at	EPB41L3	0.884	1.85	1.80E-10	1.67E-08
230480_at	PIWIL4	0.884	1.84	2.68E-05	2.53E-04
203547_at	CD4	0.884	1.84	7.68E-10	5.57E-08
205609_at	ANGPT1	0.883	1.84	7.18E-06	8.71E-05
1560531_at	LCE1B	0.883	1.84	2.74E-05	2.56E-04
210380_s_at	CACNA1G	0.883	1.84	5.78E-07	1.14E-05
225973_at	TAP2	0.882	1.84	1.73E-07	4.48E-06

204940_at	PLN	0.881	1.84	3.28E-03	1.17E-02
231003_at	SLC35B3	0.881	1.84	1.79E-06	2.87E-05
219615_s_at	KCNK5	0.88	1.84	4.90E-04	2.56E-03
218864_at	TNS1	0.88	1.84	1.40E-05	1.49E-04
236124_at	LOC153546	0.88	1.84	1.97E-05	1.98E-04
219106_s_at	KLHL41	0.879	1.84	9.90E-07	1.77E-05
219690_at	IGFLR1	0.879	1.84	1.80E-07	4.63E-06
208964_s_at	FADS1 /// MIR1908	0.879	1.84	1.63E-02	4.25E-02
235780_at	PRKACB	0.878	1.84	3.09E-06	4.46E-05
230673_at	PKHD1L1	0.878	1.84	4.70E-07	9.72E-06
223816_at	SLC46A2	0.878	1.84	6.66E-05	5.21E-04
230488_s_at	DBH-AS1	0.878	1.84	1.13E-07	3.21E-06
204563_at	SELL	0.877	1.84	3.46E-04	1.94E-03
231261_at	CATSPERG	0.876	1.84	9.93E-08	2.87E-06
240430_at	KCMF1	0.876	1.84	4.23E-08	1.45E-06
228845_at	PLBD2	0.876	1.83	9.94E-12	1.47E-09
236055_at	DQX1	0.876	1.83	3.94E-05	3.44E-04
218345_at	TMEM176A	0.875	1.83	4.16E-05	3.59E-04
232593_at	NEURL3	0.875	1.83	2.13E-07	5.31E-06
228167_at	KLHL6	0.875	1.83	4.89E-06	6.44E-05
220595_at	PDZRN4	0.875	1.83	1.94E-04	1.23E-03
218865_at	1-Mar	0.874	1.83	1.25E-03	5.39E-03
206068_s_at	ACADL	0.874	1.83	7.63E-06	9.17E-05
205456_at	CD3E	0.873	1.83	3.45E-06	4.87E-05
235372_at	FCRLA	0.873	1.83	2.20E-07	5.46E-06
209695_at	PTP4A3	0.873	1.83	2.43E-06	3.68E-05
239787_at	KCTD4	0.873	1.83	5.35E-03	1.75E-02
222253_s_at	LOC727983 /// POM121L9P	0.873	1.83	2.46E-03	9.29E-03
204529_s_at	TOX	0.873	1.83	2.38E-05	2.30E-04
217630_at	ANGEL2	0.872	1.83	1.59E-05	1.66E-04
241395_at	NIT1	0.872	1.83	1.10E-06	1.92E-05
228448_at	MAP6	0.871	1.83	5.77E-08	1.90E-06
227877_at	ANXA2R	0.87	1.83	7.73E-09	3.69E-07
203332_s_at	INPP5D	0.87	1.83	2.79E-09	1.61E-07
225165_at	PPP1R1B	0.87	1.83	5.47E-05	4.45E-04

229857_s_at	KANSL1-AS1	0.86<br>9	1.83	7.04E-03	2.17E-02
202982_s_at	ACOT1 /// ACOT2	0.86<br>9	1.83	3.21E-03	1.15E-02
230388_s_at	KANSL1-AS1	0.86<br>8	1.83	3.58E-03	1.26E-02
227563_at	FAM27E3	0.86<br>8	1.82	4.36E-05	3.71E-04
225909_at	LOC728743 /// ZNF775	0.86<br>8	1.82	1.06E-06	1.88E-05
204685_s_at	ATP2B2	0.86<br>8	1.82	4.75E-06	6.29E-05
203932_at	HLA-DMB	0.86<br>7	1.82	1.40E-11	1.96E-09
202086_at	MX1	0.86<br>7	1.82	1.03E-05	1.17E-04
219773_at	NOX4	0.86<br>7	1.82	4.02E-05	3.49E-04
211343_s_at	COL13A1	0.86<br>6	1.82	8.86E-09	4.11E-07
204670_x_at	HLA-DRB1 /// HLA-DRB4 /// LOC100507709 ///<br>LOC100507714	0.86<br>6	1.82	2.98E-07	6.91E-06
213245_at	ADCY1	0.86<br>6	1.82	6.25E-05	4.95E-04
226219_at	ARHGAP30	0.86<br>6	1.82	1.26E-08	5.42E-07
226632_at	CYGB	0.86<br>5	1.82	2.57E-06	3.86E-05
203766_s_at	LMOD1	0.86<br>5	1.82	2.73E-04	1.61E-03
244334_at	TRAM1L1	0.86<br>4	1.82	5.32E-07	1.07E-05
202156_s_at	CELF2	0.86<br>4	1.82	1.76E-07	4.54E-06
204336_s_at	RGS19	0.86<br>4	1.82	2.23E-06	3.44E-05
1562247_at	LOC286058	0.86<br>3	1.82	4.50E-06	6.03E-05
227015_at	ASPHD2	0.86<br>3	1.82	1.93E-06	3.05E-05
1556471_at	SCML4	0.86<br>3	1.82	3.02E-06	4.38E-05
206600_s_at	SLC16A5	0.86<br>2	1.82	3.03E-06	4.39E-05
202938_x_at	RRP7A /// RRP7B	0.86<br>1	1.82	1.66E-03	6.75E-03
231911_at	ERMN	0.86<br>1	1.82	4.92E-08	1.65E-06
206930_at	GLYAT	0.86<br>1	1.82	1.32E-02	3.58E-02
215363_x_at	FOLH1 /// FOLH1B	0.86<br>1	1.82	2.19E-05	2.16E-04
229454_at	BCLAF1	0.86	1.82	3.28E-08	1.20E-06
226841_at	MPEG1	0.86	1.82	7.50E-08	2.33E-06
221272_s_at	C1orf21	0.86	1.81	5.67E-08	1.87E-06
230805_at	MIR142	0.86	1.81	2.06E-08	8.18E-07
218400_at	OAS3	0.85<br>9	1.81	2.48E-06	3.75E-05
244680_at	GLRB	0.85<br>9	1.81	2.31E-05	2.25E-04
204376_at	VPRBP	0.85<br>8	1.81	1.26E-10	1.23E-08
229450_at	IFIT3	0.85<br>8	1.81	5.62E-07	1.12E-05

39763_at	HPX	0.857	1.81	1.19E-05	1.31E-04
235528_at	GUCA1B	0.857	1.81	4.48E-07	9.35E-06
236927_at	SVEP1	0.857	1.81	8.28E-06	9.81E-05
214210_at	SLC25A17	0.857	1.81	2.32E-08	9.06E-07
219471_at	KIAA0226L	0.857	1.81	4.83E-06	6.38E-05
230782_at	SORD	0.856	1.81	8.05E-07	1.50E-05
211106_at	SUPT3H	0.856	1.81	2.52E-04	1.52E-03
209375_at	XPC	0.856	1.81	3.87E-07	8.39E-06
223423_at	GPR160	0.856	1.81	7.08E-06	8.61E-05
230145_at	DUS3L	0.854	1.81	5.68E-06	7.23E-05
218876_at	TPPP3	0.854	1.81	5.06E-04	2.63E-03
241456_at	FAM78B	0.854	1.81	1.08E-05	1.21E-04
242013_at	BCL2L15	0.854	1.81	9.61E-06	1.11E-04
205439_at	GSTT2	0.854	1.81	1.83E-02	4.65E-02
214719_at	SLC46A3	0.854	1.81	8.63E-09	4.01E-07
226622_at	MUC20	0.854	1.81	5.80E-03	1.86E-02
236031_x_at	FREM1	0.853	1.81	3.64E-07	8.00E-06
244110_at	KMT2A	0.853	1.81	5.04E-04	2.61E-03
235122_at	HIVEP3	0.852	1.81	6.67E-05	5.21E-04
36030_at	IFFO1	0.852	1.8	1.41E-07	3.83E-06
214240_at	GAL	0.852	1.8	1.82E-02	4.64E-02
204938_s_at	PLN	0.851	1.8	1.02E-03	4.58E-03
209245_s_at	KIF1C	0.851	1.8	1.64E-07	4.31E-06
204785_x_at	IFNAR2	0.851	1.8	3.46E-06	4.88E-05
207861_at	CCL22	0.851	1.8	1.99E-04	1.26E-03
37145_at	GNLY	0.85	1.8	2.06E-06	3.22E-05
220356_at	CORIN	0.85	1.8	7.17E-05	5.54E-04
234946_at	ENTPD6	0.85	1.8	5.89E-05	4.71E-04
1555579_s_at	PTPRM	0.85	1.8	3.24E-08	1.19E-06
1554824_at	ZNF585A	0.849	1.8	1.12E-05	1.25E-04
204870_s_at	PCSK2	0.848	1.8	1.60E-05	1.67E-04
1552410_at	CLEC4F	0.848	1.8	6.47E-05	5.09E-04
230626_at	TSPAN12	0.848	1.8	1.70E-03	6.89E-03
239130_at	MIR101-1 /// MIR3671	0.848	1.8	1.02E-05	1.17E-04

203523_at	LSP1	0.848	1.8	1.89E-06	3.01E-05
227749_at	POU2F2	0.848	1.8	1.99E-08	7.93E-07
242957_at	VWCE	0.848	1.8	4.35E-06	5.87E-05
204199_at	RALGPS1	0.848	1.8	3.20E-09	1.80E-07
213213_at	DIDO1	0.848	1.8	4.92E-06	6.46E-05
236325_at	KIAA1377	0.847	1.8	1.92E-05	1.93E-04
1556228_a_at	VCPIP1	0.846	1.8	3.25E-10	2.76E-08
213559_s_at	ZNF467	0.846	1.8	5.84E-05	4.67E-04
1558711_at	FAM13A-AS1	0.845	1.8	3.12E-09	1.77E-07
230457_at	TMEM242	0.844	1.8	3.58E-06	5.02E-05
239309_at	DLX6	0.844	1.79	2.06E-05	2.05E-04
205898_at	CX3CR1	0.843	1.79	9.43E-07	1.70E-05
205830_at	CLGN	0.843	1.79	2.71E-05	2.55E-04
205878_at	POU6F1	0.843	1.79	1.30E-07	3.59E-06
215870_s_at	PLA2G5	0.842	1.79	3.58E-03	1.26E-02
205259_at	NR3C2	0.841	1.79	3.36E-07	7.52E-06
230539_at	FAM182A	0.841	1.79	8.19E-04	3.84E-03
238454_at	ZNF540	0.841	1.79	8.93E-08	2.65E-06
231986_at	RIMS1	0.841	1.79	1.71E-06	2.76E-05
1561589_a_at	NBEAL1	0.841	1.79	2.18E-05	2.15E-04
1552727_s_at	ADAMTS17	0.841	1.79	9.17E-07	1.66E-05
227138_at	CRTAP	0.841	1.79	1.15E-10	1.15E-08
228607_at	OAS2	0.841	1.79	6.33E-07	1.23E-05
212750_at	PPP1R16B	0.841	1.79	1.36E-08	5.72E-07
230422_at	FPR3	0.84	1.79	6.54E-05	5.13E-04
223533_at	LRRC8C	0.84	1.79	1.14E-04	8.08E-04
223694_at	TRIM7	0.84	1.79	1.58E-05	1.65E-04
239260_at	CORIN	0.84	1.79	1.62E-05	1.68E-04
236673_at	TIFAB	0.839	1.79	3.77E-07	8.24E-06
235891_at	DNM3OS /// MIR214 /// MIR3120	0.839	1.79	6.46E-07	1.26E-05
239582_at	PML	0.838	1.79	1.34E-08	5.64E-07
223710_at	CCL26	0.838	1.79	4.37E-04	2.34E-03
223522_at	MIR600 /// MIR600HG	0.838	1.79	5.19E-05	4.28E-04
227178_at	CELF2	0.837	1.79	2.07E-07	5.19E-06

206120_at	CD33	0.837	1.79	5.51E-07	1.10E-05
202032_s_at	MAN2A2	0.836	1.79	4.65E-11	5.33E-09
231980_at	DOK6	0.836	1.79	2.55E-04	1.53E-03
203559_s_at	ABP1	0.836	1.78	7.34E-07	1.40E-05
40420_at	STK10	0.836	1.78	3.80E-10	3.14E-08
241765_at	CPM	0.835	1.78	1.61E-06	2.63E-05
1560698_a_at	TRHDE-AS1	0.835	1.78	3.42E-04	1.93E-03
207381_at	ALOX12B	0.835	1.78	5.37E-03	1.75E-02
1560910_at	PPIL6	0.835	1.78	7.07E-05	5.46E-04
210185_at	CACNB1	0.835	1.78	3.43E-06	4.85E-05
213942_at	MEGF6	0.835	1.78	2.51E-07	6.02E-06
1556665_at	LOC101059957 /// TTC6	0.834	1.78	2.53E-05	2.42E-04
212827_at	IGHM	0.834	1.78	1.23E-03	5.33E-03
239683_at	CLYBL	0.834	1.78	3.02E-07	6.98E-06
236234_at	PDE1A	0.834	1.78	1.03E-05	1.17E-04
229105_at	GPR39	0.834	1.78	3.20E-04	1.83E-03
223211_at	HACL1	0.834	1.78	6.26E-06	7.80E-05
229800_at	DCLK1	0.834	1.78	7.15E-06	8.68E-05
204265_s_at	GPSM3	0.833	1.78	2.73E-08	1.04E-06
216481_at	GRIP2	0.833	1.78	6.27E-06	7.81E-05
205686_s_at	CD86	0.833	1.78	2.95E-08	1.11E-06
214147_at	MROH7	0.832	1.78	8.66E-05	6.46E-04
204502_at	SAMHD1	0.832	1.78	2.02E-06	3.16E-05
1553220_at	FAM117B	0.832	1.78	1.10E-05	1.23E-04
229348_at	UBIAD1	0.832	1.78	1.15E-03	5.04E-03
208131_s_at	PTGIS	0.831	1.78	1.83E-04	1.18E-03
239237_at	LOC100506776	0.831	1.78	3.74E-04	2.07E-03
37966_at	PARVB	0.831	1.78	1.22E-05	1.34E-04
235414_at	ZNF383	0.831	1.78	3.70E-07	8.11E-06
205473_at	ATP6V1B1	0.83	1.78	3.44E-04	1.94E-03
224162_s_at	FBXO31	0.83	1.78	1.22E-08	5.27E-07
214059_at	IFI44	0.83	1.78	1.97E-04	1.25E-03
219909_at	MMP28	0.83	1.78	7.34E-05	5.64E-04
223800_s_at	LIMS3 /// LIMS3L	0.83	1.78	7.84E-06	9.37E-05
228083_at	CACNA2D4	0.82	1.78	7.63E-06	9.17E-05

		9			
231406_at	ORAI2	0.828	1.78	1.46E-06	2.44E-05
1554003_at	ARHGEF28	0.828	1.78	3.55E-06	4.98E-05
214963_at	NUP160	0.828	1.77	5.41E-05	4.41E-04
204674_at	LRMP	0.828	1.77	3.43E-06	4.86E-05
203081_at	CTNNBIP1	0.828	1.77	1.19E-08	5.19E-07
210321_at	GZMH	0.826	1.77	6.10E-05	4.85E-04
206049_at	SELP	0.826	1.77	2.12E-04	1.32E-03
237783_at	PLAC8L1	0.826	1.77	2.77E-04	1.63E-03
221524_s_at	RRAGD	0.826	1.77	1.46E-07	3.94E-06
230238_at	SOWAHA	0.825	1.77	5.14E-05	4.24E-04
212588_at	PTPRC	0.825	1.77	1.09E-05	1.22E-04
238025_at	MLKL	0.824	1.77	4.40E-07	9.22E-06
244444_at	PKD1L2	0.824	1.77	4.95E-05	4.12E-04
223922_x_at	MS4A6A	0.823	1.77	6.37E-06	7.90E-05
203296_s_at	ATP1A2	0.823	1.77	6.25E-03	1.97E-02
220041_at	PIGZ	0.823	1.77	8.42E-06	9.93E-05
223767_at	GPR84	0.823	1.77	1.65E-07	4.33E-06
210571_s_at	CMAHP	0.823	1.77	1.20E-05	1.32E-04
222088_s_at	SLC2A14 /// SLC2A3	0.822	1.77	2.62E-04	1.56E-03
219127_at	PRR15L	0.822	1.77	2.94E-04	1.72E-03
1552633_at	ZNF101	0.822	1.77	8.32E-07	1.54E-05
227794_at	GLYATL1 /// LOC100287413	0.821	1.77	1.38E-03	5.86E-03
240048_at	STRC	0.821	1.77	1.21E-06	2.10E-05
239135_at	CPPED1	0.82	1.77	8.92E-08	2.65E-06
220016_at	AHNAK	0.82	1.77	9.17E-09	4.23E-07
237753_at	IL21R	0.82	1.77	2.26E-04	1.39E-03
220326_s_at	ARHGEF40	0.82	1.76	3.29E-07	7.42E-06
232570_s_at	ADAM33	0.819	1.76	1.26E-04	8.79E-04
217442_at	LOC100131825	0.818	1.76	8.78E-05	6.53E-04
228647_at	LOC100049716	0.818	1.76	6.97E-08	2.21E-06
204198_s_at	RUNX3	0.818	1.76	3.21E-07	7.29E-06
224735_at	CYB561A3	0.818	1.76	1.04E-10	1.06E-08
230425_at	EPHB1	0.818	1.76	4.96E-05	4.12E-04
239675_at	LINC00900	0.81	1.76	1.56E-04	1.04E-03

		7			
217690_at	ENOSF1	0.817	1.76	2.64E-07	6.26E-06
207901_at	IL12B	0.817	1.76	3.85E-04	2.12E-03
230780_at	LINC00886	0.817	1.76	4.66E-05	3.92E-04
203911_at	RAP1GAP	0.817	1.76	1.63E-06	2.65E-05
213837_at	L3MBTL1	0.817	1.76	1.08E-08	4.78E-07
235593_at	ZEB2	0.816	1.76	1.21E-04	8.48E-04
224027_at	CCL28	0.816	1.76	3.05E-08	1.13E-06
222362_at	AGFG2	0.815	1.76	1.25E-06	2.14E-05
210794_s_at	MEG3	0.815	1.76	2.14E-06	3.32E-05
228702_at	FLJ43663	0.815	1.76	1.37E-05	1.46E-04
210895_s_at	CD86	0.815	1.76	8.20E-08	2.50E-06
235173_at	MBNL1-AS1	0.814	1.76	4.60E-04	2.44E-03
201422_at	IFI30 /// PIK3R2	0.814	1.76	1.27E-08	5.43E-07
232311_at	B2M	0.814	1.76	9.26E-07	1.68E-05
209933_s_at	CD300A	0.814	1.76	1.24E-06	2.14E-05
209374_s_at	IGHM	0.813	1.76	9.87E-04	4.48E-03
204821_at	BTN3A3	0.813	1.76	4.75E-09	2.49E-07
202075_s_at	PLTP	0.813	1.76	5.39E-10	4.18E-08
236538_at	GRIA2	0.813	1.76	6.07E-03	1.93E-02
214046_at	FUT9	0.813	1.76	2.28E-04	1.40E-03
244070_at	SYNE1	0.812	1.76	1.74E-06	2.81E-05
209546_s_at	APOL1	0.812	1.76	1.42E-06	2.40E-05
224225_s_at	ETV7	0.812	1.76	6.57E-05	5.14E-04
228177_at	CREBBP	0.812	1.76	4.77E-07	9.83E-06
218126_at	RMDN3	0.812	1.76	7.90E-08	2.42E-06
234312_s_at	ACSS2	0.811	1.75	6.11E-05	4.86E-04
226558_at	LOC389834 /// MAFIP /// TEKT4P2	0.811	1.75	3.95E-03	1.36E-02
220351_at	CCRL1	0.81	1.75	4.89E-04	2.56E-03
206741_at	SSUH2	0.81	1.75	3.77E-04	2.08E-03
1554179_s_at	LYNX1	0.81	1.75	3.49E-03	1.23E-02
228802_at	RBPMS2	0.81	1.75	6.51E-04	3.21E-03
1557283_a_at	ZNF519	0.808	1.75	1.07E-05	1.21E-04
213611_at	AQP5	0.808	1.75	1.97E-02	4.95E-02
226610_at	CENPV	0.80	1.75	1.55E-06	2.55E-05

		7			
220622_at	LRRC31	0.807	1.75	2.79E-06	4.11E-05
205749_at	CYP1A1	0.806	1.75	3.21E-03	1.15E-02
214735_at	IPCEF1	0.806	1.75	3.39E-07	7.57E-06
206576_s_at	CEACAM1	0.805	1.75	1.01E-04	7.29E-04
1554878_a_at	ABCD3	0.805	1.75	1.25E-04	8.70E-04
229523_at	TMEM200C	0.805	1.75	3.52E-06	4.95E-05
50965_at	RAB26	0.805	1.75	8.68E-06	1.02E-04
205081_at	CRIP1	0.804	1.75	1.81E-06	2.89E-05
236309_x_at	ZMIZ2	0.804	1.75	1.25E-08	5.37E-07
239425_at	DCUN1D5	0.804	1.75	2.39E-07	5.82E-06
35974_at	LRMP	0.804	1.75	1.06E-05	1.19E-04
242316_at	TMOD3	0.803	1.75	1.93E-03	7.64E-03
207443_at	NR2E1	0.803	1.74	6.50E-05	5.10E-04
235639_at	CDH19	0.803	1.74	1.53E-05	1.60E-04
228290_at	PLK1S1	0.803	1.74	2.73E-06	4.05E-05
207466_at	GAL	0.803	1.74	1.47E-02	3.92E-02
214180_at	MAN1C1	0.802	1.74	1.70E-07	4.43E-06
205522_at	HOXD4	0.802	1.74	1.79E-05	1.83E-04
227606_s_at	STAMBPL1	0.801	1.74	9.37E-08	2.75E-06
226486_at	MTERFD2	0.801	1.74	6.20E-06	7.74E-05
203000_at	STMN2	0.801	1.74	2.48E-04	1.50E-03
207583_at	ABCD2	0.801	1.74	4.67E-04	2.47E-03
220460_at	SLCO1C1	0.8	1.74	1.77E-05	1.81E-04
1559839_at	TBX18	0.8	1.74	6.28E-04	3.12E-03
205880_at	PRKD1	0.8	1.74	5.59E-08	1.85E-06
226679_at	SLC26A11	0.799	1.74	5.24E-07	1.06E-05
213325_at	PVRL3	0.799	1.74	6.19E-06	7.73E-05
219525_at	SLC47A1	0.799	1.74	7.10E-05	5.48E-04
215536_at	HLA-DQB2	0.799	1.74	3.53E-04	1.97E-03
236465_at	RNF175	0.799	1.74	2.39E-05	2.31E-04
236514_at	ACOT8	0.799	1.74	7.39E-07	1.41E-05
213036_x_at	ATP2A3	0.799	1.74	6.58E-04	3.24E-03
214494_s_at	SPG7	0.798	1.74	5.08E-08	1.70E-06

206625_at	PRPH2	0.798	1.74	1.71E-03	6.92E-03
203438_at	STC2	0.798	1.74	1.65E-03	6.72E-03
230773_at	ZNF385D	0.797	1.74	8.50E-05	6.37E-04
239186_at	MGC39372	0.797	1.74	2.46E-03	9.26E-03
233647_s_at	CDADC1	0.796	1.74	9.04E-08	2.68E-06
219889_at	FRAT1	0.796	1.74	2.57E-07	6.15E-06
230443_at	NHP2L1	0.796	1.74	1.14E-05	1.27E-04
217521_at	HAL	0.795	1.74	2.66E-03	9.91E-03
226595_at	SNX21	0.795	1.74	2.49E-05	2.39E-04
201137_s_at	HLA-DPB1	0.795	1.74	1.62E-06	2.64E-05
1559584_a_at	C16orf54	0.795	1.73	1.04E-03	4.69E-03
216149_at	LRRC37BP1	0.795	1.73	1.56E-03	6.44E-03
209479_at	CCDC28A	0.795	1.73	1.50E-11	2.08E-09
1559144_x_at	LINC00910	0.795	1.73	5.08E-07	1.03E-05
207757_at	ZFP2	0.794	1.73	4.29E-05	3.67E-04
230184_at	LOC100506421	0.794	1.73	5.30E-07	1.06E-05
210179_at	KCNJ13	0.794	1.73	2.29E-04	1.41E-03
232794_at	LOC153682	0.794	1.73	5.36E-06	6.92E-05
206502_s_at	INSM1	0.794	1.73	1.32E-04	9.11E-04
230031_at	HSPA5	0.793	1.73	3.65E-08	1.30E-06
203561_at	FCGR2A	0.793	1.73	7.62E-06	9.17E-05
228641_at	CARD8	0.793	1.73	4.41E-09	2.34E-07
237974_at	ABHD12B /// MIR4454	0.793	1.73	1.23E-06	2.13E-05
214890_s_at	FAM149A	0.793	1.73	3.81E-05	3.34E-04
228727_at	ANXA11	0.792	1.73	1.06E-05	1.20E-04
222778_s_at	WHSC1	0.792	1.73	3.73E-06	5.18E-05
230751_at	WNT4	0.791	1.73	1.93E-04	1.23E-03
222905_s_at	TMEM143	0.791	1.73	3.26E-07	7.35E-06
244779_at	ZDHHC2	0.791	1.73	8.46E-06	9.96E-05
222764_at	ASRGL1	0.791	1.73	1.72E-07	4.46E-06
208268_at	ADAM28	0.791	1.73	1.35E-06	2.29E-05
206572_x_at	ZNF85	0.791	1.73	8.39E-05	6.30E-04
210517_s_at	AKAP12	0.791	1.73	9.64E-05	7.03E-04

1552726_at	ADAMTS17	0.79	1.73	1.61E-04	1.06E-03
239345_at	SLC19A3	0.79	1.73	9.89E-04	4.48E-03
204908_s_at	BCL3	0.79	1.73	6.48E-05	5.09E-04
220435_at	SLC30A10	0.79	1.73	7.15E-03	2.20E-02
209948_at	KCNMB1	0.79	1.73	1.68E-04	1.10E-03
216191_s_at	TRDV3	0.79	1.73	7.83E-03	2.37E-02
244627_at	DAK	0.79	1.73	3.82E-09	2.09E-07
216331_at	ITGA7	0.789	1.73	2.04E-05	2.03E-04
223999_at	PPIL2	0.789	1.73	2.70E-05	2.54E-04
219799_s_at	DHRS9	0.788	1.73	5.66E-04	2.87E-03
212735_at	KIAA0226	0.788	1.73	4.70E-06	6.25E-05
241782_at	NEBL	0.788	1.73	3.73E-06	5.18E-05
221142_s_at	PECR	0.787	1.72	8.71E-03	2.57E-02
222218_s_at	PILRA	0.786	1.72	6.14E-06	7.69E-05
242388_x_at	TAGAP	0.786	1.72	2.29E-05	2.23E-04
220061_at	ACSM5	0.786	1.72	2.40E-05	2.32E-04
238905_at	RHOJ	0.786	1.72	1.62E-06	2.64E-05
1557557_at	MATN1-AS1	0.786	1.72	1.18E-07	3.33E-06
239142_at	RFESD	0.786	1.72	2.66E-06	3.98E-05
206283_s_at	TAL1	0.785	1.72	1.26E-04	8.76E-04
238746_at	PXMP4	0.785	1.72	7.27E-07	1.39E-05
206955_at	AQP7 /// LOC100509620	0.785	1.72	1.47E-02	3.90E-02
204118_at	CD48	0.785	1.72	3.36E-04	1.90E-03
237748_at	SCGB2B2	0.785	1.72	3.85E-03	1.33E-02
209016_s_at	KRT7	0.785	1.72	6.15E-03	1.95E-02
229628_s_at	CCDC180 /// LOC100499484-C9ORF174	0.784	1.72	2.79E-06	4.12E-05
235913_at	ZNF880	0.784	1.72	6.44E-08	2.07E-06
243681_at	SHANK2	0.784	1.72	7.88E-03	2.38E-02
214651_s_at	HOXA10-HOXA9 /// HOXA9 /// MIR196B	0.783	1.72	5.59E-04	2.84E-03
209721_s_at	IFFO1	0.783	1.72	5.44E-07	1.09E-05
230197_s_at	TPPP	0.783	1.72	1.58E-04	1.05E-03
240063_at	GUSBP5	0.783	1.72	5.21E-05	4.29E-04
205765_at	CYP3A5	0.782	1.72	5.65E-05	4.57E-04
1561666_a_at	PSMG3-AS1	0.782	1.72	1.59E-05	1.65E-04
204438_at	MRC1	0.782	1.72	4.16E-07	8.86E-06

219801_at	ZNF34	0.782	1.72	4.67E-07	9.68E-06
214377_s_at	CTRL	0.782	1.72	4.33E-07	9.09E-06
209160_at	AKR1C3	0.782	1.72	4.30E-07	9.07E-06
243403_x_at	CPM	0.781	1.72	4.12E-08	1.42E-06
234165_at	PTGDR	0.781	1.72	2.90E-04	1.70E-03
238364_x_at	GLI4	0.781	1.72	1.33E-05	1.43E-04
224583_at	COTL1	0.781	1.72	5.86E-05	4.69E-04
220327_at	VGLL3	0.781	1.72	3.06E-05	2.80E-04
234841_x_at	OBP2A	0.781	1.72	6.85E-06	8.38E-05
206239_s_at	SPINK1	0.781	1.72	1.56E-05	1.63E-04
204151_x_at	AKR1C1	0.78	1.72	2.75E-05	2.58E-04
210233_at	IL1RAP	0.78	1.72	1.18E-04	8.33E-04
226047_at	MRVI1	0.78	1.72	4.18E-04	2.26E-03
210385_s_at	ERAP1	0.78	1.72	3.23E-04	1.84E-03
226972_s_at	CCDC136	0.78	1.72	2.91E-05	2.69E-04
1554240_a_at	ITGAL	0.78	1.72	1.56E-05	1.63E-04
221805_at	NEFL	0.779	1.72	2.51E-04	1.51E-03
230756_at	ZNF683	0.779	1.72	7.14E-10	5.26E-08
212091_s_at	COL6A1	0.779	1.72	1.29E-07	3.56E-06
225033_at	ST3GAL1	0.778	1.72	7.71E-06	9.24E-05
233111_at	PTCSC1	0.778	1.72	7.66E-07	1.44E-05
1553683_s_at	FBXL14	0.778	1.71	3.92E-07	8.46E-06
1556454_a_at	LOC100506274	0.778	1.71	2.28E-06	3.50E-05
210262_at	CRISP2	0.777	1.71	3.32E-03	1.18E-02
205762_s_at	DUS4L	0.777	1.71	8.65E-08	2.59E-06
211990_at	HLA-DPA1	0.777	1.71	1.62E-03	6.64E-03
206393_at	TNNI2	0.777	1.71	6.55E-06	8.07E-05
213820_s_at	STARD5	0.776	1.71	9.24E-07	1.67E-05
222592_s_at	ACSL5	0.776	1.71	4.34E-04	2.33E-03
216237_s_at	MCM5	0.776	1.71	1.28E-05	1.39E-04
235292_at	FLJ32255	0.775	1.71	1.33E-05	1.43E-04
1557062_at	LOC100129195	0.775	1.71	3.03E-07	7.00E-06
230538_at	SHC4	0.775	1.71	4.23E-05	3.64E-04
236738_at	C3orf80	0.774	1.71	1.17E-02	3.25E-02
204272_at	LGALS4	0.774	1.71	1.69E-03	6.87E-03

204846_at	CP	0.774	1.71	1.80E-03	7.21E-03
205304_s_at	KCNJ8	0.773	1.71	1.54E-05	1.62E-04
229741_at	MAVS	0.773	1.71	7.40E-10	5.43E-08
227429_at	EFCAB4A	0.773	1.71	5.84E-05	4.67E-04
44790_s_at	KIAA0226L	0.773	1.71	8.77E-05	6.53E-04
244251_at	LCP2	0.773	1.71	2.12E-05	2.10E-04
231175_at	BEND6	0.773	1.71	8.94E-05	6.62E-04
1561985_at	C14orf39	0.772	1.71	5.54E-03	1.79E-02
219019_at	PIDD	0.772	1.71	6.88E-04	3.35E-03
239685_at	LINC00900	0.772	1.71	1.41E-05	1.49E-04
1557116_at	APOL6	0.772	1.71	8.57E-06	1.01E-04
227610_at	TSPAN11	0.772	1.71	5.68E-05	4.58E-04
234929_s_at	SPATA7	0.772	1.71	1.57E-06	2.58E-05
221584_s_at	KCNMA1	0.771	1.71	5.42E-05	4.42E-04
218885_s_at	GALNT12	0.771	1.71	7.06E-06	8.60E-05
242720_at	ITIH4	0.771	1.71	1.49E-03	6.22E-03
225353_s_at	C1QC	0.77	1.71	8.91E-07	1.63E-05
230882_at	DLX6-AS1	0.77	1.7	4.70E-04	2.48E-03
239694_at	TRIM7	0.769	1.7	6.53E-05	5.12E-04
230250_at	PTPRB	0.769	1.7	5.09E-05	4.21E-04
203320_at	SH2B3	0.769	1.7	1.53E-06	2.54E-05
205904_at	MICA	0.768	1.7	4.75E-06	6.29E-05
205585_at	ETV6	0.768	1.7	7.72E-05	5.89E-04
229698_at	SHANK3	0.768	1.7	8.06E-07	1.50E-05
210258_at	RGS13	0.768	1.7	2.26E-03	8.68E-03
232253_at	C5orf56	0.767	1.7	2.03E-07	5.12E-06
1564706_s_at	GLS2	0.767	1.7	2.66E-05	2.52E-04
220416_at	ATP8B4	0.767	1.7	1.11E-05	1.24E-04
214453_s_at	IFI44	0.767	1.7	2.81E-05	2.62E-04
210664_s_at	TFPI	0.767	1.7	5.78E-05	4.64E-04
225764_at	ETV6	0.766	1.7	4.41E-09	2.34E-07
229872_s_at	LOC100132999 /// LOC100996473 /// LOC100996720 /// LOC100996740 /// LOC101060404 /// LOC101060562 /// LOC101060645 /// LOC101060698 /// LOC642441 /// LOC730256	0.766	1.7	4.30E-07	9.07E-06
214156_at	MYRIP	0.766	1.7	2.23E-04	1.38E-03

229687_s_at	PRDM11	0.765	1.7	1.75E-04	1.14E-03
219033_at	PARP8	0.765	1.7	2.24E-08	8.80E-07
214032_at	ZAP70	0.765	1.7	1.17E-08	5.14E-07
212276_at	LPIN1	0.765	1.7	3.57E-05	3.16E-04
206271_at	TLR3	0.765	1.7	5.18E-07	1.05E-05
220331_at	CYP46A1	0.764	1.7	5.05E-05	4.18E-04
228207_at	LOC100499489	0.764	1.7	9.70E-09	4.41E-07
1553213_a_at	KRT78	0.764	1.7	3.72E-03	1.30E-02
211421_s_at	RET	0.764	1.7	1.50E-03	6.25E-03
206337_at	CCR7	0.763	1.7	2.52E-05	2.41E-04
239074_at	GRAPL	0.763	1.7	4.20E-04	2.27E-03
222101_s_at	DCHS1	0.763	1.7	1.72E-04	1.12E-03
226776_at	ENY2	0.763	1.7	7.52E-07	1.42E-05
202803_s_at	ITGB2	0.763	1.7	4.61E-05	3.88E-04
216513_at	DCT	0.763	1.7	5.22E-03	1.71E-02
219429_at	FA2H	0.763	1.7	1.29E-04	8.92E-04
227757_at	CUL4A	0.763	1.7	1.34E-04	9.18E-04
1552400_a_at	C15orf27	0.762	1.7	7.47E-05	5.72E-04
243880_at	GOSR2	0.762	1.7	3.32E-04	1.89E-03
225597_at	SLC45A4	0.762	1.7	1.27E-07	3.52E-06
37986_at	EPOR	0.761	1.7	2.41E-07	5.85E-06
206345_s_at	PON1	0.761	1.69	1.43E-03	6.02E-03
239802_at	SAP30L	0.761	1.69	4.13E-06	5.63E-05
227486_at	NT5E	0.761	1.69	5.08E-04	2.63E-03
213849_s_at	PPP2R2B	0.76	1.69	9.62E-06	1.11E-04
219778_at	ZFPM2	0.76	1.69	5.06E-06	6.61E-05
226305_at	LYNX1	0.76	1.69	1.92E-04	1.22E-03
207016_s_at	ALDH1A2	0.76	1.69	1.68E-04	1.10E-03
230497_at	CELF5	0.759	1.69	1.28E-05	1.39E-04
207610_s_at	EMR2	0.759	1.69	5.32E-06	6.89E-05
208606_s_at	WNT4	0.759	1.69	5.25E-04	2.70E-03
219261_at	C7orf26	0.759	1.69	3.41E-07	7.59E-06
224932_at	CHCHD10	0.759	1.69	6.10E-06	7.66E-05
205790_at	SKAP1	0.759	1.69	1.15E-06	2.00E-05

1555363_s_at	LINC00663	0.759	1.69	2.32E-04	1.42E-03
207037_at	TNFRSF11A	0.758	1.69	1.17E-05	1.30E-04
206157_at	PTX3	0.758	1.69	1.14E-04	8.06E-04
212360_at	AMPD2	0.758	1.69	2.13E-06	3.30E-05
230802_at	ARHGAP24	0.757	1.69	4.09E-04	2.22E-03
242972_at	HCG18	0.757	1.69	1.37E-04	9.38E-04
208335_s_at	DARC	0.757	1.69	5.81E-08	1.91E-06
242207_at	CENPP /// LOC100128361	0.756	1.69	5.36E-05	4.38E-04
216594_x_at	AKR1C1	0.756	1.69	5.05E-05	4.18E-04
230531_at	KCNC3	0.756	1.69	1.00E-06	1.79E-05
243099_at	NFAM1	0.756	1.69	5.12E-10	4.04E-08
242426_at	NRG4	0.755	1.69	4.97E-06	6.51E-05
1564705_at	GLS2	0.755	1.69	1.63E-04	1.08E-03
1556319_at	LOC283270	0.754	1.69	2.46E-06	3.73E-05
244485_at	HLA-DPB1	0.754	1.69	6.21E-03	1.96E-02
228677_s_at	RASAL3	0.754	1.69	7.95E-07	1.49E-05
235827_at	LOC100506472 /// TAB1	0.753	1.69	5.57E-06	7.12E-05
219727_at	DUOX2	0.753	1.68	3.96E-05	3.45E-04
203508_at	TNFRSF1B	0.752	1.68	8.71E-08	2.60E-06
218999_at	TMEM140	0.752	1.68	3.07E-06	4.44E-05
214221_at	ALMS1	0.751	1.68	1.17E-06	2.04E-05
223974_at	DLGAP1-AS2	0.751	1.68	2.95E-04	1.72E-03
227482_at	ADCK1	0.75	1.68	2.09E-04	1.31E-03
205127_at	PTGS1	0.75	1.68	1.88E-03	7.47E-03
1555880_at	ERVFRD-1	0.75	1.68	1.54E-04	1.03E-03
227344_at	IKZF1	0.75	1.68	5.61E-06	7.16E-05
214981_at	POSTN	0.75	1.68	4.46E-06	5.98E-05
48580_at	CXXC1	0.75	1.68	5.28E-06	6.86E-05
1554704_at	ATP8B3	0.749	1.68	2.75E-05	2.58E-04
223836_at	FGFBP2	0.749	1.68	7.93E-03	2.39E-02
243547_at	LINC00921 /// ZNF263	0.749	1.68	3.57E-06	5.01E-05
233083_at	MTHFD2L	0.748	1.68	1.06E-05	1.19E-04
206850_at	RASL10A	0.748	1.68	3.65E-04	2.03E-03
219195_at	PPARGC1A	0.748	1.68	1.41E-03	5.95E-03
1559591_s_at	CHDH	0.748	1.68	1.48E-06	2.46E-05

224463_s_at	C11orf70	0.748	1.68	2.36E-05	2.29E-04
1554036_at	ZBTB24	0.747	1.68	3.21E-03	1.15E-02
210168_at	C6	0.747	1.68	8.18E-03	2.45E-02
219593_at	SLC15A3	0.747	1.68	1.56E-09	9.90E-08
205484_at	SIT1	0.747	1.68	1.22E-05	1.34E-04
213411_at	ADAM22	0.747	1.68	2.81E-05	2.62E-04
208056_s_at	CBFA2T3	0.746	1.68	2.41E-04	1.47E-03
204671_s_at	ANKRD6	0.746	1.68	3.71E-05	3.27E-04
217284_x_at	SERHL2	0.746	1.68	1.63E-03	6.65E-03
1553970_s_at	CEL	0.746	1.68	1.56E-03	6.42E-03
1558199_at	FN1	0.746	1.68	1.46E-03	6.11E-03
225224_at	C20orf112	0.746	1.68	4.73E-07	9.76E-06
1553614_a_at	FLJ25694	0.745	1.68	9.14E-05	6.75E-04
226459_at	PIK3AP1	0.745	1.68	8.15E-07	1.52E-05
212268_at	SERPINB1	0.745	1.68	4.93E-05	4.10E-04
236728_at	LNPEP	0.745	1.68	2.97E-03	1.08E-02
205298_s_at	BTN2A2	0.745	1.68	7.41E-07	1.41E-05
202637_s_at	ICAM1	0.745	1.68	2.84E-07	6.66E-06
232662_x_at	FAM213A	0.744	1.68	1.27E-08	5.42E-07
228206_at	HS3ST4	0.744	1.68	1.46E-05	1.54E-04
229964_at	C9orf152	0.744	1.67	6.00E-04	3.01E-03
240288_at	KCNRG /// TRIM13	0.744	1.67	2.84E-05	2.64E-04
204776_at	THBS4	0.743	1.67	7.77E-03	2.35E-02
235489_at	RHOJ	0.743	1.67	7.07E-08	2.23E-06
1552544_at	SERPINA12	0.743	1.67	9.08E-03	2.65E-02
228617_at	XAF1	0.743	1.67	9.33E-06	1.08E-04
222057_at	NOL12	0.743	1.67	6.73E-04	3.29E-03
219684_at	RTP4	0.743	1.67	1.58E-04	1.05E-03
235557_at	GPAT2	0.743	1.67	4.45E-03	1.50E-02
212793_at	DAAM2	0.743	1.67	3.71E-07	8.13E-06
224505_s_at	PLCD4	0.742	1.67	3.01E-04	1.75E-03
242372_s_at	MFSD4	0.742	1.67	1.16E-04	8.19E-04
238846_at	TNFRSF11A	0.742	1.67	2.14E-05	2.11E-04

205338_s_at	DCT	0.741	1.67	4.48E-03	1.51E-02
227342_s_at	MYEOV	0.741	1.67	8.73E-06	1.02E-04
201787_at	FBLN1	0.741	1.67	5.98E-06	7.55E-05
1557359_at	LOC285758	0.741	1.67	4.59E-04	2.44E-03
226030_at	ACADSB	0.741	1.67	3.93E-08	1.37E-06
224110_at	PRO1804	0.74	1.67	3.54E-05	3.14E-04
235688_s_at	TRAF4	0.74	1.67	8.23E-05	6.21E-04
228593_at	MTMR9LP	0.74	1.67	1.97E-05	1.97E-04
227582_at	KLHDC9	0.74	1.67	1.23E-04	8.60E-04
227702_at	CYP4X1	0.74	1.67	3.85E-06	5.32E-05
204530_s_at	TOX	0.74	1.67	1.72E-06	2.78E-05
217684_at	TYMS	0.74	1.67	2.39E-05	2.31E-04
235634_at	PURG	0.739	1.67	1.09E-03	4.86E-03
206170_at	ADRB2	0.739	1.67	7.10E-09	3.45E-07
213645_at	ENOSF1	0.739	1.67	2.34E-08	9.12E-07
209592_s_at	DCAF7	0.739	1.67	2.16E-08	8.55E-07
211883_x_at	CEACAM1	0.739	1.67	2.81E-07	6.59E-06
239477_at	EFHB	0.739	1.67	1.46E-02	3.89E-02
1553099_at	TIGD1	0.738	1.67	1.17E-04	8.27E-04
207082_at	CSF1	0.738	1.67	5.61E-08	1.85E-06
216611_s_at	SLC6A2	0.738	1.67	2.96E-05	2.73E-04
218957_s_at	PAAF1	0.738	1.67	7.04E-07	1.35E-05
224374_s_at	EMILIN2	0.737	1.67	2.55E-04	1.53E-03
1560556_a_at	PLEKHA8	0.737	1.67	5.06E-05	4.19E-04
206421_s_at	SERPINB7	0.737	1.67	7.86E-03	2.37E-02
226906_s_at	ARHGAP9	0.737	1.67	3.15E-06	4.53E-05
202192_s_at	GAS7	0.737	1.67	1.19E-10	1.17E-08
207317_s_at	CASQ2	0.736	1.67	9.75E-03	2.81E-02
219983_at	HRASLS	0.736	1.67	4.46E-03	1.50E-02
211034_s_at	HECTD4	0.736	1.67	8.63E-08	2.59E-06
210451_at	PKLR	0.736	1.67	3.20E-03	1.15E-02
209522_s_at	CRAT	0.736	1.67	2.46E-03	9.28E-03
220961_s_at	TBRG4	0.736	1.67	1.80E-04	1.16E-03
221801_x_at	NEFL	0.736	1.67	4.66E-05	3.93E-04
214774_x_at	TOX3	0.736	1.67	3.14E-03	1.13E-02

229484_at	PPM1J	0.735	1.66	5.47E-05	4.45E-04
232024_at	GIMAP2	0.735	1.66	1.29E-07	3.57E-06
207328_at	ALOX15	0.734	1.66	5.74E-04	2.90E-03
220064_at	LOC100506124 /// TTC21B	0.734	1.66	2.05E-05	2.03E-04
228667_at	AGPAT4	0.734	1.66	3.28E-04	1.87E-03
227908_at	TBC1D24	0.734	1.66	5.27E-07	1.06E-05
202465_at	PCOLCE	0.733	1.66	1.83E-07	4.68E-06
235174_s_at	LOC100128822	0.733	1.66	1.60E-04	1.06E-03
244468_at	PDZK1P1	0.733	1.66	4.48E-05	3.80E-04
214574_x_at	LST1	0.732	1.66	1.81E-06	2.90E-05
217626_at	AKR1C1	0.732	1.66	1.61E-03	6.59E-03
1553212_at	KRT78	0.732	1.66	1.26E-02	3.45E-02
204789_at	FMNL1	0.732	1.66	2.57E-08	9.84E-07
214409_at	RFPL3S	0.732	1.66	1.18E-05	1.31E-04
1552497_a_at	SLAMF6	0.732	1.66	2.53E-04	1.52E-03
228152_s_at	DDX60L	0.732	1.66	2.15E-05	2.12E-04
227721_at	CPAMD8	0.732	1.66	9.90E-04	4.49E-03
242561_at	IPO9	0.731	1.66	3.97E-06	5.46E-05
209670_at	TRAC	0.731	1.66	4.38E-06	5.90E-05
206822_s_at	L3MBTL1	0.731	1.66	8.24E-04	3.87E-03
223220_s_at	PARP9	0.731	1.66	9.47E-07	1.71E-05
205204_at	NMB	0.731	1.66	1.01E-04	7.31E-04
222139_at	ERV3-2	0.731	1.66	2.52E-04	1.52E-03
209906_at	C3AR1	0.73	1.66	1.15E-04	8.10E-04
229055_at	GPR68	0.73	1.66	1.18E-04	8.29E-04
204548_at	STAR	0.73	1.66	8.93E-07	1.63E-05
214169_at	SUN1	0.73	1.66	1.47E-04	9.92E-04
1568609_s_at	FLJ39739 /// LINC00623 /// LINC00869 /// LINC00875 /// LOC728875	0.73	1.66	2.43E-06	3.68E-05
216546_s_at	CHI3L1	0.73	1.66	4.94E-03	1.63E-02
205571_at	LIPT1	0.73	1.66	1.60E-05	1.67E-04
209083_at	CORO1A	0.729	1.66	7.16E-06	8.70E-05
204052_s_at	SFRP4	0.729	1.66	4.07E-03	1.39E-02
230384_at	ANKRD23	0.729	1.66	1.01E-03	4.56E-03
210683_at	NRTN	0.729	1.66	1.47E-06	2.46E-05
222890_at	CCDC113	0.729	1.66	1.03E-07	2.96E-06

244398_x_at	ZNF684	0.729	1.66	8.55E-08	2.57E-06
229230_at	SLC51A	0.729	1.66	4.20E-09	2.25E-07
1556346_at	COTL1	0.729	1.66	2.34E-05	2.27E-04
236204_at	COPS8	0.729	1.66	1.17E-05	1.30E-04
206219_s_at	VAV1	0.729	1.66	5.82E-06	7.39E-05
220615_s_at	FAR2	0.728	1.66	1.30E-02	3.53E-02
222520_s_at	IFT57	0.728	1.66	8.77E-05	6.53E-04
227131_at	MAP3K3	0.728	1.66	1.05E-08	4.69E-07
229734_at	MIR4697HG	0.727	1.66	2.30E-07	5.63E-06
1555898_at	ANKRD36C	0.727	1.66	1.80E-06	2.88E-05
223276_at	SMIM3	0.727	1.65	6.42E-04	3.18E-03
226201_at	DOT1L	0.727	1.65	2.93E-04	1.71E-03
209770_at	BTN3A1	0.726	1.65	6.92E-07	1.33E-05
226743_at	SLFN11	0.726	1.65	1.05E-06	1.86E-05
230435_at	FAM228B	0.726	1.65	4.07E-06	5.57E-05
226969_at	MTR	0.726	1.65	6.96E-09	3.39E-07
229509_at	MFSD8	0.726	1.65	1.96E-09	1.22E-07
229603_at	BBS12	0.725	1.65	7.24E-06	8.76E-05
226805_at	FITM2	0.725	1.65	1.98E-06	3.11E-05
227217_at	WNK2	0.725	1.65	1.27E-03	5.48E-03
204820_s_at	BTN3A2 /// BTN3A3	0.725	1.65	1.03E-07	2.97E-06
1553746_a_at	OTOGL	0.725	1.65	8.42E-08	2.54E-06
222124_at	HIF3A	0.725	1.65	9.89E-03	2.85E-02
210718_s_at	ARL17A /// ARL17B /// LOC100294341 /// LOC100996709	0.724	1.65	9.29E-03	2.70E-02
228770_at	GPR146	0.724	1.65	5.04E-05	4.18E-04
205776_at	FMO5 /// LOC101060372	0.724	1.65	6.51E-05	5.11E-04
218730_s_at	OGN	0.724	1.65	1.48E-02	3.94E-02
232686_at	SIGLEC17P	0.723	1.65	4.75E-03	1.58E-02
211018_at	LSS	0.723	1.65	1.31E-04	9.04E-04
203158_s_at	GLS	0.723	1.65	1.63E-08	6.71E-07
220384_at	NME8	0.723	1.65	1.24E-03	5.38E-03
210982_s_at	HLA-DRA	0.723	1.65	2.95E-04	1.72E-03
212732_at	MEG3	0.723	1.65	1.71E-05	1.76E-04

1564139_at	A2M-AS1	0.723	1.65	2.25E-06	3.46E-05
232812_at	LOC401052	0.722	1.65	1.16E-03	5.08E-03
244452_at	ERLEC1	0.722	1.65	1.08E-03	4.83E-03
204158_s_at	TCIRG1	0.722	1.65	6.14E-04	3.06E-03
226900_at	GABPB1-AS1	0.722	1.65	1.08E-07	3.08E-06
206804_at	CD3G	0.722	1.65	7.36E-05	5.65E-04
213952_s_at	ALOX5	0.722	1.65	2.23E-06	3.43E-05
229649_at	NRXN3	0.721	1.65	3.31E-05	2.97E-04
206349_at	LGI1	0.721	1.65	4.95E-06	6.50E-05
219870_at	ATF7IP2	0.721	1.65	2.72E-06	4.04E-05
209500_x_at	TNFSF12 /// TNFSF12-TNFSF13 /// TNFSF13	0.721	1.65	5.42E-06	6.99E-05
202962_at	KIF13B	0.721	1.65	2.01E-06	3.16E-05
205042_at	GNE	0.721	1.65	2.14E-04	1.33E-03
229887_at	ALS2CL	0.721	1.65	1.33E-04	9.16E-04
236305_at	RFESD	0.721	1.65	1.76E-04	1.14E-03
226745_at	CYP4V2	0.72	1.65	7.20E-05	5.55E-04
219232_s_at	EGLN3	0.72	1.65	2.73E-05	2.56E-04
207351_s_at	SH2D2A	0.72	1.65	6.57E-06	8.09E-05
207840_at	CD160 /// LOC101060629	0.72	1.65	2.70E-05	2.54E-04
205905_s_at	MICA /// MICB	0.72	1.65	5.55E-06	7.10E-05
232222_at	C18orf49 /// ZCCHC2	0.72	1.65	2.78E-04	1.64E-03
213116_at	NEK3	0.719	1.65	2.29E-07	5.62E-06
229003_x_at	FAM69B	0.719	1.65	4.74E-04	2.50E-03
237411_at	ADAMTS6	0.718	1.65	1.05E-03	4.69E-03
1564426_x_at	LOC729732	0.718	1.65	1.34E-03	5.71E-03
1553260_s_at	ALS2CR11	0.718	1.64	1.59E-04	1.05E-03
229842_at	ELF3	0.718	1.64	2.35E-04	1.44E-03
227923_at	SHANK3	0.718	1.64	2.23E-05	2.19E-04
210832_x_at	PTGER3	0.718	1.64	3.61E-05	3.20E-04
227113_at	ADHFE1	0.718	1.64	2.65E-05	2.51E-04
225763_at	RCSD1	0.717	1.64	3.89E-07	8.42E-06
237719_x_at	RGS7BP	0.717	1.64	2.39E-03	9.07E-03
1560981_a_at	PPARA	0.717	1.64	2.52E-04	1.52E-03
202638_s_at	ICAM1	0.717	1.64	1.98E-04	1.25E-03
45749_at	FAM65A	0.717	1.64	7.83E-10	5.66E-08

1563369_at	LINC00173	0.717	1.64	6.28E-04	3.12E-03
1568600_at	CALML4	0.716	1.64	8.24E-06	9.76E-05
238422_at	LBX2-AS1	0.716	1.64	2.98E-04	1.73E-03
217997_at	PHLDA1	0.716	1.64	6.84E-04	3.33E-03
214255_at	ATP10A	0.716	1.64	7.98E-07	1.49E-05
230499_at	BIRC3	0.716	1.64	9.79E-07	1.76E-05
204614_at	SERPINB2	0.716	1.64	1.06E-03	4.75E-03
219990_at	E2F8	0.716	1.64	1.13E-05	1.26E-04
221523_s_at	RRAGD	0.716	1.64	1.39E-06	2.35E-05
235515_at	SYNE4	0.715	1.64	1.08E-03	4.83E-03
204770_at	TAP2	0.715	1.64	6.37E-05	5.03E-04
206974_at	CXCR6	0.715	1.64	9.55E-06	1.10E-04
231562_at	APOC2	0.715	1.64	7.51E-07	1.42E-05
224456_s_at	FOXD2-AS1	0.715	1.64	3.20E-04	1.83E-03
226582_at	LOC400043	0.714	1.64	8.45E-05	6.34E-04
228338_at	C11orf93	0.714	1.64	1.77E-04	1.15E-03
217935_s_at	UQCC	0.714	1.64	8.02E-08	2.45E-06
38241_at	BTN3A3	0.714	1.64	1.89E-07	4.81E-06
215242_at	LOC100505991 /// PIGC	0.714	1.64	1.40E-06	2.37E-05
232792_at	TRIM69	0.714	1.64	6.36E-05	5.02E-04
244246_at	MIPOL1	0.714	1.64	8.05E-05	6.09E-04
209889_at	SEC31B	0.713	1.64	1.94E-08	7.76E-07
230670_at	IGSF10	0.713	1.64	9.49E-05	6.94E-04
229288_at	EPHA7	0.713	1.64	3.53E-04	1.98E-03
238135_at	AGTRAP	0.713	1.64	3.02E-05	2.78E-04
202499_s_at	SLC2A3	0.713	1.64	7.13E-04	3.44E-03
213566_at	RNASE6	0.713	1.64	1.27E-05	1.38E-04
231991_at	CCM2L	0.712	1.64	4.13E-04	2.24E-03
216250_s_at	LPXN	0.712	1.64	3.54E-08	1.27E-06
213706_at	GPD1	0.712	1.64	9.25E-03	2.69E-02
230206_at	DOCK5	0.712	1.64	4.22E-08	1.45E-06
221988_at	SMIM7	0.712	1.64	3.31E-05	2.98E-04
231345_s_at	DHRS12	0.712	1.64	6.47E-09	3.19E-07

213143_at	C2orf72	0.711	1.64	2.61E-03	9.75E-03
236320_at	CCDC17	0.711	1.64	4.45E-06	5.97E-05
233516_s_at	SPAG17	0.711	1.64	1.65E-03	6.73E-03
229608_at	FAM212B	0.71	1.64	5.46E-09	2.83E-07
206518_s_at	RGS9	0.71	1.64	2.97E-07	6.90E-06
210194_at	PLA2R1	0.71	1.64	1.59E-05	1.65E-04
231062_at	DOCK9-AS2	0.71	1.64	2.08E-04	1.30E-03
216512_s_at	DCT	0.71	1.64	2.32E-03	8.85E-03
204022_at	WWP2	0.71	1.64	7.95E-07	1.49E-05
244704_at	NFYB	0.71	1.64	2.43E-06	3.68E-05
226814_at	ADAMTS9	0.71	1.64	5.65E-05	4.57E-04
221218_s_at	TPK1	0.709	1.64	7.36E-08	2.30E-06
204773_at	IL11RA	0.709	1.64	6.84E-10	5.12E-08
202269_x_at	GBP1	0.709	1.63	4.52E-05	3.83E-04
210325_at	CD1A	0.709	1.63	1.92E-05	1.94E-04
231430_at	FAM181B	0.709	1.63	2.85E-05	2.64E-04
1565737_at	SCTR	0.709	1.63	7.63E-04	3.63E-03
243446_at	AJUBA	0.708	1.63	1.83E-04	1.18E-03
204639_at	ADA	0.708	1.63	1.77E-04	1.15E-03
205307_s_at	KMO	0.708	1.63	1.90E-05	1.92E-04
217418_x_at	MS4A1	0.708	1.63	1.13E-03	4.97E-03
1552501_a_at	GPBAR1	0.708	1.63	1.49E-04	1.00E-03
238121_at	GK5	0.708	1.63	1.89E-05	1.91E-04
204468_s_at	TIE1	0.708	1.63	1.79E-06	2.87E-05
230012_at	LINC00324	0.708	1.63	2.00E-05	2.00E-04
211470_s_at	SULT1C2	0.708	1.63	1.52E-02	4.01E-02
205839_s_at	BZRAP1	0.708	1.63	7.04E-08	2.23E-06
233510_s_at	PARVG	0.708	1.63	9.37E-05	6.87E-04
207992_s_at	AMPD3	0.708	1.63	5.78E-05	4.64E-04
229175_at	SMYD4	0.707	1.63	3.79E-07	8.25E-06
218760_at	COQ6	0.707	1.63	4.56E-07	9.50E-06
211303_x_at	FOLH1B	0.706	1.63	1.02E-04	7.33E-04
230631_s_at	IL10RB-AS1	0.705	1.63	1.18E-03	5.17E-03
221924_at	ZMIZ2	0.705	1.63	5.51E-06	7.08E-05
205893_at	NLGN1	0.705	1.63	3.37E-07	7.54E-06

235290_at	ZNF782	0.705	1.63	1.14E-03	5.02E-03
225545_at	EEF2K /// LOC101060570	0.705	1.63	3.74E-08	1.33E-06
231635_x_at	RNF31	0.704	1.63	1.69E-06	2.74E-05
228186_s_at	RSPO3	0.704	1.63	1.50E-03	6.23E-03
209699_x_at	AKR1C2 /// LOC101060798	0.704	1.63	2.30E-05	2.23E-04
244629_s_at	PDPK1	0.704	1.63	1.01E-03	4.55E-03
230351_at	FGF14-AS2	0.703	1.63	7.69E-07	1.45E-05
1569886_a_at	GLB1L3	0.703	1.63	3.28E-04	1.87E-03
229302_at	TMEM178A	0.703	1.63	3.03E-05	2.78E-04
210072_at	CCL19	0.703	1.63	8.77E-04	4.06E-03
210102_at	VWA5A	0.703	1.63	1.00E-04	7.26E-04
230234_at	FXN	0.703	1.63	6.33E-06	7.86E-05
228771_at	ADRBK2	0.702	1.63	1.09E-04	7.80E-04
227753_at	TMEM139	0.702	1.63	1.32E-03	5.64E-03
202665_s_at	WIPF1	0.702	1.63	1.89E-06	3.00E-05
1564707_x_at	GLS2	0.702	1.63	9.76E-05	7.09E-04
204106_at	TESK1	0.701	1.63	8.00E-06	9.53E-05
205236_x_at	SOD3	0.701	1.63	1.65E-03	6.71E-03
235670_at	STX11	0.701	1.63	9.07E-06	1.05E-04
202888_s_at	ANPEP	0.701	1.63	7.50E-08	2.33E-06
218821_at	LOC100652930 /// NPEPL1 /// STX16-NPEPL1	0.701	1.63	9.46E-07	1.71E-05
60471_at	RIN3	0.7	1.62	4.61E-09	2.43E-07
218638_s_at	LOC100130872 /// SPON2	0.7	1.62	6.74E-05	5.26E-04
203929_s_at	MAPT	0.7	1.62	1.07E-05	1.21E-04
1557236_at	APOL6	0.7	1.62	3.33E-05	2.99E-04
229211_at	DUSP28	0.7	1.62	4.90E-08	1.65E-06
223995_at	SLC12A9	0.699	1.62	1.99E-04	1.26E-03
221024_s_at	SLC2A10	0.699	1.62	2.93E-05	2.71E-04
52975_at	MVB12B	0.699	1.62	6.23E-05	4.94E-04
226804_at	FAM20A	0.699	1.62	2.98E-06	4.34E-05
1552509_a_at	CD300LG	0.698	1.62	1.31E-02	3.57E-02
228984_at	CARNS1	0.698	1.62	2.64E-07	6.27E-06
1431_at	CYP2E1	0.698	1.62	1.94E-05	1.95E-04
223636_at	ZMYND12	0.698	1.62	7.77E-05	5.91E-04
205100_at	GFPT2	0.69	1.62	1.44E-06	2.42E-05

		8			
1558552_s_at	C3orf55	0.697	1.62	8.99E-03	2.64E-02
232513_x_at	FAM209B	0.697	1.62	9.03E-11	9.48E-09
232001_at	PRKCQ-AS1	0.697	1.62	6.18E-03	1.95E-02
212451_at	SECISBP2L	0.697	1.62	1.32E-04	9.11E-04
232424_at	PRDM16	0.696	1.62	3.52E-03	1.24E-02
222123_s_at	HIF3A	0.696	1.62	2.21E-04	1.37E-03
209791_at	PADI2	0.696	1.62	4.15E-04	2.25E-03
206747_at	GPRIN2	0.696	1.62	8.59E-04	3.99E-03
238253_at	WDR72	0.695	1.62	1.27E-03	5.47E-03
229326_at	TNFSF13	0.695	1.62	2.04E-04	1.28E-03
205330_at	MN1	0.695	1.62	1.33E-05	1.43E-04
204368_at	SLCO2A1	0.694	1.62	2.41E-06	3.66E-05
210861_s_at	WISP3	0.694	1.62	8.96E-03	2.63E-02
1555912_at	ST7-AS1	0.694	1.62	1.45E-07	3.93E-06
235199_at	RNF125	0.694	1.62	3.70E-09	2.04E-07
205447_s_at	MAP3K12	0.694	1.62	4.40E-05	3.74E-04
1558971_at	THEMIS	0.693	1.62	6.33E-06	7.86E-05
222108_at	AMIGO2	0.693	1.62	3.95E-03	1.36E-02
222171_s_at	PKNOX2	0.693	1.62	1.01E-06	1.80E-05
213750_at	RSL1D1	0.692	1.62	8.52E-07	1.57E-05
219461_at	PAK6	0.692	1.62	8.86E-05	6.58E-04
229357_at	ADAMTS5	0.692	1.62	2.85E-04	1.67E-03
235867_at	GSTM3	0.692	1.62	2.74E-03	1.01E-02
207238_s_at	PTPRC	0.692	1.62	6.12E-04	3.06E-03
242204_at	WFDC5	0.692	1.62	2.40E-03	9.08E-03
244008_at	PARP8	0.692	1.62	1.07E-06	1.89E-05
234305_s_at	GSDMC	0.692	1.62	3.85E-03	1.33E-02
215559_at	ABCC6	0.691	1.61	5.65E-04	2.86E-03
229199_at	SCN9A	0.691	1.61	9.79E-05	7.11E-04
227251_at	DCAF5	0.691	1.61	4.74E-06	6.28E-05
204500_s_at	AGTPBP1	0.691	1.61	3.07E-06	4.43E-05
1562644_at	MTHFD2L	0.69	1.61	3.02E-04	1.75E-03
222134_at	DDO	0.69	1.61	8.82E-04	4.08E-03

244660_at	ELAVL1	0.69	1.61	7.40E-05	5.68E-04
220784_s_at	UTS2	0.69	1.61	1.05E-02	2.98E-02
235666_at	ITGA8	0.689	1.61	3.99E-03	1.37E-02
203661_s_at	TMOD1	0.689	1.61	3.62E-06	5.06E-05
219359_at	ATHL1	0.689	1.61	6.55E-04	3.22E-03
229824_at	SHC3	0.689	1.61	7.26E-08	2.27E-06
36129_at	SGSM2	0.689	1.61	2.04E-04	1.28E-03
1553635_s_at	TCTEX1D1	0.689	1.61	2.55E-04	1.53E-03
237395_at	CYP4Z1	0.689	1.61	1.41E-02	3.78E-02
228032_s_at	DENND1B	0.688	1.61	6.27E-11	6.81E-09
217371_s_at	IL15	0.687	1.61	1.27E-05	1.38E-04
212196_at	IL6ST	0.687	1.61	2.47E-06	3.74E-05
213124_at	ZNF473	0.687	1.61	1.66E-05	1.72E-04
213589_s_at	B3GNTL1	0.687	1.61	1.96E-04	1.24E-03
220848_x_at	OBP2A	0.687	1.61	6.24E-06	7.79E-05
206838_at	TBX19	0.686	1.61	6.39E-06	7.92E-05
226618_at	UBE2QL1	0.686	1.61	2.33E-05	2.27E-04
228548_at	RAP1A	0.686	1.61	1.26E-05	1.37E-04
217076_s_at	HOXD3	0.686	1.61	1.40E-04	9.54E-04
242033_at	RNF180	0.686	1.61	3.53E-04	1.98E-03
218164_at	SPATA20	0.686	1.61	1.05E-03	4.69E-03
239767_at	LRRC7	0.685	1.61	4.02E-04	2.19E-03
214070_s_at	ATP10B	0.685	1.61	4.77E-04	2.51E-03
223500_at	CPLX1	0.685	1.61	4.27E-05	3.66E-04
209892_at	FUT4	0.684	1.61	1.88E-05	1.91E-04
229954_at	CHDH	0.684	1.61	3.35E-04	1.90E-03
227145_at	LOXL4	0.684	1.61	1.13E-05	1.26E-04
214308_s_at	HGD	0.683	1.61	1.62E-03	6.62E-03
203951_at	CNN1	0.683	1.61	4.54E-03	1.52E-02
232073_at	PPFIA2	0.683	1.61	4.14E-06	5.64E-05
235749_at	UGGT2	0.683	1.61	3.08E-05	2.81E-04
221921_s_at	CADM3	0.683	1.61	8.39E-05	6.30E-04
236834_at	SCFD2	0.683	1.61	2.26E-05	2.21E-04
224856_at	FKBP5	0.682	1.6	9.52E-04	4.34E-03

210874_s_at	NAT6	0.682	1.6	1.82E-07	4.67E-06
243076_x_at	GLI4	0.682	1.6	2.42E-07	5.87E-06
210375_at	PTGER3	0.682	1.6	3.57E-04	1.99E-03
217525_at	OLFML1	0.682	1.6	1.38E-04	9.45E-04
40569_at	MZF1	0.682	1.6	9.75E-04	4.43E-03
205247_at	NOTCH4	0.682	1.6	7.73E-04	3.67E-03
229722_at	LOC100270804	0.682	1.6	4.12E-09	2.23E-07
219947_at	CLEC4A	0.682	1.6	2.62E-06	3.92E-05
236108_at	EPG5	0.681	1.6	4.69E-04	2.48E-03
220112_at	ANKRD55	0.681	1.6	4.21E-05	3.62E-04
230593_at	GRIK3	0.681	1.6	5.45E-06	7.02E-05
239547_at	HS3ST6	0.681	1.6	1.58E-04	1.05E-03
240806_at	RPL15	0.681	1.6	1.54E-06	2.54E-05
1554004_a_at	ARHGEF28	0.681	1.6	7.87E-05	5.97E-04
238862_at	MFSD4	0.681	1.6	5.17E-07	1.05E-05
214846_s_at	ALPK3	0.681	1.6	6.84E-03	2.12E-02
206606_at	LIPC	0.68	1.6	1.75E-04	1.14E-03
228426_at	CLEC2D	0.68	1.6	1.72E-03	6.97E-03
236915_at	C4orf47	0.68	1.6	3.79E-03	1.32E-02
58780_s_at	ARHGEF40	0.68	1.6	2.20E-05	2.16E-04
229395_at	STX4	0.68	1.6	1.64E-05	1.70E-04
223805_at	OSBPL6	0.68	1.6	2.57E-04	1.54E-03
227072_at	RTTN	0.679	1.6	2.93E-08	1.10E-06
232523_at	MEGF10	0.679	1.6	9.83E-03	2.83E-02
214285_at	FABP3	0.679	1.6	5.06E-08	1.69E-06
1568752_s_at	RGS13	0.679	1.6	5.49E-03	1.78E-02
1552283_s_at	LOC100996904 /// ZDHHC11 /// ZDHHC11B	0.679	1.6	2.44E-03	9.20E-03
240429_at	ZNF546	0.679	1.6	2.00E-04	1.26E-03
209501_at	CDR2	0.678	1.6	4.01E-05	3.49E-04
228104_at	PLXNA4	0.678	1.6	5.12E-04	2.65E-03
236548_at	GIPC2	0.678	1.6	5.54E-04	2.83E-03
244578_at	LCP2	0.678	1.6	6.51E-04	3.21E-03
205495_s_at	GNLY	0.678	1.6	3.98E-05	3.47E-04
226207_at	RILPL1	0.677	1.6	1.24E-05	1.36E-04
204070_at	RARRES3	0.677	1.6	3.86E-07	8.38E-06

1560762_at	LOC285972	0.677	1.6	1.43E-03	6.02E-03
1569701_at	PER3	0.677	1.6	2.43E-05	2.34E-04
241437_s_at	EP400NL	0.677	1.6	3.94E-06	5.43E-05
219886_at	CEP97	0.677	1.6	1.19E-04	8.37E-04
226673_at	SH2D3C	0.676	1.6	2.69E-07	6.36E-06
205067_at	IL1B	0.676	1.6	6.30E-03	1.98E-02
35148_at	TJP3	0.676	1.6	1.04E-04	7.50E-04
218776_s_at	TMEM62	0.676	1.6	3.56E-11	4.28E-09
203579_s_at	SLC7A6	0.676	1.6	8.11E-08	2.47E-06
224701_at	PARP14	0.676	1.6	4.76E-08	1.61E-06
227733_at	TMEM63C	0.676	1.6	2.15E-03	8.35E-03
236132_at	TLN1	0.676	1.6	5.97E-06	7.54E-05
209716_at	CSF1	0.675	1.6	8.59E-08	2.58E-06
230433_at	LOC729970	0.675	1.6	2.38E-06	3.62E-05
233621_s_at	ARHGEF12	0.675	1.6	5.00E-04	2.60E-03
204294_at	AMT	0.675	1.6	3.80E-04	2.10E-03
226448_at	FAM89A /// MIR1182	0.675	1.6	1.92E-04	1.22E-03
218924_s_at	CTBS	0.675	1.6	5.57E-06	7.13E-05
205203_at	PLD1	0.675	1.6	1.15E-04	8.11E-04
205399_at	DCLK1	0.675	1.6	1.71E-05	1.76E-04
219723_x_at	AGPAT3	0.675	1.6	3.38E-04	1.91E-03
227966_s_at	CCDC74A /// CCDC74B	0.674	1.6	2.28E-06	3.50E-05
228504_at	SCN7A	0.674	1.6	5.35E-05	4.37E-04
236541_at	ARSA	0.674	1.6	7.33E-07	1.40E-05
210576_at	CYP4F8	0.674	1.6	1.00E-02	2.87E-02
38149_at	ARHGAP25	0.674	1.6	2.23E-07	5.53E-06
224964_s_at	GNG2	0.674	1.6	1.27E-05	1.38E-04
238453_at	FGFBP3	0.674	1.59	2.68E-06	4.00E-05
227002_at	FAM78A	0.673	1.59	1.89E-07	4.81E-06
226777_at	ADAM12 /// ADAM12-OT1	0.673	1.59	2.09E-03	8.13E-03
1555476_at	IREB2	0.673	1.59	4.39E-03	1.48E-02
1569107_s_at	ZFP69	0.673	1.59	2.00E-04	1.26E-03
226072_at	FUK	0.673	1.59	3.20E-06	4.59E-05

243481_at	RHOJ	0.672	1.59	5.28E-05	4.34E-04
228489_at	TM4SF18	0.672	1.59	1.13E-04	8.03E-04
227081_at	DNALI1	0.672	1.59	1.55E-05	1.63E-04
238977_at	MCM6	0.672	1.59	3.92E-05	3.42E-04
1556639_at	LOC100996455	0.672	1.59	3.01E-05	2.77E-04
243602_at	MGC40069	0.671	1.59	2.13E-04	1.32E-03
215119_at	MYO16	0.671	1.59	1.83E-02	4.66E-02
226517_at	BCAT1	0.671	1.59	1.92E-05	1.93E-04
1556224_a_at	LOC155060 /// ZNF783	0.671	1.59	6.53E-06	8.05E-05
1559716_at	INO80C	0.671	1.59	3.15E-05	2.87E-04
238663_x_at	GRIA4	0.671	1.59	2.50E-04	1.51E-03
235306_at	GIMAP8	0.671	1.59	2.84E-06	4.17E-05
37384_at	PPM1F	0.671	1.59	2.61E-05	2.48E-04
227946_at	OSBPL7	0.671	1.59	2.01E-04	1.27E-03
236468_at	TMEM132B	0.671	1.59	1.09E-04	7.81E-04
212159_x_at	AP2A2	0.67	1.59	3.55E-07	7.86E-06
238484_s_at	SSBP2	0.67	1.59	1.01E-05	1.16E-04
239201_at	CDK15	0.67	1.59	1.96E-04	1.24E-03
1569453_a_at	LOC692247	0.67	1.59	9.50E-03	2.75E-02
226388_at	TCEA3	0.67	1.59	7.40E-08	2.31E-06
210017_at	MALT1	0.67	1.59	3.67E-07	8.05E-06
221875_x_at	HLA-F	0.67	1.59	2.65E-05	2.51E-04
238669_at	PTGS1	0.67	1.59	2.78E-06	4.11E-05
231783_at	CHRM1	0.67	1.59	4.67E-07	9.68E-06
231372_at	SLC25A48	0.67	1.59	4.79E-04	2.52E-03
229625_at	GBP5	0.67	1.59	8.52E-05	6.38E-04
241368_at	PLIN5	0.67	1.59	3.05E-05	2.79E-04
204231_s_at	FAAH	0.67	1.59	4.80E-05	4.02E-04
223809_at	RGS18	0.669	1.59	8.05E-03	2.42E-02
235352_at	MR1	0.669	1.59	1.62E-06	2.64E-05
223845_at	VSIG10	0.669	1.59	1.24E-07	3.47E-06
214099_s_at	LOC100996724 /// LOC100996761 /// LOC101060291 /// LOC101060344 /// LOC101060353 /// LOC101060463 /// LOC101060582 /// LOC728802 /// PDE4DIP	0.669	1.59	4.92E-03	1.63E-02
228482_at	CDRT4 /// TVP23C-CDRT4	0.668	1.59	6.05E-06	7.62E-05
238458_at	MICU3	0.668	1.59	6.48E-04	3.20E-03
238332_at	ANKRD29	0.668	1.59	1.02E-03	4.61E-03
206560_s_at	MIA	0.668	1.59	7.09E-04	3.43E-03

226766_at	ROBO2	0.668	1.59	2.19E-04	1.36E-03
225847_at	NCEH1	0.668	1.59	4.80E-06	6.34E-05
34210_at	CD52	0.667	1.59	5.86E-04	2.95E-03
212611_at	DTX4	0.667	1.59	2.86E-04	1.68E-03
215192_at	PMS2P4	0.667	1.59	2.70E-03	1.00E-02
205794_s_at	NOVA1	0.667	1.59	3.17E-03	1.14E-02
1556579_s_at	IGSF10	0.666	1.59	9.26E-05	6.81E-04
213422_s_at	MXRA8	0.666	1.59	3.11E-05	2.84E-04
223549_s_at	ESPN	0.666	1.59	1.24E-04	8.68E-04
229256_at	PGM2L1	0.666	1.59	7.87E-05	5.97E-04
232102_at	METTL6	0.666	1.59	7.63E-05	5.83E-04
203119_at	CCDC86	0.666	1.59	3.43E-06	4.86E-05
1563549_a_at	ANO8	0.665	1.59	1.57E-05	1.64E-04
224414_s_at	CARD6	0.665	1.59	5.72E-04	2.89E-03
203814_s_at	NQO2	0.665	1.59	9.20E-05	6.78E-04
207900_at	CCL17	0.665	1.59	1.19E-03	5.21E-03
235975_at	MTO1	0.665	1.59	2.81E-05	2.62E-04
205212_s_at	ACAP1	0.665	1.59	4.70E-07	9.72E-06
236618_at	MROH8	0.665	1.59	1.37E-04	9.38E-04
230104_s_at	TPPP	0.664	1.58	7.04E-04	3.41E-03
213727_x_at	MPPE1	0.664	1.58	2.94E-08	1.10E-06
235677_at	SRR	0.664	1.58	1.25E-06	2.15E-05
227240_at	NGEF	0.664	1.58	8.31E-07	1.54E-05
235458_at	HAVCR2	0.663	1.58	4.73E-05	3.97E-04
231577_s_at	GBP1	0.663	1.58	1.18E-03	5.16E-03
203108_at	GPRC5A	0.663	1.58	5.36E-03	1.75E-02
220034_at	IRAK3	0.663	1.58	7.80E-07	1.47E-05
210548_at	CCL23	0.663	1.58	4.07E-04	2.21E-03
224925_at	PREX1	0.663	1.58	8.33E-06	9.86E-05
218717_s_at	LEPREL1	0.663	1.58	4.02E-06	5.51E-05
231026_at	EFHC1	0.662	1.58	5.55E-04	2.83E-03
201802_at	SLC29A1	0.662	1.58	1.20E-08	5.23E-07
240512_x_at	KCTD4	0.662	1.58	1.78E-03	7.16E-03

238793_at	TIGD7	0.662	1.58	2.32E-04	1.42E-03
205685_at	CD86	0.662	1.58	3.52E-08	1.27E-06
209765_at	ADAM19	0.662	1.58	2.24E-04	1.38E-03
226984_at	FGD5	0.662	1.58	2.78E-05	2.60E-04
228184_at	DISP1	0.661	1.58	2.41E-07	5.85E-06
204154_at	CDO1	0.661	1.58	1.67E-02	4.33E-02
220178_at	MFSD12	0.661	1.58	2.95E-04	1.72E-03
222803_at	PRTFDC1	0.661	1.58	4.60E-05	3.88E-04
231947_at	MYCT1	0.661	1.58	2.48E-05	2.38E-04
223748_at	SLC4A11	0.66	1.58	1.69E-05	1.74E-04
223859_at	EPB41L4B	0.66	1.58	4.86E-03	1.61E-02
204014_at	DUSP4	0.66	1.58	8.60E-04	3.99E-03
235597_s_at	RGPD1 /// RGPD2	0.66	1.58	1.14E-02	3.18E-02
230908_at	TACR1	0.66	1.58	1.46E-05	1.54E-04
243531_at	ORAOV1	0.66	1.58	4.94E-05	4.11E-04
211126_s_at	CSRP2	0.66	1.58	2.79E-05	2.61E-04
209123_at	QDPR	0.659	1.58	2.75E-10	2.39E-08
218692_at	SYBU	0.659	1.58	4.61E-06	6.15E-05
234436_x_at	OBP2A	0.659	1.58	1.41E-05	1.50E-04
204629_at	PARVB	0.659	1.58	2.61E-04	1.56E-03
228113_at	RAB37	0.659	1.58	5.36E-04	2.75E-03
1569788_at	ST8SIA1	0.659	1.58	2.48E-03	9.32E-03
232284_at	PSMD6-AS2	0.659	1.58	3.77E-04	2.08E-03
222857_s_at	KCNMB4	0.659	1.58	7.98E-06	9.51E-05
206772_at	PTH2R	0.658	1.58	7.00E-04	3.40E-03
203936_s_at	MMP9	0.658	1.58	8.50E-04	3.96E-03
230836_at	ST8SIA4	0.658	1.58	3.39E-05	3.03E-04
244360_at	FBXL17	0.658	1.58	3.66E-07	8.05E-06
213185_at	KIAA0556	0.657	1.58	9.23E-07	1.67E-05
214033_at	ABCC6	0.657	1.58	4.84E-04	2.54E-03
204437_s_at	FOLR1	0.657	1.58	1.80E-03	7.22E-03
228831_s_at	GNG7	0.657	1.58	6.90E-07	1.33E-05
210029_at	IDO1	0.657	1.58	4.60E-07	9.57E-06
1558177_at	TMEM229B	0.656	1.58	7.44E-07	1.41E-05
205226_at	PDGFRL	0.656	1.58	5.68E-04	2.88E-03

211795_s_at	FYB	0.655	1.57	1.31E-04	9.07E-04
207354_at	CCL16	0.655	1.57	4.89E-07	1.00E-05
33197_at	MYO7A	0.655	1.57	7.41E-05	5.68E-04
235256_s_at	GALM	0.655	1.57	8.74E-06	1.02E-04
203730_s_at	ZKSCAN5	0.655	1.57	1.32E-05	1.42E-04
235368_at	ADAMTS5	0.655	1.57	4.77E-05	4.00E-04
218272_at	TTC38	0.655	1.57	1.18E-04	8.28E-04
1558827_a_at	ZNF831	0.655	1.57	1.62E-03	6.61E-03
211324_s_at	RGPD3 /// RGPD4 /// RGPD5 /// RGPD6 /// RGPD8	0.655	1.57	2.21E-03	8.52E-03
1556199_a_at	RGS9BP	0.654	1.57	1.07E-03	4.77E-03
206373_at	ZIC1	0.654	1.57	5.50E-06	7.06E-05
227935_s_at	PCGF5	0.654	1.57	5.75E-06	7.32E-05
230980_x_at	MIR142	0.654	1.57	2.45E-05	2.36E-04
204890_s_at	LCK	0.654	1.57	8.01E-06	9.53E-05
223663_at	CCDC88B	0.654	1.57	1.52E-06	2.51E-05
212358_at	CLIP3	0.654	1.57	1.51E-05	1.59E-04
217276_x_at	SERHL2	0.654	1.57	2.95E-03	1.08E-02
1569225_a_at	SCML4	0.654	1.57	1.72E-04	1.12E-03
205841_at	JAK2	0.653	1.57	4.45E-05	3.78E-04
235496_at	HRCT1	0.653	1.57	4.41E-04	2.36E-03
1560434_x_at	CLTA	0.653	1.57	7.49E-05	5.73E-04
229233_at	NRG3	0.653	1.57	1.12E-06	1.96E-05
203710_at	ITPR1	0.653	1.57	4.01E-07	8.60E-06
218579_s_at	DHX35	0.653	1.57	1.42E-07	3.86E-06
224339_s_at	ANGPTL1	0.653	1.57	3.46E-05	3.08E-04
201704_at	ENTPD6	0.653	1.57	2.52E-05	2.41E-04
206695_x_at	ZNF43	0.653	1.57	1.25E-05	1.36E-04
219734_at	SIDT1	0.653	1.57	5.22E-05	4.30E-04
242625_at	RSAD2	0.652	1.57	2.53E-04	1.52E-03
223253_at	EPDR1	0.652	1.57	3.13E-03	1.13E-02
209381_x_at	SF3A2	0.652	1.57	6.49E-05	5.10E-04
1559072_a_at	ELFN2	0.652	1.57	3.56E-07	7.87E-06
211663_x_at	PTGDS	0.651	1.57	6.17E-06	7.72E-05

219136_s_at	LMF1	0.651	1.57	2.61E-04	1.56E-03
229169_at	TTC18	0.65	1.57	2.06E-04	1.29E-03
242674_at	EIF4E	0.65	1.57	1.14E-04	8.05E-04
227626_at	PAQR8	0.65	1.57	2.92E-05	2.70E-04
229426_at	COX5A	0.65	1.57	5.94E-06	7.51E-05
225025_at	IGSF8	0.65	1.57	9.51E-08	2.78E-06
209960_at	HGF	0.65	1.57	3.53E-03	1.24E-02
227591_at	SH3BP5-AS1	0.649	1.57	6.90E-06	8.43E-05
213464_at	SHC2	0.649	1.57	5.14E-05	4.24E-04
223840_s_at	SPATA9	0.649	1.57	7.56E-05	5.78E-04
206150_at	CD27	0.648	1.57	1.31E-05	1.41E-04
228194_s_at	SORCS1	0.648	1.57	3.59E-07	7.93E-06
238333_s_at	PAOX	0.648	1.57	1.05E-02	2.99E-02
228658_at	MIAT	0.648	1.57	1.34E-03	5.70E-03
205453_at	HOXB2	0.648	1.57	4.45E-04	2.38E-03
224929_at	TMEM173	0.648	1.57	9.25E-07	1.68E-05
229039_at	SYN2	0.647	1.57	1.65E-03	6.71E-03
232643_at	POLR2F	0.647	1.57	5.08E-06	6.64E-05
209525_at	HDGFRP3	0.647	1.57	3.73E-06	5.17E-05
205718_at	ITGB7	0.647	1.57	4.17E-07	8.86E-06
228956_at	UGT8	0.647	1.57	1.27E-03	5.48E-03
219921_s_at	DOCK5	0.646	1.56	1.18E-08	5.16E-07
227894_at	WDR90	0.646	1.56	2.63E-05	2.49E-04
205511_at	FLJ10038	0.646	1.56	9.02E-05	6.67E-04
212890_at	SLC38A10	0.646	1.56	1.27E-04	8.82E-04
1557895_at	FLJ35934	0.646	1.56	2.37E-04	1.45E-03
1553734_at	AK7	0.646	1.56	3.75E-05	3.30E-04
1556736_at	LOC100129858	0.645	1.56	5.46E-05	4.45E-04
211766_s_at	PNLIPRP2	0.645	1.56	2.64E-03	9.84E-03
235627_at	PFN4	0.645	1.56	1.19E-03	5.19E-03
239805_at	SLC13A2	0.644	1.56	2.38E-03	9.03E-03
242269_at	FLJ42875	0.644	1.56	1.23E-06	2.13E-05
221207_s_at	NBEA	0.644	1.56	3.40E-04	1.92E-03
206932_at	CH25H	0.644	1.56	3.35E-03	1.19E-02
225168_at	FRMD4A	0.644	1.56	2.34E-07	5.71E-06

216350_s_at	ZNF10	0.644	1.56	1.26E-04	8.77E-04
1561336_at	DNASE1L3	0.643	1.56	5.24E-03	1.71E-02
236401_at	GIMAP1	0.643	1.56	1.15E-05	1.28E-04
236222_at	MAATS1	0.643	1.56	2.02E-04	1.27E-03
206685_at	HCG4	0.643	1.56	4.83E-03	1.60E-02
213488_at	SNED1	0.643	1.56	5.33E-06	6.89E-05
230869_at	FAM155A	0.643	1.56	5.79E-03	1.86E-02
235885_at	P2RY12	0.643	1.56	9.29E-06	1.08E-04
227944_at	PTPN3	0.642	1.56	3.42E-07	7.61E-06
210401_at	P2RX1	0.642	1.56	6.37E-05	5.03E-04
232155_at	RNF213	0.642	1.56	1.86E-04	1.19E-03
206819_at	LOC727983 /// POM121L9P	0.642	1.56	8.03E-03	2.41E-02
206844_at	FBP2	0.642	1.56	7.99E-03	2.40E-02
232676_x_at	MYEF2	0.642	1.56	7.65E-06	9.18E-05
227285_at	C1orf51	0.642	1.56	8.47E-04	3.95E-03
241694_at	PKHD1	0.642	1.56	8.09E-04	3.80E-03
243456_at	ZNF214	0.642	1.56	1.25E-03	5.43E-03
218986_s_at	DDX60	0.642	1.56	4.89E-08	1.64E-06
228863_at	PCDH17	0.642	1.56	3.26E-03	1.17E-02
229947_at	PI15	0.642	1.56	2.59E-04	1.55E-03
226735_at	TAPT1	0.642	1.56	1.72E-08	6.98E-07
218870_at	ARHGAP15	0.641	1.56	5.34E-05	4.37E-04
213010_at	PRKCDBP	0.641	1.56	1.67E-04	1.10E-03
227596_at	FLJ37453	0.641	1.56	9.73E-09	4.42E-07
242247_at	METTL15	0.641	1.56	3.52E-06	4.95E-05
209505_at	NR2F1	0.641	1.56	3.59E-04	2.00E-03
209871_s_at	APBA2	0.641	1.56	2.51E-05	2.40E-04
220371_s_at	SLC12A9	0.64	1.56	2.79E-06	4.11E-05
236430_at	TMED6	0.64	1.56	1.56E-03	6.44E-03
227657_at	RNF150	0.64	1.56	2.69E-03	9.99E-03
202202_s_at	LAMA4	0.64	1.56	4.43E-06	5.95E-05
202847_at	PCK2	0.64	1.56	2.35E-04	1.44E-03
241607_at	LOC730102	0.64	1.56	4.73E-06	6.27E-05
220114_s_at	STAB2	0.64	1.56	4.55E-05	3.85E-04
231283_at	MGAT4A	0.64	1.56	5.49E-04	2.81E-03

1557161_at	LOC100132735	0.64	1.56	7.33E-03	2.24E-02
209166_s_at	MAN2B1	0.64	1.56	1.44E-06	2.42E-05
205013_s_at	ADORA2A /// SPECC1L-ADORA2A	0.64	1.56	4.26E-08	1.46E-06
229802_at	WISP1	0.63 9	1.56	1.53E-03	6.35E-03
237745_at	TSC22D1-AS1	0.63 9	1.56	1.54E-04	1.03E-03
1553037_a_at	SYN2	0.63 9	1.56	3.31E-04	1.88E-03
235279_at	PARVA	0.63 9	1.56	1.24E-05	1.36E-04
205944_s_at	CLTCL1	0.63 9	1.56	1.95E-07	4.95E-06
228384_s_at	PYROXD2	0.63 8	1.56	1.27E-03	5.47E-03
223608_at	EFCAB2	0.63 8	1.56	1.31E-04	9.06E-04
213900_at	FAM189A2	0.63 8	1.56	1.63E-03	6.66E-03
236644_at	RNF180	0.63 8	1.56	3.72E-04	2.06E-03
203040_s_at	HMBS	0.63 8	1.56	9.51E-04	4.34E-03
228305_at	ZNF565	0.63 8	1.56	4.08E-06	5.58E-05
210549_s_at	CCL23	0.63 7	1.56	5.54E-03	1.79E-02
237408_at	DCUN1D1	0.63 7	1.56	6.17E-05	4.90E-04
205380_at	PDZK1	0.63 7	1.56	1.43E-03	6.02E-03
242470_at	EID2B	0.63 7	1.56	1.58E-05	1.65E-04
228082_at	CLMP	0.63 7	1.55	1.52E-02	4.02E-02
210653_s_at	BCKDHB	0.63 7	1.55	5.65E-03	1.82E-02
206125_s_at	KLK8	0.63 7	1.55	1.84E-02	4.68E-02
219161_s_at	CKLF /// CKLF-CMTM1	0.63 6	1.55	6.93E-06	8.46E-05
209799_at	PRKAA1	0.63 6	1.55	1.57E-09	9.96E-08
204788_s_at	PPOX	0.63 6	1.55	3.79E-07	8.26E-06
229308_at	ANKRD29	0.63 6	1.55	8.67E-03	2.56E-02
219587_at	TTC12	0.63 6	1.55	6.59E-06	8.11E-05
1553612_at	ZNF354B	0.63 5	1.55	2.09E-03	8.13E-03
214313_s_at	EIF5B	0.63 5	1.55	6.98E-04	3.39E-03
219700_at	PLXDC1	0.63 5	1.55	4.24E-05	3.64E-04
233540_s_at	CDK5RAP2	0.63 5	1.55	8.51E-06	1.00E-04
209858_x_at	MPPE1	0.63 5	1.55	1.61E-06	2.63E-05
230536_at	PBX4	0.63 5	1.55	1.53E-06	2.53E-05
215108_x_at	TOX3	0.63 5	1.55	6.40E-03	2.01E-02
227649_s_at	SRGAP2 /// SRGAP2D	0.63 4	1.55	4.63E-06	6.18E-05

219728_at	MYOT	0.634	1.55	2.08E-03	8.12E-03
228341_at	NUDT16	0.634	1.55	4.37E-06	5.90E-05
235930_at	KCNMB4	0.634	1.55	1.54E-06	2.55E-05
243843_at	N4BP2L1	0.634	1.55	3.29E-04	1.87E-03
218601_at	URGCP	0.634	1.55	6.18E-05	4.90E-04
202104_s_at	SPG7	0.634	1.55	1.08E-06	1.90E-05
238924_at	BMS1P1 /// BMS1P5 /// LOC100996797	0.634	1.55	3.07E-04	1.77E-03
236347_at	MMAA	0.633	1.55	4.73E-06	6.27E-05
212681_at	EPB41L3	0.633	1.55	1.48E-07	3.99E-06
205908_s_at	OMD	0.633	1.55	2.47E-04	1.49E-03
230822_at	TMEM61	0.633	1.55	1.14E-03	5.02E-03
221042_s_at	CLMN	0.633	1.55	3.84E-04	2.11E-03
230436_s_at	CENPV	0.633	1.55	8.37E-06	9.88E-05
212780_at	SOS1	0.633	1.55	5.54E-06	7.10E-05
218211_s_at	MLPH	0.632	1.55	3.09E-05	2.82E-04
238670_at	RAD18	0.632	1.55	6.36E-08	2.05E-06
236817_at	ADAT2	0.632	1.55	8.11E-05	6.13E-04
213471_at	NPHP4	0.632	1.55	1.36E-06	2.31E-05
1554628_at	ZNF57	0.632	1.55	2.98E-06	4.35E-05
64064_at	GIMAP1-GIMAP5 /// GIMAP5	0.632	1.55	2.53E-04	1.52E-03
219692_at	KREMEN2	0.632	1.55	4.86E-06	6.41E-05
244313_at	CR1	0.631	1.55	4.10E-04	2.23E-03
229062_at	ARL9	0.631	1.55	2.32E-08	9.05E-07
219667_s_at	BANK1	0.631	1.55	9.96E-03	2.86E-02
229696_at	FECH	0.631	1.55	1.10E-04	7.82E-04
215764_x_at	AP2A2	0.631	1.55	2.29E-07	5.63E-06
237493_at	IL22RA2	0.631	1.55	2.96E-06	4.32E-05
1559021_at	C3orf52	0.631	1.55	8.28E-04	3.88E-03
235981_at	C8orf22	0.63	1.55	1.90E-03	7.53E-03
229975_at	BMPR1B	0.63	1.55	4.16E-03	1.42E-02
1553392_at	EFCAB3	0.63	1.55	1.33E-03	5.69E-03
205114_s_at	CCL3 /// CCL3L1 /// CCL3L3 /// LOC101060267	0.63	1.55	1.19E-03	5.21E-03
204103_at	CCL4	0.63	1.55	1.20E-05	1.32E-04
219131_at	UBIAD1	0.63	1.55	1.19E-02	3.30E-02
228952_at	ENPP1	0.63	1.55	1.40E-06	2.36E-05

212705_x_at	PNPLA2	0.629	1.55	3.39E-05	3.03E-04
50221_at	TFEB	0.629	1.55	1.41E-07	3.83E-06
226096_at	FNDC5	0.629	1.55	3.36E-04	1.90E-03
235911_at	MFI2	0.629	1.55	6.58E-03	2.05E-02
40020_at	CELSR3	0.629	1.55	1.44E-06	2.42E-05
236083_at	BCL2L15	0.629	1.55	3.13E-05	2.85E-04
210356_x_at	MS4A1	0.629	1.55	5.62E-03	1.81E-02
210542_s_at	SLCO3A1	0.629	1.55	1.73E-05	1.78E-04
214060_at	SSBP1	0.629	1.55	3.28E-05	2.95E-04
216623_x_at	TOX3	0.629	1.55	8.06E-03	2.42E-02
239579_at	EPHX4	0.629	1.55	5.14E-04	2.66E-03
227807_at	PARP9	0.628	1.55	3.07E-07	7.07E-06
214344_at	LINC00950	0.628	1.55	5.97E-07	1.17E-05
227543_at	RNASEH2C	0.628	1.55	6.24E-03	1.97E-02
228395_at	GNL3	0.628	1.55	8.24E-07	1.53E-05
228555_at	CAMK2D	0.628	1.55	1.79E-08	7.24E-07
231263_at	ARMC12	0.628	1.55	1.47E-06	2.45E-05
232533_at	METTL8	0.628	1.55	8.90E-06	1.04E-04
227716_at	UBXN11	0.627	1.54	9.84E-04	4.47E-03
225067_at	ULK3	0.627	1.54	1.30E-06	2.22E-05
203260_at	HDDC2	0.627	1.54	5.86E-03	1.87E-02
229071_at	C17orf100	0.627	1.54	2.98E-04	1.73E-03
207338_s_at	ZNF200	0.627	1.54	9.76E-05	7.09E-04
215193_x_at	HLA-DRB1 /// HLA-DRB3 /// HLA-DRB4 /// LOC100507709 /// LOC100507714	0.627	1.54	1.29E-05	1.40E-04
227109_at	CYP2R1	0.627	1.54	4.49E-10	3.61E-08
213419_at	APBB2	0.627	1.54	9.95E-09	4.49E-07
207113_s_at	TNF	0.627	1.54	1.92E-05	1.94E-04
227594_at	ZMYM6	0.626	1.54	2.79E-07	6.55E-06
207961_x_at	MYH11	0.626	1.54	5.11E-03	1.68E-02
206837_at	ALX1	0.626	1.54	5.58E-05	4.53E-04
222868_s_at	IL18BP	0.625	1.54	2.18E-07	5.42E-06
214572_s_at	INSL3	0.625	1.54	4.50E-05	3.82E-04
1570505_at	ABCB4	0.625	1.54	1.41E-05	1.50E-04

209312_x_at	HLA-DRB1 /// HLA-DRB4 /// HLA-DRB5 /// LOC100507709 /// LOC100507714	0.625	1.54	7.97E-06	9.50E-05
230440_at	ZNF469	0.625	1.54	1.66E-05	1.72E-04
210847_x_at	TNFRSF25	0.625	1.54	1.05E-05	1.19E-04
1556062_at	RPP30	0.625	1.54	4.29E-06	5.81E-05
207075_at	NLRP3	0.625	1.54	2.10E-05	2.08E-04
227243_s_at	EBF3	0.625	1.54	4.40E-04	2.36E-03
212829_at	PIP4K2A	0.625	1.54	1.01E-10	1.03E-08
214438_at	HLX	0.624	1.54	2.53E-08	9.72E-07
210175_at	GCFC2	0.624	1.54	2.52E-04	1.52E-03
227526_at	CDON	0.624	1.54	3.19E-04	1.83E-03
217735_s_at	EIF2AK1	0.623	1.54	7.55E-04	3.60E-03
1552301_a_at	CORO6	0.623	1.54	1.10E-03	4.87E-03
212580_at	CAST	0.623	1.54	5.21E-04	2.68E-03
204296_at	DCTN1	0.623	1.54	2.78E-06	4.11E-05
63305_at	PKNOX2	0.623	1.54	5.24E-07	1.06E-05
202661_at	ITPR2	0.622	1.54	1.98E-05	1.99E-04
206197_at	NME5	0.622	1.54	9.16E-06	1.06E-04
235353_at	SEL1L3	0.622	1.54	2.39E-04	1.46E-03
227572_at	USP30	0.622	1.54	7.40E-10	5.43E-08
227396_at	PTPRJ	0.622	1.54	2.86E-05	2.65E-04
241450_at	RSPO1	0.622	1.54	8.18E-04	3.84E-03
229086_at	C1orf213	0.622	1.54	3.23E-06	4.63E-05
238615_at	ERLIN2	0.621	1.54	4.70E-05	3.95E-04
230744_at	FSTL1	0.621	1.54	1.11E-02	3.11E-02
233202_at	CNTNAP3	0.621	1.54	1.14E-04	8.05E-04
212757_s_at	CAMK2G	0.621	1.54	3.10E-08	1.15E-06
235238_at	SHC4	0.621	1.54	1.02E-03	4.60E-03
223126_s_at	C1orf21	0.621	1.54	1.13E-06	1.97E-05
236609_at	PMS1	0.621	1.54	1.10E-03	4.90E-03
219759_at	ERAP2	0.62	1.54	5.45E-03	1.77E-02
214058_at	MYCL1	0.62	1.54	1.19E-03	5.21E-03
227030_at	IKZF3	0.62	1.54	2.83E-06	4.16E-05
227234_at	LOC100132815	0.62	1.54	4.68E-04	2.48E-03
213248_at	LOC730101	0.62	1.54	3.86E-06	5.33E-05
232604_at	ZNF541	0.62	1.54	1.06E-04	7.63E-04

220468_at	ARL14	0.62	1.54	3.40E-05	3.04E-04
1557639_at	NFIA	0.619	1.54	3.12E-04	1.79E-03
231766_s_at	COL12A1	0.619	1.54	9.31E-03	2.71E-02
210314_x_at	TNFSF13	0.619	1.54	3.72E-04	2.06E-03
203914_x_at	HPGD	0.619	1.54	6.65E-07	1.29E-05
207240_s_at	LHCGR	0.619	1.54	5.81E-04	2.93E-03
219457_s_at	RIN3	0.618	1.54	2.93E-06	4.28E-05
204972_at	OAS2	0.618	1.53	2.45E-03	9.25E-03
223322_at	RASSF5	0.618	1.53	1.77E-06	2.84E-05
1556755_s_at	LOC286149	0.618	1.53	6.78E-05	5.28E-04
222920_s_at	TESPA1	0.618	1.53	9.68E-05	7.05E-04
225510_at	OAF	0.618	1.53	7.18E-06	8.71E-05
207496_at	MS4A2	0.618	1.53	1.39E-02	3.73E-02
204562_at	IRF4	0.618	1.53	5.14E-05	4.25E-04
237592_at	ZC2HC1B	0.617	1.53	1.32E-02	3.59E-02
222941_at	USP46	0.617	1.53	4.49E-07	9.37E-06
177_at	PLD1	0.617	1.53	5.05E-04	2.62E-03
206983_at	CCR6	0.617	1.53	1.69E-03	6.86E-03
238149_at	ZNF818P	0.617	1.53	6.82E-04	3.33E-03
209828_s_at	IL16	0.617	1.53	9.60E-05	7.01E-04
239548_at	NEGR1	0.617	1.53	6.84E-03	2.12E-02
233086_at	FAM209A	0.616	1.53	6.13E-07	1.20E-05
204882_at	ARHGAP25	0.616	1.53	1.35E-06	2.29E-05
221036_s_at	APH1B	0.616	1.53	1.68E-07	4.39E-06
204806_x_at	HLA-F	0.616	1.53	2.66E-05	2.52E-04
1553789_a_at	C21orf58	0.616	1.53	1.23E-04	8.63E-04
219597_s_at	DUOX1	0.615	1.53	3.37E-06	4.78E-05
220609_at	LOC202181	0.615	1.53	2.82E-05	2.63E-04
235587_at	LOC202781	0.615	1.53	2.41E-09	1.43E-07
1558523_at	FAM184A	0.615	1.53	1.47E-03	6.15E-03
232832_at	DKFZp434J0226	0.615	1.53	2.64E-04	1.57E-03
225386_s_at	HNRNPLL	0.615	1.53	1.31E-05	1.41E-04
229656_s_at	EML6	0.614	1.53	3.33E-03	1.19E-02
222730_s_at	ZDHHC2	0.61	1.53	9.62E-03	2.78E-02

		4			
219689_at	SEMA3G	0.614	1.53	2.59E-04	1.55E-03
203305_at	F13A1	0.614	1.53	1.48E-06	2.47E-05
215427_s_at	ZCCHC14	0.614	1.53	6.08E-07	1.19E-05
202308_at	SREBF1	0.614	1.53	4.21E-05	3.62E-04
235799_at	NSL1	0.614	1.53	4.90E-07	1.00E-05
219860_at	LY6G5C	0.614	1.53	1.71E-03	6.92E-03
218801_at	UGGT2	0.614	1.53	6.77E-05	5.28E-04
240259_at	FLRT2 /// LOC100506718	0.614	1.53	7.72E-05	5.89E-04
239279_at	LOC730102	0.613	1.53	6.01E-06	7.58E-05
236394_at	A2MP1	0.613	1.53	1.22E-05	1.34E-04
241399_at	FAM19A2	0.613	1.53	2.59E-05	2.46E-04
229153_at	SLC7A6OS	0.613	1.53	3.81E-05	3.34E-04
206693_at	IL7	0.612	1.53	1.57E-04	1.04E-03
220023_at	APOBR	0.612	1.53	2.06E-06	3.22E-05
1552803_a_at	STMN1	0.612	1.53	4.26E-03	1.45E-02
219117_s_at	FKBP11	0.612	1.53	5.44E-06	7.01E-05
229506_at	PPM1L	0.612	1.53	1.04E-05	1.18E-04
205678_at	AP3B2	0.611	1.53	3.78E-05	3.32E-04
204763_s_at	GNAO1	0.611	1.53	3.69E-04	2.05E-03
219572_at	CADPS2	0.611	1.53	6.73E-06	8.26E-05
216218_s_at	PLCL2	0.611	1.53	7.11E-06	8.64E-05
227593_at	FLJ37453	0.611	1.53	7.72E-08	2.38E-06
239282_at	CCDC41	0.611	1.53	7.20E-04	3.46E-03
214157_at	GNAS	0.611	1.53	6.76E-05	5.27E-04
218567_x_at	DPP3	0.611	1.53	9.12E-05	6.74E-04
232663_s_at	UBAP1L	0.611	1.53	5.31E-05	4.36E-04
218701_at	LACTB2	0.611	1.53	1.66E-05	1.72E-04
235561_at	TXNL1	0.611	1.53	3.34E-04	1.89E-03
227236_at	TSPAN2	0.611	1.53	2.51E-03	9.43E-03
1557567_a_at	LOC100507634	0.61	1.53	4.28E-04	2.31E-03
239799_at	LINC00476	0.61	1.53	8.67E-03	2.56E-02
1558345_a_at	LOC439911	0.61	1.53	1.10E-05	1.23E-04
214181_x_at	LST1	0.61	1.53	2.58E-05	2.45E-04
204713_s_at	F5	0.61	1.53	5.70E-03	1.83E-02

239261_s_at	CORIN	0.609	1.53	1.65E-05	1.70E-04
207857_at	LILRA2	0.609	1.53	2.26E-03	8.68E-03
231906_at	HOXD8	0.609	1.53	9.91E-05	7.18E-04
231357_at	CLEC12B	0.609	1.53	1.57E-04	1.04E-03
231582_at	CDHR3	0.609	1.53	1.97E-04	1.25E-03
239353_at	STK32A	0.609	1.52	5.74E-05	4.62E-04
228372_at	C10orf128	0.608	1.52	4.83E-05	4.04E-04
230045_at	CNTN2	0.608	1.52	4.74E-06	6.28E-05
207819_s_at	ABCB4	0.608	1.52	6.25E-06	7.79E-05
220979_s_at	ST6GALNAC5	0.608	1.52	6.98E-04	3.39E-03
1555759_a_at	CCL5	0.608	1.52	1.59E-03	6.54E-03
221013_s_at	APOL2	0.608	1.52	1.22E-05	1.34E-04
219243_at	GIMAP4	0.608	1.52	2.62E-04	1.56E-03
220042_x_at	HIVEP3	0.608	1.52	9.03E-04	4.16E-03
220346_at	MTHFD2L	0.607	1.52	1.04E-03	4.67E-03
202869_at	OAS1	0.607	1.52	1.02E-03	4.59E-03
205470_s_at	KLK11	0.607	1.52	9.97E-06	1.14E-04
207339_s_at	LTB	0.607	1.52	6.73E-06	8.26E-05
229572_at	ATP6V0A2	0.607	1.52	2.27E-07	5.59E-06
37586_at	ZNF142	0.607	1.52	1.37E-05	1.46E-04
232605_s_at	SPG20OS	0.606	1.52	1.62E-03	6.64E-03
235969_at	SCN8A	0.606	1.52	5.16E-04	2.66E-03
219583_s_at	SPATA7	0.606	1.52	2.67E-04	1.59E-03
38521_at	CD22	0.606	1.52	1.61E-03	6.60E-03
235884_at	LOC644794	0.606	1.52	3.76E-03	1.31E-02
217299_s_at	NBN	0.606	1.52	3.28E-05	2.96E-04
242525_at	SLC2A5	0.606	1.52	1.87E-02	4.74E-02
202912_at	ADM	0.606	1.52	2.35E-05	2.29E-04
204161_s_at	ENPP4	0.606	1.52	1.07E-04	7.69E-04
213249_at	FBXL7	0.606	1.52	8.32E-04	3.89E-03
239949_at	THNSL2	0.605	1.52	1.71E-02	4.42E-02
1556436_at	EXTL3-AS1	0.605	1.52	2.20E-03	8.48E-03
206766_at	ITGA10 /// LOC101060422	0.605	1.52	6.42E-04	3.18E-03

1558217_at	SLFN13	0.605	1.52	1.56E-07	4.15E-06
208894_at	HLA-DRA	0.605	1.52	4.94E-03	1.63E-02
213222_at	PLCB1	0.605	1.52	2.36E-04	1.44E-03
239426_at	SLC2A8	0.605	1.52	2.40E-03	9.08E-03
232317_at	PLXNA4	0.605	1.52	1.72E-04	1.12E-03
1558508_a_at	C1orf53	0.605	1.52	1.12E-04	7.98E-04
1554686_at	STAU2	0.605	1.52	2.20E-04	1.37E-03
202740_at	ABHD14A-ACY1 /// ACY1	0.604	1.52	3.08E-04	1.78E-03
209794_at	SRGAP3	0.604	1.52	1.32E-04	9.11E-04
1556001_at	LOC284939 /// MAPK8IP2	0.604	1.52	9.38E-08	2.75E-06
230329_s_at	NUDT6	0.604	1.52	1.70E-08	6.91E-07
213664_at	SLC1A1	0.604	1.52	1.54E-04	1.03E-03
244739_at	RDX	0.604	1.52	6.01E-04	3.01E-03
204904_at	GJA4	0.603	1.52	1.57E-04	1.04E-03
39705_at	SIN3B	0.603	1.52	3.10E-04	1.79E-03
209829_at	FAM65B	0.603	1.52	5.34E-05	4.37E-04
221059_s_at	COTL1	0.603	1.52	3.75E-04	2.08E-03
229994_at	NFIA	0.603	1.52	4.40E-08	1.50E-06
230043_at	MUC20	0.603	1.52	1.73E-02	4.46E-02
202659_at	PSMB10	0.603	1.52	4.08E-07	8.72E-06
213906_at	MYBL1	0.602	1.52	1.36E-05	1.46E-04
236562_at	ZNF439	0.602	1.52	5.63E-05	4.56E-04
204661_at	CD52	0.602	1.52	2.94E-03	1.08E-02
230573_at	SGK2	0.602	1.52	6.22E-03	1.96E-02
227397_at	TPM2	0.602	1.52	7.51E-03	2.29E-02
218430_s_at	RFX7	0.602	1.52	7.65E-04	3.64E-03
219007_at	NUP43	0.602	1.52	5.53E-06	7.10E-05
203938_s_at	TAF1C	0.602	1.52	3.74E-07	8.19E-06
203148_s_at	TRIM14	0.601	1.52	3.63E-06	5.07E-05
1556464_a_at	C2orf72	0.601	1.52	9.49E-07	1.71E-05
206133_at	XAF1	0.601	1.52	7.63E-04	3.63E-03
219279_at	DOCK10	0.601	1.52	1.11E-05	1.24E-04
219451_at	MSRB2	0.601	1.52	3.69E-06	5.13E-05

238078_at	SEC22A	0.601	1.52	2.75E-05	2.57E-04
211889_x_at	CEACAM1	0.601	1.52	5.73E-05	4.62E-04
230210_at	SUN1	0.601	1.52	6.96E-05	5.40E-04
203920_at	NR1H3	0.6	1.52	2.68E-04	1.59E-03
226634_at	METTL10	0.6	1.52	2.15E-05	2.12E-04
1563088_a_at	LOC284837	0.6	1.52	1.84E-03	7.33E-03
207509_s_at	LAIR2	0.6	1.52	5.31E-05	4.36E-04
219139_s_at	CROCCP3	0.6	1.52	4.04E-03	1.39E-02
236623_at	ATP1A1OS	0.6	1.52	2.18E-03	8.42E-03
37965_at	PARVB	0.6	1.52	1.80E-05	1.84E-04
240014_at	POLR2J4	0.599	1.52	1.00E-03	4.53E-03
242605_at	DCN	0.599	1.52	1.92E-04	1.23E-03
219014_at	PLAC8	0.599	1.52	8.31E-04	3.89E-03
220756_s_at	SLC52A1	0.599	1.52	1.62E-02	4.23E-02
215346_at	CD40	0.599	1.52	1.05E-07	2.99E-06
243951_at	ABCB1	0.599	1.51	1.05E-05	1.19E-04
214454_at	ADAMTS2	0.599	1.51	3.30E-04	1.88E-03
213392_at	IQCK	0.599	1.51	3.24E-05	2.93E-04
205590_at	RASGRP1	0.599	1.51	1.06E-06	1.88E-05
201859_at	SRGN	0.599	1.51	3.47E-06	4.89E-05
219559_at	SLC17A9	0.599	1.51	7.26E-07	1.39E-05
215239_x_at	ZNF273	0.599	1.51	4.36E-04	2.34E-03
218869_at	MLYCD	0.599	1.51	6.41E-06	7.93E-05
1552646_at	IL11RA	0.598	1.51	8.73E-07	1.60E-05
230721_at	C16orf52 /// LOC101060634	0.598	1.51	1.25E-04	8.70E-04
202709_at	FMOD	0.598	1.51	4.42E-04	2.37E-03
1558881_at	LINC00924	0.598	1.51	2.90E-04	1.70E-03
206950_at	SCN9A	0.598	1.51	4.74E-04	2.50E-03
236605_at	EIF3K	0.598	1.51	6.07E-05	4.83E-04
205083_at	AOX1	0.598	1.51	1.01E-04	7.31E-04
239441_at	ZNF780A	0.597	1.51	1.91E-03	7.58E-03
1552562_at	ZNF570	0.597	1.51	8.86E-04	4.10E-03
204382_at	NAT9	0.597	1.51	1.25E-05	1.37E-04
214470_at	KLRB1	0.597	1.51	4.10E-03	1.40E-02
224525_s_at	OLA1	0.597	1.51	4.84E-04	2.54E-03

214107_x_at	LOC101060440 /// LOC101060471 /// LOC101060522 /// LOC440434 /// NPEPPS /// TBC1D3	0.597	1.51	3.03E-03	1.10E-02
229604_at	CMAHP	0.597	1.51	4.58E-04	2.43E-03
224365_s_at	TIGD7	0.597	1.51	1.47E-04	9.88E-04
205160_at	PEX11A	0.597	1.51	3.78E-04	2.09E-03
239118_at	KCNA2	0.596	1.51	6.09E-03	1.93E-02
226938_at	DCAF4	0.596	1.51	7.41E-08	2.31E-06
203153_at	IFIT1	0.596	1.51	3.96E-04	2.16E-03
228618_at	PEAR1	0.596	1.51	4.17E-04	2.26E-03
204059_s_at	ME1	0.596	1.51	2.48E-05	2.38E-04
211434_s_at	CCRL2	0.596	1.51	3.15E-04	1.81E-03
223520_s_at	KIF13A	0.596	1.51	5.32E-05	4.36E-04
235695_at	INPP4A	0.596	1.51	5.60E-05	4.53E-04
208436_s_at	IRF7	0.596	1.51	4.83E-05	4.04E-04
228377_at	KLHL14	0.596	1.51	8.06E-04	3.79E-03
207156_at	HIST1H2AG /// HIST1H2AH /// HIST1H2AI /// HIST1H2AK /// HIST1H2AL /// HIST1H2AM	0.596	1.51	1.08E-02	3.04E-02
228213_at	H2AFJ	0.595	1.51	4.62E-07	9.60E-06
218972_at	TTC17	0.595	1.51	3.09E-07	7.09E-06
243534_at	CC2D2B /// LOC100652732	0.595	1.51	9.43E-04	4.31E-03
244345_at	CADM1	0.595	1.51	8.87E-04	4.10E-03
222052_at	C19orf54	0.595	1.51	2.36E-06	3.60E-05
204046_at	PLCB2	0.595	1.51	8.92E-07	1.63E-05
218610_s_at	CPPED1	0.595	1.51	3.53E-08	1.27E-06
212238_at	ASXL1	0.595	1.51	1.83E-05	1.86E-04
212913_at	MSH5 /// MSH5-SAPCD1 /// SAPCD1	0.594	1.51	6.55E-06	8.07E-05
235054_at	NUDT16	0.594	1.51	1.60E-06	2.62E-05
228891_at	SEMA4D	0.594	1.51	6.86E-04	3.34E-03
214427_at	NOP2	0.594	1.51	4.11E-05	3.56E-04
230725_at	ANKRD32	0.594	1.51	1.30E-06	2.22E-05
221810_at	RAB15	0.594	1.51	2.70E-05	2.54E-04
233072_at	NTNG2	0.594	1.51	1.47E-05	1.55E-04
1553973_a_at	SPINK6	0.594	1.51	3.64E-03	1.27E-02
218546_at	C1orf115	0.594	1.51	7.05E-06	8.60E-05
220418_at	UBASH3A	0.593	1.51	9.65E-06	1.11E-04

218945_at	METTL22	0.593	1.51	2.36E-05	2.29E-04
202390_s_at	HTT	0.593	1.51	3.16E-06	4.54E-05
206577_at	VIP	0.593	1.51	3.11E-03	1.13E-02
228521_s_at	MIA-RAB4B /// RAB4B	0.593	1.51	5.55E-05	4.50E-04
202902_s_at	CTSS	0.593	1.51	1.46E-04	9.84E-04
220170_at	FHL5	0.592	1.51	4.62E-04	2.45E-03
206919_at	ELK4	0.592	1.51	1.58E-04	1.05E-03
1557545_s_at	RNF165	0.592	1.51	5.12E-07	1.04E-05
239929_at	PM20D1	0.592	1.51	3.74E-03	1.30E-02
207183_at	GPR19	0.592	1.51	8.57E-04	3.98E-03
222919_at	TRDN	0.592	1.51	3.62E-04	2.02E-03
231369_at	ZNF333	0.592	1.51	2.53E-03	9.48E-03
243087_at	WDR63	0.592	1.51	3.23E-04	1.85E-03
201042_at	TGM2	0.591	1.51	7.19E-04	3.46E-03
207720_at	LOR	0.591	1.51	6.47E-04	3.19E-03
227679_at	HDAC11	0.591	1.51	6.99E-04	3.39E-03
224480_s_at	AGPAT9	0.59	1.51	3.14E-04	1.81E-03
226692_at	HYPK /// MIR1282 /// SERF2	0.59	1.51	8.21E-08	2.50E-06
223582_at	GPR98	0.59	1.51	1.39E-04	9.49E-04
232873_at	ZNF33A	0.59	1.51	1.91E-03	7.59E-03
204428_s_at	LCAT	0.59	1.51	2.19E-04	1.36E-03
238062_at	GPIHBP1	0.59	1.51	2.29E-03	8.79E-03
219622_at	RAB20	0.59	1.51	6.62E-05	5.18E-04
238036_at	SHE	0.59	1.51	1.84E-03	7.35E-03
219797_at	MGAT4A	0.59	1.51	2.53E-04	1.52E-03
225185_at	MRAS	0.59	1.51	1.47E-03	6.16E-03
55583_at	DOCK6	0.59	1.51	1.77E-05	1.81E-04
205051_s_at	KIT	0.59	1.5	3.58E-07	7.90E-06
213975_s_at	LYZ	0.59	1.5	2.59E-04	1.55E-03
227695_at	GLYATL1 /// LOC100287413	0.59	1.5	1.53E-06	2.54E-05
202182_at	KAT2A	0.589	1.5	8.60E-05	6.43E-04
239822_at	LOC100506795	0.589	1.5	3.57E-03	1.25E-02
218600_at	LIMD2	0.589	1.5	1.15E-06	1.99E-05
220048_at	EDAR	0.589	1.5	8.45E-04	3.94E-03
213651_at	INPP5J	0.589	1.5	8.05E-08	2.46E-06
204174_at	ALOX5AP	0.589	1.5	3.54E-04	1.98E-03
229925_at	SLC6A17	0.58	1.5	1.26E-06	2.16E-05

		9			
209277_at	TFPI2	0.589	1.5	3.72E-06	5.17E-05
230061_at	TM4SF18	0.589	1.5	2.71E-03	1.00E-02
219506_at	C1orf54	0.588	1.5	2.74E-06	4.07E-05
235518_at	SLC8A1	0.588	1.5	4.93E-04	2.57E-03
225524_at	ANTXR2	0.588	1.5	6.37E-04	3.16E-03
206682_at	CLEC10A	0.588	1.5	1.28E-04	8.88E-04
228875_at	FAM162B	0.587	1.5	6.60E-04	3.24E-03
214404_x_at	SPDEF	0.587	1.5	1.61E-03	6.59E-03
214582_at	PDE3B	0.587	1.5	8.78E-03	2.59E-02
230052_s_at	NFKBID	0.587	1.5	6.04E-05	4.81E-04
205527_s_at	GEMIN4	0.587	1.5	5.53E-06	7.10E-05
1556061_at	RPP30	0.586	1.5	8.82E-07	1.61E-05
232547_at	SRCIN1	0.586	1.5	7.90E-07	1.48E-05
212695_at	CRY2	0.586	1.5	4.34E-07	9.11E-06
238440_at	CLYBL	0.586	1.5	1.58E-05	1.65E-04
232012_at	CAPN1	0.586	1.5	3.31E-06	4.71E-05
202526_at	SMAD4	0.586	1.5	2.39E-04	1.45E-03
212880_at	WDR7	0.586	1.5	5.32E-06	6.89E-05
221816_s_at	PHF11	0.586	1.5	8.54E-11	9.04E-09
235350_at	C4orf19	0.586	1.5	1.09E-03	4.86E-03
235512_at	CDKL1	0.586	1.5	1.09E-04	7.77E-04
203094_at	MAD2L1BP	0.586	1.5	2.11E-06	3.29E-05
235735_at	TNFSF8	0.586	1.5	2.49E-05	2.39E-04
221045_s_at	PER3	0.585	1.5	3.48E-03	1.23E-02
1557608_a_at	LOC284080	0.585	1.5	1.52E-03	6.31E-03
235369_at	C14orf28	0.585	1.5	1.77E-06	2.85E-05
213869_x_at	THY1	0.585	1.5	4.72E-04	2.49E-03
217903_at	STRN4	0.585	1.5	2.61E-04	1.56E-03
201186_at	LRPAP1	0.585	1.5	3.24E-06	4.63E-05
1557914_s_at	ERVFH21-1	-0.585	-1.5	7.70E-09	3.68E-07
227891_s_at	TAF15	-0.585	-1.5	3.07E-06	4.44E-05
200998_s_at	CKAP4	-	-1.5	2.61E-07	6.23E-06

		0.586			
222708_s_at	STX17	-0.586	-1.5	3.45E-05	3.08E-04
219235_s_at	PHACTR4	-0.586	-1.5	1.02E-03	4.59E-03
222558_at	RPRD1A	-0.586	-1.5	5.01E-04	2.60E-03
1554557_at	ATP11B	-0.586	-1.5	1.77E-04	1.15E-03
214785_at	VPS13A	-0.586	-1.5	1.26E-05	1.37E-04
1559413_at	TCP11L2	-0.586	-1.5	1.40E-04	9.51E-04
225656_at	EFHC1	-0.587	-1.5	6.22E-03	1.96E-02
203276_at	LMNB1	-0.587	-1.5	1.63E-06	2.65E-05
239012_at	RNF144B	-0.587	-1.5	2.28E-04	1.40E-03
222559_s_at	RPRD1A	-0.587	-1.5	1.40E-03	5.94E-03
208649_s_at	VCP	-0.588	-1.5	1.56E-04	1.04E-03
207683_at	FOXN1	-0.588	-1.5	1.38E-09	9.04E-08
224008_s_at	KCNK7	-0.588	-1.5	2.56E-03	9.59E-03
238871_at	MLLT4	-0.588	-1.5	3.85E-06	5.32E-05
1569714_at	ZFYVE20	-0.588	-1.5	9.46E-06	1.09E-04
208249_s_at	TGDS	-0.589	-1.5	1.27E-08	5.43E-07
223005_s_at	TMEM245	-0.589	-1.5	6.19E-05	4.91E-04
232349_x_at	DCAF6	-0.589	-1.5	7.28E-05	5.60E-04
235281_x_at	AHNAK	-0.589	-1.5	1.03E-02	2.93E-02
217787_s_at	GALNT2	-0.589	-1.5	7.61E-07	1.44E-05
218755_at	KIF20A	-0.589	-1.5	2.65E-04	1.57E-03
222591_at	STYXL1	-0.58	-1.5	1.77E-05	1.81E-04

		9			
216600_x_at	ALDOB	-0.589	-1.5	1.67E-08	6.83E-07
226396_at	TEN1 /// TEN1-CDK3	-0.59	-1.5	1.55E-04	1.03E-03
212002_at	SZRD1	-0.59	-1.5	1.11E-03	4.90E-03
223145_s_at	AKIRIN2	-0.59	-1.51	3.81E-05	3.34E-04
237016_at	TMEM217	-0.59	-1.51	1.95E-03	7.70E-03
233660_at	EHD4	-0.59	-1.51	1.19E-04	8.37E-04
225697_at	CDK12	-0.59	-1.51	1.00E-03	4.53E-03
240078_at	SFSWAP	-0.591	-1.51	6.99E-05	5.42E-04
215533_s_at	UBE4B	-0.591	-1.51	8.28E-05	6.24E-04
205872_x_at	LOC101060353 /// PDE4DIP	-0.591	-1.51	9.53E-04	4.35E-03
210761_s_at	GRB7	-0.591	-1.51	1.56E-05	1.63E-04
229776_at	SLCO3A1	-0.591	-1.51	1.01E-06	1.80E-05
212442_s_at	CERS6	-0.591	-1.51	3.15E-06	4.53E-05
206582_s_at	GPR56	-0.592	-1.51	7.09E-07	1.36E-05
219112_at	FNIP1 /// RAPGEF6	-0.592	-1.51	1.79E-03	7.18E-03
212590_at	RRAS2	-0.592	-1.51	9.54E-07	1.72E-05
226352_at	JMY	-0.592	-1.51	9.26E-12	1.39E-09
204506_at	PPP3R1	-0.593	-1.51	2.63E-05	2.49E-04
215109_at	RC3H1	-0.593	-1.51	1.29E-03	5.53E-03
230630_at	AK4 /// LOC100507855	-0.593	-1.51	6.26E-03	1.97E-02
229252_at	ATG9B	-0.593	-1.51	2.89E-05	2.68E-04
205254_x_at	TCF7	-0.594	-1.51	1.05E-07	2.99E-06
214499_s_at	BCLAF1	-0.594	-1.51	2.84E-05	2.64E-04
33148_at	ZFR	-0.594	-1.51	2.95E-05	2.72E-04

227371_at	BAIAP2L1	-0.595	-1.51	1.50E-05	1.58E-04
1569064_at	C15orf62	-0.595	-1.51	3.83E-09	2.10E-07
212162_at	KIDINS220	-0.595	-1.51	1.94E-04	1.24E-03
203870_at	USP46	-0.595	-1.51	4.97E-07	1.02E-05
207120_at	ZNF667	-0.595	-1.51	8.75E-08	2.61E-06
232392_at	SRSF3	-0.595	-1.51	3.54E-09	1.97E-07
206652_at	ZMYM5	-0.595	-1.51	3.64E-03	1.27E-02
210711_at	LINC00260	-0.595	-1.51	2.49E-04	1.51E-03
212662_at	PVR	-0.595	-1.51	1.90E-04	1.21E-03
239148_at	MARVELD3	-0.596	-1.51	5.29E-06	6.87E-05
212720_at	PAPOLA	-0.596	-1.51	5.34E-04	2.74E-03
227405_s_at	FZD8	-0.596	-1.51	1.13E-04	8.05E-04
227241_at	MUC15	-0.597	-1.51	8.29E-05	6.24E-04
219001_s_at	DCAF10	-0.597	-1.51	6.38E-03	2.00E-02
222039_at	KIF18B	-0.597	-1.51	4.17E-06	5.67E-05
205152_at	SLC6A1	-0.597	-1.51	1.52E-04	1.02E-03
1552618_at	STX6	-0.598	-1.51	2.89E-05	2.68E-04
215754_at	SCARB2	-0.598	-1.51	2.03E-04	1.28E-03
1555972_s_at	FBXO28	-0.598	-1.51	9.76E-06	1.12E-04
243792_x_at	PTPN13	-0.598	-1.51	2.43E-03	9.17E-03
91684_g_at	EXOSC4	-0.598	-1.51	4.07E-09	2.21E-07
225211_at	PVRL1	-0.599	-1.51	9.20E-07	1.67E-05

229275_at	IGFN1	-0.599	-1.51	2.26E-05	2.21E-04
227998_at	S100A16	-0.599	-1.51	1.95E-12	3.72E-10
225387_at	TSPAN5	-0.599	-1.51	1.82E-08	7.32E-07
201364_s_at	OAZ2	-0.599	-1.52	3.89E-05	3.40E-04
40016_g_at	MAST4	-0.599	-1.52	4.28E-05	3.67E-04
204545_at	PEX6	-0.6	-1.52	2.29E-04	1.41E-03
206237_s_at	NRG1	-0.6	-1.52	1.31E-05	1.41E-04
223381_at	NUF2	-0.6	-1.52	4.73E-04	2.49E-03
221028_s_at	GFOD2	-0.6	-1.52	6.60E-08	2.10E-06
213796_at	SPRR1A	-0.6	-1.52	2.29E-06	3.50E-05
213931_at	ID2 /// ID2B	-0.601	-1.52	1.06E-04	7.61E-04
1554450_s_at	MIER3	-0.601	-1.52	1.49E-09	9.59E-08
218902_at	NOTCH1	-0.601	-1.52	1.60E-07	4.23E-06
206447_at	CELA2A /// CELA2B	-0.601	-1.52	3.76E-09	2.07E-07
203574_at	NFIL3	-0.602	-1.52	2.11E-06	3.28E-05
1566901_at	TGIF1	-0.602	-1.52	8.23E-05	6.21E-04
1553603_s_at	ATL2	-0.602	-1.52	2.11E-11	2.81E-09
208961_s_at	KLF6	-0.603	-1.52	6.49E-03	2.03E-02
219794_at	VPS53	-0.603	-1.52	3.03E-05	2.78E-04
224778_s_at	TAOK1	-0.603	-1.52	2.93E-04	1.71E-03
222654_at	IMPAD1	-0.603	-1.52	3.49E-08	1.26E-06
230468_s_at	C1orf56	-0.603	-1.52	1.41E-04	9.58E-04
223318_s_at	ALKBH7	-0.603	-1.52	1.86E-03	7.42E-03
225766_s_at	TNPO1	-0.603	-1.52	1.19E-08	5.19E-07

224674_at	TTYH3	-0.604	-1.52	6.34E-07	1.23E-05
214599_at	IVL	-0.604	-1.52	3.67E-05	3.24E-04
200733_s_at	PTP4A1	-0.604	-1.52	1.24E-11	1.79E-09
206011_at	CASP1	-0.604	-1.52	6.85E-09	3.35E-07
1554999_at	RASGEF1B	-0.604	-1.52	8.16E-05	6.16E-04
213924_at	GNAL	-0.604	-1.52	2.31E-08	9.04E-07
203313_s_at	TGIF1	-0.604	-1.52	4.52E-05	3.82E-04
1555151_s_at	TDH	-0.605	-1.52	9.00E-05	6.66E-04
203693_s_at	E2F3	-0.605	-1.52	7.56E-10	5.51E-08
202094_at	BIRC5	-0.605	-1.52	2.76E-03	1.02E-02
224631_at	ZFP91	-0.605	-1.52	1.69E-04	1.11E-03
207286_at	CEP135	-0.605	-1.52	2.57E-03	9.63E-03
222395_s_at	UBE2Z	-0.605	-1.52	3.44E-04	1.93E-03
1569385_s_at	TET2	-0.605	-1.52	1.85E-04	1.19E-03
227911_at	ARHGAP28	-0.605	-1.52	2.55E-04	1.53E-03
241946_at	ZDHHC21	-0.605	-1.52	6.63E-06	8.15E-05
235793_at	TPGS2	-0.606	-1.52	1.01E-05	1.15E-04
242722_at	LMO7	-0.606	-1.52	1.05E-02	2.97E-02
212872_s_at	MED20	-0.606	-1.52	1.64E-07	4.31E-06
201684_s_at	TOX4	-0.606	-1.52	3.69E-08	1.32E-06
202954_at	UBE2C	-0.606	-1.52	2.24E-06	3.44E-05
210305_at	PDE4DIP	-0.606	-1.52	5.62E-04	2.86E-03

221648_s_at	AGMAT	-0.606	-1.52	1.96E-03	7.74E-03
202897_at	SIRPA	-0.607	-1.52	6.65E-09	3.27E-07
225685_at	CDC42EP3	-0.607	-1.52	2.72E-05	2.56E-04
230441_at	PLEKHG4B	-0.607	-1.52	1.06E-05	1.20E-04
1556567_at	NAP1L4	-0.607	-1.52	3.10E-07	7.10E-06
229213_at	DIRC2	-0.607	-1.52	3.02E-04	1.75E-03
229596_at	AMDHD1	-0.607	-1.52	8.44E-03	2.51E-02
207724_s_at	SPAST	-0.608	-1.52	1.43E-04	9.71E-04
217080_s_at	HOMER2	-0.608	-1.52	1.35E-07	3.71E-06
212686_at	PPM1H	-0.608	-1.52	3.79E-05	3.32E-04
232232_s_at	SLC22A16	-0.608	-1.52	1.20E-03	5.23E-03
213172_at	TTC9	-0.608	-1.52	7.08E-04	3.42E-03
209647_s_at	SOCS5	-0.608	-1.52	6.39E-08	2.06E-06
239355_at	GMCL1	-0.609	-1.52	6.00E-09	3.01E-07
213713_s_at	GLB1L2	-0.609	-1.53	1.28E-05	1.38E-04
225792_at	HOOK1	-0.609	-1.53	2.16E-06	3.34E-05
229682_at	MAPRE3	-0.61	-1.53	1.25E-07	3.49E-06
1559975_at	BTG1	-0.61	-1.53	1.55E-04	1.03E-03
1558050_at	EIF2B5	-0.61	-1.53	4.36E-07	9.15E-06
208054_at	HERC4	-0.61	-1.53	1.52E-03	6.31E-03
202500_at	DNAJB2	-0.611	-1.53	3.12E-07	7.13E-06
235463_s_at	CERS6	-0.611	-1.53	8.75E-08	2.61E-06
226778_at	TDRP	-0.611	-1.53	4.01E-07	8.60E-06
210148_at	HIPK3	-0.61	-1.53	1.85E-06	2.95E-05

		2			
1554795_a_at	FBLIM1	-0.612	-1.53	2.04E-05	2.03E-04
1569065_s_at	C15orf62	-0.612	-1.53	2.56E-07	6.13E-06
201668_x_at	MARCKS	-0.612	-1.53	5.56E-05	4.51E-04
236953_s_at	NHLRC3	-0.613	-1.53	1.24E-04	8.68E-04
237346_at	TGDS	-0.613	-1.53	6.17E-05	4.89E-04
239576_at	MTUS1	-0.613	-1.53	8.19E-05	6.18E-04
226568_at	FAM102B	-0.613	-1.53	3.52E-06	4.95E-05
220528_at	VNN3	-0.613	-1.53	1.56E-02	4.11E-02
232856_at	LRRC55	-0.614	-1.53	1.42E-04	9.63E-04
203018_s_at	SSX2IP	-0.614	-1.53	1.63E-04	1.07E-03
234335_s_at	FAM84A	-0.614	-1.53	1.28E-07	3.56E-06
232371_at	7-Mar	-0.614	-1.53	5.65E-07	1.12E-05
210954_s_at	TSC22D2	-0.614	-1.53	9.96E-06	1.14E-04
229398_at	RAB18	-0.615	-1.53	1.69E-04	1.11E-03
214374_s_at	PPFIBP1	-0.615	-1.53	3.83E-03	1.33E-02
1554769_at	ZNF785	-0.615	-1.53	5.22E-05	4.30E-04
201647_s_at	SCARB2	-0.615	-1.53	3.19E-07	7.25E-06
235294_at	SIKE1	-0.616	-1.53	7.81E-04	3.70E-03
236313_at	CDKN2B	-0.616	-1.53	1.31E-12	2.63E-10
233029_at	OBSCN	-0.616	-1.53	2.16E-08	8.54E-07
214423_x_at	ALDOB	-0.616	-1.53	1.96E-08	7.83E-07
1554770_x_at	ZNF785	-0.61	-1.53	1.41E-05	1.50E-04

		6			
1559870_at	LOC100129129	-0.616	-1.53	1.73E-07	4.48E-06
209496_at	RARRES2	-0.616	-1.53	1.04E-03	4.68E-03
232322_x_at	STARD10	-0.616	-1.53	4.74E-05	3.98E-04
227283_at	EFR3B	-0.617	-1.53	2.12E-04	1.32E-03
225937_at	CUX1	-0.617	-1.53	6.34E-05	5.01E-04
239650_at	NCKAP5	-0.617	-1.53	4.35E-06	5.88E-05
205864_at	SLC7A4	-0.617	-1.53	9.88E-06	1.13E-04
220193_at	SH3D21	-0.617	-1.53	6.57E-04	3.23E-03
217494_s_at	PTENP1	-0.618	-1.53	8.65E-05	6.46E-04
203355_s_at	PSD3	-0.618	-1.53	1.37E-07	3.74E-06
230964_at	FREM2	-0.618	-1.53	1.45E-02	3.87E-02
217202_s_at	GLUL	-0.618	-1.53	1.11E-04	7.92E-04
200924_s_at	SLC3A2	-0.618	-1.53	6.53E-08	2.09E-06
1569149_at	PDLIM7	-0.618	-1.54	5.60E-09	2.87E-07
204029_at	CELSR2	-0.619	-1.54	3.98E-14	1.34E-11
203129_s_at	KIF5C	-0.619	-1.54	4.79E-10	3.81E-08
236013_at	CACNA1E	-0.619	-1.54	3.18E-04	1.82E-03
228587_at	FAM83G	-0.62	-1.54	1.14E-07	3.23E-06
1558540_s_at	SLC2A11	-0.62	-1.54	3.03E-06	4.39E-05
212532_s_at	LSM12	-0.62	-1.54	1.04E-06	1.85E-05
215318_at	MINOS1P1	-0.62	-1.54	3.57E-03	1.25E-02
36499_at	CELSR2	-0.62	-1.54	4.43E-13	1.06E-10
226003_at	KIF21A	-0.62	-1.54	9.92E-11	1.02E-08
202131_s_at	RIOK3	-0.62	-1.54	7.04E-05	5.45E-04

1552482_at	RAPH1	-0.62	-1.54	1.84E-03	7.34E-03
230332_at	ZCCHC7	-0.621	-1.54	1.00E-02	2.88E-02
202899_s_at	SRSF3	-0.621	-1.54	4.14E-07	8.83E-06
238831_at	TMEM33	-0.621	-1.54	1.19E-02	3.29E-02
203362_s_at	MAD2L1	-0.621	-1.54	4.26E-06	5.78E-05
244427_at	KIF23	-0.621	-1.54	5.58E-05	4.52E-04
228128_x_at	PAPPA	-0.621	-1.54	1.05E-05	1.19E-04
201466_s_at	JUN	-0.621	-1.54	2.20E-05	2.16E-04
241733_at	C18orf54	-0.622	-1.54	8.79E-07	1.61E-05
209803_s_at	PHLDA2	-0.622	-1.54	3.30E-04	1.88E-03
212193_s_at	LARP1	-0.622	-1.54	9.94E-04	4.50E-03
235727_at	KLHL28	-0.622	-1.54	1.04E-02	2.96E-02
202005_at	ST14	-0.622	-1.54	1.65E-04	1.09E-03
236001_at	LINC00675	-0.622	-1.54	6.39E-05	5.03E-04
205034_at	CCNE2	-0.623	-1.54	1.60E-05	1.67E-04
203803_at	PCYOX1	-0.623	-1.54	4.01E-03	1.38E-02
200695_at	PPP2R1A	-0.623	-1.54	3.30E-04	1.88E-03
201233_at	PSMD13	-0.624	-1.54	2.56E-04	1.53E-03
211352_s_at	NCOA3	-0.624	-1.54	1.44E-03	6.03E-03
202092_s_at	ARL2BP	-0.624	-1.54	6.95E-05	5.39E-04
222875_at	DHX33	-0.624	-1.54	1.67E-05	1.72E-04
226980_at	DEPDC1B	-0.625	-1.54	1.39E-04	9.47E-04
208141_s_at	DOHH	-0.62	-1.54	6.67E-05	5.21E-04

		5			
211366_x_at	CASP1	-0.625	-1.54	1.39E-10	1.33E-08
233520_s_at	CMYA5	-0.625	-1.54	5.04E-04	2.61E-03
225097_at	HIPK2	-0.626	-1.54	3.56E-04	1.99E-03
207213_s_at	USP2	-0.626	-1.54	3.71E-04	2.06E-03
208625_s_at	EIF4G1	-0.626	-1.54	8.58E-06	1.01E-04
200838_at	CTSB	-0.626	-1.54	2.76E-09	1.60E-07
1556842_at	LOC286087	-0.626	-1.54	4.58E-04	2.43E-03
1558002_at	STRAP	-0.626	-1.54	2.68E-04	1.59E-03
1556551_s_at	SLC39A6	-0.626	-1.54	2.54E-04	1.53E-03
222458_s_at	AKIRIN1	-0.627	-1.54	3.35E-04	1.90E-03
225246_at	STIM2	-0.627	-1.54	7.20E-07	1.38E-05
215084_s_at	LRRC42	-0.627	-1.54	1.14E-06	1.99E-05
234351_x_at	TRPS1	-0.627	-1.54	2.11E-04	1.32E-03
229442_at	C18orf54	-0.627	-1.54	1.71E-04	1.12E-03
205464_at	SCNN1B	-0.627	-1.54	1.97E-05	1.98E-04
213446_s_at	IQGAP1	-0.627	-1.54	9.45E-03	2.74E-02
229280_s_at	LINC00340	-0.628	-1.55	1.00E-03	4.52E-03
220625_s_at	ELF5	-0.628	-1.55	3.98E-04	2.17E-03
217921_at	MAN1A2	-0.628	-1.55	4.03E-06	5.52E-05
1560854_s_at	ZNF107	-0.628	-1.55	9.38E-04	4.29E-03
202575_at	CRABP2	-0.628	-1.55	7.75E-05	5.90E-04
237030_at	ACPP	-0.62	-1.55	2.38E-05	2.30E-04

		8			
225234_at	CBL	-0.628	-1.55	1.38E-04	9.42E-04
1559715_at	LOC100507391	-0.629	-1.55	5.75E-09	2.93E-07
203707_at	LINC00921 /// ZNF263	-0.629	-1.55	2.32E-07	5.67E-06
229290_at	DAPL1	-0.629	-1.55	3.64E-08	1.30E-06
209376_x_at	SCAF11	-0.629	-1.55	9.02E-05	6.67E-04
202969_at	DYRK2	-0.63	-1.55	8.48E-09	3.96E-07
202730_s_at	MIR4680 /// PDCD4	-0.63	-1.55	1.89E-09	1.18E-07
239654_at	CHD9	-0.63	-1.55	9.37E-04	4.29E-03
202613_at	CTPS1	-0.63	-1.55	1.99E-09	1.23E-07
1569150_x_at	PDLIM7	-0.63	-1.55	1.82E-07	4.67E-06
1553722_s_at	RNF152	-0.63	-1.55	1.22E-05	1.34E-04
1552903_at	B4GALNT2	-0.63	-1.55	4.82E-08	1.63E-06
204427_s_at	TMED2	-0.63	-1.55	1.68E-03	6.81E-03
226824_at	CPXM2	-0.631	-1.55	2.30E-06	3.52E-05
207791_s_at	RAB1A	-0.631	-1.55	1.53E-03	6.32E-03
223243_s_at	EDEM3	-0.631	-1.55	4.73E-03	1.58E-02
1555996_s_at	EIF4A2 /// MIR1248 /// SNORA4 /// SNORA63 /// SNORA81 /// SNORD2	-0.631	-1.55	6.51E-06	8.04E-05
203391_at	FKBP2	-0.631	-1.55	3.54E-05	3.14E-04
209792_s_at	KLK10	-0.631	-1.55	5.33E-05	4.37E-04
209626_s_at	OSBPL3	-0.632	-1.55	5.86E-09	2.96E-07
231881_at	CALD1	-0.632	-1.55	3.69E-05	3.25E-04
200596_s_at	EIF3A	-0.632	-1.55	1.71E-04	1.12E-03
202124_s_at	TRAK2	-0.632	-1.55	8.92E-06	1.04E-04
201732_s_at	CLCN3	-0.632	-1.55	7.63E-04	3.63E-03
227032_at	PLXNA2	-	-	6.09E-06	7.65E-05

		0.633	1.55		
225219_at	SMAD5	-0.633	-1.55	4.50E-06	6.03E-05
209925_at	OCLN	-0.633	-1.55	8.62E-06	1.01E-04
235136_at	ORMDL3	-0.633	-1.55	5.39E-06	6.96E-05
208077_at	C9orf38	-0.633	-1.55	1.06E-02	3.01E-02
1552390_a_at	C8orf47	-0.633	-1.55	6.20E-08	2.00E-06
228404_at	IRX2	-0.633	-1.55	2.65E-08	1.01E-06
244801_at	PSMB7	-0.633	-1.55	1.56E-04	1.04E-03
203397_s_at	GALNT3	-0.634	-1.55	5.37E-11	5.96E-09
242186_x_at	LPHN3	-0.634	-1.55	5.92E-04	2.97E-03
233641_s_at	FAM167A	-0.634	-1.55	2.11E-05	2.08E-04
1562321_at	PDK4	-0.634	-1.55	3.32E-03	1.19E-02
212983_at	HRAS	-0.634	-1.55	1.11E-05	1.24E-04
206196_s_at	RUNDC3A	-0.634	-1.55	2.36E-05	2.29E-04
223599_at	TRIM6	-0.635	-1.55	3.20E-03	1.15E-02
235089_at	FBXL20	-0.635	-1.55	3.58E-06	5.02E-05
222570_at	NCS1	-0.636	-1.55	5.96E-04	2.99E-03
1555372_at	BCL2L11	-0.636	-1.55	2.03E-04	1.28E-03
205375_at	MDFI	-0.636	-1.55	5.93E-09	2.98E-07
202516_s_at	DLG1	-0.636	-1.55	1.63E-05	1.69E-04
208456_s_at	RRAS2	-0.636	-1.55	1.05E-04	7.55E-04
228824_s_at	PTGR1	-0.637	-1.55	1.13E-03	4.98E-03
213899_at	METAP2	-0.63	-1.55	4.24E-07	8.96E-06

		7			
232983_s_at	SERGEF	-0.637	-1.56	3.42E-05	3.05E-04
207467_x_at	CAST	-0.637	-1.56	2.49E-11	3.20E-09
224563_at	WASF2	-0.637	-1.56	7.81E-03	2.36E-02
228080_at	LAYN	-0.638	-1.56	3.59E-04	2.00E-03
206543_at	SMARCA2	-0.638	-1.56	3.72E-05	3.27E-04
222537_s_at	CDC42SE1	-0.638	-1.56	8.53E-08	2.57E-06
227299_at	CCNI	-0.638	-1.56	7.31E-03	2.24E-02
205138_s_at	UST	-0.638	-1.56	3.75E-08	1.33E-06
238231_at	NFYC	-0.638	-1.56	5.41E-03	1.76E-02
220382_s_at	ARHGAP28	-0.638	-1.56	5.76E-07	1.14E-05
215203_at	GOLGA4	-0.639	-1.56	4.55E-03	1.53E-02
236006_s_at	AKAP10	-0.639	-1.56	2.52E-04	1.52E-03
206382_s_at	BDNF	-0.639	-1.56	1.74E-06	2.80E-05
212783_at	RBBP6	-0.639	-1.56	3.34E-08	1.21E-06
1555814_a_at	RHOA	-0.639	-1.56	2.62E-03	9.79E-03
203017_s_at	SSX2IP	-0.639	-1.56	1.02E-08	4.60E-07
241705_at	ABCA5	-0.64	-1.56	2.07E-03	8.07E-03
230195_at	LOC100131138	-0.64	-1.56	2.94E-07	6.84E-06
1569815_x_at	STRN	-0.64	-1.56	8.10E-05	6.12E-04
201830_s_at	NET1	-0.64	-1.56	8.51E-09	3.97E-07
229082_at	CCDC125	-0.64	-1.56	9.29E-04	4.26E-03
201435_s_at	EIF4E	-0.64	-1.56	3.85E-08	1.35E-06
204903_x_at	ATG4B	-0.64	-1.56	6.70E-07	1.29E-05
232303_at	ZNF608	-0.64	-1.56	3.65E-06	5.10E-05
218045_x_at	PTMS	-0.64	-	2.08E-05	2.07E-04

			1.56		
206877_at	MXD1	-0.641	-1.56	3.78E-08	1.33E-06
218136_s_at	SLC25A37	-0.641	-1.56	4.58E-07	9.54E-06
201526_at	ARF5	-0.641	-1.56	3.18E-04	1.83E-03
229474_at	MICAL3	-0.642	-1.56	1.05E-07	3.00E-06
222422_s_at	NDFIP1	-0.642	-1.56	8.30E-08	2.51E-06
202744_at	SLC20A2	-0.642	-1.56	9.67E-09	4.40E-07
209920_at	BMPR2	-0.642	-1.56	4.28E-05	3.67E-04
1569387_at	CSGALNACT1	-0.642	-1.56	1.72E-08	6.98E-07
1560553_at	TIAF1	-0.642	-1.56	2.62E-06	3.92E-05
216733_s_at	GATM	-0.642	-1.56	1.77E-03	7.12E-03
214121_x_at	PDLIM7	-0.642	-1.56	3.91E-07	8.45E-06
213341_at	FEM1C	-0.642	-1.56	1.02E-07	2.95E-06
209773_s_at	RRM2	-0.643	-1.56	2.19E-03	8.45E-03
1568717_a_at	FKBP15	-0.643	-1.56	4.88E-03	1.62E-02
214693_x_at	LOC101059961 /// LOC101060202 /// LOC101060362 /// LOC101060684 /// NBPF10 /// NBPF12 /// NBPF9	-0.644	-1.56	8.66E-05	6.46E-04
214357_at	C1orf105	-0.644	-1.56	4.50E-05	3.82E-04
201208_s_at	TNFAIP1	-0.645	-1.56	1.83E-09	1.14E-07
221815_at	ABHD2	-0.645	-1.56	4.31E-03	1.46E-02
201320_at	SMARCC2	-0.645	-1.56	5.71E-07	1.13E-05
224901_at	SCD5	-0.645	-1.56	4.20E-05	3.61E-04
1570238_at	ZNF527	-0.645	-1.56	2.05E-04	1.29E-03
1553589_a_at	PDZK1IP1	-0.64	-1.56	8.98E-03	2.63E-02

		5			
1555749_at	SF1	-0.645	-1.56	1.98E-04	1.25E-03
223906_s_at	TEX101	-0.646	-1.56	4.79E-03	1.59E-02
235088_at	C4orf46	-0.646	-1.56	6.06E-07	1.19E-05
228432_at	RAB3IP	-0.646	-1.57	5.94E-06	7.51E-05
213637_at	DDX52	-0.646	-1.57	2.39E-04	1.46E-03
238909_at	S100A10	-0.647	-1.57	3.09E-05	2.82E-04
218489_s_at	ALAD	-0.647	-1.57	1.41E-09	9.18E-08
244771_at	KBTBD12	-0.647	-1.57	4.27E-03	1.45E-02
243899_at	ARL17A /// ARL17B	-0.647	-1.57	3.14E-03	1.13E-02
214004_s_at	VGLL4	-0.647	-1.57	1.65E-07	4.33E-06
217100_s_at	UBXN7	-0.647	-1.57	4.13E-04	2.24E-03
214168_s_at	TJP1	-0.648	-1.57	6.56E-06	8.08E-05
235114_x_at	HOOK3	-0.648	-1.57	2.68E-04	1.59E-03
241620_at	SMCHD1	-0.648	-1.57	4.20E-05	3.62E-04
210417_s_at	PI4KB	-0.648	-1.57	2.88E-04	1.69E-03
229399_at	C10orf118	-0.648	-1.57	5.66E-05	4.57E-04
1555971_s_at	FBXO28	-0.648	-1.57	8.27E-08	2.51E-06
235857_at	KCTD11	-0.649	-1.57	1.38E-05	1.47E-04
211132_at	INTS3	-0.649	-1.57	9.93E-05	7.19E-04
212335_at	GNS	-0.649	-1.57	4.28E-08	1.47E-06
206235_at	LIG4	-0.649	-1.57	2.67E-09	1.56E-07
219271_at	GALNT14	-0.65	-1.57	2.38E-06	3.63E-05

214877_at	CDKAL1	-0.65	-1.57	1.71E-04	1.12E-03
225262_at	FOSL2	-0.65	-1.57	2.85E-06	4.19E-05
206367_at	REN	-0.65	-1.57	3.80E-04	2.10E-03
210009_s_at	GOSR2	-0.65	-1.57	1.74E-04	1.13E-03
1553425_at	WDR65	-0.65	-1.57	4.88E-08	1.64E-06
209970_x_at	CASP1	-0.651	-1.57	5.74E-11	6.30E-09
203842_s_at	MAPRE3	-0.651	-1.57	5.31E-11	5.91E-09
206540_at	GLB1L	-0.652	-1.57	2.99E-06	4.36E-05
228033_at	E2F7	-0.652	-1.57	6.77E-05	5.27E-04
211537_x_at	MAP3K7	-0.652	-1.57	1.40E-05	1.49E-04
242592_at	GPR137C	-0.652	-1.57	2.52E-05	2.41E-04
208705_s_at	EIF5	-0.653	-1.57	2.11E-07	5.29E-06
218960_at	TMPRSS4	-0.653	-1.57	2.84E-03	1.05E-02
206109_at	FUT1	-0.653	-1.57	1.33E-08	5.62E-07
1556990_at	PERP	-0.653	-1.57	1.07E-03	4.77E-03
214875_x_at	APLP2	-0.653	-1.57	2.47E-06	3.74E-05
37028_at	PPP1R15A	-0.654	-1.57	8.53E-06	1.00E-04
215611_at	TCF12	-0.654	-1.57	1.54E-04	1.03E-03
225612_s_at	B3GNT5	-0.654	-1.57	2.97E-11	3.70E-09
1555827_at	CCNL1	-0.655	-1.57	2.65E-04	1.57E-03
213897_s_at	MRPL23	-0.655	-1.57	5.57E-04	2.84E-03
202437_s_at	CYP1B1	-0.655	-1.57	1.06E-03	4.73E-03
231193_s_at	TAOK1	-0.655	-1.57	7.02E-04	3.40E-03
208707_at	EIF5	-0.655	-1.57	3.30E-07	7.42E-06

210535_at	B9D1	-0.655	-1.57	2.75E-08	1.04E-06
206164_at	CLCA2	-0.656	-1.58	1.59E-07	4.21E-06
201683_x_at	TOX4	-0.656	-1.58	1.10E-04	7.85E-04
238756_at	GAS2L3	-0.656	-1.58	5.37E-08	1.78E-06
226077_at	RNF145	-0.656	-1.58	7.97E-08	2.44E-06
232217_at	FAM26E	-0.657	-1.58	1.38E-02	3.72E-02
227748_at	RBMXL1	-0.657	-1.58	8.26E-04	3.87E-03
37577_at	ARHGAP19	-0.657	-1.58	7.81E-08	2.40E-06
224568_x_at	MALAT1	-0.657	-1.58	1.57E-07	4.18E-06
219980_at	C4orf29	-0.657	-1.58	1.09E-03	4.84E-03
223349_s_at	BOK	-0.658	-1.58	3.67E-06	5.12E-05
201848_s_at	BNIP3	-0.658	-1.58	1.12E-08	4.95E-07
235225_at	SCN2B	-0.658	-1.58	1.68E-07	4.39E-06
227481_at	CNKSR3	-0.658	-1.58	4.89E-07	1.00E-05
215074_at	MYO1B	-0.658	-1.58	6.42E-06	7.95E-05
229427_at	SEMA5A	-0.658	-1.58	1.30E-05	1.41E-04
202669_s_at	EFNB2	-0.658	-1.58	2.68E-03	9.97E-03
202971_s_at	DYRK2	-0.659	-1.58	1.73E-05	1.78E-04
1554572_a_at	SUV39H2	-0.659	-1.58	1.32E-07	3.62E-06
1554021_a_at	ZNF12	-0.659	-1.58	1.78E-03	7.14E-03
213913_s_at	TBC1D30	-0.659	-1.58	1.47E-02	3.92E-02
202731_at	MIR4680 /// PDCD4	-0.66	-1.58	4.25E-07	9.00E-06
217272_s_at	SERPINB13	-0.66	-1.58	4.49E-11	5.20E-09

230492_s_at	GPCPD1	-0.66	-1.58	3.57E-07	7.89E-06
236094_at	TCF7L2	-0.66	-1.58	1.98E-06	3.12E-05
39249_at	AQP3	-0.66	-1.58	1.85E-02	4.69E-02
216235_s_at	EDNRA	-0.66	-1.58	2.42E-07	5.87E-06
223281_s_at	COX15	-0.661	-1.58	4.58E-06	6.12E-05
228360_at	LYPD6B	-0.661	-1.58	2.67E-09	1.56E-07
1554411_at	CTNNB1	-0.661	-1.58	4.54E-06	6.08E-05
206400_at	LGALS7 /// LGALS7B	-0.661	-1.58	1.89E-03	7.52E-03
1567458_s_at	RAC1	-0.662	-1.58	1.41E-05	1.50E-04
205436_s_at	H2AFX	-0.662	-1.58	4.13E-06	5.63E-05
242079_at	RGS12	-0.662	-1.58	2.80E-05	2.61E-04
201123_s_at	EIF5A	-0.662	-1.58	1.05E-03	4.69E-03
220219_s_at	LRRC37A /// LRRC37A2 /// LRRC37A3 /// LRRC37A4P	-0.662	-1.58	2.28E-03	8.74E-03
235068_at	ZDHHC21	-0.662	-1.58	1.87E-10	1.73E-08
222439_s_at	THRAP3	-0.663	-1.58	5.46E-04	2.79E-03
212831_at	MEGF9	-0.663	-1.58	1.05E-03	4.71E-03
241383_at	ZNF385C	-0.664	-1.58	1.12E-04	7.98E-04
214918_at	HNRNPM	-0.664	-1.58	8.65E-03	2.55E-02
219775_s_at	CPLX3	-0.664	-1.58	6.59E-07	1.28E-05
200997_at	RBM14-RBM4 /// RBM4	-0.664	-1.58	1.82E-09	1.14E-07
223661_at	NUCKS1	-0.664	-1.58	2.93E-04	1.71E-03
1564525_at	GSN	-0.664	-1.58	1.59E-06	2.61E-05
239511_s_at	SRSF4	-0.664	-1.58	3.77E-04	2.08E-03
214580_x_at	KRT6A /// KRT6B /// KRT6C	-0.66	-1.59	2.91E-08	1.10E-06

		5			
243649_at	FBXO7	-0.665	-1.59	2.75E-06	4.08E-05
213938_at	ERC2	-0.665	-1.59	4.87E-04	2.55E-03
214730_s_at	GLG1	-0.665	-1.59	8.26E-05	6.23E-04
210125_s_at	BANF1	-0.666	-1.59	1.81E-05	1.84E-04
200638_s_at	YWHAZ	-0.666	-1.59	6.46E-08	2.07E-06
218861_at	RNF25	-0.666	-1.59	9.00E-06	1.05E-04
219671_at	HPCAL4	-0.667	-1.59	3.14E-07	7.17E-06
225240_s_at	MSI2	-0.667	-1.59	1.07E-04	7.65E-04
218929_at	CDKN2AIP	-0.667	-1.59	1.02E-10	1.05E-08
227801_at	TRIM59	-0.667	-1.59	3.70E-06	5.15E-05
206972_s_at	GPR161	-0.667	-1.59	3.87E-07	8.38E-06
222244_s_at	TUG1	-0.667	-1.59	1.24E-04	8.68E-04
203056_s_at	PRDM2	-0.668	-1.59	7.95E-04	3.75E-03
200641_s_at	YWHAZ	-0.668	-1.59	2.01E-05	2.01E-04
206581_at	BNC1	-0.668	-1.59	6.24E-10	4.77E-08
228851_s_at	ENSA	-0.668	-1.59	3.73E-04	2.07E-03
232436_at	ZNF274	-0.668	-1.59	1.24E-05	1.36E-04
215201_at	REPS1	-0.669	-1.59	8.36E-05	6.29E-04
241621_at	SMCHD1	-0.669	-1.59	2.50E-04	1.51E-03
206007_at	PRG4	-0.669	-1.59	7.70E-04	3.66E-03
1558233_s_at	ATF1	-0.669	-1.59	2.07E-07	5.20E-06
242922_at	NOMO3	-0.66	-1.59	2.61E-05	2.48E-04

		9			
210372_s_at	TPD52L1	-0.669	-1.59	1.16E-04	8.20E-04
232914_s_at	SYTL2	-0.669	-1.59	1.32E-04	9.08E-04
227828_s_at	EVA1A	-0.67	-1.59	8.03E-05	6.08E-04
219571_s_at	ZNF12	-0.67	-1.59	5.57E-04	2.84E-03
235547_at	N4BP2L2	-0.67	-1.59	3.73E-03	1.30E-02
228531_at	SAMD9	-0.67	-1.59	1.20E-08	5.23E-07
226933_s_at	ID4	-0.67	-1.59	4.46E-09	2.36E-07
219648_at	MREG	-0.67	-1.59	2.26E-15	1.35E-12
200664_s_at	DNAJB1	-0.671	-1.59	5.14E-09	2.67E-07
238168_at	TM4SF1	-0.671	-1.59	1.18E-10	1.17E-08
224889_at	FOXO3	-0.671	-1.59	2.19E-04	1.36E-03
1558249_s_at	STX16	-0.671	-1.59	1.91E-07	4.86E-06
224762_at	SERINC2	-0.672	-1.59	4.08E-08	1.41E-06
210061_at	ZNF589	-0.672	-1.59	2.03E-03	7.97E-03
231247_s_at	LOC727820	-0.672	-1.59	4.41E-03	1.49E-02
243435_at	KCNQ1OT1	-0.673	-1.59	1.22E-03	5.30E-03
212897_at	CDK19	-0.673	-1.59	2.06E-05	2.05E-04
222413_s_at	KMT2C	-0.673	-1.59	3.17E-04	1.82E-03
222407_s_at	ZNF106	-0.674	-1.6	7.78E-04	3.69E-03
213330_s_at	STIP1	-0.674	-1.6	1.18E-03	5.17E-03
215817_at	SERPINB13	-0.674	-1.6	3.09E-07	7.09E-06
1564063_a_at	ATP11B	-0.674	-1.6	1.42E-05	1.50E-04
222787_s_at	TMEM106B	-0.674	-1.6	1.93E-03	7.63E-03
215123_at	LOC613037 /// NPIPB11 /// NPIPB3 /// NPIPB4 /// NPIPB5	-0.67	-1.6	4.06E-03	1.39E-02

		4			
216086_at	SV2C	-0.674	-1.6	1.63E-05	1.69E-04
223103_at	STARD10	-0.675	-1.6	4.67E-06	6.22E-05
229240_at	ZDHHC21	-0.675	-1.6	4.95E-07	1.01E-05
202431_s_at	MYC	-0.675	-1.6	3.95E-04	2.16E-03
223682_s_at	EIF1AD	-0.675	-1.6	1.50E-07	4.03E-06
208730_x_at	RAB2A	-0.676	-1.6	1.14E-11	1.65E-09
206746_at	BFSP1	-0.676	-1.6	1.50E-09	9.61E-08
213692_s_at	VDR	-0.677	-1.6	5.15E-06	6.71E-05
236492_at	PPP2R2A	-0.677	-1.6	1.75E-04	1.14E-03
222581_at	XPR1	-0.677	-1.6	6.03E-09	3.03E-07
241455_at	C6orf132	-0.677	-1.6	4.39E-06	5.91E-05
223786_at	CHST6	-0.677	-1.6	5.12E-04	2.65E-03
205422_s_at	ITGBL1	-0.677	-1.6	6.42E-04	3.18E-03
204235_s_at	GULP1	-0.677	-1.6	1.39E-03	5.90E-03
1553757_at	IQCG	-0.678	-1.6	5.00E-03	1.65E-02
229966_at	EWSR1	-0.678	-1.6	3.00E-03	1.09E-02
222082_at	ZBTB7A	-0.678	-1.6	1.04E-06	1.84E-05
243852_at	LUC7L2	-0.679	-1.6	9.27E-06	1.07E-04
221455_s_at	WNT3	-0.679	-1.6	4.59E-13	1.08E-10
222310_at	SCAF4	-0.679	-1.6	5.64E-05	4.56E-04
222878_s_at	OTUB2	-0.679	-1.6	1.81E-04	1.17E-03
225388_at	TSPAN5	-0.67	-1.6	1.45E-06	2.43E-05

		9			
228846_at	MXD1	-0.679	-1.6	6.58E-09	3.24E-07
1562270_at	ARHGEF7	-0.679	-1.6	4.60E-03	1.54E-02
202363_at	SPOCK1	-0.68	-1.6	9.53E-06	1.10E-04
230742_at	RBM5	-0.68	-1.6	5.88E-04	2.95E-03
231366_at	FDPSP2	-0.68	-1.6	5.27E-03	1.72E-02
1554556_a_at	ATP11B	-0.68	-1.6	4.66E-06	6.20E-05
202461_at	EIF2B2	-0.681	-1.6	4.87E-08	1.64E-06
1558356_at	UACA	-0.681	-1.6	1.12E-06	1.96E-05
226027_at	SWI5	-0.681	-1.6	4.31E-07	9.09E-06
202549_at	VAPB	-0.681	-1.6	4.12E-05	3.56E-04
231716_at	RC3H2	-0.681	-1.6	2.10E-03	8.17E-03
221744_at	DCAF7	-0.682	-1.6	5.52E-05	4.48E-04
206668_s_at	SCAMP1	-0.683	-1.61	2.93E-04	1.71E-03
203706_s_at	FZD7	-0.683	-1.61	6.02E-08	1.96E-06
222636_at	MED28	-0.684	-1.61	3.67E-06	5.12E-05
229514_at	GPATCH2L	-0.684	-1.61	3.40E-05	3.04E-04
1569353_at	CCP110	-0.685	-1.61	2.11E-04	1.32E-03
202889_x_at	MAP7	-0.685	-1.61	3.93E-07	8.48E-06
225142_at	JHDM1D	-0.685	-1.61	4.01E-06	5.51E-05
1552365_at	SCIN	-0.685	-1.61	4.65E-03	1.56E-02
226313_at	C10orf35	-0.685	-1.61	1.67E-08	6.83E-07
227384_s_at	LINC00623 /// LOC727820 /// LOC728875	-0.685	-1.61	1.73E-03	7.00E-03
1553672_at	ENAH	-0.686	-1.61	1.21E-08	5.23E-07
233226_at	PTPN9	-0.68	-1.61	3.36E-05	3.01E-04

		6			
232613_at	PBRM1	-0.687	-1.61	1.48E-04	9.98E-04
239843_at	RIT1	-0.687	-1.61	1.52E-07	4.08E-06
219181_at	LIPG	-0.687	-1.61	3.18E-03	1.14E-02
202723_s_at	FOXO1	-0.688	-1.61	3.30E-04	1.88E-03
202290_at	PDAP1	-0.688	-1.61	2.30E-06	3.52E-05
205543_at	HSPA4L	-0.688	-1.61	1.06E-07	3.01E-06
209307_at	SWAP70	-0.689	-1.61	5.83E-06	7.40E-05
232233_at	SLC22A16	-0.689	-1.61	4.06E-08	1.41E-06
238807_at	ANKRD46 /// GAPDHP62	-0.689	-1.61	1.75E-04	1.14E-03
221693_s_at	MRPS18A	-0.689	-1.61	8.88E-03	2.61E-02
209257_s_at	SMC3	-0.69	-1.61	7.19E-04	3.46E-03
1553785_at	RASGEF1B	-0.69	-1.61	4.41E-04	2.36E-03
1558719_s_at	RPAIN	-0.69	-1.61	2.42E-03	9.16E-03
201487_at	CTSC	-0.69	-1.61	1.24E-05	1.36E-04
215471_s_at	MAP7	-0.691	-1.61	4.68E-06	6.22E-05
212629_s_at	PKN2	-0.691	-1.61	6.60E-04	3.24E-03
200751_s_at	HNRNPC	-0.691	-1.61	4.93E-05	4.10E-04
219330_at	VANGL1	-0.691	-1.61	2.79E-07	6.55E-06
204557_s_at	DZIP1	-0.691	-1.61	2.18E-04	1.36E-03
1559566_at	FBXO42	-0.692	-1.62	2.68E-05	2.53E-04
215434_x_at	NBPF1	-0.692	-1.62	3.76E-04	2.08E-03
1558794_at	NUTM2A-AS1	-0.692	-1.62	2.13E-04	1.33E-03
205586_x_at	VGF	-0.692	-1.62	1.52E-09	9.67E-08

210756_s_at	NOTCH2	-0.694	-1.62	3.64E-04	2.03E-03
214474_at	LOC101060511 /// PRKAB2	-0.695	-1.62	1.58E-03	6.49E-03
215952_s_at	OAZ1	-0.695	-1.62	8.95E-05	6.63E-04
1553072_at	BNIPL	-0.695	-1.62	7.76E-05	5.91E-04
228293_at	DEPDC7	-0.696	-1.62	5.81E-07	1.15E-05
221579_s_at	NUDT3 /// RPS10-NUDT3	-0.696	-1.62	2.37E-05	2.29E-04
207511_s_at	CNPPD1	-0.696	-1.62	3.31E-07	7.43E-06
203764_at	DLGAP5	-0.697	-1.62	1.79E-04	1.16E-03
218192_at	IP6K2	-0.697	-1.62	7.41E-06	8.94E-05
207826_s_at	ID3	-0.697	-1.62	5.31E-09	2.75E-07
1552620_at	SPRR4	-0.697	-1.62	3.31E-05	2.98E-04
203790_s_at	HRSP12	-0.697	-1.62	5.18E-07	1.05E-05
219858_s_at	MFSD6	-0.697	-1.62	1.42E-03	5.98E-03
244435_at	FAM196A	-0.697	-1.62	5.65E-05	4.57E-04
229765_at	ZNF207	-0.698	-1.62	2.63E-03	9.80E-03
239136_at	UNC5B-AS1	-0.698	-1.62	8.84E-03	2.60E-02
236264_at	LPHN3	-0.699	-1.62	3.06E-05	2.80E-04
1558577_at	LOC148709	-0.699	-1.62	7.65E-09	3.67E-07
1555334_s_at	SLC30A5	-0.699	-1.62	1.43E-06	2.40E-05
208899_x_at	ATP6V1D	-0.699	-1.62	1.80E-07	4.63E-06
209890_at	TSPAN5	-0.699	-1.62	1.92E-05	1.93E-04
230674_at	LGR4	-0.7	-1.62	1.37E-05	1.47E-04
219367_s_at	NRP2	-0.7	-1.62	1.44E-03	6.05E-03

1568574_x_at	SPP1	-0.7	-1.62	1.14E-02	3.18E-02
1555401_at	SOHLH2	-0.7	-1.62	4.63E-07	9.62E-06
243109_at	MCTP2	-0.701	-1.63	5.48E-05	4.45E-04
208633_s_at	MACF1	-0.701	-1.63	3.09E-09	1.76E-07
1552575_a_at	C6orf141	-0.701	-1.63	2.15E-05	2.12E-04
221234_s_at	BACH2	-0.701	-1.63	3.02E-06	4.38E-05
210020_x_at	CALML3	-0.702	-1.63	5.81E-09	2.93E-07
1559256_at	MAGI1	-0.702	-1.63	5.06E-07	1.03E-05
213476_x_at	TUBB3	-0.702	-1.63	5.31E-10	4.13E-08
201340_s_at	ENC1	-0.702	-1.63	6.17E-08	2.00E-06
238519_at	DDI2 /// RSC1A1	-0.702	-1.63	1.28E-02	3.48E-02
239635_at	RBM14	-0.702	-1.63	8.78E-05	6.53E-04
233539_at	NAPEPLD	-0.703	-1.63	1.92E-04	1.22E-03
235315_at	TSC22D1	-0.703	-1.63	1.09E-05	1.22E-04
242210_at	ZNF24	-0.703	-1.63	8.82E-05	6.55E-04
214036_at	EFNA5	-0.703	-1.63	4.09E-08	1.42E-06
202818_s_at	TCEB3	-0.703	-1.63	3.64E-05	3.22E-04
37950_at	PREP	-0.704	-1.63	5.07E-09	2.64E-07
244118_at	GABRA1	-0.704	-1.63	1.73E-06	2.80E-05
215807_s_at	PLXNB1	-0.704	-1.63	1.19E-04	8.36E-04
212081_x_at	PRRC2A	-0.705	-1.63	7.30E-06	8.82E-05
210317_s_at	YWHAE	-0.706	-1.63	4.24E-04	2.29E-03
212570_at	ENDOD1	-0.706	-1.63	1.28E-04	8.88E-04

239937_at	ZNF207	-0.707	-1.63	2.51E-04	1.51E-03
1553976_a_at	DPCD	-0.708	-1.63	5.02E-06	6.57E-05
226592_at	ZNF618	-0.708	-1.63	3.43E-07	7.62E-06
227313_at	CNPY4	-0.708	-1.63	2.27E-08	8.92E-07
229093_at	NOS3	-0.708	-1.63	1.06E-06	1.88E-05
213887_s_at	POLR2E	-0.709	-1.63	3.86E-07	8.38E-06
240282_at	WDR1	-0.709	-1.63	1.99E-04	1.26E-03
1552496_a_at	COBL	-0.709	-1.64	7.04E-05	5.45E-04
203100_s_at	CDYL	-0.709	-1.64	9.14E-05	6.75E-04
201167_x_at	ARHGDIA	-0.71	-1.64	1.15E-04	8.13E-04
224602_at	C4orf3	-0.71	-1.64	1.45E-05	1.53E-04
212098_at	MGAT5	-0.711	-1.64	5.00E-13	1.17E-10
228553_at	ENAH	-0.712	-1.64	4.29E-05	3.67E-04
222111_at	FAM63B	-0.712	-1.64	9.54E-05	6.97E-04
1564064_a_at	ATP11B	-0.712	-1.64	3.66E-06	5.11E-05
231866_at	LNPEP	-0.712	-1.64	7.34E-03	2.24E-02
1555945_s_at	FAM120A	-0.713	-1.64	2.22E-05	2.18E-04
223775_at	HHIP	-0.713	-1.64	1.23E-05	1.35E-04
204556_s_at	DZIP1	-0.713	-1.64	3.85E-04	2.12E-03
243835_at	ZDHHC21	-0.713	-1.64	6.53E-06	8.05E-05
205016_at	TGFA	-0.713	-1.64	1.47E-09	9.50E-08
212481_s_at	TPM4	-0.715	-1.64	1.21E-09	8.12E-08
1558093_s_at	MATR3 /// SNHG4	-0.715	-1.64	3.59E-04	2.00E-03

236621_at	RPS27	-0.715	-1.64	1.43E-05	1.51E-04
243643_x_at	SLC30A6	-0.715	-1.64	1.39E-05	1.48E-04
204256_at	ELOVL6	-0.715	-1.64	2.16E-14	8.31E-12
236402_at	BRAF	-0.715	-1.64	3.91E-06	5.40E-05
223539_s_at	SERF1A /// SERF1B	-0.715	-1.64	2.82E-05	2.62E-04
231705_at	HRSP12	-0.716	-1.64	1.87E-05	1.90E-04
202503_s_at	KIAA0101	-0.716	-1.64	4.48E-06	6.01E-05
201109_s_at	THBS1	-0.716	-1.64	1.10E-06	1.94E-05
1556021_at	GPR180	-0.717	-1.64	5.92E-04	2.97E-03
229274_at	GNAS	-0.717	-1.64	4.89E-04	2.56E-03
202316_x_at	UBE4B	-0.717	-1.64	1.43E-04	9.69E-04
234321_x_at	NHSL1	-0.718	-1.64	5.55E-12	9.04E-10
229574_at	TRA2A	-0.718	-1.64	6.50E-03	2.03E-02
238594_x_at	DUSP8	-0.718	-1.64	2.04E-04	1.28E-03
1558378_a_at	AHNAK2	-0.718	-1.65	1.26E-02	3.46E-02
212365_at	MYO1B	-0.718	-1.65	7.67E-08	2.36E-06
213087_s_at	EEF1D	-0.719	-1.65	5.30E-04	2.72E-03
1565716_at	FUS	-0.72	-1.65	6.57E-04	3.23E-03
237333_at	RBBP4	-0.72	-1.65	7.79E-03	2.36E-02
201521_s_at	NCBP2	-0.72	-1.65	1.30E-08	5.52E-07
238621_at	FMN1	-0.721	-1.65	3.10E-05	2.83E-04
240868_at	LOC100129406	-0.722	-1.65	4.53E-04	2.41E-03
215547_at	TSC22D2	-0.722	-1.65	1.41E-08	5.94E-07

223821_s_at	SUSD4	-0.724	-1.65	6.87E-05	5.33E-04
1559174_at	RAB6A	-0.724	-1.65	6.48E-05	5.09E-04
1553226_at	LINC00052	-0.725	-1.65	3.24E-05	2.93E-04
209193_at	PIM1	-0.725	-1.65	2.26E-07	5.59E-06
229770_at	GLT1D1	-0.726	-1.65	4.28E-05	3.67E-04
209162_s_at	PRPF4	-0.727	-1.66	1.52E-06	2.51E-05
210186_s_at	FKBP1A	-0.728	-1.66	1.63E-05	1.69E-04
1553352_x_at	ERVW-1	-0.728	-1.66	1.91E-09	1.19E-07
226817_at	DSC2	-0.729	-1.66	6.54E-10	4.98E-08
234418_x_at	CD44	-0.729	-1.66	3.01E-06	4.38E-05
216554_s_at	ENO1	-0.73	-1.66	1.45E-03	6.08E-03
226905_at	FAM101B	-0.73	-1.66	2.37E-06	3.61E-05
201291_s_at	TOP2A	-0.73	-1.66	1.12E-04	7.98E-04
221499_s_at	STX16	-0.73	-1.66	1.28E-04	8.90E-04
200054_at	ZNF259	-0.731	-1.66	2.41E-12	4.41E-10
212095_s_at	MTUS1	-0.731	-1.66	1.27E-04	8.85E-04
212364_at	MYO1B	-0.732	-1.66	2.74E-06	4.07E-05
227602_at	RAB7A	-0.734	-1.66	4.09E-04	2.22E-03
228485_s_at	SLC44A1	-0.734	-1.66	2.08E-06	3.24E-05
201075_s_at	SMARCC1	-0.734	-1.66	3.07E-06	4.44E-05
202936_s_at	SOX9	-0.735	-1.66	1.57E-07	4.17E-06
232498_at	LOC377711 /// MROH1	-0.735	-1.66	5.76E-04	2.91E-03
214297_at	CSPG4	-0.736	-1.66	1.24E-03	5.38E-03
223254_s_at	G2E3	-0.73	-1.67	1.36E-03	5.80E-03

		7			
209344_at	TPM4	-0.737	-1.67	7.92E-07	1.49E-05
238346_s_at	TGS1	-0.737	-1.67	1.19E-04	8.38E-04
209589_s_at	EPHB2	-0.737	-1.67	7.06E-07	1.35E-05
220011_at	AUNIP	-0.738	-1.67	3.82E-05	3.34E-04
218916_at	ZNF768	-0.738	-1.67	2.23E-06	3.44E-05
203935_at	ACVR1	-0.739	-1.67	1.28E-08	5.47E-07
213033_s_at	NFIB	-0.74	-1.67	5.78E-04	2.92E-03
220977_x_at	EPB41L5	-0.74	-1.67	2.26E-05	2.21E-04
226193_x_at	CBWD1 /// CBWD3 /// CBWD5 /// CBWD6 /// CBWD7 /// LOC100653334 /// LOC101060578	-0.74	-1.67	2.85E-06	4.19E-05
1556425_a_at	LOC284219	-0.74	-1.67	5.57E-09	2.85E-07
205990_s_at	WNT5A	-0.741	-1.67	2.99E-08	1.12E-06
224940_s_at	PAPPA	-0.741	-1.67	2.05E-05	2.03E-04
202272_s_at	FBXO28	-0.742	-1.67	2.90E-08	1.09E-06
232112_at	RALGPS2	-0.742	-1.67	4.53E-08	1.54E-06
214446_at	ELL2	-0.742	-1.67	1.51E-06	2.51E-05
227510_x_at	MALAT1	-0.742	-1.67	2.29E-07	5.63E-06
202458_at	PRSS23	-0.742	-1.67	2.37E-07	5.78E-06
201617_x_at	CALD1	-0.743	-1.67	1.98E-06	3.12E-05
200604_s_at	PRKAR1A	-0.744	-1.67	2.77E-06	4.10E-05
201860_s_at	PLAT	-0.744	-1.67	1.23E-05	1.35E-04
225647_s_at	CTSC	-0.745	-1.68	1.22E-05	1.34E-04
212022_s_at	MKI67	-0.745	-1.68	7.77E-06	9.29E-05
37408_at	MRC2	-0.745	-1.68	1.74E-09	1.10E-07

222311_s_at	SCAF4	-0.746	-1.68	4.47E-05	3.79E-04
242786_at	SBF2-AS1	-0.746	-1.68	1.61E-06	2.63E-05
208002_s_at	ACOT7	-0.746	-1.68	1.63E-11	2.24E-09
201793_x_at	SMG7	-0.746	-1.68	3.41E-05	3.04E-04
222372_at	MAGI1	-0.747	-1.68	3.99E-04	2.18E-03
238929_at	SRSF8	-0.748	-1.68	4.01E-06	5.51E-05
213439_x_at	RUNDC3A	-0.748	-1.68	3.53E-08	1.27E-06
205732_s_at	NCOA2	-0.748	-1.68	1.13E-05	1.26E-04
220317_at	LRAT	-0.749	-1.68	5.08E-07	1.03E-05
239043_at	ZNF404	-0.749	-1.68	1.96E-04	1.24E-03
237106_at	SLC11A2	-0.75	-1.68	8.41E-05	6.31E-04
205007_s_at	CIB2	-0.75	-1.68	3.84E-08	1.35E-06
215139_at	ARHGEF10	-0.75	-1.68	3.82E-05	3.34E-04
237968_at	ATL2	-0.75	-1.68	1.33E-05	1.43E-04
215195_at	PRKCA	-0.75	-1.68	6.99E-05	5.41E-04
220512_at	DLC1	-0.75	-1.68	5.39E-04	2.76E-03
220482_s_at	SERGEF	-0.752	-1.68	5.68E-05	4.59E-04
220613_s_at	SYTL2	-0.752	-1.68	3.39E-10	2.85E-08
215443_at	TSHR	-0.752	-1.68	6.49E-05	5.10E-04
229518_at	FAM46B	-0.754	-1.69	3.22E-10	2.75E-08
227173_s_at	BACH2	-0.754	-1.69	6.52E-06	8.04E-05
205687_at	UBFD1	-0.754	-1.69	1.51E-07	4.04E-06
202066_at	PPFIA1	-0.754	-1.69	3.15E-13	7.88E-11
1560172_at	INTS10	-0.754	-1.69	1.17E-03	5.13E-03

1564383_s_at	FLJ35934	-0.754	-1.69	8.87E-04	4.10E-03
226069_at	PRICKLE1	-0.755	-1.69	1.89E-07	4.81E-06
207112_s_at	GAB1	-0.755	-1.69	1.19E-08	5.18E-07
226494_at	CAMSAP3	-0.757	-1.69	7.25E-07	1.39E-05
211793_s_at	ABI2	-0.757	-1.69	9.15E-11	9.55E-09
1561030_at	TMC7	-0.758	-1.69	9.41E-05	6.89E-04
242422_at	G3BP1	-0.758	-1.69	9.71E-04	4.42E-03
1555460_a_at	SLC39A6	-0.758	-1.69	2.23E-05	2.19E-04
211160_x_at	ACTN1	-0.758	-1.69	1.72E-06	2.78E-05
222386_s_at	COPZ1	-0.758	-1.69	1.64E-08	6.74E-07
204148_s_at	POMZP3 /// ZP3	-0.759	-1.69	7.50E-04	3.58E-03
205472_s_at	DACH1	-0.759	-1.69	4.18E-05	3.60E-04
219916_s_at	RNF39	-0.759	-1.69	8.33E-10	5.98E-08
206008_at	TGM1	-0.76	-1.69	1.95E-06	3.08E-05
219229_at	SLCO3A1	-0.76	-1.69	3.79E-10	3.14E-08
213423_x_at	TUSC3	-0.761	-1.69	2.54E-05	2.42E-04
1566538_at	MAGI1	-0.761	-1.7	1.58E-05	1.65E-04
232350_x_at	GPR161	-0.762	-1.7	2.32E-10	2.07E-08
205948_at	PTPRT	-0.762	-1.7	7.83E-04	3.71E-03
243361_at	SREK1	-0.763	-1.7	8.26E-04	3.87E-03
213406_at	WSB1	-0.764	-1.7	2.36E-05	2.29E-04
231548_at	FOXO3	-0.765	-1.7	4.41E-05	3.75E-04
209118_s_at	TUBA1A	-0.766	-1.7	3.77E-11	4.45E-09

229004_at	ADAMTS15	-0.766	-1.7	8.87E-05	6.58E-04
224754_at	SP1	-0.767	-1.7	1.84E-03	7.33E-03
220203_at	BMP8A	-0.768	-1.7	5.91E-09	2.98E-07
230229_at	DLG1	-0.768	-1.7	1.01E-05	1.15E-04
230708_at	PRICKLE1	-0.768	-1.7	2.89E-07	6.74E-06
218834_s_at	TMEM132A	-0.768	-1.7	5.15E-07	1.04E-05
231907_at	ABL2	-0.769	-1.7	3.90E-03	1.35E-02
220431_at	TMPRSS11E	-0.769	-1.7	3.53E-03	1.24E-02
200622_x_at	CALM1 /// CALM2 /// CALM3	-0.769	-1.7	3.44E-06	4.86E-05
202207_at	ARL4C	-0.77	-1.7	1.52E-08	6.30E-07
208719_s_at	DDX17	-0.77	-1.71	1.74E-06	2.81E-05
227300_at	TMEM119	-0.771	-1.71	9.30E-05	6.83E-04
219341_at	CLN8	-0.771	-1.71	3.20E-05	2.90E-04
207764_s_at	HIPK3	-0.772	-1.71	3.27E-08	1.19E-06
201861_s_at	LRRFIP1	-0.773	-1.71	7.73E-05	5.89E-04
201229_s_at	ARIH2	-0.773	-1.71	2.89E-05	2.68E-04
228527_s_at	SLC25A37	-0.773	-1.71	1.37E-07	3.75E-06
226899_at	UNC5B	-0.775	-1.71	1.96E-06	3.08E-05
242021_at	XBP1	-0.776	-1.71	2.37E-06	3.60E-05
37425_g_at	CCHCR1	-0.776	-1.71	7.61E-08	2.35E-06
222927_s_at	CPLX3	-0.777	-1.71	3.89E-09	2.12E-07
1569053_at	AP3M2	-0.777	-1.71	1.26E-04	8.80E-04
232348_at	C18orf8	-0.777	-1.71	3.35E-10	2.83E-08

220021_at	TMC7	-0.777	-1.71	1.33E-07	3.65E-06
208932_at	PPP4C	-0.778	-1.71	5.18E-04	2.67E-03
242583_at	STON2	-0.778	-1.71	1.04E-06	1.85E-05
212468_at	SPAG9	-0.779	-1.72	4.85E-03	1.61E-02
238028_at	C6orf132	-0.779	-1.72	6.04E-07	1.19E-05
1556037_s_at	HHIP	-0.779	-1.72	4.32E-06	5.85E-05
205139_s_at	UST	-0.78	-1.72	5.51E-11	6.07E-09
204173_at	MYL6B	-0.78	-1.72	2.51E-07	6.02E-06
218113_at	TMEM2	-0.78	-1.72	3.01E-09	1.73E-07
232705_at	LRRFIP2	-0.781	-1.72	1.26E-03	5.44E-03
208653_s_at	CD164	-0.781	-1.72	9.12E-07	1.66E-05
205195_at	AP1S1	-0.781	-1.72	2.10E-05	2.08E-04
202391_at	BASP1	-0.782	-1.72	3.16E-09	1.78E-07
213606_s_at	ARHGDIA	-0.782	-1.72	5.83E-05	4.67E-04
227953_at	CMTM6	-0.783	-1.72	5.90E-05	4.72E-04
1559964_at	FLJ38717	-0.783	-1.72	8.86E-04	4.10E-03
230976_at	AK8	-0.783	-1.72	2.21E-04	1.37E-03
228699_at	NRP2	-0.783	-1.72	1.42E-06	2.39E-05
1562271_x_at	ARHGEF7	-0.783	-1.72	9.34E-04	4.28E-03
206605_at	ENDOU	-0.783	-1.72	9.51E-10	6.60E-08
214359_s_at	HSP90AB1	-0.784	-1.72	1.86E-03	7.42E-03
209882_at	RIT1	-0.784	-1.72	8.65E-07	1.59E-05
209198_s_at	SYT11	-0.785	-1.72	2.96E-04	1.72E-03

215269_at	TRAPPC10	-0.785	-1.72	5.73E-05	4.62E-04
213577_at	SQLE	-0.786	-1.72	1.46E-05	1.54E-04
243759_at	SCAF4	-0.787	-1.73	1.89E-02	4.78E-02
1557384_at	LOC100506639 /// ZNF131	-0.787	-1.73	1.39E-03	5.88E-03
233203_at	ROPN1	-0.788	-1.73	2.40E-04	1.46E-03
222528_s_at	SLC25A37	-0.788	-1.73	6.73E-07	1.30E-05
212841_s_at	PPFIBP2	-0.788	-1.73	2.08E-11	2.79E-09
231403_at	TRIO	-0.788	-1.73	1.06E-04	7.60E-04
219687_at	HHAT	-0.788	-1.73	2.39E-11	3.09E-09
209078_s_at	TXN2	-0.788	-1.73	4.08E-05	3.54E-04
229694_at	WDR11	-0.788	-1.73	8.84E-03	2.60E-02
1553749_at	FAM76B	-0.789	-1.73	1.95E-04	1.24E-03
202345_s_at	FABP5 /// LOC101060453	-0.789	-1.73	8.96E-10	6.32E-08
225478_at	MFHAS1	-0.789	-1.73	9.91E-12	1.47E-09
219987_at	ERVMER34-1	-0.789	-1.73	3.68E-05	3.24E-04
235095_at	CCDC64B	-0.789	-1.73	2.74E-07	6.45E-06
201072_s_at	SMARCC1	-0.789	-1.73	9.50E-05	6.94E-04
243318_at	DCAF8	-0.79	-1.73	2.45E-04	1.48E-03
213517_at	PCBP2	-0.79	-1.73	1.58E-06	2.60E-05
210929_s_at	AHSG	-0.79	-1.73	1.87E-07	4.78E-06
229817_at	ZNF608	-0.791	-1.73	2.65E-07	6.28E-06
236371_s_at	TGS1	-0.792	-1.73	3.01E-04	1.75E-03
238449_at	LOC595101	-0.793	-1.73	1.13E-03	4.98E-03

235977_at	LONRF2	-0.793	-1.73	8.56E-06	1.01E-04
232304_at	PELI1	-0.793	-1.73	3.78E-04	2.09E-03
211571_s_at	VCAN	-0.793	-1.73	1.46E-04	9.86E-04
1557034_s_at	LOC100134445 /// LOC100288778 /// WASH1 /// WASH2P /// WASH3P /// WASH5P /// WASH7P	-0.794	-1.73	6.21E-04	3.09E-03
203221_at	TLE1	-0.794	-1.73	3.69E-11	4.39E-09
217649_at	ZFAND5	-0.795	-1.73	7.24E-06	8.76E-05
1559658_at	KATNBL1	-0.795	-1.74	3.31E-06	4.71E-05
212103_at	KPNA6	-0.795	-1.74	3.11E-05	2.84E-04
242458_at	RALGPS2	-0.796	-1.74	5.65E-07	1.12E-05
1558028_x_at	LINC00657	-0.796	-1.74	2.36E-05	2.29E-04
219340_s_at	CLN8	-0.798	-1.74	6.89E-10	5.15E-08
204761_at	USP6NL	-0.798	-1.74	6.48E-11	7.01E-09
213344_s_at	H2AFX	-0.798	-1.74	2.36E-09	1.41E-07
221881_s_at	CLIC4	-0.798	-1.74	1.22E-05	1.34E-04
201939_at	PLK2	-0.8	-1.74	8.12E-07	1.51E-05
241669_x_at	PRKD2	-0.8	-1.74	1.85E-04	1.19E-03
222146_s_at	TCF4	-0.801	-1.74	3.10E-06	4.47E-05
228977_at	LOC729680	-0.801	-1.74	4.54E-06	6.08E-05
212144_at	SUN2	-0.801	-1.74	8.52E-09	3.97E-07
213272_s_at	TMEM159	-0.801	-1.74	2.25E-05	2.20E-04
219478_at	WFDC1	-0.802	-1.74	1.55E-03	6.40E-03
52651_at	COL8A2	-0.802	-1.74	2.21E-07	5.47E-06
215855_s_at	TMF1	-0.803	-1.74	3.24E-05	2.93E-04

222810_s_at	RASAL2	-0.803	-1.74	9.83E-06	1.13E-04
212013_at	PXDN	-0.804	-1.75	9.36E-06	1.08E-04
223478_at	TIMM8B	-0.804	-1.75	1.07E-05	1.20E-04
209288_s_at	CDC42EP3	-0.804	-1.75	1.93E-08	7.74E-07
232213_at	PELI1	-0.804	-1.75	2.50E-05	2.40E-04
209127_s_at	SART3	-0.804	-1.75	2.16E-05	2.13E-04
1555007_s_at	WDR66	-0.805	-1.75	9.54E-07	1.72E-05
226026_at	DIRC2	-0.805	-1.75	4.81E-11	5.45E-09
213478_at	KAZN	-0.805	-1.75	7.31E-06	8.84E-05
225950_at	SAMD8	-0.806	-1.75	3.84E-11	4.52E-09
231873_at	BMPR2	-0.806	-1.75	1.28E-04	8.87E-04
1553185_at	RASEF	-0.807	-1.75	1.69E-08	6.88E-07
226920_at	CSNK1A1	-0.807	-1.75	4.34E-04	2.33E-03
208577_at	HIST1H3A /// HIST1H3B /// HIST1H3C /// HIST1H3D /// HIST1H3E /// HIST1H3F /// HIST1H3G /// HIST1H3H /// HIST1H3I /// HIST1H3J	-0.807	-1.75	2.75E-04	1.62E-03
201693_s_at	EGR1	-0.807	-1.75	3.02E-04	1.75E-03
220456_at	SPTLC3	-0.807	-1.75	2.04E-09	1.25E-07
205150_s_at	TRIL	-0.807	-1.75	2.59E-05	2.46E-04
211362_s_at	SERPINB13	-0.808	-1.75	4.93E-09	2.57E-07
242512_at	MYO9A	-0.808	-1.75	1.60E-04	1.06E-03
226651_at	HOMER1	-0.808	-1.75	1.23E-08	5.29E-07
209280_at	MRC2	-0.81	-1.75	5.28E-10	4.12E-08
224397_s_at	TMTC1	-0.81	-1.75	1.79E-06	2.87E-05
224851_at	CDK6	-0.81	-1.75	4.73E-04	2.50E-03

232864_s_at	AFF4	-0.81	-1.75	1.45E-05	1.54E-04
212417_at	SCAMP1	-0.811	-1.75	4.08E-04	2.22E-03
230464_at	S1PR5	-0.811	-1.75	8.41E-06	9.92E-05
209793_at	GRIA1	-0.812	-1.76	4.52E-03	1.52E-02
202378_s_at	LEPROT	-0.813	-1.76	5.41E-04	2.77E-03
41856_at	UNC5B	-0.814	-1.76	3.39E-07	7.57E-06
209125_at	KRT6A	-0.814	-1.76	3.77E-08	1.33E-06
213999_at	YIPF4	-0.814	-1.76	1.81E-06	2.89E-05
209873_s_at	PKP3	-0.815	-1.76	1.83E-07	4.69E-06
241990_at	RHOV	-0.815	-1.76	3.48E-07	7.73E-06
212800_at	STX6	-0.815	-1.76	6.30E-09	3.12E-07
1568126_at	ANXA2	-0.816	-1.76	3.55E-05	3.14E-04
217871_s_at	MIF	-0.816	-1.76	3.11E-05	2.83E-04
220816_at	LPAR3	-0.816	-1.76	2.02E-08	8.05E-07
217294_s_at	ENO1	-0.818	-1.76	1.59E-03	6.52E-03
220489_s_at	SERINC2	-0.818	-1.76	2.97E-07	6.90E-06
218484_at	NDUFA4L2	-0.819	-1.76	1.03E-04	7.43E-04
227461_at	STON2	-0.819	-1.76	3.57E-12	6.25E-10
216629_at	SRRM2	-0.819	-1.76	2.73E-05	2.56E-04
229929_at	SPSB4	-0.819	-1.76	8.32E-09	3.91E-07
1556423_at	VASH1	-0.819	-1.76	1.39E-05	1.48E-04
221487_s_at	ENSA	-0.819	-1.76	5.98E-08	1.95E-06
232360_at	EHF	-0.81	-1.76	3.48E-03	1.23E-02

		9			
1565939_at	C5orf22	-0.819	-1.76	6.43E-05	5.06E-04
228998_at	TNRC6B	-0.819	-1.76	2.72E-05	2.56E-04
203692_s_at	E2F3	-0.819	-1.76	1.23E-06	2.13E-05
219806_s_at	SMCO4	-0.82	-1.77	9.49E-08	2.77E-06
210419_at	BARX2	-0.82	-1.77	1.57E-11	2.18E-09
208978_at	CRIP2	-0.821	-1.77	5.22E-07	1.05E-05
228126_x_at	CTXN1	-0.822	-1.77	2.72E-08	1.03E-06
213096_at	TMCC2	-0.822	-1.77	1.04E-12	2.22E-10
215199_at	CALD1	-0.823	-1.77	3.03E-06	4.39E-05
226071_at	ADAMTSL4	-0.823	-1.77	8.70E-05	6.48E-04
221539_at	EIF4EBP1	-0.824	-1.77	2.18E-11	2.87E-09
201266_at	TXNRD1	-0.826	-1.77	4.21E-14	1.38E-11
214805_at	EIF4A1 /// SNORA48 /// SNORA67 /// SNORD10	-0.826	-1.77	8.13E-05	6.14E-04
204818_at	HSD17B2	-0.828	-1.77	3.92E-04	2.15E-03
222701_s_at	CHCHD7	-0.828	-1.77	9.58E-11	9.95E-09
204654_s_at	TFAP2A	-0.829	-1.78	1.01E-05	1.15E-04
213100_at	UNC5B	-0.829	-1.78	1.22E-06	2.12E-05
205187_at	SMAD5	-0.829	-1.78	1.66E-03	6.76E-03
201986_at	MED13	-0.83	-1.78	4.07E-05	3.53E-04
234923_at	RALGAPA1	-0.83	-1.78	8.14E-06	9.66E-05
214829_at	AASS	-0.831	-1.78	1.37E-05	1.46E-04
226187_at	CDS1	-0.832	-1.78	1.02E-11	1.50E-09
1569320_at	GPBP1L1	-0.832	-1.78	7.31E-05	5.62E-04

206156_at	GJB5	-0.832	-1.78	6.91E-05	5.36E-04
201737_s_at	6-Mar	-0.834	-1.78	3.15E-04	1.81E-03
211672_s_at	ARPC4	-0.834	-1.78	2.61E-04	1.55E-03
1559881_s_at	ZNF12	-0.834	-1.78	7.53E-06	9.07E-05
1553349_at	ARID2	-0.836	-1.78	1.69E-05	1.74E-04
221771_s_at	MPHOSPH8	-0.836	-1.79	5.98E-05	4.77E-04
235367_at	MYPN	-0.836	-1.79	1.74E-12	3.38E-10
223940_x_at	MALAT1	-0.837	-1.79	1.98E-09	1.22E-07
240144_at	DNASE1	-0.837	-1.79	7.82E-06	9.34E-05
228040_at	MGC21881	-0.837	-1.79	1.17E-05	1.30E-04
222164_at	FGFR1	-0.838	-1.79	4.50E-09	2.38E-07
203987_at	FZD6	-0.838	-1.79	1.35E-10	1.31E-08
214119_s_at	FKBP1A	-0.838	-1.79	9.12E-08	2.69E-06
213038_at	RNF19B	-0.839	-1.79	4.90E-11	5.54E-09
239899_at	RNF145	-0.84	-1.79	5.92E-07	1.16E-05
201183_s_at	CHD4	-0.84	-1.79	9.49E-06	1.10E-04
206114_at	EPHA4	-0.841	-1.79	6.13E-05	4.87E-04
209457_at	DUSP5	-0.841	-1.79	1.99E-07	5.03E-06
205209_at	ACVR1B	-0.842	-1.79	3.38E-04	1.91E-03
203634_s_at	CPT1A	-0.842	-1.79	1.41E-05	1.50E-04
1565703_at	SMAD4	-0.844	-1.79	4.30E-05	3.68E-04
243611_at	MICALCL	-0.844	-1.79	2.66E-08	1.01E-06
213425_at	WNT5A	-0.844	-1.8	2.24E-09	1.35E-07

224854_s_at	SLAIN2	-0.845	-1.8	5.45E-07	1.09E-05
201645_at	TNC	-0.845	-1.8	1.98E-06	3.12E-05
226541_at	FBXO30	-0.846	-1.8	2.39E-10	2.13E-08
238001_at	KCTD6	-0.846	-1.8	1.14E-08	5.02E-07
217238_s_at	ALDOB	-0.847	-1.8	1.13E-08	5.00E-07
202416_at	DNAJC7	-0.847	-1.8	9.59E-11	9.95E-09
1553186_x_at	RASEF	-0.848	-1.8	8.56E-08	2.57E-06
209591_s_at	BMP7	-0.848	-1.8	6.08E-06	7.64E-05
208908_s_at	CAST	-0.848	-1.8	3.47E-10	2.92E-08
232249_at	FMNL3	-0.849	-1.8	3.13E-05	2.85E-04
1565717_s_at	FUS	-0.85	-1.8	7.15E-05	5.52E-04
210655_s_at	FOXO3 /// FOXO3B	-0.851	-1.8	3.81E-08	1.34E-06
1558387_at	UG0898H09	-0.851	-1.8	1.42E-04	9.66E-04
239913_at	SLC10A4	-0.852	-1.8	1.54E-07	4.12E-06
225852_at	ANKRD17	-0.853	-1.81	5.89E-05	4.71E-04
1555225_at	C1orf43	-0.854	-1.81	1.25E-07	3.48E-06
231439_at	FAM84A	-0.854	-1.81	4.18E-08	1.44E-06
231108_at	FUS	-0.854	-1.81	3.41E-04	1.92E-03
230369_at	GPR161	-0.855	-1.81	3.31E-10	2.81E-08
203016_s_at	SSX2IP	-0.855	-1.81	3.64E-12	6.34E-10
244682_at	CAMSAP1	-0.855	-1.81	1.68E-05	1.74E-04
242881_x_at	LOC100506303 /// LOC100653149 /// LOC101060483 /// LOC400879 /// LOC440157	-0.856	-1.81	2.87E-03	1.05E-02
224559_at	MALAT1	-0.85	-1.81	1.28E-06	2.19E-05

		6			
1554671_a_at	SRRM2	-0.857	-1.81	1.55E-04	1.03E-03
1569126_at	CCNC	-0.857	-1.81	2.06E-05	2.05E-04
210910_s_at	POMZP3	-0.858	-1.81	5.49E-04	2.81E-03
202208_s_at	ARL4C	-0.858	-1.81	4.00E-10	3.26E-08
224325_at	FZD8	-0.859	-1.81	2.57E-04	1.54E-03
1552334_at	TRIOBP	-0.859	-1.81	2.22E-06	3.43E-05
239707_at	SLC5A10	-0.859	-1.81	1.33E-11	1.90E-09
1567107_s_at	TPM4	-0.859	-1.81	3.11E-08	1.15E-06
206315_at	CRLF1	-0.86	-1.81	5.18E-10	4.07E-08
232537_x_at	MARK3	-0.86	-1.82	3.58E-04	2.00E-03
1569519_at	LOC101059961 /// LOC101060202 /// LOC101060226 /// LOC101060362 /// LOC101060684 /// NBPF1 /// NBPF10 /// NBPF11 /// NBPF12 /// NBPF24 /// NBPF7 /// NBPF8 /// NBPF9	-0.861	-1.82	8.36E-07	1.54E-05
228786_at	PTCHD3P1	-0.861	-1.82	4.05E-06	5.54E-05
204117_at	PREP	-0.861	-1.82	6.04E-11	6.59E-09
230830_at	SLC51B	-0.861	-1.82	7.04E-10	5.22E-08
1568617_a_at	CAMSAP3	-0.862	-1.82	1.78E-06	2.86E-05
239062_at	TNRC6C-AS1	-0.862	-1.82	1.62E-07	4.27E-06
240383_at	UBE2D3	-0.863	-1.82	7.99E-06	9.52E-05
236696_at	U2SURP	-0.863	-1.82	5.40E-03	1.76E-02
205918_at	SLC4A3	-0.863	-1.82	1.04E-05	1.18E-04
217591_at	SKIL	-0.864	-1.82	2.38E-07	5.80E-06
207011_s_at	PTK7	-0.865	-1.82	6.85E-08	2.18E-06
217897_at	FXYD6	-0.866	-1.82	3.56E-06	5.00E-05

219869_s_at	SLC39A8	-0.866	-1.82	3.70E-05	3.26E-04
225830_at	PDZD8	-0.868	-1.83	5.38E-11	5.96E-09
243496_at	RAB18	-0.868	-1.83	2.05E-06	3.21E-05
230028_at	KIAA0907	-0.869	-1.83	3.51E-06	4.95E-05
204750_s_at	DSC2	-0.869	-1.83	3.17E-07	7.22E-06
220067_at	SPTBN5	-0.869	-1.83	5.75E-05	4.62E-04
215646_s_at	VCAN	-0.869	-1.83	3.78E-04	2.09E-03
220244_at	LINC00312	-0.869	-1.83	1.16E-05	1.28E-04
210074_at	CTSL2	-0.869	-1.83	6.26E-05	4.96E-04
237466_s_at	HHIP	-0.869	-1.83	2.86E-07	6.68E-06
203021_at	SLPI	-0.87	-1.83	3.74E-08	1.33E-06
217608_at	SREK1IP1	-0.87	-1.83	4.66E-05	3.92E-04
215224_at	RPL23 /// SNORA21	-0.872	-1.83	2.72E-03	1.01E-02
222757_s_at	ZAK	-0.872	-1.83	6.93E-06	8.47E-05
241955_at	HECTD1	-0.872	-1.83	2.51E-06	3.78E-05
220022_at	ZNF334	-0.873	-1.83	4.50E-04	2.40E-03
232094_at	KATNBL1	-0.874	-1.83	1.33E-04	9.16E-04
213069_at	HEG1	-0.874	-1.83	5.00E-10	3.95E-08
232168_x_at	MACF1	-0.875	-1.83	2.24E-06	3.44E-05
1557953_at	ZKSCAN1	-0.875	-1.83	3.99E-04	2.18E-03
207268_x_at	ABI2	-0.876	-1.84	4.42E-13	1.06E-10
1553691_at	B3GALNT2	-0.876	-1.84	2.00E-10	1.83E-08
215066_at	PTPRF	-0.876	-1.84	7.78E-06	9.31E-05

211503_s_at	RAB14	-0.876	-1.84	2.57E-05	2.45E-04
242906_at	SESN3	-0.877	-1.84	5.82E-07	1.15E-05
222364_at	SLC44A1	-0.878	-1.84	5.47E-05	4.45E-04
242191_at	NBPF10	-0.878	-1.84	2.16E-04	1.34E-03
209679_s_at	SMAGP	-0.878	-1.84	7.18E-08	2.26E-06
206116_s_at	TPM1	-0.878	-1.84	2.87E-05	2.67E-04
1563497_at	USP25	-0.879	-1.84	6.13E-06	7.69E-05
203904_x_at	CD82	-0.879	-1.84	2.10E-09	1.28E-07
1565525_a_at	TCP11L2	-0.881	-1.84	6.87E-09	3.35E-07
235684_s_at	SESN3	-0.881	-1.84	3.26E-08	1.19E-06
239512_at	SRSF4	-0.882	-1.84	5.54E-04	2.83E-03
222420_s_at	UBE2H	-0.882	-1.84	5.97E-12	9.59E-10
217185_s_at	ZNF259 /// ZNF259P1	-0.883	-1.84	1.11E-08	4.93E-07
207038_at	SLC16A6	-0.885	-1.85	3.47E-08	1.25E-06
204141_at	TUBB2A	-0.887	-1.85	1.33E-17	1.89E-14
220407_s_at	TGFB2	-0.887	-1.85	2.12E-05	2.10E-04
1554703_at	ARHGEF10	-0.887	-1.85	5.56E-06	7.12E-05
1568954_s_at	C16orf72	-0.888	-1.85	6.82E-09	3.34E-07
230622_at	MLLT4	-0.888	-1.85	2.95E-11	3.68E-09
206375_s_at	HSPB3	-0.889	-1.85	7.45E-06	8.97E-05
202695_s_at	STK17A	-0.89	-1.85	1.13E-07	3.21E-06
203779_s_at	MPZL2	-0.89	-1.85	3.11E-08	1.15E-06
1564307_a_at	A2ML1	-0.89	-1.85	1.03E-03	4.63E-03

235275_at	BMP8B	-0.891	-1.86	3.56E-07	7.87E-06
227269_s_at	RAB40C	-0.893	-1.86	5.70E-07	1.13E-05
1569142_at	TRIM13	-0.896	-1.86	3.43E-06	4.85E-05
210716_s_at	CLIP1	-0.896	-1.86	9.81E-07	1.76E-05
1564796_at	EMP1	-0.897	-1.86	1.29E-04	8.95E-04
212157_at	SDC2	-0.897	-1.86	6.48E-06	8.01E-05
213836_s_at	WIPI1	-0.898	-1.86	4.50E-11	5.20E-09
1556204_a_at	ZNF814	-0.899	-1.86	6.17E-07	1.21E-05
205157_s_at	JUP /// KRT17	-0.899	-1.87	7.05E-07	1.35E-05
221803_s_at	NRBF2	-0.899	-1.87	2.63E-12	4.73E-10
222348_at	MAST4	-0.9	-1.87	1.02E-08	4.60E-07
202935_s_at	SOX9	-0.9	-1.87	1.17E-06	2.03E-05
244755_at	XPR1	-0.9	-1.87	1.88E-08	7.54E-07
203722_at	ALDH4A1	-0.902	-1.87	1.06E-09	7.23E-08
220962_s_at	PADI1	-0.902	-1.87	4.40E-12	7.42E-10
36564_at	RNF19B	-0.903	-1.87	2.16E-12	4.05E-10
210338_s_at	HSPA8 /// SNORD14C /// SNORD14D	-0.904	-1.87	2.29E-04	1.41E-03
225119_at	CHMP4B	-0.904	-1.87	1.10E-08	4.87E-07
202089_s_at	SLC39A6	-0.905	-1.87	7.75E-08	2.39E-06
214972_at	MGEA5	-0.906	-1.87	3.47E-05	3.09E-04
225718_at	KIAA1715	-0.906	-1.87	2.41E-03	9.10E-03
201981_at	PAPPA	-0.91	-1.88	1.56E-08	6.48E-07
1558388_a_at	UG0898H09	-0.91	-1.88	3.71E-04	2.06E-03
234925_at	LINC00917	-0.91	-1.88	6.99E-10	5.20E-08

230748_at	SLC16A6	-0.91	-1.88	7.10E-10	5.25E-08
209243_s_at	PEG3	-0.911	-1.88	6.35E-06	7.88E-05
1565759_at	RPL13	-0.912	-1.88	3.74E-08	1.33E-06
203705_s_at	FZD7	-0.912	-1.88	7.20E-07	1.38E-05
220085_at	HELLS	-0.912	-1.88	3.75E-04	2.08E-03
227317_at	LMCD1	-0.913	-1.88	1.61E-06	2.63E-05
201514_s_at	G3BP1	-0.915	-1.89	3.81E-05	3.34E-04
224036_s_at	LMBR1	-0.916	-1.89	1.20E-07	3.37E-06
201105_at	LGALS1	-0.916	-1.89	3.79E-05	3.32E-04
229581_at	ELFN1	-0.917	-1.89	1.72E-07	4.46E-06
228121_at	TGFB2	-0.917	-1.89	2.39E-07	5.82E-06
208650_s_at	CD24	-0.918	-1.89	2.62E-07	6.23E-06
214670_at	ZKSCAN1	-0.919	-1.89	1.41E-03	5.96E-03
227443_at	LURAP1L	-0.919	-1.89	4.98E-10	3.95E-08
224853_at	SLAIN2	-0.92	-1.89	9.05E-06	1.05E-04
206004_at	TGM3	-0.92	-1.89	6.86E-07	1.32E-05
223044_at	SLC40A1	-0.92	-1.89	1.95E-14	7.66E-12
212154_at	SDC2	-0.921	-1.89	9.85E-06	1.13E-04
1556820_a_at	DLEU2	-0.921	-1.89	7.71E-06	9.24E-05
218368_s_at	TNFRSF12A	-0.921	-1.89	1.50E-10	1.43E-08
219369_s_at	OTUB2	-0.922	-1.89	5.82E-08	1.91E-06
242660_at	C10orf112	-0.922	-1.89	8.56E-04	3.98E-03
219660_s_at	ATP8A2	-0.922	-1.89	3.95E-06	5.43E-05
204657_s_at	SHB	-0.92	-1.9	1.50E-09	9.61E-08

		2			
210868_s_at	ELOVL6	-0.922	-1.9	8.61E-13	1.89E-10
204182_s_at	ZBTB43	-0.925	-1.9	2.18E-04	1.35E-03
205534_at	PCDH7	-0.927	-1.9	4.26E-05	3.65E-04
209273_s_at	ISCA1	-0.93	-1.9	3.01E-11	3.73E-09
205635_at	KALRN	-0.93	-1.91	9.96E-07	1.78E-05
201856_s_at	ZFR	-0.931	-1.91	3.90E-10	3.19E-08
226499_at	NRARP	-0.932	-1.91	2.41E-14	9.08E-12
207233_s_at	MITF	-0.932	-1.91	3.71E-07	8.14E-06
200907_s_at	PALLD	-0.933	-1.91	2.99E-09	1.73E-07
220245_at	SLC45A2	-0.933	-1.91	3.19E-06	4.57E-05
210276_s_at	TRIOBP	-0.933	-1.91	4.15E-10	3.36E-08
222833_at	LPCAT2	-0.934	-1.91	5.95E-07	1.17E-05
244758_at	SCAND3	-0.935	-1.91	2.19E-08	8.65E-07
208555_x_at	CST2	-0.936	-1.91	4.38E-13	1.06E-10
218888_s_at	NETO2	-0.936	-1.91	5.04E-07	1.03E-05
238688_at	TPM1	-0.936	-1.91	3.18E-07	7.23E-06
219976_at	HOOK1	-0.937	-1.91	9.49E-09	4.33E-07
223468_s_at	RGMA	-0.937	-1.91	2.32E-06	3.55E-05
201267_s_at	PSMC3	-0.938	-1.92	5.68E-04	2.88E-03
230068_s_at	PEG3-AS1	-0.939	-1.92	1.49E-07	4.01E-06
214697_s_at	PTBP3	-0.94	-1.92	8.47E-08	2.56E-06
1558404_at	LINC00622	-0.94	-1.92	1.86E-06	2.96E-05
222874_s_at	CLN8	-0.94	-1.92	2.55E-11	3.25E-09
228329_at	DAB1	-0.94	-1.92	5.52E-04	2.82E-03

210057_at	LOC101060589 /// SMG1	-0.94	-1.92	1.21E-03	5.26E-03
204730_at	RIMS3	-0.943	-1.92	2.53E-12	4.59E-10
1563900_at	FAM83B	-0.944	-1.92	5.89E-04	2.96E-03
1555730_a_at	CFL1	-0.945	-1.93	2.23E-05	2.18E-04
218980_at	FHOD3	-0.945	-1.93	9.49E-05	6.94E-04
1555074_a_at	KCNH5	-0.946	-1.93	1.93E-05	1.94E-04
1554524_a_at	OLFM3	-0.946	-1.93	4.20E-07	8.90E-06
207192_at	DNASE1L2	-0.947	-1.93	1.56E-06	2.57E-05
215198_s_at	CALD1	-0.948	-1.93	4.18E-09	2.24E-07
231935_at	ARPP21	-0.948	-1.93	8.99E-12	1.37E-09
241771_at	RIMBP2	-0.949	-1.93	3.34E-08	1.21E-06
238775_at	ITFG1	-0.951	-1.93	2.09E-06	3.26E-05
221664_s_at	F11R	-0.952	-1.93	1.46E-10	1.40E-08
202341_s_at	TRIM2	-0.952	-1.93	2.26E-05	2.21E-04
203742_s_at	LOC732360 /// TDG	-0.952	-1.93	1.59E-08	6.59E-07
238513_at	PRRG4	-0.955	-1.94	3.90E-08	1.36E-06
243910_x_at	CAND1	-0.955	-1.94	3.19E-05	2.89E-04
221920_s_at	SLC25A37	-0.956	-1.94	2.67E-08	1.02E-06
1553711_a_at	FAM218A	-0.957	-1.94	8.63E-07	1.58E-05
200824_at	GSTP1	-0.958	-1.94	2.29E-05	2.23E-04
1556316_s_at	LOC284889	-0.959	-1.94	3.46E-06	4.88E-05
201418_s_at	SOX4	-0.962	-1.95	5.91E-06	7.48E-05
209588_at	EPHB2	-0.96	-1.95	6.58E-08	2.10E-06

		2			
219529_at	CLIC3	-0.962	-1.95	2.65E-08	1.01E-06
206376_at	SLC6A15	-0.963	-1.95	5.11E-07	1.04E-05
231259_s_at	CCND2	-0.964	-1.95	9.20E-07	1.67E-05
241245_at	SRSF4	-0.964	-1.95	5.00E-05	4.15E-04
1554804_a_at	CLDN19	-0.966	-1.95	9.63E-09	4.39E-07
208621_s_at	EZR	-0.966	-1.95	2.75E-04	1.62E-03
206137_at	RIMS2	-0.967	-1.96	8.11E-12	1.26E-09
226876_at	FAM101B	-0.967	-1.96	8.11E-08	2.47E-06
225496_s_at	SYTL2	-0.968	-1.96	6.22E-08	2.01E-06
218677_at	S100A14	-0.97	-1.96	9.06E-08	2.68E-06
204320_at	COL11A1	-0.971	-1.96	1.11E-02	3.11E-02
231192_at	LPAR3	-0.972	-1.96	1.52E-16	1.45E-13
231771_at	GJB6	-0.973	-1.96	1.14E-12	2.35E-10
222506_at	LMBR1	-0.974	-1.96	3.54E-06	4.97E-05
232865_at	AFF4	-0.974	-1.96	3.60E-07	7.93E-06
234331_s_at	FAM84A	-0.975	-1.97	1.66E-07	4.35E-06
226390_at	STARD4	-0.976	-1.97	3.29E-06	4.69E-05
215599_at	GUSBP3 /// GUSBP9 /// LOC100653061 /// LOC101060519	-0.976	-1.97	5.54E-06	7.10E-05
201485_s_at	RCN2	-0.976	-1.97	4.33E-08	1.48E-06
242899_at	SESN3	-0.977	-1.97	3.98E-07	8.57E-06
211002_s_at	TRIM29	-0.978	-1.97	1.33E-05	1.43E-04
222784_at	SMOC1	-0.979	-1.97	1.19E-05	1.31E-04

205255_x_at	TCF7	-0.98	-1.97	5.84E-12	9.43E-10
220527_at	MRPL20	-0.981	-1.97	5.56E-09	2.85E-07
202693_s_at	STK17A	-0.982	-1.98	2.25E-09	1.35E-07
205334_at	S100A1	-0.982	-1.98	3.16E-05	2.87E-04
211466_at	NFIB	-0.983	-1.98	1.84E-06	2.94E-05
238789_at	KANK1	-0.984	-1.98	8.58E-07	1.58E-05
1570511_at	ARHGEF10L	-0.984	-1.98	6.07E-08	1.97E-06
203367_at	DUSP14	-0.985	-1.98	8.22E-15	3.66E-12
203243_s_at	PDLIM5	-0.985	-1.98	1.25E-04	8.74E-04
231230_at	KCNK10	-0.986	-1.98	2.52E-09	1.49E-07
203917_at	CXADR	-0.989	-1.98	4.09E-05	3.55E-04
218574_s_at	LMCD1	-0.99	-1.99	4.02E-08	1.40E-06
228636_at	BHLHE22	-0.99	-1.99	1.89E-05	1.91E-04
201148_s_at	TIMP3	-0.994	-1.99	3.13E-08	1.16E-06
235388_at	CHD9	-0.995	-1.99	2.66E-05	2.52E-04
223278_at	GJB2	-0.995	-1.99	1.13E-12	2.35E-10
210948_s_at	LEF1	-0.996	-1.99	3.70E-10	3.10E-08
210943_s_at	LYST	-0.999	-2	1.26E-05	1.37E-04
1555226_s_at	C1orf43	-1	-2	2.85E-06	4.19E-05
229097_at	DIAPH3	-1	-2	1.15E-05	1.27E-04
238706_at	PAPD4	-1	-2	6.61E-05	5.17E-04
210978_s_at	TAGLN2	-1	-2	4.77E-05	4.00E-04
222354_at	F11R	-1	-2	1.44E-06	2.41E-05
1553915_at	C10orf126	-1	-2	3.64E-09	2.01E-07
235591_at	SSTR1	-1	-2	4.98E-06	6.52E-05
217893_s_at	AKIRIN1	-1	-2.01	6.30E-06	7.84E-05
230254_at	FAM26E	-1	-2.01	7.52E-04	3.59E-03

1554334_a_at	DNAJA4	-1	-2.01	4.44E-12	7.46E-10
201846_s_at	RYBP	-1	-2.01	1.85E-06	2.95E-05
231858_x_at	AP5B1	-1.01	-2.01	2.96E-04	1.72E-03
231875_at	KIF21A	-1.01	-2.01	8.74E-10	6.20E-08
230498_at	MCHR1	-1.01	-2.01	2.48E-06	3.75E-05
223925_s_at	MTPN	-1.01	-2.02	1.54E-05	1.62E-04
229900_at	CD109	-1.01	-2.02	3.63E-11	4.34E-09
1558173_a_at	LUZP1	-1.01	-2.02	1.32E-08	5.61E-07
210172_at	SF1	-1.01	-2.02	1.46E-03	6.10E-03
1557944_s_at	CTNND1 /// TMX2-CTNND1	-1.01	-2.02	4.74E-06	6.28E-05
215374_at	PAPOLA	-1.01	-2.02	4.81E-05	4.03E-04
1557910_at	HSP90AB1	-1.01	-2.02	2.44E-04	1.48E-03
226623_at	PHYHIPL	-1.02	-2.02	8.05E-09	3.83E-07
210393_at	LGR5	-1.02	-2.03	1.81E-09	1.14E-07
34478_at	RAB11B	-1.02	-2.03	1.11E-04	7.91E-04
215028_at	SEMA6A	-1.02	-2.03	5.37E-07	1.07E-05
224218_s_at	TRPS1	-1.02	-2.03	7.04E-07	1.35E-05
216641_s_at	LAD1	-1.02	-2.03	1.39E-07	3.80E-06
200835_s_at	MAP4	-1.02	-2.03	2.18E-05	2.14E-04
215268_at	KIAA0754	-1.02	-2.03	7.95E-10	5.74E-08
233971_at	FAM166A	-1.03	-2.04	1.10E-06	1.93E-05
225105_at	C12orf75	-1.03	-2.04	5.16E-09	2.68E-07
202436_s_at	CYP1B1	-1.03	-2.04	7.35E-06	8.88E-05
242141_at	HDAC2	-1.03	-2.04	2.24E-10	2.01E-08
235057_at	ITCH	-1.03	-2.04	7.51E-05	5.75E-04
226599_at	FHDC1	-1.03	-2.04	1.53E-16	1.45E-13
222162_s_at	ADAMTS1	-1.03	-2.04	1.47E-10	1.40E-08
226065_at	PRICKLE1	-1.03	-2.05	4.50E-10	3.61E-08
228720_at	SORCS2	-1.03	-2.05	3.54E-07	7.85E-06
219225_at	PGBD5	-1.03	-2.05	8.04E-09	3.82E-07
217234_s_at	EZR	-1.04	-2.05	3.02E-05	2.77E-04
1553493_a_at	TDH	-1.04	-2.06	4.89E-07	1.00E-05
226952_at	EAF1	-1.04	-2.06	4.31E-16	3.54E-13

1555299_s_at	ERVW-1	-1.04	-2.06	5.76E-09	2.93E-07
222651_s_at	TRPS1	-1.05	-2.07	2.49E-07	5.98E-06
228303_at	GALNT6	-1.05	-2.07	2.32E-09	1.39E-07
227499_at	FZD3	-1.05	-2.07	4.10E-11	4.80E-09
236241_at	MED31	-1.05	-2.07	3.63E-04	2.02E-03
240024_at	SEC14L2	-1.06	-2.08	4.70E-06	6.24E-05
214698_at	PTBP3	-1.06	-2.08	9.45E-08	2.76E-06
1558732_at	MAP4K4	-1.06	-2.08	8.74E-06	1.02E-04
203184_at	FBN2	-1.06	-2.08	8.50E-05	6.37E-04
1559433_at	APCDD1L-AS1	-1.06	-2.08	5.63E-08	1.86E-06
1553288_a_at	NYAP1	-1.06	-2.08	1.16E-09	7.81E-08
232149_s_at	NSMAF	-1.06	-2.09	2.84E-08	1.07E-06
204378_at	BCAS1	-1.06	-2.09	6.17E-09	3.08E-07
223690_at	LTBP2	-1.06	-2.09	1.00E-09	6.90E-08
232069_at	KIF26A	-1.06	-2.09	1.56E-09	9.91E-08
204736_s_at	CSPG4	-1.07	-2.09	1.00E-07	2.89E-06
216504_s_at	SLC39A8	-1.07	-2.09	3.91E-05	3.41E-04
226637_at	UBE2H	-1.07	-2.1	1.35E-11	1.91E-09
228900_at	SPECC1	-1.07	-2.1	1.86E-08	7.49E-07
207008_at	CXCR2	-1.07	-2.1	6.68E-08	2.13E-06
209242_at	PEG3	-1.07	-2.1	1.31E-06	2.24E-05
217230_at	EZR	-1.07	-2.1	1.98E-05	1.98E-04
205592_at	SLC4A1	-1.07	-2.1	3.97E-09	2.16E-07
220710_at	ANP32A-IT1	-1.07	-2.1	1.67E-07	4.37E-06
236179_at	CDH11	-1.07	-2.11	1.20E-04	8.41E-04
205125_at	PLCD1	-1.08	-2.11	2.25E-12	4.16E-10
227524_at	FZD3	-1.08	-2.11	1.16E-10	1.15E-08
204268_at	S100A2	-1.08	-2.11	1.05E-08	4.67E-07
1564274_at	C9orf47	-1.08	-2.12	2.53E-08	9.72E-07
200644_at	MARCKSL1	-1.08	-2.12	1.04E-07	2.98E-06
216591_s_at	SDHC	-1.08	-2.12	2.60E-04	1.55E-03
206774_at	FRMPD1	-1.08	-2.12	2.03E-09	1.24E-07
203499_at	EPHA2	-1.08	-2.12	2.21E-13	5.66E-11
216222_s_at	MYO10	-1.08	-2.12	1.12E-06	1.96E-05
201616_s_at	CALD1	-1.09	-2.12	1.00E-09	6.90E-08

235022_at	FAM210A	-1.09	-2.13	1.02E-11	1.50E-09
211367_s_at	CASP1	-1.09	-2.13	3.01E-10	2.60E-08
203423_at	RBP1	-1.09	-2.13	1.26E-05	1.37E-04
208613_s_at	FLNB	-1.09	-2.13	1.52E-08	6.30E-07
226829_at	AFAP1L2	-1.09	-2.13	5.52E-17	6.80E-14
1552511_a_at	CPA6	-1.09	-2.13	9.05E-12	1.37E-09
219612_s_at	FGG	-1.1	-2.14	4.87E-06	6.41E-05
207649_at	KRT37	-1.1	-2.14	1.33E-02	3.61E-02
214104_at	GPR161	-1.1	-2.14	2.19E-10	1.97E-08
1556821_x_at	DLEU2	-1.1	-2.14	1.58E-05	1.65E-04
204132_s_at	FOXO3 /// FOXO3B	-1.1	-2.14	1.66E-06	2.69E-05
227069_at	CUX1	-1.1	-2.14	4.15E-09	2.24E-07
232704_s_at	LRRFIP2	-1.1	-2.14	9.48E-05	6.94E-04
1552626_a_at	TMEM163	-1.1	-2.15	5.74E-05	4.62E-04
206044_s_at	BRAF	-1.1	-2.15	2.13E-09	1.29E-07
1552685_a_at	GRHL1	-1.1	-2.15	8.98E-10	6.32E-08
230204_at	HAPLN1	-1.11	-2.15	2.13E-03	8.28E-03
1552487_a_at	BNC1	-1.11	-2.16	1.24E-09	8.22E-08
203797_at	VSNL1	-1.11	-2.16	2.15E-11	2.84E-09
242163_at	THRAP3	-1.11	-2.16	7.22E-06	8.74E-05
214939_x_at	MLLT4	-1.12	-2.17	3.82E-18	8.82E-15
200052_s_at	ILF2	-1.12	-2.17	5.51E-07	1.10E-05
219382_at	SERTAD3	-1.12	-2.17	1.99E-09	1.23E-07
223078_s_at	TMOD3	-1.12	-2.17	4.13E-12	7.07E-10
242321_at	PTPN14	-1.12	-2.17	1.30E-03	5.57E-03
208539_x_at	SPRR2B	-1.12	-2.17	5.70E-04	2.88E-03
222774_s_at	NETO2	-1.12	-2.18	7.14E-09	3.47E-07
201559_s_at	CLIC4	-1.12	-2.18	1.45E-10	1.39E-08
210413_x_at	SERPINB3 /// SERPINB4	-1.12	-2.18	1.51E-02	4.01E-02
1554333_at	DNAJA4	-1.12	-2.18	1.07E-10	1.09E-08
221102_s_at	TRPM6	-1.13	-2.18	3.16E-10	2.71E-08
204181_s_at	ZBTB43	-1.13	-2.18	3.83E-05	3.35E-04
239719_at	CD109	-1.13	-2.19	5.87E-11	6.42E-09

236140_at	GCLM	-1.13	-2.19	2.18E-05	2.15E-04
205941_s_at	COL10A1	-1.13	-2.19	5.49E-09	2.83E-07
217924_at	C6orf106	-1.14	-2.2	5.19E-08	1.73E-06
208055_s_at	HERC4	-1.14	-2.2	1.98E-07	5.00E-06
1555247_a_at	FNIP1 /// RAPGEF6	-1.14	-2.2	8.10E-06	9.63E-05
221389_at	PLA2G2E	-1.14	-2.2	3.60E-14	1.22E-11
229065_at	SLC35F3	-1.14	-2.21	6.83E-07	1.32E-05
203691_at	PI3	-1.14	-2.21	8.48E-03	2.52E-02
201445_at	CNN3	-1.14	-2.21	3.31E-08	1.20E-06
202147_s_at	IFRD1	-1.15	-2.21	3.21E-12	5.74E-10
1566465_at	KCNK1	-1.15	-2.22	2.09E-06	3.25E-05
213456_at	SOSTDC1	-1.15	-2.22	1.15E-10	1.15E-08
211538_s_at	HSPA2	-1.15	-2.22	2.22E-15	1.35E-12
226968_at	KIF1B	-1.15	-2.23	2.83E-07	6.63E-06
224941_at	PAPPA	-1.15	-2.23	1.27E-07	3.53E-06
1555462_at	PPP1R1C	-1.16	-2.23	1.66E-05	1.72E-04
213759_at	ARL4C	-1.16	-2.23	2.18E-12	4.07E-10
213358_at	SOGA2	-1.16	-2.23	3.35E-09	1.87E-07
219768_at	VTCN1	-1.16	-2.24	2.97E-04	1.73E-03
208637_x_at	ACTN1	-1.17	-2.24	6.03E-08	1.96E-06
37892_at	COL11A1	-1.17	-2.24	8.48E-03	2.51E-02
209343_at	EFHD1	-1.17	-2.24	2.86E-04	1.68E-03
211071_s_at	MLLT11	-1.17	-2.25	3.32E-13	8.07E-11
221795_at	NTRK2	-1.17	-2.25	1.90E-06	3.01E-05
205899_at	CCNA1	-1.17	-2.25	1.06E-05	1.20E-04
224595_at	SLC44A1	-1.17	-2.25	3.10E-09	1.76E-07
219956_at	GALNT6	-1.17	-2.25	1.15E-09	7.75E-08
241412_at	BTC	-1.17	-2.26	1.92E-06	3.03E-05
226767_s_at	FAHD1	-1.17	-2.26	2.48E-07	5.96E-06
202206_at	ARL4C	-1.17	-2.26	2.70E-10	2.36E-08
206448_at	ZNF365	-1.18	-2.26	8.29E-06	9.82E-05
206758_at	EDN2	-1.18	-2.26	3.37E-11	4.11E-09
203222_s_at	TLE1	-1.18	-2.26	1.58E-09	9.97E-08
1552536_at	VTI1A	-1.18	-2.26	5.87E-05	4.70E-04

213385_at	CHN2	-1.18	-2.26	9.91E-08	2.87E-06
201341_at	ENC1	-1.18	-2.27	2.48E-07	5.97E-06
236700_at	EIF3C	-1.18	-2.27	4.73E-05	3.97E-04
222783_s_at	SMOC1	-1.18	-2.27	1.42E-07	3.86E-06
212807_s_at	SORT1	-1.18	-2.27	1.63E-10	1.53E-08
224520_s_at	BEST3	-1.19	-2.27	1.26E-06	2.16E-05
220518_at	ABI3BP	-1.19	-2.28	4.07E-08	1.41E-06
226535_at	ITGB6	-1.19	-2.28	1.99E-10	1.82E-08
229856_s_at	PITHD1	-1.19	-2.28	3.40E-11	4.13E-09
1557122_s_at	GABRB2	-1.19	-2.28	3.24E-06	4.63E-05
209772_s_at	CD24	-1.19	-2.28	1.56E-08	6.48E-07
210335_at	RASSF9	-1.19	-2.28	3.74E-08	1.33E-06
1553710_at	FAM218A	-1.19	-2.29	3.23E-07	7.31E-06
204790_at	SMAD7	-1.2	-2.29	3.57E-11	4.28E-09
1556186_s_at	EMC1	-1.2	-2.3	1.83E-05	1.86E-04
205523_at	HAPLN1	-1.2	-2.3	3.13E-04	1.80E-03
201147_s_at	TIMP3	-1.2	-2.3	1.31E-07	3.61E-06
223427_s_at	EPB41L4B	-1.2	-2.3	3.66E-08	1.30E-06
223879_s_at	OXR1	-1.21	-2.31	9.18E-10	6.42E-08
211764_s_at	UBE2D1	-1.21	-2.32	3.05E-15	1.76E-12
238460_at	FAM83A	-1.21	-2.32	1.54E-12	3.05E-10
202883_s_at	PPP2R1B	-1.22	-2.32	6.88E-15	3.27E-12
224277_at	MOP-1	-1.22	-2.33	8.51E-12	1.31E-09
233555_s_at	SULF2	-1.22	-2.33	1.50E-08	6.26E-07
41469_at	PI3	-1.22	-2.33	7.25E-03	2.22E-02
203827_at	WIPI1	-1.22	-2.33	1.12E-12	2.35E-10
208216_at	DLX4	-1.22	-2.33	3.19E-07	7.26E-06
241193_at	ETS2	-1.22	-2.33	2.22E-04	1.37E-03
201615_x_at	CALD1	-1.22	-2.34	1.74E-07	4.50E-06
211361_s_at	SERPINB13	-1.23	-2.34	3.32E-14	1.16E-11
207195_at	CNTN6	-1.23	-2.34	1.33E-05	1.43E-04
206409_at	TIAM1	-1.23	-2.35	8.52E-07	1.57E-05
202016_at	MEST	-1.23	-2.35	1.89E-07	4.82E-06
200744_s_at	GNB1	-1.23	-2.35	3.86E-15	2.10E-12

239155_at	CXADR	-1.23	-2.35	4.13E-09	2.23E-07
220999_s_at	CYFIP2	-1.23	-2.35	3.53E-08	1.27E-06
209098_s_at	JAG1	-1.23	-2.35	4.84E-12	8.05E-10
205535_s_at	PCDH7	-1.23	-2.35	8.80E-08	2.62E-06
228910_at	CD82	-1.24	-2.36	4.87E-12	8.08E-10
223541_at	HAS3	-1.24	-2.37	5.32E-10	4.13E-08
229163_at	CAMK2N1	-1.24	-2.37	7.19E-05	5.55E-04
231395_at	ATP8A2	-1.24	-2.37	2.56E-10	2.26E-08
209442_x_at	ANK3	-1.24	-2.37	4.43E-07	9.27E-06
223650_s_at	NRBF2	-1.24	-2.37	1.22E-12	2.48E-10
210387_at	NCALD	-1.24	-2.37	4.73E-07	9.77E-06
217933_s_at	LAP3	-1.25	-2.37	4.04E-12	6.95E-10
205637_s_at	SH3GL3	-1.25	-2.37	2.06E-08	8.19E-07
230138_at	NCS1	-1.25	-2.37	3.96E-12	6.88E-10
223503_at	TMEM163	-1.25	-2.38	2.86E-05	2.66E-04
215150_at	YOD1	-1.25	-2.38	3.75E-11	4.44E-09
235075_at	DSG3	-1.25	-2.39	4.89E-14	1.56E-11
221114_at	AMBN	-1.26	-2.39	3.88E-08	1.36E-06
222853_at	FLRT3	-1.26	-2.39	9.76E-10	6.74E-08
242344_at	GABRB2	-1.26	-2.4	5.12E-06	6.69E-05
228915_at	DACH1	-1.27	-2.41	3.13E-06	4.50E-05
211219_s_at	LHX2	-1.27	-2.41	3.30E-07	7.42E-06
239586_at	FAM83A	-1.29	-2.44	2.04E-10	1.85E-08
216258_s_at	SERPINB13	-1.29	-2.45	1.16E-09	7.81E-08
216604_s_at	SLC7A8	-1.29	-2.45	4.84E-09	2.54E-07
221425_s_at	ISCA1	-1.29	-2.45	5.28E-14	1.67E-11
205709_s_at	CDS1	-1.3	-2.46	2.24E-11	2.91E-09
1556421_at	LOC286189	-1.3	-2.46	2.45E-07	5.91E-06
204733_at	KLK6	-1.3	-2.46	3.24E-06	4.64E-05
201043_s_at	ANP32A	-1.3	-2.46	1.10E-09	7.50E-08
212531_at	LCN2	-1.3	-2.47	6.36E-05	5.02E-04
221558_s_at	LEF1	-1.31	-2.47	6.65E-11	7.14E-09
241854_at	DNASE1	-1.31	-2.47	2.07E-09	1.26E-07
212822_at	HEG1	-1.31	-	3.85E-08	1.35E-06

			2.48		
235479_at	CPEB2	-1.31	-2.48	8.92E-11	9.40E-09
200906_s_at	PALLD	-1.31	-2.48	1.26E-10	1.23E-08
212942_s_at	KIAA1199	-1.32	-2.49	5.52E-15	2.93E-12
238593_at	C11orf80	-1.32	-2.49	1.07E-05	1.21E-04
230469_at	RTKN2	-1.32	-2.5	1.22E-05	1.34E-04
203798_s_at	VSNL1	-1.32	-2.5	2.66E-10	2.33E-08
222015_at	CSNK1E	-1.32	-2.5	4.57E-12	7.65E-10
205595_at	DSG3	-1.33	-2.51	2.41E-09	1.43E-07
206089_at	NELL1	-1.33	-2.51	1.82E-04	1.18E-03
228922_at	SHF	-1.34	-2.53	3.44E-11	4.17E-09
204751_x_at	DSC2	-1.34	-2.53	1.65E-11	2.26E-09
214417_s_at	FETUB	-1.34	-2.53	3.34E-09	1.87E-07
227410_at	FAM43A	-1.34	-2.54	3.35E-14	1.16E-11
241985_at	JMY	-1.34	-2.54	4.72E-11	5.37E-09
228605_at	UBXN2A	-1.35	-2.54	9.44E-10	6.57E-08
205525_at	CALD1	-1.35	-2.55	3.07E-11	3.78E-09
215271_at	TNN	-1.35	-2.55	1.57E-04	1.04E-03
239028_at	LYPD6	-1.35	-2.55	2.41E-11	3.11E-09
220800_s_at	TMOD3	-1.36	-2.57	6.36E-12	1.01E-09
209875_s_at	SPP1	-1.36	-2.57	3.04E-05	2.79E-04
1552807_a_at	SIGLEC10	-1.36	-2.57	1.03E-08	4.60E-07
232378_at	SLC5A9	-1.36	-2.57	2.02E-07	5.10E-06
203650_at	PROCR	-1.37	-2.58	6.49E-14	1.97E-11
233340_at	SPINK13	-1.37	-2.59	5.86E-08	1.92E-06
217395_at	MT4	-1.37	-2.59	2.85E-11	3.59E-09
229228_at	CREB5 /// LOC401317	-1.38	-2.6	8.23E-09	3.88E-07
206504_at	CYP24A1	-1.38	-2.6	2.17E-07	5.41E-06
219842_at	ARL15	-1.39	-2.61	5.16E-12	8.47E-10
206561_s_at	AKR1B10	-1.39	-2.61	3.62E-05	3.20E-04
204705_x_at	ALDOB	-1.39	-2.62	5.24E-10	4.09E-08
229271_x_at	COL11A1	-1.39	-2.62	8.70E-04	4.03E-03
214636_at	CALCB	-1.4	-2.63	3.19E-10	2.73E-08
226681_at	UBE2H	-1.4	-2.63	7.84E-14	2.28E-11
223679_at	CTNNB1	-1.41	-2.65	2.51E-10	2.22E-08

230276_at	FAM49A	-1.41	-2.65	2.23E-08	8.79E-07
231743_at	WNT3	-1.41	-2.66	6.56E-17	7.57E-14
212478_at	RMND5A	-1.41	-2.66	1.01E-14	4.39E-12
208708_x_at	EIF5	-1.42	-2.67	7.54E-18	1.27E-14
204351_at	S100P	-1.42	-2.67	2.47E-07	5.95E-06
228068_at	GOLGA7B	-1.42	-2.68	3.26E-13	8.02E-11
214226_at	PRSS53	-1.42	-2.68	1.19E-12	2.43E-10
203354_s_at	PSD3	-1.42	-2.68	2.46E-05	2.36E-04
200730_s_at	PTP4A1	-1.42	-2.68	5.24E-10	4.09E-08
239203_at	LSMEM1	-1.43	-2.69	3.78E-09	2.08E-07
226698_at	FCHSD1	-1.43	-2.7	1.78E-11	2.40E-09
224724_at	SULF2	-1.43	-2.7	6.14E-13	1.42E-10
1558687_a_at	FOXN1	-1.44	-2.71	1.37E-13	3.80E-11
236255_at	PLEKHG4B	-1.44	-2.72	7.06E-11	7.56E-09
218384_at	CARHSP1	-1.45	-2.72	4.22E-11	4.92E-09
212915_at	PDZRN3	-1.45	-2.74	9.32E-12	1.39E-09
227650_at	HSPA14	-1.46	-2.74	5.87E-09	2.96E-07
205676_at	CYP27B1	-1.46	-2.75	7.69E-07	1.45E-05
244533_at	PTPN14	-1.46	-2.75	3.07E-07	7.07E-06
220129_at	CCDC169-SOHLH2 /// SOHLH2	-1.46	-2.75	3.33E-08	1.21E-06
222871_at	KLHDC8A	-1.46	-2.76	7.20E-10	5.30E-08
235683_at	SESN3	-1.46	-2.76	3.05E-10	2.62E-08
203130_s_at	KIF5C	-1.47	-2.77	4.10E-06	5.61E-05
215779_s_at	HIST1H2BC /// HIST1H2BE /// HIST1H2BF /// HIST1H2BG /// HIST1H2BI /// NCALD	-1.47	-2.77	5.95E-08	1.94E-06
242138_at	DLX1	-1.47	-2.78	5.38E-07	1.08E-05
220038_at	C8orf44-SGK3 /// SGK3	-1.48	-2.78	4.84E-08	1.63E-06
1556533_at	LINC00868	-1.48	-2.79	8.07E-15	3.64E-12
210521_s_at	FETUB	-1.48	-2.79	1.12E-10	1.12E-08
220984_s_at	SLCO5A1	-1.49	-2.8	9.50E-09	4.34E-07
227955_s_at	EFNA5	-1.49	-2.81	1.46E-14	6.07E-12
229942_at	BNC2	-1.5	-2.83	2.23E-10	2.01E-08
236651_at	KALRN	-1.5	-2.83	5.99E-06	7.56E-05
228996_at	RC3H1	-1.51	-2.84	1.12E-12	2.35E-10
232361_s_at	EHF	-1.51	-2.85	3.39E-06	4.81E-05

1569852_at	LSMEM1	-1.51	-2.86	7.62E-11	8.14E-09
1554097_a_at	MIR31HG	-1.52	-2.87	2.67E-14	9.87E-12
239726_at	ANK3	-1.52	-2.87	3.06E-08	1.14E-06
213457_at	MFHAS1	-1.53	-2.9	1.86E-14	7.50E-12
217799_x_at	UBE2H	-1.54	-2.9	6.72E-13	1.54E-10
207291_at	PRRG4	-1.54	-2.91	1.08E-10	1.09E-08
217428_s_at	COL10A1	-1.54	-2.91	4.65E-07	9.65E-06
219463_at	LAMP5	-1.54	-2.92	5.93E-10	4.56E-08
238568_s_at	NPC1	-1.55	-2.92	1.74E-12	3.38E-10
236263_at	SHH	-1.55	-2.93	1.14E-08	5.03E-07
206140_at	LHX2	-1.55	-2.93	5.78E-05	4.64E-04
230740_at	EHD3	-1.55	-2.93	7.43E-10	5.44E-08
209126_x_at	KRT6B	-1.55	-2.93	1.47E-08	6.16E-07
1553539_at	KRT74	-1.55	-2.93	2.60E-11	3.30E-09
227950_at	UBE2H	-1.56	-2.94	1.44E-14	6.05E-12
243541_at	IL31RA	-1.56	-2.95	3.16E-05	2.87E-04
207367_at	ATP12A	-1.56	-2.95	1.36E-05	1.46E-04
202435_s_at	CYP1B1	-1.56	-2.95	4.25E-06	5.77E-05
228653_at	SAMD5	-1.56	-2.96	3.62E-08	1.29E-06
201149_s_at	TIMP3	-1.58	-2.98	1.63E-08	6.72E-07
237981_at	CMYA5	-1.58	-2.98	1.10E-12	2.33E-10
201110_s_at	THBS1	-1.58	-2.99	1.63E-09	1.03E-07
210689_at	CLDN14	-1.58	-2.99	1.10E-10	1.10E-08
220782_x_at	KLK12	-1.58	-2.99	2.93E-08	1.10E-06
239082_at	FZD3	-1.59	-3	2.55E-09	1.51E-07
232170_at	S100A7A	-1.59	-3.01	1.26E-02	3.46E-02
1553494_at	TDH	-1.59	-3.01	8.61E-07	1.58E-05
214590_s_at	UBE2D1	-1.6	-3.02	6.90E-15	3.27E-12
209372_x_at	TUBB2A /// TUBB2B	-1.6	-3.02	3.57E-15	1.97E-12
230135_at	HHIP	-1.6	-3.04	1.23E-09	8.22E-08
203413_at	NELL2	-1.6	-3.04	1.55E-07	4.12E-06
226122_at	PLEKHG1	-1.61	-3.05	9.48E-16	6.26E-13
238741_at	FAM83A	-1.61	-3.06	1.43E-13	3.91E-11
242329_at	CREB5 /// LOC401317	-1.63	-3.09	6.83E-12	1.08E-09

203535_at	S100A9	-1.64	-3.11	4.22E-04	2.28E-03
200790_at	ODC1	-1.64	-3.11	2.17E-13	5.63E-11
215034_s_at	TM4SF1	-1.64	-3.11	6.71E-10	5.09E-08
221416_at	PLA2G2F	-1.64	-3.11	1.92E-15	1.23E-12
243478_at	CHST2	-1.64	-3.12	4.76E-10	3.79E-08
234488_s_at	GMCL1 /// GMCL1P1	-1.65	-3.14	1.04E-12	2.22E-10
231778_at	DLX3	-1.65	-3.15	1.12E-14	4.82E-12
225295_at	SLC39A10	-1.66	-3.16	5.64E-14	1.77E-11
231867_at	TENM2	-1.66	-3.16	1.72E-10	1.61E-08
220272_at	BNC2	-1.67	-3.18	8.62E-10	6.14E-08
209387_s_at	TM4SF1	-1.67	-3.19	1.09E-10	1.10E-08
211906_s_at	SERPINB4	-1.68	-3.19	4.36E-03	1.48E-02
219250_s_at	FLRT3	-1.68	-3.2	3.36E-14	1.16E-11
205471_s_at	DACH1	-1.69	-3.22	1.42E-09	9.18E-08
226887_at	HSPA14	-1.69	-3.22	3.97E-17	5.06E-14
227961_at	CTSB	-1.69	-3.22	5.80E-09	2.93E-07
234316_x_at	KLK12	-1.7	-3.24	1.68E-10	1.58E-08
229084_at	CNTN4	-1.7	-3.24	3.26E-07	7.35E-06
209683_at	FAM49A	-1.71	-3.26	1.33E-10	1.30E-08
206882_at	SLC1A6	-1.71	-3.27	2.09E-12	3.99E-10
218458_at	GMCL1	-1.72	-3.29	1.39E-17	1.91E-14
237003_at	BEST3	-1.73	-3.32	6.18E-09	3.08E-07
206604_at	OVOL1	-1.76	-3.38	5.97E-13	1.39E-10
215785_s_at	CYFIP2	-1.76	-3.39	9.17E-15	4.03E-12
240389_at	TRPM6	-1.77	-3.4	2.20E-09	1.32E-07
219683_at	FZD3	-1.77	-3.41	4.04E-12	6.95E-10
206935_at	PCDH8	-1.77	-3.41	8.02E-07	1.50E-05
223739_at	PADI1	-1.77	-3.42	6.78E-10	5.11E-08
212479_s_at	RMND5A	-1.78	-3.43	4.89E-18	9.89E-15
206912_at	FOXE1	-1.78	-3.43	1.52E-10	1.44E-08
242062_at	SAMD8	-1.78	-3.43	7.72E-12	1.21E-09
213793_s_at	HOMER1	-1.79	-3.46	7.85E-14	2.28E-11
1553388_at	FAM26D	-1.8	-3.47	5.84E-12	9.43E-10
1569433_at	SAMD5	-1.8	-3.47	2.68E-12	4.81E-10

208290_s_at	EIF5	-1.8	-3.48	5.74E-17	6.84E-14
208250_s_at	DMBT1	-1.8	-3.49	9.70E-08	2.82E-06
220624_s_at	ELF5	-1.82	-3.54	4.08E-14	1.35E-11
219554_at	RHCG	-1.83	-3.54	2.63E-10	2.31E-08
1555774_at	ZAR1	-1.83	-3.55	7.66E-16	5.55E-13
232555_at	CREB5	-1.84	-3.57	1.04E-12	2.22E-10
213774_s_at	PPP1R2	-1.84	-3.59	6.64E-15	3.27E-12
217031_at	KRT84	-1.85	-3.6	6.18E-09	3.08E-07
217678_at	SLC7A11	-1.86	-3.62	5.21E-11	5.84E-09
219301_s_at	CNTNAP2	-1.87	-3.65	1.38E-10	1.33E-08
235723_at	BNC2	-1.87	-3.65	3.65E-10	3.06E-08
1553574_at	IFNE	-1.87	-3.66	4.18E-12	7.12E-10
225809_at	PARM1	-1.88	-3.67	1.71E-12	3.34E-10
233814_at	EFNA5	-1.9	-3.73	7.78E-13	1.74E-10
233586_s_at	KLK12	-1.9	-3.73	1.33E-09	8.77E-08
232682_at	MREG	-1.9	-3.74	6.06E-14	1.87E-11
1553829_at	CYP1B1-AS1	-1.9	-3.74	2.59E-09	1.52E-07
216092_s_at	SLC7A8	-1.91	-3.76	1.59E-14	6.52E-12
1557321_a_at	CAPN14	-1.92	-3.78	1.13E-11	1.65E-09
228975_at	SP6	-1.92	-3.79	5.35E-18	9.89E-15
229030_at	CAPN8	-1.92	-3.79	1.51E-07	4.06E-06
206326_at	GRP	-1.93	-3.81	2.55E-04	1.53E-03
205290_s_at	BMP2	-1.93	-3.81	1.68E-12	3.30E-10
243445_at	BNC2	-1.94	-3.83	1.14E-10	1.14E-08
213880_at	LGR5	-1.94	-3.85	6.21E-08	2.01E-06
233301_at	OXCT2	-1.96	-3.89	7.03E-14	2.09E-11
223720_at	SPINK7	-1.96	-3.9	4.32E-07	9.09E-06
237905_at	KRT25	-1.96	-3.9	4.55E-05	3.85E-04
238625_at	C1orf168	-1.97	-3.91	7.62E-13	1.72E-10
202752_x_at	SLC7A8	-1.97	-3.93	4.52E-13	1.07E-10
202884_s_at	PPP2R1B	-1.97	-3.93	2.17E-13	5.63E-11
214576_at	KRT36	-1.98	-3.94	2.92E-14	1.06E-11
213107_at	TNIK	-2	-4.01	7.86E-12	1.23E-09
1554593_s_at	SLC1A6	-2.03	-4.08	1.11E-09	7.51E-08

1554592_a_at	SLC1A6	-2.04	-4.12	1.41E-12	2.82E-10
202886_s_at	PPP2R1B	-2.08	-4.22	1.83E-12	3.52E-10
1553861_at	TCP11L2	-2.09	-4.25	8.81E-18	1.42E-14
209757_s_at	MYCN	-2.09	-4.27	7.76E-09	3.70E-07
209859_at	TRIM9	-2.1	-4.29	1.53E-10	1.45E-08
222351_at	PPP2R1B	-2.11	-4.31	3.85E-08	1.35E-06
205931_s_at	CREB5 /// LOC401317	-2.11	-4.31	7.85E-13	1.75E-10
218935_at	EHD3	-2.11	-4.32	2.06E-11	2.77E-09
232568_at	MGC24103	-2.18	-4.52	1.91E-14	7.60E-12
209981_at	CSDC2	-2.19	-4.57	8.54E-12	1.31E-09
205555_s_at	MSX2	-2.19	-4.57	3.30E-13	8.07E-11
218266_s_at	NCS1	-2.2	-4.59	9.20E-14	2.63E-11
209921_at	SLC7A11	-2.2	-4.59	9.03E-13	1.97E-10
224412_s_at	TRPM6	-2.2	-4.59	1.78E-11	2.40E-09
210082_at	ABCA4	-2.2	-4.59	2.11E-12	4.00E-10
241014_at	FLG-AS1	-2.21	-4.62	3.10E-11	3.79E-09
210319_x_at	MSX2	-2.21	-4.62	8.22E-13	1.82E-10
205724_at	PKP1	-2.22	-4.65	9.24E-19	2.84E-15
203921_at	CHST2	-2.22	-4.67	1.86E-10	1.72E-08
221962_s_at	UBE2H	-2.23	-4.69	8.47E-17	9.21E-14
224262_at	IL1F10	-2.23	-4.7	1.01E-09	6.94E-08
233123_at	SLC40A1	-2.24	-4.73	6.35E-12	1.01E-09
227506_at	SLC16A9	-2.29	-4.88	9.61E-16	6.26E-13
205289_at	BMP2	-2.29	-4.9	2.18E-13	5.63E-11
202166_s_at	PPP1R2	-2.34	-5.05	6.93E-19	2.33E-15
209618_at	CTNND2	-2.35	-5.09	1.01E-07	2.90E-06
210311_at	FGF5	-2.37	-5.19	5.79E-14	1.80E-11
209617_s_at	CTNND2	-2.38	-5.2	4.66E-10	3.72E-08
209800_at	KRT16	-2.38	-5.2	1.05E-12	2.24E-10
231461_at	KRT71	-2.46	-5.5	2.90E-06	4.25E-05
230722_at	BNC2	-2.46	-5.51	2.69E-13	6.82E-11
213780_at	TCHH	-2.47	-5.54	3.88E-06	5.36E-05
220090_at	CRNN	-2.49	-5.61	9.47E-10	6.58E-08
207147_at	DLX2	-2.49	-5.62	7.57E-10	5.51E-08
228646_at	PPP1R1C	-2.49	-	3.11E-13	7.81E-11

			5.63		
221627_at	TRIM10	-2.5	-5.64	2.13E-12	4.01E-10
213909_at	LRRC15	-2.52	-5.72	1.35E-09	8.85E-08
229823_at	RIMS2	-2.53	-5.78	1.77E-11	2.40E-09
202376_at	SERPINA3	-2.54	-5.83	1.26E-09	8.36E-08
237507_at	KRT73	-2.58	-5.99	1.11E-09	7.53E-08
211485_s_at	FGF18	-2.59	-6.02	5.55E-15	2.93E-12
227763_at	LYPD6	-2.62	-6.16	8.28E-12	1.28E-09
1553537_at	KRT73	-2.63	-6.19	1.30E-12	2.63E-10
211357_s_at	ALDOB	-2.63	-6.2	4.23E-10	3.42E-08
219932_at	SLC27A6	-2.64	-6.24	6.60E-16	4.94E-13
203304_at	BAMBI	-2.65	-6.29	1.80E-10	1.67E-08
205374_at	SLN	-2.67	-6.37	6.21E-14	1.90E-11
230493_at	SHISA2	-2.67	-6.37	3.61E-08	1.29E-06
228705_at	CAPN12	-2.75	-6.75	8.33E-16	5.81E-13
204687_at	PARM1	-2.76	-6.8	1.01E-17	1.49E-14
239572_at	GJA3	-2.78	-6.86	7.25E-14	2.14E-11
229158_at	WNK4	-2.78	-6.88	2.50E-09	1.48E-07
1564435_a_at	KRT72	-2.79	-6.94	4.68E-11	5.33E-09
238478_at	BNC2	-2.81	-7	3.45E-14	1.18E-11
219832_s_at	HOXC13	-2.91	-7.53	3.51E-18	8.64E-15
204720_s_at	DNAJC6	-2.92	-7.55	3.71E-16	3.12E-13
211429_s_at	SERPINA1	-2.96	-7.76	2.15E-11	2.84E-09
207065_at	KRT75	-3.02	-8.1	1.38E-11	1.94E-09
229730_at	SMTNL2	-3.05	-8.28	2.20E-11	2.89E-09
1562628_at	KRT40	-3.05	-8.31	7.93E-12	1.24E-09
237732_at	PRR9	-3.09	-8.5	2.14E-07	5.34E-06
1555775_a_at	ZAR1	-3.14	-8.79	5.69E-15	2.95E-12
219659_at	ATP8A2	-3.14	-8.81	2.23E-12	4.15E-10
231583_at	KRT74	-3.23	-9.35	7.52E-08	2.33E-06
202833_s_at	SERPINA1	-3.24	-9.42	2.24E-11	2.91E-09
220256_s_at	OXCT2	-3.29	-9.77	5.18E-18	9.89E-15
240388_at	KRT27	-3.31	-9.92	7.10E-08	2.24E-06
206994_at	CST4	-3.44	-10.9	7.88E-15	3.59E-12
227764_at	LYPD6	-3.48	-11.1	5.97E-15	3.02E-12

206987_x_at	FGF18	-3.48	-11.2	4.82E-16	3.79E-13
211029_x_at	FGF18	-3.49	-11.3	2.93E-15	1.72E-12
208092_s_at	FAM49A	-3.57	-11.8	6.86E-15	3.27E-12
220635_at	PSORS1C2	-3.63	-12.4	1.08E-18	3.06E-15
205713_s_at	COMP	-3.94	-15.4	1.94E-13	5.15E-11
230720_at	RNF182	-3.97	-15.6	2.02E-15	1.26E-12
214284_s_at	FGF18	-4.05	-16.6	6.75E-14	2.03E-11
214517_at	KRTAP5-9	-4.06	-16.7	6.44E-15	3.22E-12
232887_at	PIRT	-4.07	-16.8	1.37E-16	1.41E-13
1554398_at	LYG2	-4.16	-17.8	1.80E-17	2.38E-14
206027_at	S100A3	-4.19	-18.2	6.02E-18	1.06E-14
231382_at	FGF18	-4.23	-18.8	9.23E-16	6.26E-13
1564921_at	KRTAP13-1	-4.37	-20.7	1.63E-11	2.24E-09
207716_at	KRT38	-4.43	-21.6	4.36E-14	1.40E-11
206423_at	ANGPTL7	-4.46	-21.9	2.30E-12	4.23E-10
220779_at	PADI3	-4.56	-23.6	1.59E-12	3.15E-10
1562629_a_at	KRT40	-4.78	-27.5	1.46E-13	3.97E-11
242301_at	CBLN2	-4.86	-29.1	5.64E-10	4.36E-08
237853_x_at	KRTAP10-12	-5.04	-32.9	7.30E-20	3.86E-16
206224_at	CST1	-5.06	-33.4	4.31E-18	9.37E-15
207146_at	KRT32	-5.18	-36.3	7.24E-15	3.36E-12
206677_at	KRT31	-5.25	-38	4.41E-16	3.54E-13
215189_at	KRT86 /// LOC100509764	-5.35	-40.7	1.55E-13	4.18E-11
207670_at	KRT85	-5.65	-50.1	9.22E-13	2.00E-10
233158_at	KRT82	-5.78	-54.9	3.38E-16	2.98E-13
240967_at	KRTAP19-3	-6.07	-67.2	9.65E-16	6.26E-13
216921_s_at	KRT35	-6.16	-71.5	3.08E-11	3.78E-09
234680_at	KRTAP17-1	-6.25	-76.3	1.87E-14	7.50E-12
219270_at	CHAC1	-6.27	-77.4	5.72E-20	3.61E-16
234685_x_at	KRTAP4-9	-6.44	-86.5	7.27E-13	1.65E-10
233681_at	KRTAP3-3	-6.5	-90.5	4.35E-12	7.37E-10
233631_x_at	KRTAP9-2 /// KRTAP9-8 /// LOC100996762	-6.57	-95	6.75E-13	1.54E-10
208532_x_at	KRTAP5-8	-6.57	-95.2	5.86E-20	3.61E-16
1560897_a_at	KRTAP10-11	-6.75	-108	7.35E-17	8.23E-14

234633_at	KRTAP4-11	-6.76	-108	9.71E-17	1.03E-13
1561330_at	DSG4	-6.76	-109	9.23E-18	1.42E-14
1556410_a_at	KRTAP19-1	-6.76	-109	5.75E-15	2.95E-12
213711_at	KRT81	-6.81	-112	3.38E-18	8.64E-15
1564803_at	KRTAP11-1	-6.84	-114	2.92E-14	1.06E-11
206969_at	KRT34 /// LOC100653049	-6.95	-124	2.59E-14	9.66E-12
207669_at	KRT83	-7	-128	1.34E-20	3.61E-16
234631_at	KRTAP4-8	-7.02	-130	2.55E-16	2.33E-13
234772_s_at	KRTAP2-1 /// KRTAP2-2	-7.03	-130	2.06E-13	5.43E-11
233534_at	KRTAP3-2	-7.05	-132	1.76E-13	4.71E-11
216810_at	KRTAP4-7	-7.07	-135	9.35E-14	2.66E-11
233537_at	KRTAP3-1	-7.08	-136	2.31E-13	5.89E-11
207457_s_at	LY6G6D	-7.2	-147	3.65E-20	3.61E-16
220978_at	KRTAP1-3	-7.28	-155	1.39E-13	3.82E-11
220970_s_at	KRTAP2-3 /// KRTAP2-4	-7.28	-156	3.08E-14	1.10E-11
234678_at	KRTAP4-3	-7.31	-159	7.92E-16	5.63E-13
233533_at	KRTAP1-5	-7.33	-161	9.69E-14	2.73E-11
1564960_at	KRTAP7-1	-7.35	-164	3.60E-19	1.33E-15
234635_at	KRTAP4-1	-7.42	-172	4.30E-14	1.39E-11
220976_s_at	KRTAP1-1	-7.44	-173	4.09E-14	1.35E-11
234639_x_at	KRTAP9-8	-7.52	-184	2.13E-15	1.31E-12
234679_at	KRTAP9-3	-7.58	-192	6.68E-16	4.94E-13
224269_at	KRTAP4-12	-7.59	-192	2.95E-14	1.06E-11
208483_x_at	KRT33A	-7.6	-194	3.00E-19	1.23E-15
207787_at	KRT33B	-7.61	-195	7.28E-15	3.36E-12
233640_x_at	KRTAP9-4	-7.62	-197	3.47E-15	1.97E-12
234691_at	KRTAP2-1	-7.62	-197	6.17E-16	4.75E-13
234637_at	KRTAP4-5	-7.63	-197	1.38E-14	5.85E-12
234683_at	KRTAP4-11 /// KRTAP4-6 /// LOC100134267	-7.64	-200	2.23E-14	8.49E-12
221297_at	GPRC5D	-7.78	-219	5.32E-20	3.61E-16
234880_x_at	KRTAP1-3	-7.88	-235	1.93E-19	8.93E-16
220972_s_at	KRTAP9-9	-7.92	-242	3.54E-15	1.97E-12
1555673_at	KRTAP2-3 /// KRTAP2-4	-8.07	-268	1.43E-16	1.43E-13

234671_at	KRTAP4-2	-8.15	- 284	3.49E-16	3.00E-13
234684_s_at	KRTAP4-4	-8.24	- 301	2.58E-16	2.33E-13
1564974_at	KRTAP8-1	-8.65	- 401	3.14E-20	3.61E-16

Supplemental Table 5. AAP v NC Differential Expression

ProbeSetID	GeneSymbol	logFC	F	p.value	BH_fdr
203915_at	CXCL9	3.2	9.16	5.15E-09	2.57E-06
204580_at	MMP12	3.09	8.54	9.76E-08	2.19E-05
204533_at	CXCL10	2.75	6.71	2.73E-07	4.68E-05
209924_at	CCL18 /// LOC101060271	2.62	6.17	2.99E-06	2.67E-04
32128_at	CCL18 /// LOC101060271	2.53	5.79	6.63E-06	4.70E-04
206407_s_at	CCL13	1.9	3.74	4.83E-07	7.34E-05
216714_at	CCL13	1.81	3.52	1.43E-06	1.55E-04
205758_at	CD8A	1.66	3.17	1.08E-09	9.25E-07
213537_at	HLA-DPA1	1.62	3.07	1.58E-06	1.66E-04
1405_i_at	CCL5	1.6	3.02	5.85E-10	6.26E-07
205987_at	CD1C	1.52	2.87	9.75E-09	4.09E-06
205821_at	KLRC4-KLRK1 /// KLRK1	1.52	2.87	1.37E-09	1.01E-06
204655_at	CCL5	1.5	2.82	1.81E-09	1.26E-06
219386_s_at	SLAMF8	1.45	2.74	1.09E-07	2.37E-05
1558972_s_at	THEMIS	1.43	2.69	6.60E-07	9.10E-05
206666_at	GZMK	1.36	2.57	5.04E-06	3.86E-04
205488_at	GZMA	1.35	2.55	2.02E-06	1.96E-04
206749_at	CD1B	1.34	2.54	1.57E-04	4.72E-03
223710_at	CCL26	1.31	2.49	1.73E-04	5.07E-03
205831_at	CD2	1.28	2.43	2.25E-07	4.17E-05
1552584_at	IL12RB1	1.25	2.38	6.17E-07	8.76E-05
205419_at	GPR183	1.22	2.33	3.26E-06	2.81E-04
226218_at	IL7R	1.21	2.32	8.50E-06	5.65E-04
217147_s_at	TRAT1	1.19	2.29	6.86E-05	2.59E-03
229437_at	MIR155 /// MIR155HG	1.17	2.26	1.38E-06	1.52E-04
1559263_s_at	ZC3H12D	1.15	2.22	2.05E-08	7.35E-06
220005_at	P2RY13	1.15	2.22	2.02E-07	3.85E-05
205890_s_at	GABBR1 /// UBD	1.14	2.21	3.00E-05	1.42E-03
206637_at	P2RY14	1.13	2.2	2.26E-08	7.97E-06
209823_x_at	HLA-DQB1 /// LOC101060835	1.12	2.17	1.25E-05	7.40E-04
211796_s_at	TRBC1	1.12	2.17	7.91E-06	5.35E-04
227346_at	IKZF1	1.11	2.17	6.05E-07	8.67E-05
216935_at	LINC00302	1.1	2.15	1.16E-03	1.87E-02
238725_at	IRF1	1.09	2.13	4.55E-06	3.58E-04
1552870_s_at	AXDND1	1.09	2.13	2.95E-03	3.52E-02
206715_at	TFEC	1.09	2.13	4.19E-07	6.47E-05
207651_at	GPR171	1.09	2.13	2.12E-05	1.10E-03
206134_at	ADAMDEC1	1.08	2.11	1.07E-05	6.64E-04
216718_at	LINC00302	1.07	2.11	2.47E-03	3.13E-02
210915_x_at	TRBC1	1.07	2.1	1.81E-06	1.80E-04

229641_at	CCBE1	1.07	2.1	2.87E-06	2.60E-04
211657_at	CEACAM6	1.06	2.09	8.92E-04	1.57E-02
207861_at	CCL22	1.06	2.08	3.01E-05	1.42E-03
213193_x_at	TRBC1	1.05	2.07	8.11E-06	5.45E-04
207430_s_at	MSMB	1.05	2.07	4.39E-03	4.57E-02
213475_s_at	ITGAL	1.04	2.06	5.96E-06	4.37E-04
244363_at	ROS1	1.04	2.06	8.89E-04	1.57E-02
214617_at	PRF1	1.04	2.05	2.65E-04	6.74E-03
204891_s_at	LCK	1.04	2.05	1.25E-06	1.41E-04
227553_at	PIK3R5	1.04	2.05	7.89E-06	5.35E-04
224555_x_at	IL37	1.03	2.04	6.48E-04	1.27E-02
213539_at	CD3D	1.02	2.03	2.01E-07	3.85E-05
1564786_at	LOC338667	1.01	2.02	1.45E-03	2.18E-02
219666_at	MS4A6A	1.01	2.01	1.31E-06	1.46E-04
227266_s_at	FYB	1	2.01	1.78E-06	1.79E-04
242641_at	COL6A6	0.998	2	6.96E-04	1.33E-02
207466_at	GAL	0.997	2	4.69E-03	4.78E-02
211656_x_at	HLA-DQB1 /// LOC101060835	0.997	2	1.03E-05	6.46E-04
215049_x_at	CD163	0.995	1.99	1.44E-05	8.24E-04
204205_at	APOBEC3G	0.993	1.99	3.21E-06	2.79E-04
211339_s_at	ITK	0.991	1.99	7.47E-05	2.75E-03
236295_s_at	NLRC3	0.991	1.99	3.00E-06	2.67E-04
213240_s_at	KRT4	0.99	1.99	1.31E-03	2.03E-02
216920_s_at	TARP /// TRGC2	0.99	1.99	2.48E-04	6.48E-03
228362_s_at	FAM26F	0.986	1.98	6.50E-05	2.49E-03
1553062_at	MOGAT1	0.977	1.97	2.16E-03	2.86E-02
220358_at	BATF3	0.974	1.96	8.34E-07	1.05E-04
205686_s_at	CD86	0.97	1.96	3.02E-09	1.77E-06
210247_at	SYN2	0.969	1.96	2.66E-04	6.74E-03
216233_at	CD163	0.968	1.96	7.96E-05	2.89E-03
214038_at	CCL8	0.968	1.96	2.50E-04	6.52E-03
207277_at	CD209	0.965	1.95	1.13E-05	6.84E-04
1564474_at	ZBED3-AS1	0.963	1.95	5.67E-04	1.16E-02
229391_s_at	FAM26F	0.962	1.95	8.49E-05	3.03E-03
214450_at	CTSW	0.961	1.95	4.31E-05	1.85E-03
214321_at	NOV	0.961	1.95	1.97E-04	5.51E-03
219607_s_at	MS4A4A	0.96	1.95	1.07E-04	3.55E-03
210164_at	GZMB	0.96	1.94	4.99E-05	2.04E-03
231776_at	EOMES	0.958	1.94	2.59E-04	6.66E-03
244413_at	CLECL1	0.958	1.94	7.64E-06	5.20E-04
203416_at	CD53	0.957	1.94	1.04E-06	1.25E-04
236028_at	IBSP	0.953	1.94	9.48E-05	3.28E-03
220330_s_at	SAMSN1	0.95	1.93	1.98E-04	5.52E-03

204440_at	CD83	0.946	1.93	1.68E-05	9.27E-04
230937_at	LOC285835	0.946	1.93	5.65E-04	1.16E-02
210146_x_at	LILRB2	0.945	1.93	3.34E-04	8.04E-03
204446_s_at	ALOX5	0.943	1.92	5.73E-05	2.26E-03
236175_at	TRIM55	0.943	1.92	2.21E-03	2.91E-02
206247_at	MICB	0.942	1.92	6.04E-05	2.35E-03
229241_at	LDHD	0.939	1.92	9.57E-04	1.65E-02
229390_at	FAM26F	0.938	1.92	8.11E-05	2.92E-03
206932_at	CH25H	0.934	1.91	9.99E-05	3.40E-03
214913_at	ADAMTS3	0.934	1.91	6.71E-05	2.54E-03
44673_at	SIGLEC1	0.925	1.9	9.30E-06	6.02E-04
236782_at	SAMD3	0.923	1.9	2.37E-04	6.26E-03
215806_x_at	TARP /// TRGC2	0.922	1.89	4.32E-04	9.69E-03
209606_at	CYTIP	0.919	1.89	1.62E-05	9.01E-04
203645_s_at	CD163	0.919	1.89	4.24E-05	1.82E-03
232428_at	MOGAT2	0.912	1.88	4.06E-03	4.34E-02
204912_at	IL10RA	0.91	1.88	3.44E-08	1.07E-05
206545_at	CD28	0.909	1.88	8.95E-05	3.14E-03
206104_at	ISL1	0.907	1.88	6.38E-07	8.90E-05
206082_at	HCP5	0.907	1.87	2.76E-06	2.51E-04
230040_at	ADAMTS18	0.906	1.87	6.94E-04	1.33E-02
209816_at	PTCH1	0.903	1.87	3.14E-04	7.68E-03
214567_s_at	XCL1 /// XCL2	0.9	1.87	5.97E-06	4.37E-04
219594_at	NINJ2	0.898	1.86	3.98E-07	6.29E-05
227645_at	PIK3R5	0.896	1.86	8.18E-06	5.48E-04
206296_x_at	MAP4K1	0.896	1.86	2.24E-06	2.13E-04
230422_at	FPR3	0.894	1.86	5.71E-05	2.25E-03
203761_at	SLA	0.894	1.86	3.46E-07	5.61E-05
219836_at	ZBED2	0.893	1.86	2.76E-04	6.94E-03
204714_s_at	F5	0.89	1.85	2.10E-03	2.81E-02
205364_at	ACOX2	0.888	1.85	5.02E-03	4.98E-02
219519_s_at	SIGLEC1	0.887	1.85	4.51E-05	1.91E-03
203471_s_at	PLEK	0.885	1.85	5.30E-06	4.01E-04
230773_at	ZNF385D	0.883	1.84	4.26E-04	9.61E-03
210184_at	ITGAX	0.881	1.84	6.07E-06	4.40E-04
206227_at	CILP	0.881	1.84	2.09E-03	2.81E-02
1562364_at	GVINP1	0.88	1.84	1.01E-05	6.37E-04
208161_s_at	ABCC3	0.878	1.84	1.43E-06	1.55E-04
228869_at	SNX20	0.877	1.84	3.32E-06	2.84E-04
227677_at	JAK3	0.875	1.83	2.15E-05	1.11E-03
203757_s_at	CEACAM6	0.874	1.83	1.59E-03	2.34E-02
214012_at	ERAP1	0.872	1.83	1.46E-03	2.19E-02
228094_at	AMICA1	0.867	1.82	1.09E-06	1.30E-04

226142_at	GLIPR1	0.866	1.82	4.19E-06	3.35E-04
228532_at	C1orf162	0.865	1.82	1.02E-06	1.24E-04
205269_at	LCP2	0.861	1.82	2.53E-05	1.26E-03
205786_s_at	ITGAM	0.86	1.82	5.74E-05	2.26E-03
223343_at	MS4A7	0.86	1.81	6.69E-04	1.30E-02
210889_s_at	FCGR2B	0.859	1.81	4.64E-06	3.63E-04
222945_x_at	OLAH	0.858	1.81	4.48E-03	4.64E-02
214339_s_at	MAP4K1	0.858	1.81	2.73E-06	2.49E-04
202953_at	C1QB	0.856	1.81	2.52E-07	4.46E-05
214399_s_at	KRT4	0.855	1.81	2.87E-03	3.45E-02
207901_at	IL12B	0.855	1.81	8.98E-05	3.15E-03
205101_at	CIITA	0.854	1.81	9.75E-06	6.22E-04
220485_s_at	SIRPG	0.853	1.81	4.69E-05	1.95E-03
229510_at	MS4A14	0.853	1.81	7.11E-05	2.65E-03
239427_at	SLAMF1	0.848	1.8	9.37E-06	6.04E-04
211144_x_at	TARP /// TRGC2	0.848	1.8	2.74E-04	6.89E-03
236234_at	PDE1A	0.846	1.8	1.07E-04	3.55E-03
229383_at	1-Mar	0.846	1.8	3.13E-06	2.75E-04
205067_at	IL1B	0.844	1.8	4.49E-04	9.96E-03
235221_at	CBLN3	0.843	1.79	3.17E-03	3.70E-02
206214_at	PLA2G7	0.838	1.79	7.34E-04	1.38E-02
221978_at	HLA-F	0.837	1.79	1.16E-05	6.98E-04
221080_s_at	DENND1C	0.836	1.79	1.26E-04	4.03E-03
211991_s_at	HLA-DPA1	0.835	1.78	7.95E-07	1.02E-04
214219_x_at	MAP4K1	0.835	1.78	1.62E-06	1.69E-04
214981_at	POSTN	0.834	1.78	4.67E-05	1.95E-03
244385_at	KDM4C	0.834	1.78	2.40E-04	6.32E-03
209813_x_at	TARP	0.833	1.78	3.70E-04	8.64E-03
227168_at	MIAT	0.83	1.78	9.62E-05	3.31E-03
219385_at	SLAMF8	0.83	1.78	1.82E-05	9.77E-04
206366_x_at	XCL1	0.827	1.77	2.31E-05	1.18E-03
210538_s_at	BIRC3	0.825	1.77	5.68E-04	1.16E-02
235557_at	GPAT2	0.825	1.77	5.65E-04	1.16E-02
230983_at	FAM129C	0.822	1.77	2.00E-03	2.72E-02
220560_at	C11orf21	0.822	1.77	3.84E-05	1.69E-03
239237_at	LOC100506776	0.817	1.76	7.93E-04	1.46E-02
210176_at	TLR1	0.817	1.76	4.36E-06	3.45E-04
205180_s_at	ADAM8	0.817	1.76	3.31E-05	1.52E-03
205456_at	CD3E	0.815	1.76	5.45E-07	8.06E-05
210038_at	PRKCQ	0.812	1.76	3.12E-03	3.65E-02
239529_at	C5orf20 /// TIFAB	0.812	1.76	1.19E-06	1.37E-04
240197_at	SYN2	0.812	1.76	2.58E-05	1.28E-03
205270_s_at	LCP2	0.811	1.75	5.32E-06	4.01E-04

206785_s_at	KLRC1 /// KLRC2	0.81	1.75	3.62E-05	1.62E-03
228658_at	MIAT	0.81	1.75	9.70E-04	1.67E-02
209498_at	CEACAM1	0.808	1.75	2.57E-03	3.21E-02
209879_at	SELPLG	0.804	1.75	2.35E-05	1.19E-03
209975_at	CYP2E1	0.802	1.74	1.75E-04	5.07E-03
209734_at	NCKAP1L	0.801	1.74	1.84E-07	3.64E-05
205291_at	IL2RB	0.8	1.74	3.18E-05	1.47E-03
231930_at	ELMOD1 /// LOC643923	0.798	1.74	1.91E-03	2.64E-02
206761_at	CD96	0.798	1.74	5.68E-05	2.24E-03
222134_at	DDO	0.795	1.74	1.74E-04	5.07E-03
228381_at	ATF7IP2 /// LOC100287628	0.794	1.73	1.34E-03	2.07E-02
223562_at	PARVG	0.794	1.73	3.62E-05	1.62E-03
1563369_at	LINC00173	0.791	1.73	1.13E-03	1.85E-02
210163_at	CXCL11	0.788	1.73	1.29E-03	2.01E-02
232311_at	B2M	0.787	1.73	1.18E-05	7.11E-04
235080_at	CLUAP1	0.785	1.72	2.23E-04	6.01E-03
206199_at	CEACAM7	0.785	1.72	3.19E-03	3.71E-02
204614_at	SERPINB2	0.783	1.72	7.33E-04	1.38E-02
31637_s_at	NR1D1 /// THRA	0.781	1.72	1.30E-03	2.02E-02
219045_at	RHOF	0.779	1.72	5.19E-07	7.73E-05
228573_at	ANTXR2	0.777	1.71	5.32E-04	1.12E-02
203828_s_at	IL32	0.777	1.71	2.69E-05	1.31E-03
235175_at	GBP4	0.777	1.71	6.42E-05	2.46E-03
204279_at	PSMB9	0.776	1.71	1.94E-05	1.03E-03
229802_at	WISP1	0.775	1.71	3.64E-04	8.57E-03
208885_at	LCP1	0.774	1.71	1.91E-06	1.88E-04
222088_s_at	SLC2A14 /// SLC2A3	0.774	1.71	5.73E-04	1.17E-02
242268_at	CELF2	0.774	1.71	1.29E-04	4.10E-03
210395_x_at	MYL4	0.773	1.71	8.28E-04	1.49E-02
208438_s_at	FGR	0.773	1.71	1.76E-05	9.55E-04
241981_at	FAM20A	0.772	1.71	7.06E-04	1.35E-02
219890_at	CLEC5A	0.769	1.7	1.06E-03	1.77E-02
239178_at	FGF9	0.768	1.7	9.64E-05	3.31E-03
227609_at	EPSTI1	0.766	1.7	2.81E-03	3.42E-02
206978_at	CCR2	0.765	1.7	7.43E-04	1.39E-02
206914_at	CRTAM	0.765	1.7	1.91E-05	1.02E-03
212671_s_at	HLA-DQA1 /// HLA-DQA2 /// LOC100509457	0.762	1.7	4.38E-03	4.57E-02
244572_at	KY	0.761	1.69	7.59E-04	1.42E-02
208121_s_at	PTPRO	0.759	1.69	1.61E-05	8.97E-04
230550_at	MS4A6A	0.757	1.69	1.39E-04	4.34E-03
1559194_a_at	CLEC4GP1	0.756	1.69	4.49E-04	9.96E-03
215332_s_at	CD8B /// LOC100996919	0.755	1.69	3.16E-08	1.03E-05

336_at	TBXA2R	0.754	1.69	2.72E-03	3.34E-02
231093_at	FCRL3	0.753	1.69	4.02E-04	9.22E-03
212587_s_at	PTPRC	0.753	1.69	5.26E-04	1.11E-02
1556472_s_a t	SCML4	0.751	1.68	2.08E-04	5.74E-03
226136_at	GLIPR1	0.75	1.68	4.23E-06	3.37E-04
232581_x_at	HIVEP3	0.748	1.68	1.81E-03	2.56E-02
213849_s_at	PPP2R2B	0.745	1.68	4.92E-06	3.79E-04
204502_at	SAMHD1	0.745	1.68	8.18E-06	5.48E-04
1553311_at	C20orf197	0.743	1.67	1.09E-04	3.60E-03
206625_at	PRPH2	0.742	1.67	4.70E-04	1.02E-02
230391_at	CD84	0.742	1.67	5.28E-05	2.13E-03
227353_at	TMC8	0.74	1.67	4.63E-04	1.02E-02
229597_s_at	WDFY4	0.739	1.67	9.70E-06	6.21E-04
232081_at	ABCG1	0.737	1.67	7.95E-05	2.89E-03
204014_at	DUSP4	0.735	1.66	4.04E-04	9.26E-03
231898_x_at	SOX2-OT	0.735	1.66	1.44E-03	2.16E-02
205859_at	LY86	0.734	1.66	2.53E-04	6.56E-03
231124_x_at	LY9	0.734	1.66	3.19E-04	7.79E-03
224451_x_at	ARHGAP9	0.734	1.66	4.06E-05	1.76E-03
209969_s_at	STAT1	0.733	1.66	4.04E-04	9.26E-03
205804_s_at	TRAF3IP3	0.732	1.66	6.08E-04	1.22E-02
226603_at	SAMD9L	0.732	1.66	8.48E-05	3.03E-03
207757_at	ZFP2	0.73	1.66	1.41E-04	4.39E-03
1555993_at	CACNA1D	0.729	1.66	3.47E-04	8.27E-03
204760_s_at	NR1D1 /// THRA	0.729	1.66	4.06E-03	4.34E-02
202957_at	HCLS1	0.729	1.66	3.48E-05	1.57E-03
1557474_at	LOC284578	0.727	1.66	4.59E-03	4.72E-02
212613_at	BTN3A2	0.726	1.65	2.27E-03	2.96E-02
207957_s_at	PRKCB	0.725	1.65	1.87E-03	2.61E-02
230782_at	SORD	0.722	1.65	4.67E-05	1.95E-03
229534_at	ACOT4	0.721	1.65	1.42E-03	2.15E-02
229218_at	COL1A2	0.719	1.65	1.18E-03	1.89E-02
226612_at	UBE2QL1	0.717	1.64	9.61E-05	3.31E-03
204122_at	TYROBP	0.717	1.64	1.72E-05	9.43E-04
230753_at	PATL2	0.716	1.64	1.14E-04	3.74E-03
244033_at	CEP128	0.715	1.64	5.94E-05	2.32E-03
219723_x_at	AGPAT3	0.714	1.64	2.20E-04	5.97E-03
39402_at	IL1B	0.713	1.64	6.68E-04	1.30E-02
206623_at	PDE6A	0.713	1.64	4.54E-03	4.68E-02
218795_at	ACP6	0.712	1.64	1.25E-03	1.97E-02
215303_at	DCLK1	0.711	1.64	1.25E-03	1.98E-02
210439_at	ICOS	0.71	1.64	5.91E-04	1.20E-02

229947_at	PI15	0.709	1.64	9.71E-04	1.67E-02
231581_at	LINC00664	0.708	1.63	2.48E-03	3.14E-02
226818_at	MPEG1	0.708	1.63	1.50E-06	1.60E-04
217478_s_at	HLA-DMA	0.708	1.63	6.25E-07	8.76E-05
202531_at	IRF1	0.708	1.63	1.15E-04	3.75E-03
206181_at	SLAMF1	0.706	1.63	6.22E-05	2.40E-03
1554704_at	ATP8B3	0.706	1.63	6.52E-06	4.64E-04
211734_s_at	FCER1A	0.706	1.63	6.34E-05	2.44E-03
229723_at	TAGAP	0.705	1.63	2.53E-03	3.19E-02
205997_at	ADAM28	0.704	1.63	4.84E-05	2.00E-03
211990_at	HLA-DPA1	0.703	1.63	2.79E-03	3.41E-02
214203_s_at	PRODH	0.702	1.63	3.35E-04	8.04E-03
210385_s_at	ERAP1	0.701	1.63	2.59E-03	3.22E-02
224356_x_at	MS4A6A	0.701	1.63	6.42E-06	4.59E-04
224357_s_at	MS4A4A	0.699	1.62	5.43E-05	2.17E-03
238567_at	SGPP2	0.698	1.62	2.35E-03	3.03E-02
1560396_at	KLHL6	0.698	1.62	2.56E-03	3.21E-02
212588_at	PTPRC	0.697	1.62	1.49E-05	8.45E-04
204959_at	MNDA	0.697	1.62	4.87E-04	1.05E-02
212873_at	HMHA1	0.697	1.62	3.02E-05	1.42E-03
214651_s_at	HOXA10-HOXA9 /// HOXA9 /// MIR196B	0.696	1.62	2.80E-04	7.00E-03
204994_at	MX2	0.696	1.62	1.93E-04	5.44E-03
210895_s_at	CD86	0.692	1.62	4.56E-06	3.58E-04
206576_s_at	CEACAM1	0.691	1.61	2.79E-03	3.40E-02
230440_at	ZNF469	0.69	1.61	2.32E-05	1.18E-03
209905_at	HOXA10-HOXA9 /// HOXA9 /// MIR196B	0.689	1.61	5.77E-04	1.18E-02
1560531_at	LCE1B	0.689	1.61	6.85E-04	1.32E-02
1559186_at	PRKXP1	0.688	1.61	1.66E-03	2.41E-02
231175_at	BEND6	0.687	1.61	4.45E-04	9.92E-03
219319_at	HIF3A	0.687	1.61	2.55E-03	3.20E-02
229649_at	NRXN3	0.686	1.61	2.08E-04	5.74E-03
206337_at	CCR7	0.685	1.61	7.32E-05	2.71E-03
244276_at	KLB	0.684	1.61	2.85E-03	3.45E-02
219616_at	ACSS3	0.684	1.61	5.30E-04	1.11E-02
223687_s_at	LY6K	0.681	1.6	2.14E-03	2.85E-02
1554240_a_at	ITGAL	0.681	1.6	2.14E-05	1.11E-03
229634_at	TMEM139	0.681	1.6	2.72E-03	3.34E-02
204259_at	MMP7	0.681	1.6	1.75E-04	5.07E-03
206693_at	IL7	0.677	1.6	5.53E-05	2.20E-03
213479_at	NPTX2	0.677	1.6	1.43E-03	2.16E-02
1569886_a_at	GLB1L3	0.676	1.6	9.23E-04	1.62E-02
229497_at	ANKDD1A	0.674	1.6	3.02E-05	1.42E-03

225033_at	ST3GAL1	0.674	1.6	2.31E-05	1.18E-03
237974_at	ABHD12B /// MIR4454	0.674	1.6	1.09E-05	6.72E-04
237493_at	IL22RA2	0.673	1.59	4.93E-06	3.80E-04
205098_at	CCR1	0.672	1.59	2.91E-05	1.39E-03
204197_s_at	RUNX3	0.672	1.59	4.12E-07	6.40E-05
242286_at	GRIN2A	0.672	1.59	1.03E-04	3.47E-03
204153_s_at	MFNG	0.67	1.59	1.46E-04	4.48E-03
207900_at	CCL17	0.67	1.59	8.37E-04	1.51E-02
219452_at	DPEP2	0.669	1.59	3.23E-05	1.49E-03
213603_s_at	RAC2	0.668	1.59	4.75E-06	3.71E-04
208146_s_at	CPVL	0.668	1.59	8.31E-05	2.98E-03
219820_at	SLC6A16	0.667	1.59	2.36E-03	3.04E-02
205295_at	CKMT2	0.667	1.59	1.05E-03	1.76E-02
229800_at	DCLK1	0.666	1.59	1.70E-03	2.44E-02
204411_at	KIF21B	0.665	1.59	3.31E-03	3.81E-02
206513_at	AIM2	0.665	1.59	1.20E-04	3.89E-03
232458_at	COL3A1	0.664	1.58	2.69E-03	3.32E-02
225701_at	AKNA	0.664	1.58	5.90E-06	4.33E-04
210607_at	FLT3LG	0.664	1.58	1.37E-04	4.30E-03
219799_s_at	DHRS9	0.664	1.58	1.97E-03	2.70E-02
223280_x_at	MS4A6A	0.663	1.58	2.04E-05	1.07E-03
236514_at	ACOT8	0.662	1.58	1.35E-05	7.91E-04
205213_at	ACAP1	0.658	1.58	1.45E-05	8.24E-04
206991_s_at	CCR5	0.654	1.57	6.03E-06	4.39E-04
202524_s_at	SPOCK2	0.654	1.57	1.03E-03	1.74E-02
218345_at	TMEM176A	0.652	1.57	2.06E-04	5.71E-03
208894_at	HLA-DRA	0.651	1.57	5.76E-04	1.18E-02
229779_at	COL4A4	0.651	1.57	4.36E-03	4.55E-02
226777_at	ADAM12 /// ADAM12-OT1	0.651	1.57	3.62E-04	8.53E-03
210982_s_at	HLA-DRA	0.65	1.57	2.79E-04	6.99E-03
204670_x_at	HLA-DRB1 /// HLA-DRB4 /// LOC100507709 /// LOC100507714	0.65	1.57	1.47E-04	4.50E-03
236253_at	ZNF546	0.648	1.57	4.67E-04	1.02E-02
203485_at	RTN1	0.648	1.57	6.55E-04	1.28E-02
203547_at	CD4	0.647	1.57	6.01E-07	8.65E-05
230110_at	MCOLN2	0.645	1.56	4.19E-03	4.44E-02
227210_at	SFMBT2	0.644	1.56	1.00E-04	3.41E-03
235291_s_at	FLJ32255	0.644	1.56	1.72E-03	2.46E-02
220014_at	PRR16	0.643	1.56	6.76E-04	1.31E-02
235597_s_at	RGPD1 /// RGPD2	0.641	1.56	4.41E-03	4.58E-02
225598_at	SLC45A4	0.641	1.56	6.68E-04	1.30E-02
238422_at	LBX2-AS1	0.64	1.56	2.86E-03	3.45E-02
237411_at	ADAMTS6	0.637	1.55	1.36E-03	2.09E-02

226474_at	NLRC5	0.634	1.55	4.94E-04	1.06E-02
211795_s_at	FYB	0.633	1.55	3.06E-04	7.52E-03
235742_at	RHOC	0.633	1.55	4.57E-05	1.92E-03
235518_at	SLC8A1	0.632	1.55	5.51E-04	1.14E-02
1568794_at	LOC643355	0.632	1.55	1.26E-03	1.98E-02
236995_x_at	TFEC	0.631	1.55	6.14E-07	8.76E-05
236270_at	NFATC4	0.63	1.55	8.93E-04	1.57E-02
204575_s_at	MMP19	0.63	1.55	4.35E-04	9.73E-03
207238_s_at	PTPRC	0.628	1.55	5.45E-04	1.13E-02
204629_at	PARVB	0.628	1.55	6.96E-04	1.33E-02
244598_at	LCP2	0.628	1.55	1.76E-04	5.09E-03
230631_s_at	IL10RB-AS1	0.628	1.55	6.98E-04	1.34E-02
203104_at	CSF1R	0.627	1.54	2.41E-06	2.26E-04
1559584_a_at	C16orf54	0.627	1.54	2.31E-03	2.99E-02
35974_at	LRMP	0.627	1.54	7.48E-04	1.40E-02
211372_s_at	IL1R2	0.625	1.54	3.42E-03	3.89E-02
225353_s_at	C1QC	0.622	1.54	1.28E-05	7.54E-04
205266_at	LIF	0.622	1.54	2.58E-03	3.22E-02
241866_at	SLC16A7	0.622	1.54	7.62E-04	1.42E-02
44790_s_at	KIAA0226L	0.621	1.54	4.51E-04	1.00E-02
232024_at	GIMAP2	0.621	1.54	8.55E-05	3.04E-03
209426_s_at	AMACR /// C1QTNF3 /// C1QTNF3-AMACR	0.62	1.54	2.40E-03	3.07E-02
239929_at	PM20D1	0.618	1.53	3.15E-03	3.67E-02
222592_s_at	ACSL5	0.617	1.53	3.71E-03	4.10E-02
213418_at	HSPA6	0.617	1.53	1.45E-03	2.17E-02
209670_at	TRAC	0.616	1.53	1.43E-05	8.18E-04
215116_s_at	DNM1	0.615	1.53	1.24E-03	1.97E-02
205403_at	IL1R2	0.615	1.53	1.71E-04	5.02E-03
204057_at	IRF8	0.614	1.53	1.23E-03	1.96E-02
219232_s_at	EGLN3	0.614	1.53	3.85E-04	8.96E-03
1557283_a_at	ZNF519	0.613	1.53	5.69E-04	1.16E-02
202075_s_at	PLTP	0.613	1.53	2.03E-05	1.07E-03
205432_at	OVGP1	0.613	1.53	1.79E-03	2.53E-02
204114_at	NID2	0.613	1.53	1.20E-03	1.92E-02
220532_s_at	TMEM176B	0.611	1.53	5.91E-05	2.31E-03
211742_s_at	EVI2B	0.61	1.53	3.19E-04	7.78E-03
228943_at	MAP6	0.609	1.52	2.50E-03	3.15E-02
218627_at	DRAM1	0.608	1.52	3.29E-04	7.96E-03
232543_x_at	ARHGAP9	0.608	1.52	9.11E-05	3.19E-03
207554_x_at	TBXA2R	0.607	1.52	5.09E-04	1.08E-02
224210_s_at	PXMP4	0.607	1.52	1.43E-03	2.16E-02
230480_at	PIWIL4	0.607	1.52	1.45E-04	4.48E-03

221588_x_at	ALDH6A1	0.606	1.52	3.06E-03	3.60E-02
219734_at	SIDT1	0.606	1.52	1.29E-04	4.08E-03
235574_at	GBP4	0.606	1.52	9.94E-05	3.40E-03
220309_at	TTC22	0.605	1.52	2.71E-03	3.34E-02
235004_at	RBM24	0.605	1.52	4.75E-03	4.83E-02
236886_at	LOC100049716	0.605	1.52	2.09E-03	2.81E-02
217525_at	OLFML1	0.603	1.52	1.09E-03	1.80E-02
204777_s_at	MAL	0.601	1.52	4.05E-03	4.33E-02
223655_at	CD163L1	0.601	1.52	3.77E-07	5.98E-05
203932_at	HLA-DMB	0.6	1.52	1.85E-07	3.64E-05
231773_at	ANGPTL1	0.598	1.51	1.10E-03	1.81E-02
230867_at	COL6A6	0.598	1.51	1.30E-03	2.02E-02
209765_at	ADAM19	0.598	1.51	8.43E-04	1.51E-02
216054_x_at	MYL4	0.597	1.51	5.67E-04	1.16E-02
242517_at	KISS1R	0.597	1.51	4.89E-04	1.05E-02
215051_x_at	AIF1	0.597	1.51	6.67E-06	4.72E-04
210321_at	GZMH	0.596	1.51	8.74E-06	5.75E-04
227194_at	FAM3B	0.595	1.51	3.57E-03	4.00E-02
229029_at	CAMK4	0.595	1.51	2.88E-04	7.15E-03
210088_x_at	MYL4	0.594	1.51	4.27E-03	4.50E-02
226743_at	SLFN11	0.594	1.51	2.07E-05	1.08E-03
207415_at	PLA2R1	0.594	1.51	1.63E-04	4.85E-03
235343_at	VASH2	0.593	1.51	2.79E-03	3.41E-02
1564276_at	C5orf56	0.593	1.51	1.93E-04	5.43E-03
226878_at	HLA-DOA	0.593	1.51	1.29E-03	2.01E-02
209901_x_at	AIF1	0.593	1.51	3.05E-05	1.43E-03
232792_at	TRIM69	0.592	1.51	4.90E-04	1.05E-02
219574_at	1-Mar	0.591	1.51	1.45E-04	4.48E-03
223922_x_at	MS4A6A	0.59	1.51	8.95E-05	3.14E-03
41577_at	PPP1R16B	0.59	1.51	3.33E-04	8.02E-03
244251_at	LCP2	0.589	1.5	1.17E-06	1.35E-04
226972_s_at	CCDC136	0.588	1.5	1.93E-03	2.66E-02
205159_at	CSF2RB	0.587	1.5	5.70E-05	2.25E-03
37966_at	PARVB	0.587	1.5	1.49E-03	2.22E-02
227285_at	C1orf51	0.587	1.5	2.22E-03	2.92E-02
207072_at	IL18RAP	0.587	1.5	7.08E-04	1.35E-02
226906_s_at	ARHGAP9	0.586	1.5	1.73E-05	9.47E-04
215807_s_at	PLXNB1	-0.585	-1.5	8.56E-04	1.53E-02
200906_s_at	PALLD	-0.586	-1.5	2.52E-04	6.56E-03
217787_s_at	GALNT2	-0.586	-1.5	1.08E-07	2.37E-05
203707_at	LINC00921 /// ZNF263	-0.587	-1.5	9.10E-08	2.06E-05
210954_s_at	TSC22D2	-0.587	-1.5	5.64E-06	4.19E-04
240024_at	SEC14L2	-0.587	-1.5	4.66E-03	4.77E-02

226168_at	ZFAND2B	-0.588	-1.5	9.47E-05	3.28E-03
218916_at	ZNF768	-0.588	-1.5	1.59E-05	8.90E-04
201148_s_at	TIMP3	-0.588	-1.5	4.12E-04	9.39E-03
218574_s_at	LMCD1	-0.588	-1.5	2.73E-04	6.88E-03
227069_at	CUX1	-0.589	-1.5	1.07E-03	1.78E-02
238001_at	KCTD6	-0.589	-1.5	5.51E-07	8.11E-05
201846_s_at	RYBP	-0.589	-1.5	9.35E-04	1.63E-02
200644_at	MARCKSL1	-0.59	-1.5	1.15E-03	1.86E-02
203499_at	EPHA2	-0.59	-1.51	3.77E-06	3.09E-04
244118_at	GABRA1	-0.59	-1.51	5.03E-05	2.05E-03
219229_at	SLCO3A1	-0.593	-1.51	9.04E-07	1.12E-04
208978_at	CRIP2	-0.593	-1.51	3.44E-06	2.92E-04
209873_s_at	PKP3	-0.595	-1.51	1.68E-06	1.71E-04
210655_s_at	FOXO3 /// FOXO3B	-0.596	-1.51	1.26E-04	4.04E-03
222927_s_at	CPLX3	-0.596	-1.51	5.77E-07	8.39E-05
239355_at	GMCL1	-0.597	-1.51	5.75E-08	1.55E-05
220362_at	PSORS1C1	-0.597	-1.51	6.38E-04	1.26E-02
219660_s_at	ATP8A2	-0.598	-1.51	5.58E-04	1.15E-02
206137_at	RIMS2	-0.598	-1.51	5.09E-05	2.06E-03
201183_s_at	CHD4	-0.6	-1.52	1.02E-04	3.45E-03
204557_s_at	DZIP1	-0.6	-1.52	2.55E-03	3.20E-02
204761_at	USP6NL	-0.601	-1.52	2.05E-07	3.87E-05
217080_s_at	HOMER2	-0.601	-1.52	2.83E-07	4.81E-05
221920_s_at	SLC25A37	-0.602	-1.52	1.47E-04	4.50E-03
209126_x_at	KRT6B	-0.604	-1.52	2.31E-04	6.14E-03
238741_at	FAM83A	-0.604	-1.52	1.10E-04	3.63E-03
1555074_a_at	KCNH5	-0.605	-1.52	2.90E-03	3.48E-02
210417_s_at	PI4KB	-0.605	-1.52	8.99E-04	1.58E-02
212816_s_at	CBS	-0.605	-1.52	1.07E-04	3.55E-03
215198_s_at	CALD1	-0.608	-1.52	1.27E-04	4.06E-03
221455_s_at	WNT3	-0.608	-1.52	1.67E-09	1.21E-06
230830_at	SLC51B	-0.608	-1.52	1.60E-05	8.93E-04
212157_at	SDC2	-0.609	-1.53	1.36E-03	2.09E-02
209280_at	MRC2	-0.609	-1.53	2.36E-06	2.22E-04
207826_s_at	ID3	-0.609	-1.53	5.91E-07	8.56E-05
212942_s_at	KIAA1199	-0.609	-1.53	1.17E-06	1.35E-04
207233_s_at	MITF	-0.609	-1.53	4.08E-05	1.77E-03
211367_s_at	CASP1	-0.61	-1.53	5.06E-04	1.08E-02
222153_at	MYEF2	-0.61	-1.53	7.53E-04	1.41E-02
214877_at	CDKAL1	-0.611	-1.53	1.88E-03	2.61E-02
231935_at	ARPP21	-0.611	-1.53	1.44E-06	1.56E-04
222528_s_at	SLC25A37	-0.612	-1.53	6.92E-05	2.60E-03

206044_s_at	BRAF	-0.612	-1.53	1.03E-04	3.46E-03
211219_s_at	LHX2	-0.612	-1.53	2.85E-03	3.45E-02
224762_at	SERINC2	-0.614	-1.53	1.73E-06	1.75E-04
203904_x_at	CD82	-0.614	-1.53	6.37E-05	2.45E-03
1561030_at	TMC7	-0.614	-1.53	4.11E-04	9.37E-03
206191_at	ENTPD3	-0.616	-1.53	6.54E-04	1.28E-02
228293_at	DEPDC7	-0.616	-1.53	9.69E-07	1.19E-04
206367_at	REN	-0.617	-1.53	5.97E-04	1.20E-02
229290_at	DAPL1	-0.617	-1.53	7.92E-07	1.02E-04
209591_s_at	BMP7	-0.617	-1.53	1.18E-04	3.82E-03
205732_s_at	NCOA2	-0.618	-1.53	3.90E-06	3.18E-04
226858_at	CSNK1E /// LOC100996460	-0.618	-1.53	7.07E-07	9.53E-05
244758_at	SCAND3	-0.619	-1.54	1.49E-04	4.52E-03
212662_at	PVR	-0.619	-1.54	2.58E-05	1.28E-03
220962_s_at	PADI1	-0.622	-1.54	1.24E-07	2.65E-05
219671_at	HPCAL4	-0.623	-1.54	1.64E-06	1.69E-04
216086_at	SV2C	-0.624	-1.54	8.93E-06	5.85E-04
224397_s_at	TMTC1	-0.626	-1.54	1.02E-03	1.73E-02
242583_at	STON2	-0.626	-1.54	1.22E-05	7.27E-04
1557034_s_at	LOC100134445 /// LOC100288778 /// WASH1 /// WASH2P /// WASH3P /// WASH5P /// WASH7P	-0.626	-1.54	6.41E-04	1.26E-02
1568954_s_at	C16orf72	-0.627	-1.54	2.10E-06	2.02E-04
226390_at	STARD4	-0.628	-1.55	1.72E-04	5.05E-03
205268_s_at	ADD2	-0.63	-1.55	5.36E-09	2.64E-06
219511_s_at	SNCAIP	-0.63	-1.55	4.77E-04	1.03E-02
209078_s_at	TXN2	-0.631	-1.55	2.11E-04	5.79E-03
230498_at	MCHR1	-0.634	-1.55	1.38E-03	2.11E-02
225950_at	SAMD8	-0.634	-1.55	2.14E-07	4.01E-05
211361_s_at	SERPINB13	-0.635	-1.55	1.56E-06	1.65E-04
235977_at	LONRF2	-0.635	-1.55	1.73E-04	5.07E-03
220244_at	LINC00312	-0.635	-1.55	1.74E-04	5.07E-03
203018_s_at	SSX2IP	-0.636	-1.55	3.47E-05	1.56E-03
232069_at	KIF26A	-0.636	-1.55	4.13E-05	1.78E-03
230464_at	S1PR5	-0.636	-1.55	6.71E-04	1.30E-02
229097_at	DIAPH3	-0.638	-1.56	9.33E-04	1.63E-02
208613_s_at	FLNB	-0.639	-1.56	4.20E-05	1.80E-03
207268_x_at	ABI2	-0.64	-1.56	1.04E-08	4.31E-06
205255_x_at	TCF7	-0.64	-1.56	2.10E-09	1.36E-06
213606_s_at	ARHGDIA	-0.641	-1.56	6.72E-04	1.30E-02
1555007_s_at	WDR66	-0.641	-1.56	3.11E-05	1.45E-03
1556316_s_at	LOC284889	-0.643	-1.56	2.28E-05	1.17E-03
206427_s_at	MLANA	-0.645	-1.56	6.94E-05	2.60E-03

220067_at	SPTBN5	-0.645	-1.56	4.68E-03	4.77E-02
37425_g_at	CCHCR1	-0.647	-1.57	1.64E-06	1.69E-04
211002_s_at	TRIM29	-0.647	-1.57	8.65E-04	1.54E-02
220527_at	MRPL20	-0.647	-1.57	8.65E-06	5.72E-04
239586_at	FAM83A	-0.649	-1.57	3.29E-06	2.82E-04
231867_at	TENM2	-0.65	-1.57	4.63E-04	1.02E-02
203798_s_at	VSNL1	-0.652	-1.57	4.01E-05	1.75E-03
228786_at	PTCHD3P1	-0.652	-1.57	3.79E-05	1.67E-03
226637_at	UBE2H	-0.652	-1.57	6.70E-06	4.72E-04
1559256_at	MAGI1	-0.654	-1.57	1.49E-06	1.60E-04
226590_at	ZNF618	-0.655	-1.57	8.14E-04	1.48E-02
1553352_x_at	ERVW-1	-0.655	-1.57	7.22E-08	1.79E-05
204751_x_at	DSC2	-0.655	-1.58	5.99E-05	2.34E-03
201105_at	LGALS1	-0.658	-1.58	1.57E-03	2.31E-02
222784_at	SMOC1	-0.658	-1.58	4.28E-03	4.50E-02
223679_at	CTNNB1	-0.658	-1.58	1.76E-03	2.50E-02
215817_at	SERPINB13	-0.658	-1.58	8.54E-07	1.07E-04
204132_s_at	FOXO3 /// FOXO3B	-0.66	-1.58	4.71E-04	1.02E-02
1552365_at	SCIN	-0.66	-1.58	1.17E-03	1.88E-02
226952_at	EAF1	-0.662	-1.58	3.26E-08	1.05E-05
232579_at	LOC100134229	-0.663	-1.58	1.22E-05	7.26E-04
204903_x_at	ATG4B	-0.665	-1.59	2.57E-06	2.37E-04
213439_x_at	RUNDC3A	-0.666	-1.59	6.23E-07	8.76E-05
226767_s_at	FAHD1	-0.667	-1.59	1.50E-03	2.23E-02
1553711_a_at	FAM218A	-0.668	-1.59	4.05E-04	9.28E-03
228527_s_at	SLC25A37	-0.668	-1.59	7.44E-06	5.09E-04
222853_at	FLRT3	-0.67	-1.59	2.82E-05	1.36E-03
226027_at	SWI5	-0.67	-1.59	4.06E-07	6.36E-05
226535_at	ITGB6	-0.671	-1.59	3.27E-05	1.50E-03
202744_at	SLC20A2	-0.671	-1.59	8.38E-08	1.97E-05
202416_at	DNAJC7	-0.671	-1.59	7.91E-07	1.02E-04
228284_at	TLE1	-0.672	-1.59	4.09E-05	1.77E-03
201860_s_at	PLAT	-0.673	-1.59	2.34E-05	1.19E-03
205595_at	DSG3	-0.674	-1.6	2.10E-04	5.79E-03
220407_s_at	TGFB2	-0.674	-1.6	2.77E-03	3.39E-02
204556_s_at	DZIP1	-0.674	-1.6	9.36E-04	1.63E-02
226313_at	C10orf35	-0.676	-1.6	2.50E-07	4.46E-05
239719_at	CD109	-0.677	-1.6	3.47E-06	2.92E-04
206758_at	EDN2	-0.677	-1.6	8.00E-05	2.90E-03
226494_at	CAMSAP3	-0.677	-1.6	4.03E-06	3.28E-04
220011_at	AUNIP	-0.677	-1.6	3.36E-05	1.53E-03
209243_s_at	PEG3	-0.679	-1.6	8.87E-05	3.12E-03

202693_s_at	STK17A	-0.68	-1.6	2.04E-05	1.07E-03
201616_s_at	CALD1	-0.68	-1.6	4.92E-06	3.79E-04
201110_s_at	THBS1	-0.681	-1.6	3.30E-03	3.80E-02
215547_at	TSC22D2	-0.683	-1.61	1.50E-07	3.10E-05
206375_s_at	HSPB3	-0.685	-1.61	7.08E-04	1.35E-02
231395_at	ATP8A2	-0.685	-1.61	1.14E-04	3.74E-03
220489_s_at	SERINC2	-0.686	-1.61	3.75E-05	1.66E-03
205428_s_at	CALB2	-0.688	-1.61	6.58E-04	1.29E-02
206109_at	FUT1	-0.688	-1.61	1.96E-08	7.17E-06
217109_at	MUC4	-0.689	-1.61	4.21E-03	4.45E-02
213967_at	RALYL	-0.689	-1.61	7.16E-05	2.66E-03
214417_s_at	FETUB	-0.69	-1.61	3.27E-03	3.78E-02
1555151_s_at	TDH	-0.692	-1.62	8.07E-07	1.03E-04
235022_at	FAM210A	-0.693	-1.62	2.49E-07	4.46E-05
214168_s_at	TJP1	-0.693	-1.62	1.12E-05	6.83E-04
206426_at	MLANA	-0.695	-1.62	7.25E-06	4.97E-04
1553691_at	B3GALNT2	-0.696	-1.62	6.45E-08	1.67E-05
222783_s_at	SMOC1	-0.696	-1.62	5.93E-04	1.20E-02
1556037_s_at	HHIP	-0.697	-1.62	4.93E-05	2.02E-03
212983_at	HRAS	-0.698	-1.62	2.46E-07	4.46E-05
229942_at	BNC2	-0.698	-1.62	6.55E-05	2.50E-03
211362_s_at	SERPINB13	-0.698	-1.62	2.55E-07	4.47E-05
232568_at	MGC24103	-0.699	-1.62	9.99E-05	3.40E-03
226681_at	UBE2H	-0.7	-1.62	1.23E-06	1.39E-04
229228_at	CREB5 /// LOC401317	-0.702	-1.63	3.47E-04	8.27E-03
206630_at	TYR	-0.703	-1.63	7.46E-05	2.75E-03
231875_at	KIF21A	-0.703	-1.63	1.93E-06	1.88E-04
204730_at	RIMS3	-0.704	-1.63	3.00E-09	1.77E-06
216629_at	SRRM2	-0.705	-1.63	9.82E-06	6.22E-04
221803_s_at	NRBF2	-0.706	-1.63	9.37E-08	2.11E-05
227961_at	CTSB	-0.707	-1.63	4.98E-03	4.96E-02
243611_at	MICALCL	-0.708	-1.63	6.47E-07	8.99E-05
223103_at	STARD10	-0.709	-1.63	5.07E-07	7.62E-05
209679_s_at	SMAGP	-0.709	-1.63	1.11E-07	2.38E-05
1554524_a_at	OLFM3	-0.716	-1.64	1.71E-04	5.02E-03
222774_s_at	NETO2	-0.717	-1.64	7.24E-05	2.68E-03
212915_at	PDZRN3	-0.717	-1.64	4.11E-04	9.37E-03
228303_at	GALNT6	-0.718	-1.64	6.05E-06	4.39E-04
219529_at	CLIC3	-0.718	-1.64	1.67E-05	9.23E-04
220782_x_at	KLK12	-0.718	-1.64	3.15E-03	3.68E-02
213713_s_at	GLB1L2	-0.718	-1.65	1.06E-06	1.27E-04
226829_at	AFAP1L2	-0.719	-1.65	8.77E-11	1.67E-07

226187_at	CDS1	-0.72	-1.65	8.17E-10	7.55E-07
216604_s_at	SLC7A8	-0.72	-1.65	9.57E-04	1.65E-02
210276_s_at	TRIOBP	-0.722	-1.65	7.56E-08	1.83E-05
224724_at	SULF2	-0.722	-1.65	4.09E-07	6.37E-05
228720_at	SORCS2	-0.723	-1.65	6.87E-04	1.32E-02
233555_s_at	SULF2	-0.724	-1.65	6.61E-05	2.51E-03
217294_s_at	ENO1	-0.724	-1.65	1.78E-03	2.52E-02
220203_at	BMP8A	-0.728	-1.66	9.10E-09	3.94E-06
202147_s_at	IFRD1	-0.729	-1.66	3.21E-07	5.32E-05
226623_at	PHYHIPL	-0.731	-1.66	1.75E-05	9.51E-04
232914_s_at	SYTL2	-0.731	-1.66	2.75E-05	1.34E-03
237981_at	CMYA5	-0.731	-1.66	1.03E-04	3.47E-03
205709_s_at	CDS1	-0.733	-1.66	8.52E-06	5.65E-04
201267_s_at	PSMC3	-0.734	-1.66	2.64E-03	3.27E-02
223599_at	TRIM6	-0.734	-1.66	7.10E-04	1.35E-02
213793_s_at	HOMER1	-0.736	-1.67	1.65E-04	4.87E-03
1552496_a_at	COBL	-0.737	-1.67	4.66E-05	1.95E-03
241990_at	RHOV	-0.737	-1.67	1.64E-06	1.69E-04
217185_s_at	ZNF259 /// ZNF259P1	-0.741	-1.67	2.02E-07	3.85E-05
225496_s_at	SYTL2	-0.745	-1.68	6.89E-05	2.59E-03
203742_s_at	LOC732360 /// TDG	-0.746	-1.68	7.59E-08	1.83E-05
232067_at	FAXC	-0.747	-1.68	3.07E-03	3.62E-02
1552626_a_at	TMEM163	-0.752	-1.68	2.07E-03	2.79E-02
210948_s_at	LEF1	-0.752	-1.68	2.15E-06	2.05E-04
228900_at	SPECC1	-0.753	-1.69	4.38E-06	3.46E-04
237466_s_at	HHIP	-0.76	-1.69	2.10E-06	2.02E-04
220272_at	BNC2	-0.76	-1.69	5.45E-04	1.13E-02
222874_s_at	CLN8	-0.76	-1.69	3.41E-09	1.94E-06
235275_at	BMP8B	-0.76	-1.69	1.23E-05	7.31E-04
211538_s_at	HSPA2	-0.761	-1.69	2.89E-07	4.88E-05
211764_s_at	UBE2D1	-0.763	-1.7	3.91E-08	1.16E-05
208399_s_at	EDN3	-0.765	-1.7	1.71E-03	2.45E-02
214104_at	GPR161	-0.765	-1.7	2.61E-07	4.53E-05
228605_at	UBXN2A	-0.768	-1.7	5.09E-04	1.08E-02
200052_s_at	ILF2	-0.771	-1.71	3.36E-05	1.53E-03
214939_x_at	MLLT4	-0.773	-1.71	1.41E-10	2.35E-07
209273_s_at	ISCA1	-0.774	-1.71	1.90E-08	7.01E-06
203423_at	RBP1	-0.778	-1.71	2.60E-04	6.68E-03
215150_at	YOD1	-0.778	-1.72	1.08E-05	6.68E-04
213836_s_at	WIPI1	-0.779	-1.72	1.44E-07	2.99E-05
239136_at	UNC5B-AS1	-0.779	-1.72	1.55E-03	2.29E-02
34478_at	RAB11B	-0.779	-1.72	1.43E-03	2.16E-02

213457_at	MFHAS1	-0.78	-1.72	4.24E-04	9.59E-03
223879_s_at	OXR1	-0.781	-1.72	2.67E-05	1.31E-03
205117_at	FGF1	-0.783	-1.72	4.66E-03	4.76E-02
216641_s_at	LAD1	-0.784	-1.72	5.47E-05	2.18E-03
205422_s_at	ITGBL1	-0.786	-1.72	1.40E-05	8.08E-04
233971_at	FAM166A	-0.787	-1.73	1.30E-04	4.10E-03
209848_s_at	PMEL	-0.796	-1.74	1.56E-05	8.76E-04
228653_at	SAMD5	-0.798	-1.74	1.36E-03	2.09E-02
232555_at	CREB5	-0.798	-1.74	2.59E-04	6.66E-03
212822_at	HEG1	-0.799	-1.74	6.64E-05	2.52E-03
223078_s_at	TMOD3	-0.8	-1.74	8.74E-08	1.99E-05
208555_x_at	CST2	-0.801	-1.74	3.82E-09	2.02E-06
212807_s_at	SORT1	-0.801	-1.74	1.52E-07	3.10E-05
218384_at	CARHSP1	-0.806	-1.75	3.32E-05	1.52E-03
219612_s_at	FGG	-0.808	-1.75	8.16E-04	1.48E-02
235095_at	CCDC64B	-0.808	-1.75	8.16E-08	1.93E-05
231192_at	LPAR3	-0.809	-1.75	2.00E-12	2.46E-08
207195_at	CNTN6	-0.81	-1.75	1.82E-03	2.56E-02
213486_at	COPG2IT1	-0.81	-1.75	4.88E-04	1.05E-02
219367_s_at	NRP2	-0.812	-1.76	1.01E-04	3.42E-03
205931_s_at	CREB5 /// LOC401317	-0.812	-1.76	2.66E-04	6.74E-03
1553915_at	C10orf126	-0.813	-1.76	5.64E-06	4.19E-04
233586_s_at	KLK12	-0.813	-1.76	1.89E-03	2.63E-02
206912_at	FOXE1	-0.814	-1.76	1.76E-04	5.09E-03
206504_at	CYP24A1	-0.814	-1.76	2.25E-04	6.03E-03
203222_s_at	TLE1	-0.814	-1.76	3.26E-06	2.81E-04
217799_x_at	UBE2H	-0.814	-1.76	2.26E-06	2.14E-04
226237_at	COL8A1	-0.815	-1.76	3.89E-03	4.23E-02
209242_at	PEG3	-0.815	-1.76	1.38E-04	4.32E-03
239082_at	FZD3	-0.815	-1.76	2.80E-04	7.00E-03
239707_at	SLC5A10	-0.819	-1.76	9.01E-11	1.67E-07
216092_s_at	SLC7A8	-0.821	-1.77	9.77E-06	6.22E-04
227955_s_at	EFNA5	-0.821	-1.77	6.48E-08	1.67E-05
219369_s_at	OTUB2	-0.822	-1.77	1.12E-06	1.32E-04
1552685_a_at	GRHL1	-0.823	-1.77	3.87E-06	3.17E-04
239913_at	SLC10A4	-0.824	-1.77	3.57E-06	2.99E-04
234316_x_at	KLK12	-0.826	-1.77	1.58E-04	4.73E-03
1568617_a_at	CAMSAP3	-0.828	-1.78	5.31E-06	4.01E-04
1554334_a_at	DNAJA4	-0.832	-1.78	2.95E-09	1.77E-06
226698_at	FCHSD1	-0.834	-1.78	7.13E-08	1.79E-05
201147_s_at	TIMP3	-0.835	-1.78	6.19E-05	2.39E-03
213759_at	ARL4C	-0.837	-1.79	1.88E-08	7.01E-06

210387_at	NCALD	-0.837	-1.79	7.99E-06	5.40E-04
235723_at	BNC2	-0.838	-1.79	3.60E-04	8.51E-03
202883_s_at	PPP2R1B	-0.84	-1.79	1.54E-08	6.07E-06
209387_s_at	TM4SF1	-0.843	-1.79	7.15E-05	2.66E-03
205471_s_at	DACH1	-0.846	-1.8	3.45E-04	8.23E-03
210521_s_at	FETUB	-0.846	-1.8	5.42E-04	1.13E-02
219250_s_at	FLRT3	-0.848	-1.8	3.38E-08	1.07E-05
220245_at	SLC45A2	-0.861	-1.82	8.86E-07	1.10E-04
228910_at	CD82	-0.862	-1.82	8.18E-07	1.04E-04
1552511_a_at	CPA6	-0.862	-1.82	1.77E-07	3.54E-05
215034_s_at	TM4SF1	-0.865	-1.82	6.16E-05	2.38E-03
220999_s_at	CYFIP2	-0.865	-1.82	1.45E-05	8.24E-04
201149_s_at	TIMP3	-0.87	-1.83	4.07E-04	9.31E-03
223650_s_at	NRBF2	-0.871	-1.83	1.85E-08	6.99E-06
1553710_at	FAM218A	-0.871	-1.83	2.04E-04	5.66E-03
241985_at	JMY	-0.872	-1.83	3.06E-07	5.14E-05
1555299_s_at	ERVW-1	-0.872	-1.83	9.21E-07	1.13E-04
205637_s_at	SH3GL3	-0.873	-1.83	9.95E-06	6.29E-04
203413_at	NELL2	-0.874	-1.83	9.40E-04	1.63E-02
228922_at	SHF	-0.877	-1.84	5.54E-06	4.14E-04
227410_at	FAM43A	-0.878	-1.84	1.23E-09	9.83E-07
221558_s_at	LEF1	-0.879	-1.84	1.35E-06	1.49E-04
229856_s_at	PITHD1	-0.88	-1.84	4.67E-07	7.16E-05
241412_at	BTC	-0.882	-1.84	4.20E-05	1.80E-03
220129_at	CCDC169-SOHLH2 /// SOHLH2	-0.883	-1.84	1.75E-04	5.07E-03
221102_s_at	TRPM6	-0.884	-1.85	6.50E-07	9.00E-05
203367_at	DUSP14	-0.884	-1.85	5.05E-10	6.18E-07
210372_s_at	TPD52L1	-0.885	-1.85	7.52E-06	5.14E-04
220038_at	C8orf44-SGK3 /// SGK3	-0.887	-1.85	1.14E-03	1.85E-02
219842_at	ARL15	-0.887	-1.85	4.07E-06	3.30E-04
235479_at	CPEB2	-0.894	-1.86	7.55E-07	1.00E-04
220625_s_at	ELF5	-0.895	-1.86	3.18E-05	1.47E-03
226122_at	PLEKHG1	-0.895	-1.86	3.87E-11	1.02E-07
201043_s_at	ANP32A	-0.899	-1.86	1.20E-05	7.17E-04
202016_at	MEST	-0.9	-1.87	4.61E-04	1.01E-02
232149_s_at	NSMAF	-0.9	-1.87	5.13E-07	7.67E-05
242329_at	CREB5 /// LOC401317	-0.903	-1.87	5.56E-05	2.21E-03
216504_s_at	SLC39A8	-0.904	-1.87	9.54E-04	1.65E-02
204378_at	BCAS1	-0.904	-1.87	3.14E-06	2.75E-04
230135_at	HHIP	-0.904	-1.87	1.37E-04	4.30E-03
225295_at	SLC39A10	-0.906	-1.87	3.59E-08	1.09E-05
232378_at	SLC5A9	-0.918	-1.89	1.06E-03	1.77E-02

219301_s_at	CNTNAP2	-0.924	-1.9	2.04E-04	5.65E-03
205535_s_at	PCDH7	-0.925	-1.9	2.53E-06	2.35E-04
205125_at	PLCD1	-0.929	-1.9	1.12E-09	9.37E-07
219683_at	FZD3	-0.929	-1.9	6.67E-05	2.53E-03
206004_at	TGM3	-0.93	-1.9	1.87E-06	1.85E-04
220800_s_at	TMOD3	-0.932	-1.91	2.35E-08	8.21E-06
202752_x_at	SLC7A8	-0.932	-1.91	5.24E-06	3.98E-04
205534_at	PCDH7	-0.933	-1.91	4.72E-05	1.96E-03
1556421_at	LOC286189	-0.936	-1.91	1.86E-04	5.30E-03
1553539_at	KRT74	-0.938	-1.92	3.66E-05	1.63E-03
237732_at	PRR9	-0.938	-1.92	4.22E-03	4.46E-02
200744_s_at	GNB1	-0.942	-1.92	6.11E-10	6.27E-07
230722_at	BNC2	-0.944	-1.92	1.00E-04	3.41E-03
210335_at	RASSF9	-0.947	-1.93	1.10E-05	6.76E-04
1555462_at	PPP1R1C	-0.947	-1.93	2.18E-04	5.92E-03
217924_at	C6orf106	-0.949	-1.93	1.36E-06	1.51E-04
224277_at	MOP-1	-0.95	-1.93	6.08E-09	2.92E-06
231461_at	KRT71	-0.955	-1.94	4.97E-03	4.96E-02
1557122_s_at	GABRB2	-0.959	-1.94	1.68E-04	4.95E-03
205592_at	SLC4A1	-0.96	-1.95	2.46E-08	8.42E-06
213909_at	LRRC15	-0.961	-1.95	2.04E-04	5.65E-03
1553493_a_at	TDH	-0.966	-1.95	1.17E-06	1.35E-04
206774_at	FRMPD1	-0.971	-1.96	2.84E-07	4.81E-05
242344_at	GABRB2	-0.976	-1.97	1.74E-04	5.07E-03
205348_s_at	DYNC1I1	-0.976	-1.97	4.25E-04	9.59E-03
206498_at	OCA2	-0.978	-1.97	9.33E-06	6.02E-04
204790_at	SMAD7	-0.979	-1.97	3.57E-08	1.09E-05
205941_s_at	COL10A1	-0.98	-1.97	2.33E-07	4.28E-05
1569433_at	SAMD5	-0.983	-1.98	9.28E-05	3.23E-03
214433_s_at	SELENBP1	-0.983	-1.98	1.40E-05	8.10E-04
236255_at	PLEKHG4B	-0.984	-1.98	1.39E-05	8.07E-04
222015_at	CSNK1E	-0.985	-1.98	5.73E-09	2.78E-06
228068_at	GOLGA7B	-0.987	-1.98	4.94E-09	2.50E-06
1554333_at	DNAJA4	-0.988	-1.98	8.62E-08	1.99E-05
224520_s_at	BEST3	-0.989	-1.98	9.40E-05	3.26E-03
216258_s_at	SERPINB13	-1.01	-2.01	2.16E-07	4.03E-05
233340_at	SPINK13	-1.01	-2.02	4.77E-05	1.97E-03
205334_at	S100A1	-1.02	-2.02	3.41E-05	1.54E-03
209372_x_at	TUBB2A /// TUBB2B	-1.02	-2.03	7.06E-07	9.53E-05
221389_at	PLA2G2E	-1.03	-2.04	1.69E-11	6.44E-08
203827_at	WIPI1	-1.04	-2.05	6.44E-10	6.43E-07
208708_x_at	EIF5	-1.04	-2.06	6.67E-11	1.45E-07

227950_at	UBE2H	-1.04	-2.06	2.56E-08	8.59E-06
206777_s_at	CRYBB2 /// CRYBB2P1	-1.04	-2.06	3.19E-03	3.71E-02
212479_s_at	RMND5A	-1.05	-2.07	2.03E-09	1.36E-06
243478_at	CHST2	-1.05	-2.07	3.05E-05	1.43E-03
239028_at	LYPD6	-1.05	-2.08	4.80E-08	1.36E-05
219768_at	VTCN1	-1.05	-2.08	1.74E-03	2.48E-02
217395_at	MT4	-1.06	-2.08	2.60E-07	4.53E-05
203650_at	PROCR	-1.06	-2.08	3.32E-10	4.54E-07
228996_at	RC3H1	-1.06	-2.09	5.63E-08	1.53E-05
236263_at	SHH	-1.06	-2.09	1.23E-04	3.95E-03
217933_s_at	LAP3	-1.07	-2.1	1.30E-09	9.98E-07
209617_s_at	CTNND2	-1.08	-2.11	1.38E-04	4.32E-03
218458_at	GMCL1	-1.08	-2.12	6.44E-09	3.05E-06
240389_at	TRPM6	-1.08	-2.12	2.64E-04	6.73E-03
214590_s_at	UBE2D1	-1.09	-2.13	4.72E-10	6.01E-07
230493_at	SHISA2	-1.09	-2.14	2.30E-03	2.99E-02
215779_s_at	HIST1H2BC /// HIST1H2BE /// HIST1H2BF /// HIST1H2BG /// HIST1H2BI /// NCALD	-1.1	-2.14	4.26E-06	3.39E-04
231743_at	WNT3	-1.1	-2.14	2.92E-11	9.00E-08
225809_at	PARM1	-1.1	-2.14	1.32E-05	7.76E-04
230740_at	EHD3	-1.1	-2.14	5.32E-05	2.14E-03
213385_at	CHN2	-1.1	-2.14	2.34E-07	4.28E-05
230138_at	NCS1	-1.12	-2.17	3.89E-08	1.16E-05
238478_at	BNC2	-1.12	-2.17	2.97E-05	1.41E-03
219554_at	RHCG	-1.12	-2.18	1.69E-06	1.72E-04
214226_at	PRSS53	-1.13	-2.19	2.43E-09	1.55E-06
223427_s_at	EPB41L4B	-1.14	-2.21	1.64E-07	3.33E-05
210689_at	CLDN14	-1.14	-2.21	1.48E-06	1.59E-04
229030_at	CAPN8	-1.15	-2.22	9.78E-04	1.67E-02
1554097_a_at	MIR31HG	-1.15	-2.23	5.46E-10	6.26E-07
214636_at	CALCB	-1.15	-2.23	1.00E-07	2.23E-05
221425_s_at	ISCA1	-1.16	-2.23	8.01E-11	1.64E-07
239203_at	LSMEM1	-1.16	-2.24	1.01E-05	6.34E-04
222871_at	KLHDC8A	-1.16	-2.24	5.72E-07	8.35E-05
233814_at	EFNA5	-1.17	-2.25	3.30E-07	5.43E-05
215785_s_at	CYFIP2	-1.17	-2.25	1.07E-07	2.35E-05
224412_s_at	TRPM6	-1.17	-2.25	1.58E-04	4.74E-03
235683_at	SESN3	-1.18	-2.26	3.61E-07	5.75E-05
218980_at	FHOD3	-1.18	-2.26	1.69E-05	9.27E-04
230469_at	RTKN2	-1.18	-2.26	3.14E-05	1.46E-03
208216_at	DLX4	-1.18	-2.27	2.53E-06	2.35E-04
202376_at	SERPINA3	-1.18	-2.27	4.49E-05	1.90E-03

212478_at	RMND5A	-1.19	-2.28	9.87E-12	5.21E-08
209800_at	KRT16	-1.19	-2.28	6.87E-06	4.78E-04
1552807_a_at	SIGLEC10	-1.2	-2.29	1.43E-07	2.98E-05
1556533_at	LINC00868	-1.2	-2.3	6.02E-11	1.43E-07
234488_s_at	GMCL1 /// GMCL1P1	-1.21	-2.31	9.13E-09	3.94E-06
235591_at	SSTR1	-1.22	-2.32	7.02E-06	4.85E-04
203921_at	CHST2	-1.22	-2.33	2.36E-05	1.20E-03
229163_at	CAMK2N1	-1.22	-2.33	1.12E-04	3.69E-03
221416_at	PLA2G2F	-1.22	-2.33	5.65E-10	6.26E-07
206376_at	SLC6A15	-1.23	-2.35	1.46E-10	2.35E-07
206882_at	SLC1A6	-1.23	-2.35	3.54E-07	5.67E-05
200790_at	ODC1	-1.24	-2.37	6.19E-11	1.43E-07
217031_at	KRT84	-1.26	-2.4	2.39E-05	1.21E-03
242138_at	DLX1	-1.26	-2.4	2.85E-05	1.37E-03
226887_at	HSPA14	-1.26	-2.4	9.54E-12	5.21E-08
209343_at	EFHD1	-1.27	-2.42	3.91E-04	9.06E-03
217678_at	SLC7A11	-1.27	-2.42	6.34E-06	4.55E-04
227763_at	LYPD6	-1.27	-2.42	9.37E-05	3.26E-03
220984_s_at	SLCO5A1	-1.29	-2.44	3.38E-07	5.53E-05
242062_at	SAMD8	-1.29	-2.45	2.79E-08	9.30E-06
230276_at	FAM49A	-1.29	-2.45	1.76E-07	3.54E-05
233123_at	SLC40A1	-1.3	-2.45	9.14E-07	1.13E-04
207092_at	LEP	-1.3	-2.46	3.85E-03	4.20E-02
206604_at	OVOL1	-1.3	-2.47	9.98E-10	9.00E-07
231778_at	DLX3	-1.31	-2.47	1.95E-11	6.53E-08
243445_at	BNC2	-1.31	-2.48	1.13E-06	1.33E-04
1553574_at	IFNE	-1.31	-2.48	4.27E-08	1.24E-05
219932_at	SLC27A6	-1.32	-2.49	7.20E-06	4.94E-04
1569852_at	LSMEM1	-1.34	-2.53	4.68E-09	2.42E-06
205290_s_at	BMP2	-1.34	-2.53	7.33E-09	3.39E-06
219463_at	LAMP5	-1.35	-2.54	2.43E-08	8.38E-06
205724_at	PKP1	-1.35	-2.55	6.78E-10	6.59E-07
221962_s_at	UBE2H	-1.36	-2.56	1.10E-07	2.37E-05
1555774_at	ZAR1	-1.36	-2.57	1.07E-09	9.25E-07
238568_s_at	NPC1	-1.36	-2.57	3.52E-09	1.94E-06
1553388_at	FAM26D	-1.37	-2.58	1.35E-07	2.83E-05
231583_at	KRT74	-1.37	-2.58	2.91E-03	3.48E-02
240388_at	KRT27	-1.38	-2.6	8.90E-04	1.57E-02
1558687_a_at	FOXN1	-1.38	-2.6	3.36E-12	3.00E-08
227506_at	SLC16A9	-1.39	-2.62	1.76E-08	6.76E-06
228646_at	PPP1R1C	-1.39	-2.62	1.12E-06	1.32E-04
227650_at	HSPA14	-1.39	-2.62	5.54E-08	1.53E-05

1553829_at	CYP1B1-AS1	-1.4	-2.63	1.13E-05	6.86E-04
210082_at	ABCA4	-1.4	-2.64	8.67E-08	1.99E-05
208290_s_at	EIF5	-1.41	-2.66	3.80E-11	1.02E-07
1554592_a_a t	SLC1A6	-1.41	-2.66	5.34E-08	1.48E-05
203130_s_at	KIF5C	-1.43	-2.69	1.20E-05	7.17E-04
205289_at	BMP2	-1.44	-2.71	5.98E-07	8.63E-05
241014_at	FLG-AS1	-1.44	-2.71	7.30E-08	1.80E-05
1553861_at	TCP11L2	-1.45	-2.73	5.93E-10	6.26E-07
204687_at	PARM1	-1.45	-2.74	2.39E-07	4.36E-05
223739_at	PADI1	-1.46	-2.75	3.15E-07	5.26E-05
207065_at	KRT75	-1.46	-2.75	1.37E-05	7.99E-04
214576_at	KRT36	-1.47	-2.77	6.53E-09	3.05E-06
202884_s_at	PPP2R1B	-1.47	-2.77	3.52E-08	1.08E-05
209757_s_at	MYCN	-1.48	-2.78	5.20E-06	3.95E-04
209859_at	TRIM9	-1.48	-2.78	1.12E-05	6.83E-04
202886_s_at	PPP2R1B	-1.48	-2.79	1.38E-08	5.50E-06
1553494_at	TDH	-1.48	-2.8	2.11E-06	2.03E-04
208250_s_at	DMBT1	-1.48	-2.8	1.11E-05	6.82E-04
228975_at	SP6	-1.49	-2.81	1.14E-12	2.10E-08
211357_s_at	ALDOB	-1.49	-2.81	5.89E-04	1.19E-02
223720_at	SPINK7	-1.49	-2.82	4.33E-05	1.85E-03
237003_at	BEST3	-1.5	-2.82	3.24E-07	5.35E-05
1554593_s_a t	SLC1A6	-1.5	-2.83	1.41E-06	1.54E-04
1557321_a_a t	CAPN14	-1.5	-2.84	1.19E-06	1.36E-04
213107_at	TNIK	-1.51	-2.84	5.14E-08	1.44E-05
221627_at	TRIM10	-1.51	-2.84	6.82E-07	9.30E-05
205374_at	SLN	-1.51	-2.85	2.13E-06	2.03E-04
213774_s_at	PPP1R2	-1.51	-2.85	2.39E-10	3.39E-07
220624_s_at	ELF5	-1.52	-2.86	1.28E-10	2.26E-07
229158_at	WNK4	-1.52	-2.86	2.10E-04	5.78E-03
217428_s_at	COL10A1	-1.54	-2.9	7.19E-08	1.79E-05
238625_at	C1orf168	-1.55	-2.92	7.95E-08	1.91E-05
232682_at	MREG	-1.56	-2.94	1.25E-09	9.83E-07
209683_at	FAM49A	-1.57	-2.96	9.36E-09	3.97E-06
229823_at	RIMS2	-1.57	-2.96	6.81E-06	4.75E-04
233631_x_at	KRTAP9-2 /// KRTAP9-8 /// LOC100996762	-1.57	-2.97	6.82E-04	1.32E-02
218935_at	EHD3	-1.6	-3.03	2.50E-07	4.46E-05
239572_at	GJA3	-1.62	-3.08	5.57E-08	1.53E-05
202833_s_at	SERPINA1	-1.62	-3.08	2.37E-04	6.26E-03
234685_x_at	KRTAP4-9	-1.65	-3.14	3.27E-04	7.94E-03
210311_at	FGF5	-1.66	-3.17	6.29E-08	1.66E-05
211485_s_at	FGF18	-1.67	-3.19	3.43E-08	1.07E-05

233301_at	OXCT2	-1.68	-3.2	3.58E-10	4.73E-07
211429_s_at	SERPINA1	-1.7	-3.26	1.62E-05	9.01E-04
207716_at	KRT38	-1.71	-3.27	8.38E-04	1.51E-02
219659_at	ATP8A2	-1.75	-3.37	3.08E-06	2.71E-04
227764_at	LYPD6	-1.78	-3.42	3.04E-06	2.69E-04
210319_x_at	MSX2	-1.79	-3.46	8.56E-08	1.99E-05
218266_s_at	NCS1	-1.8	-3.49	5.18E-10	6.18E-07
205555_s_at	MSX2	-1.84	-3.57	4.72E-09	2.42E-06
219832_s_at	HOXC13	-1.84	-3.59	2.06E-10	3.04E-07
209981_at	CSDC2	-1.87	-3.66	1.84E-09	1.26E-06
233681_at	KRTAP3-3	-1.87	-3.67	1.05E-04	3.50E-03
209921_at	SLC7A11	-1.89	-3.7	5.15E-08	1.44E-05
1564921_at	KRTAP13-1	-1.92	-3.79	2.62E-03	3.26E-02
1564435_a_at	KRT72	-1.92	-3.79	6.18E-06	4.46E-04
237507_at	KRT73	-1.95	-3.87	1.67E-05	9.23E-04
206987_x_at	FGF18	-1.98	-3.94	3.21E-07	5.32E-05
202166_s_at	PPP1R2	-1.98	-3.95	1.93E-13	7.14E-09
215189_at	KRT86 /// LOC100509764	-1.99	-3.97	3.15E-06	2.75E-04
228705_at	CAPN12	-1.99	-3.98	3.60E-09	1.96E-06
216810_at	KRTAP4-7	-2	-4	7.67E-05	2.81E-03
211029_x_at	FGF18	-2.01	-4.02	2.05E-07	3.87E-05
229730_at	SMTNL2	-2.01	-4.02	5.15E-06	3.93E-04
233534_at	KRTAP3-2	-2.02	-4.04	2.55E-04	6.60E-03
206423_at	ANGPTL7	-2.03	-4.07	3.69E-04	8.64E-03
222351_at	PPP2R1B	-2.04	-4.1	6.73E-08	1.70E-05
220090_at	CRNN	-2.04	-4.1	1.19E-06	1.36E-04
207147_at	DLX2	-2.07	-4.19	1.91E-06	1.87E-04
224262_at	IL1F10	-2.07	-4.21	5.88E-08	1.56E-05
1562628_at	KRT40	-2.1	-4.28	7.78E-07	1.02E-04
1556410_a_at	KRTAP19-1	-2.11	-4.33	7.86E-05	2.86E-03
234772_s_at	KRTAP2-1 /// KRTAP2-2	-2.12	-4.35	1.50E-04	4.56E-03
1553537_at	KRT73	-2.13	-4.37	1.26E-08	5.06E-06
233533_at	KRTAP1-5	-2.17	-4.5	2.60E-04	6.68E-03
220978_at	KRTAP1-3	-2.17	-4.5	1.08E-04	3.56E-03
240967_at	KRTAP19-3	-2.17	-4.5	4.25E-04	9.60E-03
203304_at	BAMBI	-2.2	-4.6	1.85E-07	3.64E-05
234635_at	KRTAP4-1	-2.21	-4.62	1.61E-04	4.80E-03
220635_at	PSORS1C2	-2.21	-4.62	1.15E-09	9.44E-07
214284_s_at	FGF18	-2.21	-4.63	1.15E-06	1.34E-04
220970_s_at	KRTAP2-3 /// KRTAP2-4	-2.22	-4.65	9.89E-05	3.39E-03
205713_s_at	COMP	-2.23	-4.68	4.88E-06	3.79E-04
204720_s_at	DNAJC6	-2.23	-4.69	2.63E-09	1.63E-06

234639_x_at	KRTAP9-8	-2.23	-4.71	4.76E-05	1.97E-03
233640_x_at	KRTAP9-4	-2.25	-4.74	5.06E-05	2.06E-03
234683_at	KRTAP4-11 /// KRTAP4-6 /// LOC100134267	-2.25	-4.75	2.56E-04	6.62E-03
231382_at	FGF18	-2.27	-4.84	3.61E-06	3.00E-04
242301_at	CBLN2	-2.28	-4.84	4.29E-04	9.64E-03
206969_at	KRT34 /// LOC100653049	-2.33	-5.04	2.57E-05	1.28E-03
220976_s_at	KRTAP1-1	-2.39	-5.23	7.41E-05	2.73E-03
233537_at	KRTAP3-1	-2.43	-5.37	1.60E-04	4.76E-03
1555775_a_at	ZAR1	-2.44	-5.42	1.77E-09	1.26E-06
224269_at	KRTAP4-12	-2.44	-5.42	7.21E-05	2.67E-03
206027_at	S100A3	-2.46	-5.51	2.49E-08	8.44E-06
232887_at	PIRT	-2.5	-5.65	3.42E-09	1.94E-06
220256_s_at	OXCT2	-2.51	-5.68	4.06E-12	3.00E-08
220779_at	PADI3	-2.54	-5.82	3.34E-06	2.84E-04
1554398_at	LYG2	-2.56	-5.9	9.01E-09	3.94E-06
234678_at	KRTAP4-3	-2.57	-5.94	8.75E-06	5.75E-04
234680_at	KRTAP17-1	-2.58	-5.99	8.48E-06	5.65E-04
208092_s_at	FAM49A	-2.6	-6.05	2.08E-09	1.36E-06
234633_at	KRTAP4-11	-2.63	-6.18	1.85E-06	1.83E-04
234679_at	KRTAP9-3	-2.63	-6.21	1.43E-05	8.18E-04
206677_at	KRT31	-2.68	-6.4	8.16E-07	1.04E-04
206994_at	CST4	-2.69	-6.45	8.07E-10	7.55E-07
234691_at	KRTAP2-1	-2.75	-6.72	4.19E-06	3.35E-04
216921_s_at	KRT35	-2.76	-6.76	1.67E-05	9.23E-04
1562629_a_at	KRT40	-2.77	-6.82	3.21E-06	2.79E-04
230720_at	RNF182	-2.79	-6.94	8.92E-09	3.94E-06
234637_at	KRTAP4-5	-2.8	-6.96	2.50E-05	1.26E-03
207670_at	KRT85	-2.8	-6.98	3.44E-06	2.92E-04
214517_at	KRTAP5-9	-2.85	-7.19	8.63E-09	3.94E-06
207146_at	KRT32	-2.85	-7.23	5.61E-07	8.23E-05
220972_s_at	KRTAP9-9	-2.88	-7.37	2.06E-05	1.08E-03
234684_s_at	KRTAP4-4	-2.9	-7.45	8.04E-06	5.42E-04
234671_at	KRTAP4-2	-2.94	-7.67	3.24E-06	2.80E-04
1555673_at	KRTAP2-3 /// KRTAP2-4	-2.96	-7.81	1.13E-05	6.86E-04
234631_at	KRTAP4-8	-3.01	-8.03	6.69E-07	9.19E-05
1564803_at	KRTAP11-1	-3.04	-8.23	4.22E-06	3.37E-04
207787_at	KRT33B	-3.14	-8.83	5.08E-06	3.88E-04
1560897_a_at	KRTAP10-11	-3.2	-9.19	1.60E-06	1.68E-04
237853_x_at	KRTAP10-12	-3.26	-9.55	1.35E-09	1.01E-06
233158_at	KRT82	-3.28	-9.71	2.51E-07	4.46E-05
208532_x_at	KRTAP5-8	-3.49	-11.3	1.61E-08	6.25E-06
1564960_at	KRTAP7-1	-3.63	-12.4	6.37E-06	4.56E-04

213711_at	KRT81	-3.64	-12.5	1.05E-07	2.32E-05
1564974_at	KRTAP8-1	-3.71	-13.1	2.15E-05	1.11E-03
219270_at	CHAC1	-3.83	-14.2	1.92E-10	2.95E-07
206224_at	CST1	-3.92	-15.1	1.74E-11	6.44E-08
208483_x_at	KRT33A	-3.92	-15.2	8.15E-08	1.93E-05
234880_x_at	KRTAP1-3	-3.95	-15.4	3.27E-08	1.05E-05
207669_at	KRT83	-4.13	-17.5	3.32E-08	1.06E-05
207457_s_at	LY6G6D	-4.2	-18.4	2.64E-09	1.63E-06
1561330_at	DSG4	-4.21	-18.6	1.93E-07	3.76E-05
221297_at	GPRC5D	-4.27	-19.3	9.17E-09	3.94E-06

Supplemental Table 6. KEGG pathways upregulated in AAP.

Terms	Genes	Count	Fold Enrichment	PValue
hsa05332:Graft-versus-host disease	HLA-DQB1, PRF1, HLA-DRB1, LOC100133583, GZMB, HLA-DMB, HLA-DMA, HLA-DQA1, HLA-F, LOC100133678, CD86, HLA-DRB4, IL1B, HLA-DPA1, HLA-DOA, KLRC1, CD28, HLA-DRA	15	13.48	7.52E-13
hsa04940:Type I diabetes mellitus	HLA-DQB1, PRF1, HLA-DRB1, LOC100133583, GZMB, HLA-DMB, HLA-DMA, HLA-DQA1, HLA-F, LOC100133678, CD86, HLA-DRB4, IL1B, HLA-DPA1, IL12B, HLA-DOA, CD28, HLA-DRA	15	12.44	2.76E-12
hsa05330:Allograft rejection	HLA-DQB1, PRF1, HLA-DRB1, LOC100133583, GZMB, HLA-DMB, HLA-DMA, HLA-DQA1, HLA-F, LOC100133678, CD86, HLA-DRB4, HLA-DPA1, IL12B, HLA-DOA, CD28, HLA-DRA	14	13.72	4.26E-12
hsa04514:Cell adhesion molecules (CAMs)	HLA-DQB1, ITGAL, PTPRC, HLA-DRB1, CD8A, CD8B, NRXN3, LOC100133583, HLA-DMB, HLA-DMA, ITGAM, HLA-DQA1, HLA-F, LOC100133678, SIGLEC1, CD86, ICOS, HLA-DRB4, CD2, CD4, HLA-DPA1, HLA-DOA, SELPLG, CD28, HLA-DRA	22	5.56	1.53E-10
hsa04060:Cytokine-cytokine receptor interaction	IL1R2, CCR1, CXCL9, CCL8, CCL5, IL7R, CXCL11, CXCL10, CCL26, FLT3LG, LIF, CCL22, IL12RB1, IL10RA, IL1B, CSF2RB, CSF1R, IL2RB, IL18RAP, IL7, CCL18, CCL17, IFNAR2, CCL13, CCR7, CCR5, CCR2, IL12B, XCL1, IL22RA2	30	3.80	3.67E-10
hsa04640:Hematopoietic cell lineage	IL1R2, CD3D, CD8A, HLA-DRB1, CD8B, CD3E, IL7, CD1C, CD1B, IL7R, ITGAM, FLT3LG, CD2, HLA-DRB4, IL1B, CD4, CSF1R, HLA-DRA	17	6.55	2.92E-09

hsa04612:Antigen processing and presentation	HLA-DQB1, CIITA, CD8A, HLA-DRB1, CD8B, LOC100133583, HLA-DMB, HLA-DMA, HLA-DQA1, B2M, HLA-F, LOC100133678, HSPA6, HLA-DRB4, HSPA7, CD4, HLA-DPA1, HLA-DOA, KLRC1, HLA-DRA	16	6.63	8.19E-09
hsa05320:Autoimmune thyroid disease	HLA-DQB1, PRF1, HLA-DRB1, GZMB, LOC100133583, HLA-DMB, HLA-DMA, HLA-DQA1, HLA-F, LOC100133678, CD86, HLA-DRB4, HLA-DPA1, HLA-DOA, HLA-DRA, CD28	13	8.95	9.25E-09
hsa05340:Primary immunodeficiency	CIITA, PTPRC, CD8A, CD3D, CD8B, CD3E, ICOS, LCK, CD4, JAK3, IL7R	11	10.16	5.20E-08
hsa05416:Viral myocarditis	HLA-DQB1, ITGAL, PRF1, HLA-DRB1, LOC100133583, HLA-DMB, HLA-DMA, HLA-DQA1, HLA-F, LOC100133678, CD86, RAC2, HLA-DRB4, HLA-DPA1, HLA-DOA, CD28, HLA-DRA	14	6.86	6.36E-08
hsa04062:Chemokine signaling pathway	ITK, FGR, CCR1, CXCL9, CCL8, STAT1, CCL5, CXCL11, CCL18, CCL17, CCL26, CXCL10, PRKCB, CCL22, CCL13, CCR7, CCR5, RAC2, CCR2, PIK3R5, JAK3, XCL1	22	3.91	1.04E-07
hsa05310:Asthma	FCER1A, HLA-DQB1, LOC100133678, HLA-DRB1, HLA-DRB4, LOC100133583, HLA-DPA1, HLA-DMB, HLA-DOA, HLA-DMA, HLA-DQA1, HLA-DRA	9	10.78	8.83E-07
hsa05322:Systemic lupus erythematosus	HLA-DQB1, HLA-DRB1, GRIN2A, LOC100133583, HLA-DMB, HLA-DMA, C1QC, HLA-DQA1, LOC100133678, C1QB, CD86, FCGR2B, FCGR2C, HLA-DRB4, HLA-DPA1, HLA-DOA, CD28, HLA-DRA	14	5.52	9.09E-07

hsa04672:Intestinal immune network for IgA production	HLA-DQB1, HLA-DRB1, LOC100133583, HLA-DMB, HLA-DMA, HLA-DQA1, LOC100133678, CD86, ICOS, HLA-DRB4, HLA-DPA1, HLA-DOA, CD28, HLA-DRA	11	7.57	1.07E-06
hsa04660:T cell receptor signaling pathway	PTPRC, ITK, CD3D, CD8A, CD3E, CD8B, PRKCQ, ICOS, LCK, PIK3R5, NFATC4, CD4, LCP2, CD28	14	4.23	1.90E-05
hsa04650:Natural killer cell mediated cytotoxicity	PRF1, ITGAL, MICB, KLRK1, GZMB, PRKCB, IFNAR2, RAC2, LCK, PIK3R5, NFATC4, KLRC1, LCP2, TYROBP	14	3.54	1.28E-04
hsa04620:Toll-like receptor signaling pathway	IFNAR2, CD86, TLR1, CXCL9, IL1B, PIK3R5, IL12B, CXCL11, STAT1, CCL5, CXCL10	11	3.59	8.20E-04
hsa04630:Jak-STAT signaling pathway	LIF, IFNAR2, IL2RB, IL12RB1, IL7, IL10RA, CSF2RB, PIK3R5, IL12B, JAK3, IL7R, STAT1, IL22RA2	13	2.77	0.002248905

Supplemental Table 7. KEGG pathways upregulated in AT/AU.

Terms	Genes	Count	Fold Enrichment	PValue
hsa04640:Hematopoietic cell lineage	IL1R2, TNF, HLA-DRB1, CD8A, CD8B, HLA-DRB3, CSF1, ANPEP, KIT, IL7R, ITGAM, FLT3LG, GP1BB, LOC652799, CD2, HLA-DRB4, MS4A1, IL1B, CD22, CD4, CSF1R, CR1, CD3G, CD3D, IL7, CD3E, CD1C, CD1B, CD1A, IL11RA, CD1E, CD33, EPOR, LOC653882, CD14, HLA-DRA	33	3.45	1.32E-10
hsa04514:Cell adhesion molecules (CAMs)	HLA-DQB1, ITGAL, CADM3, CADM1, HLA-DRB1, CD8A, CD8B, HLA-DRB3, LOC100133583, ITGB2, HLA-DMB, HLA-DMA, ITGAM, LOC100133678, PVRL3, ITGB7, ICOS, HLA-DRB4, CD2, CD22, CD4, HLA-DPB1, HLA-DOA, SELPLG, HLA-DOB, NEGR1, CD28, ICAM1, SELP, PTPRC, PTPRM, NRXN3, SELL, NLGN1, CD40, HLA-DQA1, HLA-F, SIGLEC1, CD86, ITGA8, CNTN2, HLA-DPA1, JAM2, HLA-DRA	41	2.81	7.29E-10
hsa05330:Allograft rejection	HLA-DQB1, PRF1, TNF, HLA-DRB1, HLA-DRB3, LOC100133583, GZMB, CD40, HLA-DMB, HLA-DMA, HLA-DQA1, HLA-F, LOC100133678, CD86, HLA-DRB4, HLA-DPA1, HLA-DPB1, IL12B, HLA-DOA, HLA-DOB, CD28, HLA-DRA	19	5.05	1.55E-09

hsa04062:Chemokine signaling pathway	ADCY1, ADCY2, ADCY5, PREX1, CXCL11, CXCL10, PLCB4, CCL3L1, CCL3L3, CXCR6, GNG2, PRKACB, PLCB1, SHC2, PLCB2, GNG7, SHC4, CCNL2, PRKCB, CCR7, CCR6, CX3CR1, CCR2, FGR, CCR1, CXCL9, ADRBK2, CCL8, CCL5, CCL4, CCL28, CCL27, CCL26, DOCK2, CCL22, CCL23, RAC2, SOS1, PIK3R5, ITK, HCK, CCL19, STAT1, CCL16, VAV1, CCL18, CCL17, CCL13, RAP1A, JAK2, JAK3, XCL1	50	2.41	2.66E-09
hsa04940:Type I diabetes mellitus	HLA-DQB1, PRF1, ICA1, TNF, HLA-DRB1, HLA-DRB3, LOC100133583, GZMB, HLA-DMB, HLA-DMA, HLA-DQA1, HLA-F, LOC100133678, CD86, HLA-DRB4, IL1B, HLA-DPA1, HLA-DPB1, IL12B, HLA-DOA, HLA-DOB, CD28, HLA-DRA	20	4.50	6.55E-09
hsa05332:Graft-versus-host disease	HLA-DQB1, PRF1, TNF, HLA-DRB1, HLA-DRB3, LOC100133583, GZMB, HLA-DMB, HLA-DMA, HLA-DQA1, HLA-F, LOC100133678, CD86, HLA-DRB4, IL1B, HLA-DPA1, HLA-DPB1, HLA-DOA, HLA-DOB, KLRC1, CD28, HLA-DRA	19	4.63	9.78E-09
hsa05310:Asthma	HLA-DQB1, FCER1A, TNF, HLA-DRB1, HLA-DRB3, PRG2, LOC100133583, CD40, HLA-DMB, HLA-DMA, HLA-DQA1, LOC100133678, HLA-DRB4, MS4A2, HLA-DPA1, HLA-DPB1, HLA-DOA, HLA-DOB, HLA-DRA	16	5.20	2.70E-08

hsa04060:Cytokine-cytokine receptor interaction	IL6ST, IL21R, TNFSF13, IL15, TNFSF12, CXCL11, CXCL10, FLT3LG, TNFRSF11A, CCL3L1, IL1RAP, CCL3L3, CXCR6, IL1B, LTB, IL18RAP, CD40, EDAR, IL11RA, CCNL2, IFNAR2, CCR7, CCR6, CX3CR1, CCR2, LOC653882, IL12B, TNFSF12-TNFSF13, IL1R2, TNF, TNFRSF25, CCR1, CSF1, CXCL9, CCL8, KIT, IL7R, CCL5, CCL28, CCL4, CCL27, CCL26, CCL22, TNFRSF1B, IL12RB1, CCL23, LOC652799, IL10RA, CD27, CSF1R, IL2RB, IL7, FLT4, CCL19, HGF, CCL16, CCL18, CCL17, TNFSF8, CCL13, EPOR, BMPR1B, XCL1, IL22RA2	58	2.00	1.93E-07
hsa04672:Intestinal immune network for IgA production	HLA-DQB1, HLA-DRB1, HLA-DRB3, TNFSF13, LOC100133583, TNFSF12, CD40, IL15, HLA-DMB, HLA-DMA, CCL28, CCL27, HLA-DQA1, LOC100133678, CD86, ICOS, ITGB7, HLA-DRB4, HLA-DPA1, HLA-DPB1, TNFSF12-TNFSF13, HLA-DOA, HLA-DOB, CD28, HLA-DRA	20	3.73	2.75E-07
hsa05340:Primary immunodeficiency	CIITA, PTPRC, CD3D, CD8A, CD8B, CD3E, CD40, IL7R, ADA, TAP2, ICOS, LCK, TAP1, ZAP70, CD4, JAK3	16	4.01	2.10E-06
hsa04612:Antigen processing and presentation	HLA-DQB1, CD8A, HLA-DRB1, CD8B, HLA-DRB3, IFI30, LOC100133583, NFYB, HLA-DMB, HLA-DMA, B2M, LOC100133678, TAP2, TAP1, HSPA6, HLA-DRB4, HSPA7, CD4, HLA-DPB1, HSPA5, HLA-DOA, HLA-DOB, CIITA, CTSS, HLA-DQA1, HLA-F, LOC400750, HLA-DPA1, KLRC1, HLA-DRA	25	2.81	2.76E-06

hsa05416:Viral myocarditis	HLA-DQB1, ITGAL, ICAM1, PRF1, HLA-DRB1, HLA-DRB3, LOC100133583, ITGB2, CD40, HLA-DMB, HLA-DMA, HLA-DQA1, HLA-F, LOC100133678, CD86, SGCG, RAC2, HLA-DRB4, MYH11, HLA-DPA1, HLA-DPB1, HLA-DOA, HLA-DOB, CD28, HLA-DRA	22	2.93	6.47E-06
hsa05320:Autoimmune thyroid disease	HLA-DQB1, TG, PRF1, HLA-DRB1, HLA-DRB3, LOC100133583, GZMB, CD40, HLA-DMB, HLA-DMA, HLA-DQA1, HLA-F, LOC100133678, CD86, HLA-DRB4, HLA-DPA1, HLA-DPB1, HLA-DOA, HLA-DOB, CD28, HLA-DRA	18	3.36	7.19E-06
hsa05322:Systemic lupus erythematosus	HLA-DQB1, TNF, HLA-DRB1, HIST1H2AG, C6, HLA-DRB3, LOC100133583, HLA-DMB, HLA-DMA, C1QC, LOC100133678, HLA-DRB4, HLA-DPB1, HLA-DOA, HLA-DOB, CD28, GRIN2A, CD40, H2AFJ, HLA-DQA1, C1QB, CD86, FCGR2B, FCGR2C, HIST1H2AI, HIST1H2AH, HIST1H2AK, HLA-DPA1, FCGR2A, HIST1H2AM, HIST1H2AL, HLA-DRA	23	2.46	7.72E-05
hsa04660:T cell receptor signaling pathway	PTPRC, ITK, TNF, CD3G, CD3D, CD8A, CD8B, CD3E, MALT1, VAV1, PAK6, LAT, PRKCQ, ICOS, SOS1, RASGRP1, LCK, ZAP70, PIK3R5, NFATC4, CD4, CD28, LCP2	23	1.89	0.003799116
hsa04650:Natural killer cell mediated cytotoxicity	PRF1, ITGAL, MICB, MICA, TNF, KLRK1, ITGB2, CD48, RAC2, SOS1, ZAP70, PIK3R5, NFATC4, SHC2, TYROBP, SHC4, ICAM1, GZMB, VAV1, PRKCB, IFNAR2, LAT, LCK, KLRC1, LCP2	25	1.71	0.008643092
hsa05414:Dilated cardiomyopathy	ADCY1, SLC8A1, ADCY2, TNF, ADCY5, CACNB1, ITGA10, TTN, TPM2, CACNA2D4, SGCG, ITGB7, ITGA8, PLN, ITGA7, RYR2, GNAS, PRKACB, CACNA1D	19	1.87	0.010033937

hsa04270:Vascular smooth muscle contraction	KCNMA1, KCNMB4, ADCY1, ADCY2, ADORA2A, ADCY5, MRVI1, ARHGEF12, KCNMB1, ITPR1, ITPR2, PRKCB, PRKCQ, PLCB4, MYH11, GNAS, PRKACB, PLCB1, PLA2G3, CACNA1D, PLCB2, PLA2G5	22	1.74	0.012265283
hsa04020:Calcium signaling pathway	ADCY1, ADCY2, ERBB4, ADORA2A, CAMK2G, TACR1, LHCGR, ATP2B2, PLCB4, PDE1A, CAMK2D, PLCD4, PRKACB, PLCB1, PLCB2, SLC8A1, PTGER3, GRIN2A, ITPR1, ITPR2, PRKCB, ADRB2, P2RX1, CHRM3, ATP2A3, PLN, CHRM1, CACNA1G, RYR2, GNAS, CACNA1D	31	1.55	0.01304301
hsa04912:GnRH signaling pathway	PLD1, ADCY1, ADCY2, ADCY5, CAMK2G, ITPR1, ITPR2, PRKCB, PLCB4, MAP3K3, SOS1, CAMK2D, GNAS, PRKACB, PLCB1, PLA2G3, CACNA1D, PLCB2, PLA2G5	19	1.72	0.023510417
hsa04720:Long-term potentiation	ADCY1, PLCB4, GRIA2, CAMK2G, CREBBP, CAMK2D, GRIN2A, RAP1A, PRKACB, PLCB1, PLCB2, ITPR1, ITPR2, PRKCB	14	1.81	0.040205858

Supplemental Table 8. Affymetrix Probe Set IDs assigned to the brown and green modules by WGCNA

ProbeSetID	GeneSymbol	Entrez.Gene	moduleColor
117_at	HSPA6	3310	brown
1431_at	CYP2E1	1571	brown
1552278_a_at	SLC46A1	113235	brown
1552279_a_at	SLC46A1	113235	brown
1552365_at	SCIN	85477	brown
1552390_a_at	C8orf47	203111	brown
1552487_a_at	BNC1	646	brown
1552496_a_at	COBL	23242	brown
1552511_a_at	CPA6	57094	brown
1552546_a_at	LETM2	137994	brown
1552578_a_at	MYO3B	140469	brown
1552618_at	STX6	10228	brown
1552620_at	SPRR4	163778	brown
1552626_a_at	TMEM163	81615	brown
1552703_s_at	CARD16 /// CASP1	834 /// 114769	brown
1552721_a_at	FGF1	2246	brown
1552755_at	C9orf66	157983	brown
1552807_a_at	SIGLEC10	89790	brown
1552903_at	B4GALNT2	124872	brown
1553023_a_at	NOX5	79400	brown
1553202_at	STOX1	219736	brown
1553226_at	LINC00052	145978	brown
1553288_a_at	NYAP1	222950	brown
1553310_at	PLB1	151056	brown
1553311_at	C20orf197	284756	brown
1553352_x_at	ERVW-1	30816	brown
1553388_at	FAM26D	221301	brown
1553425_at	WDR65	149465	brown
1553493_a_at	TDH	157739	brown
1553494_at	TDH	157739	brown
1553534_at	NLRP10	338322	brown
1553537_at	KRT73	319101	brown
1553539_at	KRT74	121391	brown
1553574_at	IFNE	338376	brown
1553672_at	ENAH	55740	brown
1553691_at	B3GALNT2	148789	brown
1553697_at	CCSAP	126731	brown
1553710_at	FAM218A	152756	brown
1553711_a_at	FAM218A	152756	brown

1553722_s_at	RNF152	220441	brown
1553734_at	AK7	122481	brown
1553829_at	CYP1B1-AS1	285154	brown
1553861_at	TCP11L2	255394	brown
1553915_at	C10orf126	283080	brown
1553972_a_at	CBS	875	brown
1553976_a_at	DPCD	25911	brown
1554067_at	C12orf66	144577	brown
1554068_s_at	C12orf66	144577	brown
1554097_a_at	MIR31HG	554202	brown
1554127_s_at	MSRB3	253827	brown
1554333_at	DNAJA4	55466	brown
1554334_a_at	DNAJA4	55466	brown
1554398_at	LYG2	254773	brown
1554411_at	CTNNB1	1499	brown
1554438_at	KIAA1217	56243	brown
1554450_s_at	MIER3	166968	brown
1554524_a_at	OLFM3	118427	brown
1554572_a_at	SUV39H2	79723	brown
1554592_a_at	SLC1A6	6511	brown
1554593_s_at	SLC1A6	6511	brown
1554609_at	LOC100287896	100287896	brown
1554795_a_at	FBLIM1	54751	brown
1554804_a_at	CLDN19	149461	brown
1555006_at	WDR66	144406	brown
1555007_s_at	WDR66	144406	brown
1555074_a_at	KCNH5	27133	brown
1555151_s_at	TDH	157739	brown
1555192_at	ZNF277	11179	brown
1555299_s_at	ERVW-1	30816	brown
1555401_at	SOHLH2	54937	brown
1555462_at	PPP1R1C	151242	brown
1555480_a_at	FBLIM1	54751	brown
1555673_at	KRTAP2-3 /// KRTAP2-4	85294 /// 730755	brown
1555774_at	ZAR1	326340	brown
1555775_a_at	ZAR1	326340	brown
1555872_a_at	LOC728903 /// MGC21881	389741 /// 728903	brown
1555971_s_at	FBXO28	23219	brown
1556037_s_at	HHIP	64399	brown
1556410_a_at	KRTAP19-1	337882	brown
1556421_at	LOC286189	286189	brown
1556423_at	VASH1	22846	brown
1556425_a_at	LOC284219	284219	brown

1556533_at	LINC00868	283994	brown
1556619_at	SHISA9	729993	brown
1556755_s_at	LOC286149	286149	brown
1556842_at	LOC286087	286087	brown
1557071_s_at	NUB1	51667	brown
1557122_s_at	GABRB2	2561	brown
1557321_a_at	CAPN14	440854	brown
1557347_at	MCPH1	79648	brown
1557458_s_at	SHB	6461	brown
1557545_s_at	RNF165	494470	brown
1557914_s_at	ERVFH21-1	100616104	brown
1557984_s_at	RPAP3	79657	brown
1558050_at	EIF2B5	8893	brown
1558177_at	TMEM229B	161145	brown
1558356_at	UACA	55075	brown
1558387_at	UG0898H09	643763	brown
1558388_a_at	UG0898H09	643763	brown
1558540_s_at	SLC2A11	66035	brown
1558577_at	LOC148709	148709	brown
1558687_a_at	FOXN1	8456	brown
1558816_at	ZNF664	144348	brown
1558920_at	SLC8A1-AS1	100128590	brown
1559256_at	MAGI1	9223	brown
1559433_at	APCDD1L-AS1	149773	brown
1559715_at	LOC100507391	100507391	brown
1559870_at	LOC100129129	100129129	brown
1559960_x_at	SYCE1L	100130958	brown
1560011_at	JRK	8629	brown
1560514_at	LINC00636	285205	brown
1560553_at	TIAF1	9220	brown
1560803_at	DNAH3	55567	brown
1560897_a_at	KRTAP10-11	386678	brown
1560988_a_at	LINC00556	100861551	brown
1561030_at	TMC7	79905	brown
1561330_at	DSG4	147409	brown
1562628_at	KRT40	125115	brown
1562629_a_at	KRT40	125115	brown
1563834_a_at	AKNAD1	254268	brown
1564274_at	C9orf47	286223	brown
1564435_a_at	KRT72	140807	brown
1564803_at	KRTAP11-1	337880	brown
1564921_at	KRTAP13-1	140258	brown

1564960_at	KRTAP7-1	337878	brown
1564974_at	KRTAP8-1	337879	brown
1565546_at	RNF141	50862	brown
1566987_s_at	LINC00674	100499466	brown
1568592_at	TRIM69	140691	brown
1568954_s_at	C16orf72	29035	brown
1569064_at	C15orf62	643338	brown
1569065_s_at	C15orf62	643338	brown
1569369_at	ZFYVE28	57732	brown
1569387_at	CSGALNACT1	55790	brown
1569433_at	SAMD5	389432	brown
1569701_at	PER3	8863	brown
1569852_at	LSMEM1	286006	brown
1569886_a_at	GLB1L3	112937	brown
1570156_s_at	FMN1	342184	brown
1570163_at	EGFLAM-AS4	100852408	brown
1570505_at	ABCB4	5244	brown
200644_at	MARCKSL1	65108	brown
200730_s_at	PTP4A1	7803	brown
200731_s_at	PTP4A1	7803	brown
200744_s_at	GNB1	2782	brown
200790_at	ODC1	4953	brown
200838_at	CTSB	1508	brown
200906_s_at	PALLD	23022	brown
200923_at	LGALS3BP	3959	brown
200924_s_at	SLC3A2	6520	brown
200998_s_at	CKAP4	10970	brown
201037_at	PFKP	5214	brown
201043_s_at	ANP32A	8125	brown
201051_at	ANP32A	8125	brown
201109_s_at	THBS1	7057	brown
201110_s_at	THBS1	7057	brown
201147_s_at	TIMP3	7078	brown
201148_s_at	TIMP3	7078	brown
201149_s_at	TIMP3	7078	brown
201150_s_at	TIMP3	7078	brown
201206_s_at	RRBP1	6238	brown
201208_s_at	TNFAIP1	7126	brown
201266_at	TXNRD1	7296	brown
201340_s_at	ENC1	8507	brown
201341_at	ENC1	8507	brown
201422_at	IFI30 /// PIK3R2	5296 /// 10437	brown

201430_s_at	DPYSL3	1809	brown
201506_at	TGFBI	7045	brown
201521_s_at	NCBP2	22916	brown
201559_s_at	CLIC4	25932	brown
201616_s_at	CALD1	800	brown
201631_s_at	IER3	8870	brown
201645_at	TNC	3371	brown
201723_s_at	GALNT1	2589	brown
201767_s_at	ELAC2	60528	brown
201846_s_at	RYBP	23429	brown
201848_s_at	BNIP3	664	brown
201860_s_at	PLAT	5327	brown
201871_s_at	UBXN1	51035	brown
201925_s_at	CD55	1604	brown
201981_at	PAPPA	5069	brown
202016_at	MEST	4232	brown
202066_at	PPFIA1	8500	brown
202075_s_at	PLTP	5360	brown
202103_at	BRD4	23476	brown
202147_s_at	IFRD1	3475	brown
202166_s_at	PPP1R2	5504	brown
202178_at	PRKCZ	5590	brown
202206_at	ARL4C	10123	brown
202207_at	ARL4C	10123	brown
202208_s_at	ARL4C	10123	brown
202272_s_at	FBXO28	23219	brown
202290_at	PDAP1	11333	brown
202295_s_at	CTSH	1512	brown
202307_s_at	TAP1	6890	brown
202357_s_at	CFB	629	brown
202376_at	SERPINA3	12	brown
202391_at	BASP1	10409	brown
202416_at	DNAJC7	7266	brown
202435_s_at	CYP1B1	1545	brown
202436_s_at	CYP1B1	1545	brown
202458_at	PRSS23	11098	brown
202461_at	EIF2B2	8892	brown
202468_s_at	CTNNAL1	8727	brown
202531_at	IRF1	3659	brown
202570_s_at	DLGAP4	22839	brown
202613_at	CTPS1	1503	brown
202626_s_at	LYN	4067	brown
202659_at	PSMB10	5699	brown

202679_at	NPC1	4864	brown
202693_s_at	STK17A	9263	brown
202695_s_at	STK17A	9263	brown
202744_at	SLC20A2	6575	brown
202752_x_at	SLC7A8	23428	brown
202793_at	LPCAT3	10162	brown
202805_s_at	ABCC1	4363	brown
202833_s_at	SERPINA1	5265	brown
202883_s_at	PPP2R1B	5519	brown
202884_s_at	PPP2R1B	5519	brown
202886_s_at	PPP2R1B	5519	brown
202889_x_at	MAP7	9053	brown
202895_s_at	SIRPA	140885	brown
202896_s_at	SIRPA	140885	brown
202897_at	SIRPA	140885	brown
202923_s_at	GCLC	2729	brown
202935_s_at	SOX9	6662	brown
202936_s_at	SOX9	6662	brown
202949_s_at	FHL2	2274	brown
202969_at	DYRK2	8445	brown
202990_at	PYGL	5836	brown
203004_s_at	MEF2D	4209	brown
203016_s_at	SSX2IP	117178	brown
203017_s_at	SSX2IP	117178	brown
203018_s_at	SSX2IP	117178	brown
203021_at	SLPI	6590	brown
203087_s_at	KIF2A	3796	brown
203129_s_at	KIF5C	3800	brown
203130_s_at	KIF5C	3800	brown
203148_s_at	TRIM14	9830	brown
203150_at	RABEPK	10244	brown
203158_s_at	GLS	2744	brown
203184_at	FBN2	2201	brown
203221_at	TLE1	7088	brown
203222_s_at	TLE1	7088	brown
203236_s_at	LGALS9	3965	brown
203287_at	LAD1	3898	brown
203304_at	BAMBI	25805	brown
203349_s_at	ETV5	2119	brown
203355_s_at	PSD3	23362	brown
203367_at	DUSP14	11072	brown
203413_at	NELL2	4753	brown
203417_at	MFAP2	4237	brown

203560_at	GGH	8836	brown
203570_at	LOXL1	4016	brown
203574_at	NFIL3	4783	brown
203596_s_at	IFIT5	24138	brown
203650_at	PROCR	10544	brown
203693_s_at	E2F3	1871	brown
203705_s_at	FZD7	8324	brown
203706_s_at	FZD7	8324	brown
203707_at	LINC00921 /// ZNF263	10127 /// 283876	brown
203742_s_at	LOC732360 /// TDG	6996 /// 732360	brown
203764_at	DLGAP5	9787	brown
203790_s_at	HRSP12	10247	brown
203797_at	VSNL1	7447	brown
203798_s_at	VSNL1	7447	brown
203821_at	HBEGF	1839	brown
203827_at	WIPI1	55062	brown
203842_s_at	MAPRE3	22924	brown
203882_at	IRF9	10379	brown
203904_x_at	CD82	3732	brown
203921_at	CHST2	9435	brown
203925_at	GCLM	2730	brown
203929_s_at	MAPT	4137	brown
203935_at	ACVR1	90	brown
203968_s_at	CDC6	990	brown
203987_at	FZD6	8323	brown
204141_at	TUBB2A	7280	brown
204197_s_at	RUNX3	864	brown
204198_s_at	RUNX3	864	brown
204256_at	ELOVL6	79071	brown
204268_at	S100A2	6273	brown
204279_at	PSMB9	5698	brown
204284_at	PPP1R3C	5507	brown
204306_s_at	CD151	977	brown
204351_at	S100P	6286	brown
204378_at	BCAS1	8537	brown
204394_at	SLC43A1	8501	brown
204448_s_at	PDCL	5082	brown
204501_at	NOV	4856	brown
204541_at	SEC14L2	23541	brown
204556_s_at	DZIP1	22873	brown
204557_s_at	DZIP1	22873	brown
204614_at	SERPINB2	5055	brown
204656_at	SHB	6461	brown

204657_s_at	SHB	6461	brown
204665_at	SIKE1	80143	brown
204687_at	PARM1	25849	brown
204705_x_at	ALDOB	229	brown
204720_s_at	DNAJC6	9829	brown
204730_at	RIMS3	9783	brown
204733_at	KLK6	5653	brown
204736_s_at	CSPG4	1464	brown
204751_x_at	DSC2	1824	brown
204761_at	USP6NL	9712	brown
204790_at	SMAD7	4092	brown
204818_at	HSD17B2	3294	brown
204820_s_at	BTN3A2 /// BTN3A3	10384 /// 11118	brown
204821_at	BTN3A3	10384	brown
204869_at	PCSK2	5126	brown
204870_s_at	PCSK2	5126	brown
204903_x_at	ATG4B	23192	brown
204992_s_at	PFN2	5217	brown
204994_at	MX2	4600	brown
205027_s_at	MAP3K8	1326	brown
205053_at	PRIM1	5557	brown
205073_at	CYP2J2	1573	brown
205081_at	CRIP1	1396	brown
205093_at	PLEKHA6	22874	brown
205125_at	PLCD1	5333	brown
205138_s_at	UST	10090	brown
205139_s_at	UST	10090	brown
205150_s_at	TRIL	9865	brown
205151_s_at	TRIL	9865	brown
205157_s_at	JUP /// KRT17	3728 /// 3872	brown
205184_at	GNG4	2786	brown
205249_at	EGR2	1959	brown
205254_x_at	TCF7	6932	brown
205255_x_at	TCF7	6932	brown
205268_s_at	ADD2	119	brown
205289_at	BMP2	650	brown
205290_s_at	BMP2	650	brown
205334_at	S100A1	6271	brown
205374_at	SLN	6588	brown
205375_at	MDFI	4188	brown
205376_at	INPP4B	8821	brown
205393_s_at	CHEK1	1111	brown
205436_s_at	H2AFX	3014	brown

205471_s_at	DACH1	1602	brown
205472_s_at	DACH1	1602	brown
205515_at	PRSS12	8492	brown
205518_s_at	CMAHP	8418	brown
205525_at	CALD1	800	brown
205534_at	PCDH7	5099	brown
205535_s_at	PCDH7	5099	brown
205551_at	SV2B	9899	brown
205555_s_at	MSX2	4488	brown
205566_at	ABHD2	11057	brown
205586_x_at	VGF	7425	brown
205590_at	RASGRP1	10125	brown
205592_at	SLC4A1	6521	brown
205612_at	MMRN1	22915	brown
205635_at	KALRN	8997	brown
205637_s_at	SH3GL3	6457	brown
205676_at	CYP27B1	1594	brown
205686_s_at	CD86	942	brown
205687_at	UBFD1	56061	brown
205713_s_at	COMP	1311	brown
205724_at	PKP1	5317	brown
205732_s_at	NCOA2	10499	brown
205747_at	CBLN1	869	brown
205833_s_at	PART1	25859	brown
205841_at	JAK2	3717	brown
205864_at	SLC7A4	6545	brown
205899_at	CCNA1	8900	brown
205918_at	SLC4A3	6508	brown
205926_at	IL27RA	9466	brown
205931_s_at	CREB5 /// LOC401317	9586 /// 401317	brown
205941_s_at	COL10A1	1300	brown
205988_at	CD84	8832	brown
205990_s_at	WNT5A	7474	brown
206027_at	S100A3	6274	brown
206044_s_at	BRAF	673	brown
206082_at	HCP5	10866	brown
206089_at	NELL1	4745	brown
206116_s_at	TPM1	7168	brown
206132_at	MCC	4163	brown
206137_at	RIMS2	9699	brown
206140_at	LHX2	9355	brown
206170_at	ADRB2	154	brown
206171_at	ADORA3	140	brown

206191_at	ENTPD3	956	brown
206224_at	CST1	1469	brown
206235_at	LIG4	3981	brown
206237_s_at	NRG1	3084	brown
206259_at	PROC	5624	brown
206312_at	GUCY2C	2984	brown
206315_at	CRLF1	9244	brown
206326_at	GRP	2922	brown
206356_s_at	GNAL	2774	brown
206367_at	REN	5972	brown
206375_s_at	HSPB3	8988	brown
206376_at	SLC6A15	55117	brown
206382_s_at	BDNF	627	brown
206423_at	ANGPTL7	10218	brown
206439_at	EPYC	1833	brown
206447_at	CELA2A /// CELA2B	51032 /// 63036	brown
206448_at	ZNF365	22891	brown
206504_at	CYP24A1	1591	brown
206540_at	GLB1L	79411	brown
206581_at	BNC1	646	brown
206591_at	RAG1	5896	brown
206595_at	CST6	1474	brown
206604_at	OVOL1	5017	brown
206605_at	ENDOU	8909	brown
206665_s_at	BCL2L1	598	brown
206677_at	KRT31	3881	brown
206693_at	IL7	3574	brown
206710_s_at	EPB41L3	23136	brown
206723_s_at	LPAR2	9170	brown
206737_at	WNT11	7481	brown
206746_at	BFSP1	631	brown
206758_at	EDN2	1907	brown
206774_at	FRMPD1	22844	brown
206788_s_at	CBFB	865	brown
206882_at	SLC1A6	6511	brown
206912_at	FOXE1	2304	brown
206935_at	PCDH8	5100	brown
206969_at	KRT34 /// LOC100653049	3885 /// 100653049	brown
206972_s_at	GPR161	23432	brown
206987_x_at	FGF18	8817	brown
206994_at	CST4	1472	brown
207037_at	TNFRSF11A	8792	brown
207062_at	IAPP	3375	brown

207065_at	KRT75	9119	brown
207074_s_at	SLC18A1	6570	brown
207082_at	CSF1	1435	brown
207113_s_at	TNF	7124	brown
207146_at	KRT32	3882	brown
207147_at	DLX2	1746	brown
207154_at	DIO3	1735	brown
207192_at	DNASE1L2	1775	brown
207195_at	CNTN6	27255	brown
207268_x_at	ABI2	10152	brown
207344_at	AKAP3	10566	brown
207354_at	CCL16	6360	brown
207367_at	ATP12A	479	brown
207457_s_at	LY6G6D	58530	brown
207547_s_at	FAM107A /// LOC101059937	11170 /// 101059937	brown
207669_at	KRT83	3889	brown
207670_at	KRT85	3891	brown
207716_at	KRT38	8687	brown
207759_s_at	DISC1 /// TSNAX-DISC1	27185 /// 100303453	brown
207764_s_at	HIPK3	10114	brown
207787_at	KRT33B	3884	brown
207826_s_at	ID3	3399	brown
207908_at	KRT2	3849	brown
207955_at	CCL27	10850	brown
207957_s_at	PRKCB	5579	brown
208002_s_at	ACOT7	11332	brown
208062_s_at	NRG2	9542	brown
208092_s_at	FAM49A	81553	brown
208161_s_at	ABCC3	8714	brown
208216_at	DLX4	1748	brown
208250_s_at	DMBT1	1755	brown
208290_s_at	EIF5	1983	brown
208378_x_at	FGF5	2250	brown
208483_x_at	KRT33A	3883	brown
208490_x_at	HIST1H2BC /// HIST1H2BE /// HIST1H2BF /// HIST1H2BG /// HIST1H2BI	8339 /// 8343 /// 8344 /// 8346 /// 8347	brown
208523_x_at	HIST1H2BC /// HIST1H2BE /// HIST1H2BF /// HIST1H2BG /// HIST1H2BI	8339 /// 8343 /// 8344 /// 8346 /// 8347	brown
208527_x_at	HIST1H2BC /// HIST1H2BE /// HIST1H2BF /// HIST1H2BG /// HIST1H2BI	8339 /// 8343 /// 8344 /// 8346 /// 8347	brown

208532_x_at	KRTAP5-8	57830	brown
208546_x_at	HIST1H2BH	8345	brown
208555_x_at	CST2	1470	brown
208579_x_at	H2BFS	54145	brown
208583_x_at	HIST1H2AG /// HIST1H2AH /// HIST1H2AI /// HIST1H2AJ /// HIST1H2AK /// HIST1H2AL /// HIST1H2AM /// HIST1H3F	8329 /// 8330 /// 8331 /// 8332 /// 8336 /// 8968 /// 8969 /// 85235	brown
208588_at	FKSG2	59347	brown
208613_s_at	FLNB	2317	brown
208633_s_at	MACF1	23499	brown
208637_x_at	ACTN1	87	brown
208653_s_at	CD164	8763	brown
208702_x_at	APLP2	334	brown
208705_s_at	EIF5	1983	brown
208708_x_at	EIF5	1983	brown
208730_x_at	RAB2A	5862	brown
208785_s_at	MAP1LC3B	81631	brown
209062_x_at	NCOA3	8202	brown
209118_s_at	TUBA1A	7846	brown
209125_at	KRT6A	3853	brown
209126_x_at	KRT6B	3854	brown
209152_s_at	TCF3	6929	brown
209162_s_at	PRPF4	9128	brown
209228_x_at	TUSC3	7991	brown
209242_at	PEG3	5178	brown
209267_s_at	SLC39A8	64116	brown
209273_s_at	ISCA1	81689	brown
209280_at	MRC2	9902	brown
209286_at	CDC42EP3	10602	brown
209288_s_at	CDC42EP3	10602	brown
209343_at	EFHD1	80303	brown
209369_at	ANXA3	306	brown
209372_x_at	TUBB2A /// TUBB2B	7280 /// 347733	brown
209387_s_at	TM4SF1	4071	brown
209447_at	SYNE1	23345	brown
209588_at	EPHB2	2048	brown
209589_s_at	EPHB2	2048	brown
209591_s_at	BMP7	655	brown
209615_s_at	PAK1	5058	brown
209617_s_at	CTNND2	1501	brown
209618_at	CTNND2	1501	brown
209626_s_at	OSBPL3	26031	brown

209631_s_at	GPR37 /// SEL1L2	2861 /// 80343	brown
209640_at	PML	5371	brown
209683_at	FAM49A	81553	brown
209685_s_at	PRKCB	5579	brown
209698_at	CCHCR1	54535	brown
209757_s_at	MYCN	4613	brown
209792_s_at	KLK10	5655	brown
209793_at	GRIA1	2890	brown
209799_at	PRKAA1	5562	brown
209800_at	KRT16	3868	brown
209806_at	HIST1H2BK	85236	brown
209859_at	TRIM9	114088	brown
209866_s_at	LPHN3	23284	brown
209867_s_at	LPHN3	23284	brown
209911_x_at	HIST1H2BD	3017	brown
209921_at	SLC7A11	23657	brown
209933_s_at	CD300A	11314	brown
209938_at	TADA2A	6871	brown
209945_s_at	GSK3B	2932	brown
209970_x_at	CASP1	834	brown
209975_at	CYP2E1	1571	brown
209976_s_at	CYP2E1	1571	brown
209981_at	CSDC2	27254	brown
210002_at	GATA6	2627	brown
210007_s_at	GPD2	2820	brown
210020_x_at	CALML3	810	brown
210048_at	NAPG	8774	brown
210074_at	CTSL2	1515	brown
210082_at	ABCA4	24	brown
210115_at	RPL39L	116832	brown
210145_at	PLA2G4A	5321	brown
210148_at	HIPK3	10114	brown
210186_s_at	FKBP1A	2280	brown
210235_s_at	PPFIA1	8500	brown
210261_at	KCNK2	3776	brown
210276_s_at	TRIOBP	11078	brown
210305_at	PDE4DIP	9659	brown
210311_at	FGF5	2250	brown
210319_x_at	MSX2	4488	brown
210335_at	RASSF9	9182	brown
210385_s_at	ERAP1	51752	brown

210387_at	NCALD	83988	brown
210393_at	LGR5	8549	brown
210419_at	BARX2	8538	brown
210473_s_at	GPR125	166647	brown
210521_s_at	FETUB	26998	brown
210534_s_at	B9D1	27077	brown
210535_at	B9D1	27077	brown
210568_s_at	RECQL	5965	brown
210571_s_at	CMAHP	8418	brown
210689_at	CLDN14	23562	brown
210868_s_at	ELOVL6	79071	brown
210871_x_at	SSX2IP	117178	brown
210889_s_at	FCGR2B	2213	brown
210929_s_at	AHSG	197	brown
210942_s_at	ST3GAL6	10402	brown
210948_s_at	LEF1	51176	brown
210954_s_at	TSC22D2	9819	brown
211018_at	LSS	4047	brown
211029_x_at	FGF18	8817	brown
211062_s_at	CPZ /// GPR78	8532 /// 27201	brown
211071_s_at	MLLT11	10962	brown
211075_s_at	CD47	961	brown
211113_s_at	ABCG1	9619	brown
211160_x_at	ACTN1	87	brown
211219_s_at	LHX2	9355	brown
211222_s_at	HAP1	9001	brown
211357_s_at	ALDOB	229	brown
211361_s_at	SERPINB13	5275	brown
211362_s_at	SERPINB13	5275	brown
211366_x_at	CASP1	834	brown
211368_s_at	CASP1	834	brown
211376_s_at	NSMCE4A	54780	brown
211404_s_at	APLP2	334	brown
211429_s_at	SERPINA1	5265	brown
211485_s_at	FGF18	8817	brown
211518_s_at	BMP4	652	brown
211520_s_at	GRIA1	2890	brown
211538_s_at	HSPA2	3306	brown
211764_s_at	UBE2D1	7321	brown
211776_s_at	EPB41L3	23136	brown
211793_s_at	ABI2	10152	brown
211828_s_at	TNIK	23043	brown
211844_s_at	NRP2	8828	brown

212098_at	MGAT5	4249	brown
212144_at	SUN2	25777	brown
212154_at	SDC2	6383	brown
212157_at	SDC2	6383	brown
212236_x_at	JUP /// KRT17	3728 /// 3872	brown
212365_at	MYO1B	4430	brown
212471_at	AVL9	23080	brown
212478_at	RMND5A	64795	brown
212479_s_at	RMND5A	64795	brown
212521_s_at	PDE8A	5151	brown
212531_at	LCN2	3934	brown
212580_at	CAST	831	brown
212613_at	BTN3A2	11118	brown
212623_at	TMEM41B	440026	brown
212681_at	EPB41L3	23136	brown
212686_at	PPM1H	57460	brown
212703_at	TLN2	83660	brown
212738_at	ARHGAP19	84986	brown
212807_s_at	SORT1	6272	brown
212816_s_at	CBS	875	brown
212821_at	PLEKHG3	26030	brown
212822_at	HEG1	57493	brown
212829_at	PIP4K2A	5305	brown
212841_s_at	PPFIBP2	8495	brown
212902_at	SEC24A	10802	brown
212915_at	PDZRN3	23024	brown
212942_s_at	KIAA1199	57214	brown
213035_at	ANKRD28	23243	brown
213038_at	RNF19B	127544	brown
213069_at	HEG1	57493	brown
213096_at	TMCC2	9911	brown
213100_at	UNC5B	219699	brown
213107_at	TNIK	23043	brown
213122_at	TSPYL5	85453	brown
213344_s_at	H2AFX	3014	brown
213358_at	SOGA2	23255	brown
213385_at	CHN2	1124	brown
213413_at	STON1	11037	brown
213418_at	HSPA6	3310	brown
213419_at	APBB2	323	brown
213423_x_at	TUSC3	7991	brown
213425_at	WNT5A	7474	brown
213438_at	NFASC	23114	brown

213439_x_at	RUNDC3A	10900	brown
213489_at	MAPRE2	10982	brown
213558_at	PCLO	27445	brown
213631_x_at	DHODH	1723	brown
213638_at	PHACTR1	221692	brown
213692_s_at	VDR	7421	brown
213711_at	KRT81	3887	brown
213713_s_at	GLB1L2	89944	brown
213749_at	MASP1	5648	brown
213759_at	ARL4C	10123	brown
213774_s_at	PPP1R2	5504	brown
213780_at	TCHH	7062	brown
213796_at	SPRR1A	6698	brown
213802_at	PRSS12	8492	brown
213836_s_at	WIPI1	55062	brown
213844_at	HOXA5	3202	brown
213849_s_at	PPP2R2B	5521	brown
213880_at	LGR5	8549	brown
213895_at	EMP1	2012	brown
213899_at	METAP2	10988	brown
213909_at	LRRC15	131578	brown
213912_at	TBC1D30	23329	brown
213913_s_at	TBC1D30	23329	brown
213924_at	GNAL	2774	brown
213967_at	RALYL	138046	brown
214000_s_at	RGS10	6001	brown
214012_at	ERAP1	51752	brown
214036_at	EFNA5	1946	brown
214068_at	BEAN1	146227	brown
214104_at	GPR161	23432	brown
214121_x_at	PDLIM7	9260	brown
214163_at	HSPB11	51668	brown
214168_s_at	TJP1	7082	brown
214186_s_at	HCG26	352961	brown
214203_s_at	PRODH	5625	brown
214222_at	DNAH7	56171	brown
214226_at	PRSS53	339105	brown
214230_at	CDC42	998	brown
214270_s_at	MAPRE3	22924	brown
214284_s_at	FGF18	8817	brown
214285_at	FABP3	2170	brown
214321_at	NOV	4856	brown
214324_at	GP2	2813	brown

214417_s_at	FETUB	26998	brown
214423_x_at	ALDOB	229	brown
214424_s_at	ALDOB	229	brown
214433_s_at	SELENBP1	8991	brown
214446_at	ELL2	22936	brown
214517_at	KRTAP5-9	3846	brown
214549_x_at	SPRR1A	6698	brown
214576_at	KRT36	8689	brown
214580_x_at	KRT6A /// KRT6B /// KRT6C	3853 /// 3854 /// 286887	brown
214587_at	COL8A1	1295	brown
214590_s_at	UBE2D1	7321	brown
214632_at	NRP2	8828	brown
214636_at	CALCB	797	brown
214657_s_at	MIR612 /// NEAT1	283131 /// 693197	brown
214755_at	UAP1L1	91373	brown
214858_at	PP14571	100130449	brown
214875_x_at	APLP2	334	brown
214887_at	N4BP2L1	90634	brown
214906_x_at	N4BP2L1	90634	brown
214939_x_at	MLLT4	4301	brown
214956_at	AAK1	22848	brown
214981_at	POSTN	10631	brown
215033_at	TM4SF1	4071	brown
215034_s_at	TM4SF1	4071	brown
215150_at	YOD1	55432	brown
215189_at	KRT86 /// LOC100509764	3892 /// 100509764	brown
215222_x_at	MACF1	23499	brown
215253_s_at	RCAN1	1827	brown
215271_at	TNN	63923	brown
215274_at	SLC12A3	6559	brown
215302_at	LOC257152	257152	brown
215313_x_at	HLA-A	3105	brown
215346_at	CD40	958	brown
215471_s_at	MAP7	9053	brown
215547_at	TSC22D2	9819	brown
215779_s_at	HIST1H2BC /// HIST1H2BE /// HIST1H2BF /// HIST1H2BG /// HIST1H2BI /// NCALD	8339 /// 8343 /// 8344 /// 8346 /// 8347 /// 83988	brown
215785_s_at	CYFIP2	26999	brown
215817_at	SERPINB13	5275	brown
216086_at	SV2C	22987	brown
216092_s_at	SLC7A8	23428	brown
216235_s_at	EDNRA	1909	brown

216258_s_at	SERPINB13	5275	brown
216504_s_at	SLC39A8	64116	brown
216595_at	FAM186A	121006	brown
216600_x_at	ALDOB	229	brown
216604_s_at	SLC7A8	23428	brown
216622_at	LAMB4	22798	brown
216810_at	KRTAP4-7	100132476	brown
216921_s_at	KRT35	3886	brown
217025_s_at	DBN1	1627	brown
217031_at	KRT84	3890	brown
217185_s_at	ZNF259 /// ZNF259P1	8882 /// 442240	brown
217197_x_at	N4BP2L1	90634	brown
217238_s_at	ALDOB	229	brown
217272_s_at	SERPINB13	5275	brown
217395_at	MT4	84560	brown
217428_s_at	COL10A1	1300	brown
217557_s_at	CPM	1368	brown
217678_at	SLC7A11	23657	brown
217738_at	NAMPT	10135	brown
217739_s_at	NAMPT	10135	brown
217771_at	GOLM1	51280	brown
217787_s_at	GALNT2	2590	brown
217799_x_at	UBE2H	7328	brown
217875_s_at	PMEPA1	56937	brown
217897_at	FXYD6	53826	brown
217905_at	MCMBP	79892	brown
217921_at	MAN1A2	10905	brown
217924_at	C6orf106	64771	brown
217933_s_at	LAP3	51056	brown
218032_at	SNN	8303	brown
218051_s_at	NT5DC2	64943	brown
218113_at	TMEM2	23670	brown
218168_s_at	ADCK3	56997	brown
218257_s_at	UGGT1	56886	brown
218266_s_at	NCS1	23413	brown
218326_s_at	LGR4	55366	brown
218368_s_at	TNFRSF12A	51330	brown
218384_at	CARHSP1	23589	brown
218433_at	PANK3	79646	brown
218458_at	GMCL1	64395	brown
218487_at	ALAD	210	brown
218489_s_at	ALAD	210	brown
218502_s_at	TRPS1	7227	brown

218543_s_at	PARP12	64761	brown
218574_s_at	LMCD1	29995	brown
218610_s_at	CPPED1	55313	brown
218627_at	DRAM1	55332	brown
218717_s_at	LEPREL1	55214	brown
218736_s_at	PALMD	54873	brown
218813_s_at	SH3GLB2	56904	brown
218834_s_at	TMEM132A	54972	brown
218851_s_at	SFT2D3 /// WDR33	55339 /// 84826	brown
218854_at	DSE	29940	brown
218876_at	TPPP3	51673	brown
218888_s_at	NETO2	81831	brown
218929_at	CDKN2AIP	55602	brown
218935_at	EHD3	30845	brown
218980_at	FHOD3	80206	brown
219018_s_at	CCDC85C	317762	brown
219033_at	PARP8	79668	brown
219054_at	NPR3	4883	brown
219090_at	SLC24A3	57419	brown
219132_at	PELI2	57161	brown
219142_at	RASL11B	65997	brown
219225_at	PGBD5	79605	brown
219229_at	SLCO3A1	28232	brown
219232_s_at	EGLN3	112399	brown
219234_x_at	SCRN3	79634	brown
219250_s_at	FLRT3	23767	brown
219270_at	CHAC1	79094	brown
219271_at	GALNT14	79623	brown
219300_s_at	CNTNAP2	26047	brown
219301_s_at	CNTNAP2	26047	brown
219330_at	VANGL1	81839	brown
219340_s_at	CLN8	2055	brown
219344_at	SLC29A3	55315	brown
219349_s_at	EXOC2	55770	brown
219367_s_at	NRP2	8828	brown
219369_s_at	OTUB2	78990	brown
219383_at	PRR5L	79899	brown
219463_at	LAMP5	24141	brown
219511_s_at	SNCAIP	9627	brown
219521_at	B3GAT1	27087	brown
219554_at	RHCG	51458	brown
219610_at	ARHGEF28	64283	brown
219612_s_at	FGG	2266	brown

219648_at	MREG	55686	brown
219659_at	ATP8A2	51761	brown
219660_s_at	ATP8A2	51761	brown
219671_at	HPCAL4	51440	brown
219683_at	FZD3	7976	brown
219687_at	HHAT	55733	brown
219688_at	BBS7	55212	brown
219691_at	SAMD9	54809	brown
219697_at	HS3ST2	9956	brown
219750_at	TMEM144	55314	brown
219768_at	VTCN1	79679	brown
219775_s_at	CPLX3	594855	brown
219806_s_at	SMCO4	56935	brown
219828_at	RABL6	55684	brown
219832_s_at	HOXC13	3229	brown
219842_at	ARL15	54622	brown
219869_s_at	SLC39A8	64116	brown
219870_at	ATF7IP2	80063	brown
219916_s_at	RNF39	80352	brown
219926_at	POPDC3	64208	brown
219932_at	SLC27A6	28965	brown
219946_x_at	MYH14	79784	brown
219987_at	ERVMER34-1	100288413	brown
219991_at	SLC2A9	56606	brown
220011_at	AUNIP	79000	brown
220021_at	TMC7	79905	brown
220090_at	CRNN	49860	brown
220129_at	CCDC169-SOHLH2 /// SOHLH2	54937 /// 100526761	brown
220203_at	BMP8A	353500	brown
220244_at	LINC00312	29931	brown
220245_at	SLC45A2	51151	brown
220256_s_at	OXCT2	64064	brown
220272_at	BNC2	54796	brown
220317_at	LRAT	9227	brown
220351_at	CCRL1	51554	brown
220406_at	TGFB2	7042	brown
220407_s_at	TGFB2	7042	brown
220431_at	TMPRSS11E	28983	brown
220456_at	SPTLC3	55304	brown
220489_s_at	SERINC2	347735	brown
220624_s_at	ELF5	2001	brown
220635_at	PSORS1C2	170680	brown
220641_at	NOX5	79400	brown

220724_at	CWH43	80157	brown
220753_s_at	CRYL1	51084	brown
220768_s_at	CSNK1G3	1456	brown
220779_at	PADI3	51702	brown
220780_at	PLA2G3	50487	brown
220782_x_at	KLK12	43849	brown
220800_s_at	TMOD3	29766	brown
220840_s_at	C1orf112	55732	brown
220962_s_at	PADI1	29943	brown
220970_s_at	KRTAP2-3 /// KRTAP2-4	85294 /// 730755	brown
220972_s_at	KRTAP9-9	81870	brown
220976_s_at	KRTAP1-1	81851	brown
220978_at	KRTAP1-3	81850	brown
220984_s_at	SLCO5A1	81796	brown
220999_s_at	CYFIP2	26999	brown
221102_s_at	TRPM6	140803	brown
221114_at	AMBN	258	brown
221222_s_at	C1orf56	54964	brown
221234_s_at	BACH2	60468	brown
221245_s_at	FZD5	7855	brown
221266_s_at	DCSTAMP	81501	brown
221272_s_at	C1orf21	81563	brown
221292_at	PTCH2	8643	brown
221297_at	GPRC5D	55507	brown
221389_at	PLA2G2E	30814	brown
221416_at	PLA2G2F	64600	brown
221425_s_at	ISCA1	81689	brown
221455_s_at	WNT3	7473	brown
221497_x_at	EGLN1	54583	brown
221515_s_at	LCMT1	51451	brown
221524_s_at	RRAGD	58528	brown
221539_at	EIF4EBP1	1978	brown
221558_s_at	LEF1	51176	brown
221627_at	TRIM10	10107	brown
221648_s_at	AGMAT	79814	brown
221716_s_at	ACSBG2	81616	brown
221803_s_at	NRBF2	29982	brown
221816_s_at	PHF11	51131	brown
221898_at	PDPN	10630	brown
221962_s_at	UBE2H	7328	brown
221978_at	HLA-F	3134	brown
222007_s_at	FKBP8	23770	brown
222015_at	CSNK1E	1454	brown

222067_x_at	HIST1H2BD		3017	brown
222075_s_at	OAZ3		51686	brown
222162_s_at	ADAMTS1		9510	brown
222164_at	FGFR1		2260	brown
222351_at	PPP2R1B		5519	brown
222386_s_at	COPZ1		22818	brown
222420_s_at	UBE2H		7328	brown
222429_at	DBNL		28988	brown
222486_s_at	ADAMTS1		9510	brown
222537_s_at	CDC42SE1		56882	brown
222581_at	XPR1		9213	brown
222598_s_at	NAV2		89797	brown
222651_s_at	TRPS1		7227	brown
222653_at	PNPO		55163	brown
222701_s_at	CHCHD7		79145	brown
222702_x_at	CRIPT		9419	brown
222706_at	CWC25		54883	brown
222774_s_at	NETO2		81831	brown
222783_s_at	SMOC1		64093	brown
222784_at	SMOC1		64093	brown
222799_at	WDR91		29062	brown
222833_at	LPCAT2		54947	brown
222853_at	FLRT3		23767	brown
222871_at	KLHDC8A		55220	brown
222874_s_at	CLN8		2055	brown
222878_s_at	OTUB2		78990	brown
222890_at	CCDC113		29070	brown
222927_s_at	CPLX3		594855	brown
222942_s_at	LOC100505519 /// TIAM2		26230 /// 100505519	brown
223044_at	SLC40A1		30061	brown
223078_s_at	TMOD3		29766	brown
223126_s_at	C1orf21		81563	brown
223145_s_at	AKIRIN2		55122	brown
223220_s_at	PARP9		83666	brown
223278_at	GJB2		2706	brown
223281_s_at	COX15		1355	brown
223349_s_at	BOK		666	brown
223362_s_at		3-Sep	55964	brown
223395_at	ABI3BP		25890	brown
223427_s_at	EPB41L4B		54566	brown
223461_at	TBC1D7		51256	brown
223468_s_at	RGMA		56963	brown
223471_at	RAB3IP		117177	brown

223498_at	SPECC1	92521	brown
223503_at	TMEM163	81615	brown
223538_at	SERF1A /// SERF1B	8293 /// 728492	brown
223539_s_at	SERF1A /// SERF1B	8293 /// 728492	brown
223548_at	SWT1	54823	brown
223593_at	AADAT	51166	brown
223599_at	TRIM6	117854	brown
223650_s_at	NRBF2	29982	brown
223682_s_at	EIF1AD	84285	brown
223690_at	LTBP2	4053	brown
223720_at	SPINK7	84651	brown
223739_at	PADI1	29943	brown
223775_at	HHIP	64399	brown
223786_at	CHST6	4166	brown
223805_at	OSBPL6	114880	brown
223812_at	FAM178B	51252	brown
223821_s_at	SUSD4	55061	brown
223822_at	SUSD4	55061	brown
223824_at	RNLS	55328	brown
223912_s_at	CLN8	2055	brown
223987_at	CHRDL2	25884	brown
223991_s_at	GALNT2	2590	brown
224022_x_at	WNT16	51384	brown
224218_s_at	TRPS1	7227	brown
224225_s_at	ETV7	51513	brown
224262_at	IL1F10	84639	brown
224269_at	KRTAP4-12	83755	brown
224277_at	MOP-1	643616	brown
224325_at	FZD8	8325	brown
224397_s_at	TMTC1	83857	brown
224412_s_at	TRPM6	140803	brown
224520_s_at	BEST3	144453	brown
224593_at	ZNF664	144348	brown
224674_at	TTYH3	80727	brown
224701_at	PARP14	54625	brown
224724_at	SULF2	55959	brown
224762_at	SERINC2	347735	brown
224836_at	TP53INP2	58476	brown
224840_at	FKBP5	2289	brown
224856_at	FKBP5	2289	brown
224901_at	SCD5	79966	brown
224940_s_at	PAPPA	5069	brown
224941_at	PAPPA	5069	brown

224950_at	PTGFRN	5738	brown
225076_s_at	ZNFX1	57169	brown
225105_at	C12orf75	387882	brown
225119_at	CHMP4B	128866	brown
225142_at	JHDM1D	80853	brown
225258_at	FBLIM1	54751	brown
225282_at	SMAP2	64744	brown
225295_at	SLC39A10	57181	brown
225390_s_at	KLF13	51621	brown
225491_at	SLC1A2	6506	brown
225496_s_at	SYTL2	54843	brown
225545_at	EEF2K /// LOC101060570	29904 /// 101060570	brown
225597_at	SLC45A4	57210	brown
225659_at	SPOPL	339745	brown
225660_at	SEMA6A	57556	brown
225685_at	CDC42EP3	10602	brown
225686_at	SKA2	348235	brown
225723_at	CCDC167	154467	brown
225766_s_at	TNPO1	3842	brown
225803_at	FBXO32	114907	brown
225809_at	PARM1	25849	brown
225904_at	CCSAP	126731	brown
225935_at	CUX1	1523	brown
225950_at	SAMD8	142891	brown
225985_at	PRKAA1	5562	brown
226003_at	KIF21A	55605	brown
226009_at	DPCD	25911	brown
226026_at	DIRC2	84925	brown
226030_at	ACADSB	36	brown
226056_at	ARHGAP31	57514	brown
226065_at	PRICKLE1	144165	brown
226069_at	PRICKLE1	144165	brown
226122_at	PLEKHG1	57480	brown
226187_at	CDS1	1040	brown
226287_at	CCDC34	91057	brown
226313_at	C10orf35	219738	brown
226380_at	PTPN21	11099	brown
226390_at	STARD4	134429	brown
226416_at	ERI1	90459	brown
226474_at	NLRC5	84166	brown
226499_at	NRARP	441478	brown
226535_at	ITGB6	3694	brown
226541_at	FBXO30	84085	brown

226590_at	ZNF618	114991	brown
226592_at	ZNF618	114991	brown
226599_at	FHDC1	85462	brown
226609_at	DCBLD1	285761	brown
226613_at	GATSL3	652968	brown
226623_at	PHYHIPL	84457	brown
226637_at	UBE2H	7328	brown
226656_at	CRTAP	10491	brown
226657_at	C17orf103	256302	brown
226681_at	UBE2H	7328	brown
226698_at	FCHSD1	89848	brown
226706_at	FLJ23867 /// QSOX1	5768 /// 200058	brown
226778_at	TDRP	157695	brown
226817_at	DSC2	1824	brown
226824_at	CPXM2	119587	brown
226829_at	AFAP1L2	84632	brown
226858_at	CSNK1E /// LOC100996460	1454 /// 100996460	brown
226884_at	LRRN1	57633	brown
226887_at	HSPA14	51182	brown
226899_at	UNC5B	219699	brown
226905_at	FAM101B	359845	brown
226949_at	GOLGA3	2802	brown
226952_at	EAF1	85403	brown
226988_s_at	MYH14	79784	brown
227032_at	PLXNA2	5362	brown
227080_at	ZNF697	90874	brown
227091_at	CCDC146	57639	brown
227138_at	CRTAP	10491	brown
227173_s_at	BACH2	60468	brown
227283_at	EFR3B	22979	brown
227300_at	TMEM119	338773	brown
227313_at	CNPY4	245812	brown
227317_at	LMCD1	29995	brown
227405_s_at	FZD8	8325	brown
227410_at	FAM43A	131583	brown
227439_at	ANKS1B	56899	brown
227443_at	LURAP1L	286343	brown
227461_at	STON2	85439	brown
227468_at	CPT1C	126129	brown
227481_at	CNKSR3	154043	brown
227491_at	ELOVL6	79071	brown
227499_at	FZD3	7976	brown
227506_at	SLC16A9	220963	brown

227524_at	FZD3	7976	brown
227526_at	CDON	50937	brown
227596_at	FLJ37453	729614	brown
227606_s_at	STAMBPL1	57559	brown
227650_at	HSPA14	51182	brown
227723_at	LOH12CR1	118426	brown
227763_at	LYPD6	130574	brown
227764_at	LYPD6	130574	brown
227801_at	TRIM59	286827	brown
227807_at	PARP9	83666	brown
227828_s_at	EVA1A	84141	brown
227889_at	LPCAT2	54947	brown
227936_at	TMEM68	137695	brown
227942_s_at	CRIPT	9419	brown
227944_at	PTPN3	5774	brown
227950_at	UBE2H	7328	brown
227955_s_at	EFNA5	1946	brown
227993_at	METAP2	10988	brown
228068_at	GOLGA7B	401647	brown
228080_at	LAYN	143903	brown
228090_at	NMNAT3	349565	brown
228121_at	TGFB2	7042	brown
228126_x_at	CTXN1	404217	brown
228128_x_at	PAPPA	5069	brown
228132_at	ABLIM2	84448	brown
228181_at	SLC30A1	7779	brown
228260_at	ELAVL2	1993	brown
228284_at	TLE1	7088	brown
228293_at	DEPDC7	91614	brown
228305_at	ZNF565	147929	brown
228306_at	CNIH4	29097	brown
228307_at	EMILIN3	90187	brown
228314_at	LRRC8C	84230	brown
228329_at	DAB1	1600	brown
228360_at	LYPD6B	130576	brown
228404_at	IRX2	153572	brown
228412_at	LOC643072	643072	brown
228485_s_at	SLC44A1	23446	brown
228486_at	SLC44A1	23446	brown
228490_at	ABHD2	11057	brown
228531_at	SAMD9	54809	brown
228555_at	CAMK2D	817	brown
228587_at	FAM83G	644815	brown

228636_at	BHLHE22	27319	brown
228646_at	PPP1R1C	151242	brown
228653_at	SAMD5	389432	brown
228697_at	HINT3	135114	brown
228703_at	P4HA3	283208	brown
228705_at	CAPN12	147968	brown
228720_at	SORCS2	57537	brown
228780_at	POU3F3	5455	brown
228786_at	PTCHD3P1	387647	brown
228832_at	FLJ20021	90024	brown
228837_at	TCF4	6925	brown
228846_at	MXD1	4084	brown
228882_at	TUB	7275	brown
228885_at	MAMDC2	256691	brown
228894_at	LOC253842 /// NR6A1	2649 /// 253842	brown
228900_at	SPECC1	92521	brown
228910_at	CD82	3732	brown
228915_at	DACH1	1602	brown
228922_at	SHF	90525	brown
228945_s_at	SLC39A8	64116	brown
228975_at	SP6	80320	brown
228977_at	LOC729680	729680	brown
228996_at	RC3H1	149041	brown
229004_at	ADAMTS15	170689	brown
229030_at	CAPN8	388743	brown
229055_at	GPR68	8111	brown
229065_at	SLC35F3	148641	brown
229084_at	CNTN4	152330	brown
229093_at	NOS3	4846	brown
229097_at	DIAPH3	81624	brown
229110_at	SLC24A2	25769	brown
229158_at	WNK4	65266	brown
229168_at	COL23A1	91522	brown
229213_at	DIRC2	84925	brown
229228_at	CREB5 /// LOC401317	9586 /// 401317	brown
229252_at	ATG9B	285973	brown
229275_at	IGFN1	91156	brown
229280_s_at	LINC00340	401237	brown
229290_at	DAPL1	92196	brown
229337_at	USP2	9099	brown
229378_at	STOX1	219736	brown
229427_at	SEMA5A	9037	brown
229442_at	C18orf54	162681	brown

229474_at	MICAL3	57553	brown
229518_at	FAM46B	115572	brown
229581_at	ELFN1	392617	brown
229610_at	CKAP2L	150468	brown
229641_at	CCBE1	147372	brown
229682_at	MAPRE3	22924	brown
229718_at	N4BP2L1	90634	brown
229720_at	BAG1	573	brown
229730_at	SMTNL2	342527	brown
229734_at	MIR4697HG	283174	brown
229738_at	DNAH10	196385	brown
229776_at	SLCO3A1	28232	brown
229817_at	ZNF608	57507	brown
229823_at	RIMS2	9699	brown
229900_at	CD109	135228	brown
229901_at	ZNF488	118738	brown
229909_at	B4GALNT3	283358	brown
229929_at	SPSB4	92369	brown
229933_at	C1orf74	148304	brown
229942_at	BNC2	54796	brown
230021_at	TICRR	90381	brown
230051_at	PROSER2	254427	brown
230068_s_at	PEG3-AS1	100169890	brown
230104_s_at	TPPP	11076	brown
230135_at	HHIP	64399	brown
230138_at	NCS1	23413	brown
230150_at	BCAP29	55973	brown
230184_at	LOC100506421	100506421	brown
230195_at	LOC100131138	100131138	brown
230197_s_at	TPPP	11076	brown
230201_at	FXR1	8087	brown
230254_at	FAM26E	254228	brown
230276_at	FAM49A	81553	brown
230280_at	TRIM9	114088	brown
230369_at	GPR161	23432	brown
230430_at	ENTPD2	954	brown
230441_at	PLEKHG4B	153478	brown
230467_at	TMEM52	339456	brown
230469_at	RTKN2	219790	brown
230493_at	SHISA2	387914	brown
230498_at	MCHR1	2847	brown
230526_at	TNRC6C-AS1	100131096	brown
230560_at	STXBP6	29091	brown

230622_at	MLLT4	4301	brown
230673_at	PKHD1L1	93035	brown
230708_at	PRICKLE1	144165	brown
230720_at	RNF182	221687	brown
230722_at	BNC2	54796	brown
230740_at	EHD3	30845	brown
230788_at	GCNT2	2651	brown
230830_at	SLC51B	123264	brown
231192_at	LPAR3	23566	brown
231230_at	KCNK10	54207	brown
231325_at	UNC5D	137970	brown
231382_at	FGF18	8817	brown
231395_at	ATP8A2	51761	brown
231439_at	FAM84A	151354	brown
231461_at	KRT71	112802	brown
231565_at	C4orf22	255119	brown
231583_at	KRT74	121391	brown
231609_at	C10orf82	143379	brown
231669_at	SEPP1	6414	brown
231733_at	CARD18	59082	brown
231743_at	WNT3	7473	brown
231771_at	GJB6	10804	brown
231778_at	DLX3	1747	brown
231844_at	MGC27345	157247	brown
231867_at	TENM2	57451	brown
231875_at	KIF21A	55605	brown
231935_at	ARPP21	10777	brown
232009_at	EMR2	30817	brown
232026_at	HERC4	26091	brown
232027_at	SYNE1	23345	brown
232069_at	KIF26A	26153	brown
232105_at	BLACAT1	101669762	brown
232232_s_at	SLC22A16	85413	brown
232233_at	SLC22A16	85413	brown
232253_at	C5orf56	441108	brown
232303_at	ZNF608	57507	brown
232311_at	B2M	567	brown
232322_x_at	STARD10	10809	brown
232348_at	C18orf8	29919	brown
232378_at	SLC5A9	200010	brown
232408_at	ZFYVE28	57732	brown
232547_at	SRCIN1	80725	brown
232555_at	CREB5	9586	brown

232568_at	MGC24103	158295	brown
232596_at	DIAPH3	81624	brown
232662_x_at	FAM213A	84293	brown
232682_at	MREG	55686	brown
232717_at	KALRN	8997	brown
232720_at	LINGO2	158038	brown
232887_at	PIRT	644139	brown
232914_s_at	SYTL2	54843	brown
232977_x_at	MYH14	79784	brown
233029_at	OBSCN	84033	brown
233111_at	PTCSC1	100302522	brown
233123_at	SLC40A1	30061	brown
233158_at	KRT82	3888	brown
233203_at	ROPN1	54763	brown
233301_at	OXCT2	64064	brown
233340_at	SPINK13	153218	brown
233410_at	CYP1B1-AS1	285154	brown
233520_s_at	CMYA5	202333	brown
233533_at	KRTAP1-5	83895	brown
233534_at	KRTAP3-2	83897	brown
233537_at	KRTAP3-1	83896	brown
233555_s_at	SULF2	55959	brown
233564_s_at	CDADC1	81602	brown
233586_s_at	KLK12	43849	brown
233587_s_at	SIPA1L2	57568	brown
233631_x_at	KRTAP9-2 /// KRTAP9-8 /// LOC100996762	83899 /// 83901 /// 100996762	brown
233640_x_at	KRTAP9-4	85280	brown
233681_at	KRTAP3-3	85293	brown
233814_at	EFNA5	1946	brown
234290_x_at	MYH14	79784	brown
234316_x_at	KLK12	43849	brown
234335_s_at	FAM84A	151354	brown
234351_x_at	TRPS1	7227	brown
234488_s_at	GMCL1 /// GMCL1P1	64395 /// 64396	brown
234631_at	KRTAP4-8	728224	brown
234633_at	KRTAP4-11	653240	brown
234635_at	KRTAP4-1	85285	brown
234637_at	KRTAP4-5	85289	brown
234639_x_at	KRTAP9-8	83901	brown
234671_at	KRTAP4-2	85291	brown

234678_at	KRTAP4-3	85290	brown
234679_at	KRTAP9-3	83900	brown
234680_at	KRTAP17-1	83902	brown
234683_at	KRTAP4-11 /// KRTAP4-6 /// LOC100134267	81871 /// 653240 /// 100134267	brown
234684_s_at	KRTAP4-4	84616	brown
234685_x_at	KRTAP4-9	100132386	brown
234691_at	KRTAP2-1	81872	brown
234772_s_at	KRTAP2-1 /// KRTAP2-2	81872 /// 728279	brown
234880_x_at	KRTAP1-3	81850	brown
234925_at	LINC00917	732275	brown
234986_at	GCLM	2730	brown
234987_at	SAMHD1	25939	brown
235012_at	LRCH1	23143	brown
235020_at	TAF4B	6875	brown
235022_at	FAM210A	125228	brown
235068_at	ZDHHC21	340481	brown
235088_at	C4orf46	201725	brown
235095_at	CCDC64B	146439	brown
235107_at	PIAS2	9063	brown
235144_at	RASEF	158158	brown
235224_s_at	CAND1	55832	brown
235225_at	SCN2B	6327	brown
235256_s_at	GALM	130589	brown
235275_at	BMP8B	656	brown
235276_at	EPSTI1	94240	brown
235310_at	GCSAM	257144	brown
235315_at	TSC22D1	8848	brown
235367_at	MYPN	84665	brown
235479_at	CPEB2	132864	brown
235516_at	SEPSECS	51091	brown
235591_at	SSTR1	6751	brown
235672_at	MAP6	4135	brown
235683_at	SESN3	143686	brown
235684_s_at	SESN3	143686	brown
235703_at	PLB1	151056	brown
235723_at	BNC2	54796	brown
235737_at	TSLP	85480	brown
235871_at	LIPH	200879	brown
236001_at	LINC00675	100289255	brown

236013_at	CACNA1E	777	brown
236255_at	PLEKHG4B	153478	brown
236261_at	OSBPL6	114880	brown
236263_at	SHH	6469	brown
236264_at	LPHN3	23284	brown
236285_at	KLHDC7B	113730	brown
236402_at	BRAF	673	brown
236514_at	ACOT8	10005	brown
236548_at	GIPC2	54810	brown
236651_at	KALRN	8997	brown
236817_at	ADAT2	134637	brown
236900_x_at	CCDC163P	126661	brown
237003_at	BEST3	144453	brown
237016_at	TMEM217	221468	brown
237120_at	KRT77	374454	brown
237466_s_at	HHIP	64399	brown
237507_at	KRT73	319101	brown
237623_at	CST3	1471	brown
237732_at	PRR9	574414	brown
237833_s_at	SNCAIP	9627	brown
237853_x_at	KRTAP10-12	386685	brown
237905_at	KRT25	147183	brown
237974_at	ABHD12B /// MIR4454	145447 /// 100616234	brown
237981_at	CMYA5	202333	brown
238001_at	KCTD6	200845	brown
238168_at	TM4SF1	4071	brown
238222_at	GKN2	200504	brown
238444_at	ZNF618	114991	brown
238453_at	FGFBP3	143282	brown
238460_at	FAM83A	84985	brown
238478_at	BNC2	54796	brown
238479_at	DCUN1D5	84259	brown
238520_at	TRERF1	55809	brown
238568_s_at	NPC1	4864	brown
238575_at	OSBPL6	114880	brown
238583_at	MSRB3	253827	brown
238584_at	IQCA1	79781	brown
238625_at	C1orf168	199920	brown
238669_at	PTGS1	5742	brown
238741_at	FAM83A	84985	brown
238756_at	GAS2L3	283431	brown
238789_at	KANK1	23189	brown
238871_at	MLLT4	4301	brown

239028_at	LYPD6	130574	brown
239062_at	TNRC6C-AS1	100131096	brown
239082_at	FZD3	7976	brown
239136_at	UNC5B-AS1	728978	brown
239142_at	RFESD	317671	brown
239148_at	MARVELD3	91862	brown
239183_at	ANGPTL1	9068	brown
239202_at	RAB3B	5865	brown
239203_at	LSMEM1	286006	brown
239217_x_at	ABCC3	8714	brown
239309_at	DLX6	1750	brown
239350_at	MARVELD3	91862	brown
239355_at	GMCL1	64395	brown
239425_at	DCUN1D5	84259	brown
239572_at	GJA3	2700	brown
239582_at	PML	5371	brown
239586_at	FAM83A	84985	brown
239650_at	NCKAP5	344148	brown
239707_at	SLC5A10	125206	brown
239719_at	CD109	135228	brown
239913_at	SLC10A4	201780	brown
240024_at	SEC14L2	23541	brown
240306_at	LINC00312	29931	brown
240388_at	KRT27	342574	brown
240389_at	TRPM6	140803	brown
240717_at	ABCB5	340273	brown
240806_at	RPL15	6138	brown
240967_at	KRTAP19-3	337970	brown
241014_at	FLG-AS1	339400	brown
241371_at	TNFRSF10A	8797	brown
241383_at	ZNF385C	201181	brown
241412_at	BTC	685	brown
241733_at	C18orf54	162681	brown
241765_at	CPM	1368	brown
241851_x_at	LOC100130429	100130429	brown
241985_at	JMY	133746	brown
242062_at	SAMD8	142891	brown
242138_at	DLX1	1745	brown
242186_x_at	LPHN3	23284	brown
242283_at	DNAH14	127602	brown
242301_at	CBLN2	147381	brown
242329_at	CREB5 /// LOC401317	9586 /// 401317	brown
242344_at	GABRB2	2561	brown

242364_x_at	TNRC6C-AS1	100131096	brown
242417_at	LOC283278	283278	brown
242450_at	RGMB	285704	brown
242516_x_at	RBM46	166863	brown
242583_at	STON2	85439	brown
242592_at	GPR137C	283554	brown
242626_at	SAMD5	389432	brown
242660_at	C10orf112	340895	brown
242767_at	LMCD1	29995	brown
242899_at	SESN3	143686	brown
242906_at	SESN3	143686	brown
242940_x_at	DLX6	1750	brown
243161_x_at	ZFP42	132625	brown
243167_at	ABCB5	340273	brown
243403_x_at	CPM	1368	brown
243445_at	BNC2	54796	brown
243478_at	CHST2	9435	brown
243541_at	IL31RA	133396	brown
243595_at	SHB	6461	brown
243611_at	MICALCL	84953	brown
243818_at	SFTA1P	207107	brown
243843_at	N4BP2L1	90634	brown
244118_at	GABRA1	2554	brown
244294_at	GTF2H5	404672	brown
244324_at	C18orf54	162681	brown
244620_at	SLC8A1-AS1	100128590	brown
244650_at	FAM105A	54491	brown
244758_at	SCAND3	114821	brown
34697_at	LRP6	4040	brown
36564_at	RNF19B	127544	brown
37408_at	MRC2	9902	brown
37424_at	CCHCR1	54535	brown
37425_g_at	CCHCR1	54535	brown
37577_at	ARHGAP19	84986	brown
38037_at	HBEGF	1839	brown
38241_at	BTN3A3	10384	brown
40148_at	APBB2	323	brown
40420_at	STK10	6793	brown
41856_at	UNC5B	219699	brown

42361_g_at	CCHCR1	54535	brown
52651_at	COL8A2	1296	brown
57588_at	SLC24A3	57419	brown
64488_at	IRGQ	126298	brown
91684_g_at	EXOSC4	54512	brown
1405_i_at	CCL5	6352	green
1552398_a_at	CLEC12A	160364	green
1552480_s_at	PTPRC	5788	green
1552497_a_at	SLAMF6	114836	green
1552552_s_at	CLEC4C	170482	green
1552553_a_at	NLRC4	58484	green
1552584_at	IL12RB1	3594	green
1552849_at	M1AP	130951	green
1553260_s_at	ALS2CR11	151254	green
1553378_a_at	C17orf66	256957	green
1553645_at	CCDC141	285025	green
1553906_s_at	FGD2	221472	green
1554240_a_at	ITGAL	3683	green
1554335_at	CYTH4	27128	green
1554406_a_at	CLEC7A	64581	green
1554503_a_at	OSCAR	126014	green
1554519_at	CD80	941	green
1554569_a_at	CELF2	10659	green
1554704_at	ATP8B3	148229	green
1554899_s_at	FCER1G	2207	green
1554930_a_at	FUT8	2530	green
1555120_at	CD96	10225	green
1555275_a_at	KLHL6	89857	green
1555638_a_at	SAMSN1	64092	green
1555728_a_at	MS4A4A	51338	green
1555756_a_at	CLEC7A	64581	green
1555962_at	B3GNT7	93010	green
1555963_x_at	B3GNT7	93010	green
1556136_at	MYLK4	340156	green
1556346_at	COTL1	23406	green
1556378_a_at	LOC440896	440896	green
1556471_at	SCML4	256380	green
1556472_s_at	SCML4	256380	green
1557116_at	APOL6	80830	green
1558827_a_at	ZNF831	128611	green
1558881_at	LINC00924	145820	green
1558971_at	THEMIS	387357	green
1558972_s_at	THEMIS	387357	green

1559263_s_at	ZC3H12D	340152	green
1559584_a_at	C16orf54	283897	green
1560762_at	LOC285972	285972	green
1561226_at	XCR1	2829	green
1562364_at	GVINP1	387751	green
1563638_at	TVP23A	780776	green
1563674_at	FCRL2	79368	green
1564276_at	C5orf56	441108	green
1564310_a_at	PARP15	165631	green
1568609_s_at	FLJ39739 /// LINC00623 /// LINC00869 /// LINC00875 /// LOC728875	57234 /// 388685 /// 728855 /// 728875 /// 100286793	green
1569225_a_at	SCML4	256380	green
200887_s_at	STAT1	6772	green
201859_at	SRGN	5552	green
202156_s_at	CELF2	10659	green
202499_s_at	SLC2A3	6515	green
202510_s_at	TNFAIP2	7127	green
202524_s_at	SPOCK2	9806	green
202625_at	LYN	4067	green
202637_s_at	ICAM1	3383	green
202643_s_at	TNFAIP3	7128	green
202644_s_at	TNFAIP3	7128	green
202663_at	WIPF1	7456	green
202665_s_at	WIPF1	7456	green
202803_s_at	ITGB2	3689	green
202901_x_at	CTSS	1520	green
202912_at	ADM	133	green
202952_s_at	ADAM12	8038	green
202953_at	C1QB	713	green
202957_at	HCLS1	3059	green
203052_at	C2	717	green
203241_at	UVRAG	7405	green
203305_at	F13A1	2162	green
203320_at	SH2B3	10019	green
203332_s_at	INPP5D	3635	green
203402_at	KCNAB2	8514	green
203416_at	CD53	963	green
203471_s_at	PLEK	5341	green
203485_at	RTN1	6252	green
203508_at	TNFRSF1B	7133	green
203523_at	LSP1	4046	green
203547_at	CD4	920	green

203645_s_at	CD163	9332	green
203761_at	SLA	6503	green
203779_s_at	MPZL2	10205	green
203879_at	PIK3CD	5293	green
203915_at	CXCL9	4283	green
203932_at	HLA-DMB	3109	green
204046_at	PLCB2	5330	green
204070_at	RARRES3	5920	green
204103_at	CCL4	6351	green
204122_at	TYROBP	7305	green
204150_at	STAB1	23166	green
204153_s_at	MFNG	4242	green
204205_at	APOBEC3G	60489	green
204220_at	GMFG	9535	green
204232_at	FCER1G	2207	green
204265_s_at	GPSM3	63940	green
204352_at	TRAF5	7188	green
204411_at	KIF21B	23046	green
204438_at	MRC1	4360	green
204440_at	CD83	9308	green
204502_at	SAMHD1	25939	green
204533_at	CXCL10	3627	green
204563_at	SELL	6402	green
204580_at	MMP12	4321	green
204588_s_at	SLC7A7	9056	green
204655_at	CCL5	6352	green
204670_x_at	HLA-DRB1 /// HLA-DRB4 /// LOC100507709 /// LOC100507714	3123 /// 3126 /// 100507709 /// 100507714	green
204674_at	LRMP	4033	green
204689_at	HHEX	3087	green
204735_at	PDE4A	5141	green
204770_at	TAP2	6891	green
204774_at	EVI2A	2123	green
204789_at	FMNL1	752	green
204829_s_at	FOLR2	2350	green
204852_s_at	PTPN7	5778	green
204882_at	ARHGAP25	9938	green
204891_s_at	LCK	3932	green
204912_at	IL10RA	3587	green
204951_at	RHOH	399	green
204959_at	MNDA	4332	green
204960_at	PTPRCAP	5790	green
204961_s_at	NCF1 /// NCF1B /// NCF1C	653361 /// 654816 ///	green

205013_s_at	ADORA2A /// SPECC1L-ADORA2A	654817<br>135 /// 101730217	green
205038_at	IKZF1	10320	green
205098_at	CCR1	1230	green
205101_at	CIITA	4261	green
205114_s_at	CCL3 /// CCL3L1 /// CCL3L3 ///<br>LOC101060267	6348 /// 6349 /// 414062 ///<br>101060267	green
205147_x_at	NCF4	4689	green
205159_at	CSF2RB	1439	green
205180_s_at	ADAM8	101	green
205212_s_at	ACAP1	9744	green
205213_at	ACAP1	9744	green
205269_at	LCP2	3937	green
205270_s_at	LCP2	3937	green
205291_at	IL2RB	3560	green
205306_x_at	KMO	8564	green
205307_s_at	KMO	8564	green
205419_at	GPR183	1880	green
205456_at	CD3E	916	green
205484_at	SIT1	27240	green
205488_at	GZMA	3001	green
205495_s_at	GNLY	10578	green
205543_at	HSPA4L	22824	green
205554_s_at	DNASE1L3	1776	green
205639_at	AOAH	313	green
205640_at	ALDH3B1	221	green
205660_at	OASL	8638	green
205668_at	LY75	4065	green
205685_at	CD86	942	green
205758_at	CD8A	925	green
205786_s_at	ITGAM	3684	green
205790_at	SKAP1	8631	green
205804_s_at	TRAF3IP3	80342	green
205819_at	MARCO	8685	green
205821_at	KLRC4-KLRK1 /// KLRK1	22914 /// 100528032	green
205831_at	CD2	914	green
205898_at	CX3CR1	1524	green
205987_at	CD1C	911	green

205992_s_at	IL15	3600	green
205997_at	ADAM28	10863	green
206060_s_at	PTPN22	26191	green
206090_s_at	DISC1 /// TSNAX-DISC1	27185 /// 100303453	green
206118_at	STAT4	6775	green
206120_at	CD33	945	green
206134_at	ADAMDEC1	27299	green
206181_at	SLAMF1	6504	green
206219_s_at	VAV1	7409	green
206247_at	MICB	4277	green
206271_at	TLR3	7098	green
206296_x_at	MAP4K1	11184	green
206337_at	CCR7	1236	green
206366_x_at	XCL1	6375	green
206370_at	PIK3CG	5294	green
206407_s_at	CCL13	6357	green
206502_s_at	INSM1	3642	green
206513_at	AIM2	9447	green
206545_at	CD28	940	green
206569_at	IL24	11009	green
206584_at	LY96	23643	green
206637_at	P2RY14	9934	green
206666_at	GZMK	3003	green
206674_at	FLT3	2322	green
206707_x_at	FAM65B	9750	green
206715_at	TFEC	22797	green
206749_at	CD1B	910	green
206761_at	CD96	10225	green
206785_s_at	KLRC1 /// KLRC2	3821 /// 3822	green
206804_at	CD3G	917	green
206856_at	LILRB5	10990	green
206914_at	CRTAM	56253	green
206950_at	SCN9A	6335	green
206974_at	CXCR6	10663	green
206978_at	CCR2	729230	green
206991_s_at	CCR5	1234	green
207072_at	IL18RAP	8807	green
207075_at	NLRP3	114548	green
207277_at	CD209	30835	green
207315_at	CD226	10666	green
207328_at	ALOX15	246	green
207339_s_at	LTB	4050	green

207351_s_at	SH2D2A	9047	green
207376_at	VENTX	27287	green
207509_s_at	LAIR2	3904	green
207571_x_at	THEMIS2	9473	green
207610_s_at	EMR2	30817	green
207677_s_at	NCF4	4689	green
207697_x_at	LILRB2	10288	green
207777_s_at	SP140	11262	green
207861_at	CCL22	6367	green
207901_at	IL12B	3593	green
207979_s_at	CD8B /// LOC100996919	926 /// 100996919	green
208018_s_at	HCK	3055	green
208121_s_at	PTPRO	5800	green
208146_s_at	CPVL	54504	green
208189_s_at	MYO7A	4647	green
208268_at	ADAM28	10863	green
208306_x_at	HLA-DRB1 /// LOC100507709 /// LOC100507714	3123 /// 100507709 /// 100507714	green
208438_s_at	FGR	2268	green
208650_s_at	CD24	100133941	green
208651_x_at	CD24	100133941	green
209083_at	CORO1A	11151	green
209312_x_at	HLA-DRB1 /// HLA-DRB4 /// HLA-DRB5 /// LOC100507709 /// LOC100507714	3123 /// 3126 /// 3127 /// 100507709 /// 100507714	green
209525_at	HDGFRP3	50810	green
209546_s_at	APOL1	8542	green
209606_at	CYTIP	9595	green
209619_at	CD74	972	green
209670_at	TRAC	28755	green
209734_at	NCKAP1L	3071	green
209765_at	ADAM19	8728	green
209770_at	BTN3A1	11119	green
209772_s_at	CD24	100133941	green
209813_x_at	TARP	445347	green
209827_s_at	IL16	3603	green
209879_at	SELPLG	6404	green
209882_at	RIT1	6016	green
209892_at	FUT4	2526	green
209901_x_at	AIF1	199	green
209906_at	C3AR1	719	green
209924_at	CCL18 /// LOC101060271	6362 /// 101060271	green

209969_s_at	STAT1	6772	green
210029_at	IDO1	3620	green
210031_at	CD247	919	green
210038_at	PRKCQ	5588	green
210140_at	CST7	8530	green
210146_x_at	LILRB2	10288	green
210163_at	CXCL11	6373	green
210164_at	GZMB	3002	green
210184_at	ITGAX	3687	green
210222_s_at	RTN1	6252	green
210321_at	GZMH	2999	green
210354_at	IFNG	3458	green
210356_x_at	MS4A1	931	green
210439_at	ICOS	29851	green
210448_s_at	P2RX5	5026	green
210538_s_at	BIRC3	330	green
210607_at	FLT3LG	2323	green
210784_x_at	LILRA6 /// LILRB3	11025 /// 79168	green
210785_s_at	THEMIS2	9473	green
210845_s_at	PLAUR	5329	green
210895_s_at	CD86	942	green
210915_x_at	TRBC1	28639	green
211122_s_at	CXCL11	6373	green
211138_s_at	KMO	8564	green
211144_x_at	TARP /// TRGC2	6967 /// 445347	green
211339_s_at	ITK	3702	green
211434_s_at	CCRL2	9034	green
211734_s_at	FCER1A	2205	green
211742_s_at	EVI2B	2124	green
211990_at	HLA-DPA1	3113	green
211991_s_at	HLA-DPA1	3113	green
212268_at	SERPINB1	1992	green
212588_at	PTPRC	5788	green
212750_at	PPP1R16B	26051	green
212827_at	IGHM	3507	green
212873_at	HMHA1	23526	green
213095_x_at	AIF1	199	green
213160_at	DOCK2	1794	green
213193_x_at	TRBC1	28639	green
213293_s_at	TRIM22	10346	green
213309_at	PLCL2	23228	green

213416_at	ITGA4	3676	green
213475_s_at	ITGAL	3683	green
213537_at	HLA-DPA1	3113	green
213539_at	CD3D	915	green
213603_s_at	RAC2	5880	green
213733_at	MYO1F	4542	green
213790_at	ADAM12	8038	green
213888_s_at	TRAF3IP3	80342	green
213915_at	NKG7	4818	green
213975_s_at	LYZ	4069	green
214032_at	ZAP70	7535	green
214038_at	CCL8	6355	green
214054_at	DOK2	9046	green
214084_x_at	NCF1C	654817	green
214180_at	MAN1C1	57134	green
214219_x_at	MAP4K1	11184	green
214339_s_at	MAP4K1	11184	green
214450_at	CTSW	1521	green
214467_at	GPR65	8477	green
214470_at	KLRB1	3820	green
214567_s_at	XCL1 /// XCL2	6375 /// 6846	green
214572_s_at	INSL3	3640	green
214617_at	PRF1	5551	green
214735_at	IPCEF1	26034	green
215049_x_at	CD163	9332	green
215051_x_at	AIF1	199	green
215193_x_at	HLA-DRB1 /// HLA-DRB3 /// HLA-DRB4 /// LOC100507709 /// LOC100507714	3123 /// 3125 /// 3126 /// 100507709 /// 100507714	green
215305_at	PDGFRA	5156	green
215332_s_at	CD8B /// LOC100996919	926 /// 100996919	green
215784_at	CD1E	913	green
215806_x_at	TARP /// TRGC2	6967 /// 445347	green
215967_s_at	LY9	4063	green
215979_s_at	SLC7A1	6541	green
216233_at	CD163	9332	green
216250_s_at	LPXN	9404	green
216714_at	CCL13	6357	green
216748_at	PYHIN1	149628	green
216920_s_at	TARP /// TRGC2	6967 /// 445347	green
217147_s_at	TRAT1	50852	green
217371_s_at	IL15	3600	green
217418_x_at	MS4A1	931	green

217478_s_at	HLA-DMA	3108	green
217767_at	C3	718	green
218232_at	C1QA	712	green
218345_at	TMEM176A	55365	green
218600_at	LIMD2	80774	green
218724_s_at	TGIF2	60436	green
218870_at	ARHGAP15	55843	green
219014_at	PLAC8	51316	green
219045_at	RHOF	54509	green
219161_s_at	CKLF /// CKLF-CMTM1	51192 /// 100529251	green
219183_s_at	CYTH4	27128	green
219209_at	IFIH1	64135	green
219279_at	DOCK10	55619	green
219385_at	SLAMF8	56833	green
219386_s_at	SLAMF8	56833	green
219387_at	CCDC88A	55704	green
219452_at	DPEP2	64174	green
219471_at	KIAA0226L	80183	green
219505_at	CECR1	51816	green
219506_at	C1orf54	79630	green
219519_s_at	SIGLEC1	6614	green
219574_at	1-Mar	55016	green
219593_at	SLC15A3	51296	green
219594_at	NINJ2	4815	green
219607_s_at	MS4A4A	51338	green
219620_x_at	TOR4A	54863	green
219666_at	MS4A6A	64231	green
219690_at	IGFLR1	79713	green
219716_at	APOL6	80830	green
219812_at	PVRIG	79037	green
219885_at	SLFN12	55106	green
219892_at	TM6SF1	53346	green
219947_at	CLEC4A	50856	green
220000_at	SIGLEC5	8778	green
220005_at	P2RY13	53829	green
220032_at	CPED1	79974	green
220112_at	ANKRD55	79722	green
220330_s_at	SAMSN1	64092	green
220358_at	BATF3	55509	green
220418_at	UBASH3A	53347	green
220423_at	PLA2G2D	26279	green
220485_s_at	SIRPG	55423	green
220532_s_at	TMEM176B	28959	green

220560_at	C11orf21	29125	green
220577_at	GVINP1	387751	green
220749_at	C10orf68	79741	green
221058_s_at	CKLF	51192	green
221080_s_at	DENND1C	79958	green
221087_s_at	APOL3	80833	green
221218_s_at	TPK1	27010	green
221241_s_at	BCL2L14	79370	green
221487_s_at	ENSA	2029	green
221601_s_at	FAIM3	9214	green
221698_s_at	CLEC7A	64581	green
221724_s_at	CLEC4A	50856	green
222868_s_at	IL18BP	10068	green
223280_x_at	MS4A6A	64231	green
223303_at	FERMT3	83706	green
223343_at	MS4A7	58475	green
223501_at	TNFSF13B	10673	green
223533_at	LRRC8C	84230	green
223562_at	PARVG	64098	green
223640_at	HCST	10870	green
223710_at	CCL26	10344	green
223725_at	LINC00852	84657	green
223922_x_at	MS4A6A	64231	green
224356_x_at	MS4A6A	64231	green
224357_s_at	MS4A4A	51338	green
224451_x_at	ARHGAP9	64333	green
224480_s_at	AGPAT9	84803	green
224909_s_at	PREX1	57580	green
225285_at	BCAT1	586	green
225353_s_at	C1QC	714	green
225415_at	DTX3L	151636	green
225602_at	GLIPR2	152007	green
225701_at	AKNA	80709	green
225763_at	RCSD1	92241	green
225973_at	TAP2	6891	green
226136_at	GLIPR1	11010	green
226142_at	GLIPR1	11010	green
226218_at	IL7R	3575	green
226219_at	ARHGAP30	257106	green

226333_at	IL6R		3570	green
226423_at	PAQR8		85315	green
226436_at	RASSF4		83937	green
226517_at	BCAT1		586	green
226603_at	SAMD9L		219285	green
226641_at	ANKRD44		91526	green
226673_at	SH2D3C		10044	green
226743_at	SLFN11		91607	green
226777_at	ADAM12 /// ADAM12-OT1		8038 /// 100967225	green
226818_at	MPEG1		219972	green
226841_at	MPEG1		219972	green
226878_at	HLA-DOA		3111	green
226879_at	HVCN1		84329	green
226906_s_at	ARHGAP9		64333	green
227002_at	FAM78A		286336	green
227030_at	IKZF3		22806	green
227145_at	LOXL4		84171	green
227168_at	MIAT		440823	green
227266_s_at	FYB		2533	green
227344_at	IKZF1		10320	green
227346_at	IKZF1		10320	green
227353_at	TMC8		147138	green
227552_at		1-Sep	1731	green
227553_at	PIK3R5		23533	green
227609_at	EPSTI1		94240	green
227645_at	PIK3R5		23533	green
227647_at	KCNE3		10008	green
227677_at	JAK3		3718	green
227749_at	POU2F2		5452	green
227867_at	TRABD2A		129293	green
227877_at	ANXA2R		389289	green
228055_at	NAPSB		256236	green
228056_s_at	NAPSB		256236	green
228071_at	GIMAP7		168537	green
228094_at	AMICA1		120425	green
228113_at	RAB37		326624	green
228167_at	KLHL6		89857	green
228258_at	TBC1D10C		374403	green
228298_at	PCED1B		91523	green
228362_s_at	FAM26F		441168	green
228532_at	C1orf162		128346	green
228592_at	MS4A1		931	green
228599_at	MS4A1		931	green

228677_s_at	RASAL3		64926	green
228869_at	SNX20		124460	green
229029_at	CAMK4		814	green
229383_at		1-Mar	55016	green
229390_at	FAM26F		441168	green
229391_s_at	FAM26F		441168	green
229437_at	MIR155 /// MIR155HG		114614 /// 406947	green
229510_at	MS4A14		84689	green
229585_at	ADAMTSL1		92949	green
229597_s_at	WDFY4		57705	green
229619_at	FOLR2		2350	green
229625_at	GBP5		115362	green
229723_at	TAGAP		117289	green
229779_at	COL4A4		1286	green
229872_s_at	LOC100132999 /// LOC100996473 /// LOC100996720 /// LOC100996740 /// LOC101060404 /// LOC101060562 /// LOC101060645 /// LOC101060698 /// LOC642441 /// LOC730256		642441 /// 730256 /// 100132999 /// 100996473 /// 100996720 /// 100996740 /// 101060404 /// 101060562 /// 101060645 /// 101060698	green
230000_at	RNF213		57674	green
230025_at	GJD3		125111	green
230036_at	SAMD9L		219285	green
230110_at	MCOLN2		255231	green
230261_at	ST8SIA4		7903	green
230391_at	CD84		8832	green
230422_at	FPR3		2359	green
230499_at	BIRC3		330	green
230550_at	MS4A6A		64231	green
230753_at	PATL2		197135	green
230756_at	ZNF683		257101	green
230805_at	MIR142		406934	green
230836_at	ST8SIA4		7903	green
230867_at	COL6A6		131873	green
230925_at	APBB1IP		54518	green
230966_at	IL4I1		259307	green
230980_x_at	MIR142		406934	green
230983_at	FAM129C		199786	green
231093_at	FCRL3		115352	green
231124_x_at	LY9		4063	green
231406_at	ORAI2		80228	green
231776_at	EOMES		8320	green
231911_at	ERMN		57471	green

232001_at	PRKCQ-AS1	439949	green
232024_at	GIMAP2	26157	green
232234_at	SLA2	84174	green
232383_at	TFEC	22797	green
232543_x_at	ARHGAP9	64333	green
232617_at	CTSS	1520	green
232724_at	MS4A6A	64231	green
233086_at	FAM209A	200232	green
233510_s_at	PARVG	64098	green
234165_at	PTGDR	5729	green
234974_at	GALM	130589	green
235080_at	CLUAP1	23059	green
235175_at	GBP4	115361	green
235353_at	SEL1L3	23231	green
235458_at	HAVCR2	84868	green
235529_x_at	SAMHD1	25939	green
235556_at	CREBRF	153222	green
235574_at	GBP4	115361	green
235735_at	TNFSF8	944	green
235964_x_at	SAMHD1	25939	green
236226_at	BTLA	151888	green
236295_s_at	NLRC3	197358	green
236539_at	PTPN22	26191	green
236583_at	GIMAP1	170575	green
236673_at	TIFAB	497189	green
236782_at	SAMD3	154075	green
236854_at	LINC00494	284749	green
236995_x_at	TFEC	22797	green
237753_at	IL21R	50615	green
238429_at	TMEM71	137835	green
238581_at	GBP5	115362	green
238600_at	JAKMIP1	152789	green
238621_at	FMN1	342184	green
238725_at	IRF1	3659	green
239427_at	SLAMF1	6504	green
239529_at	C5orf20 /// TIFAB	140947 /// 497189	green
239802_at	SAP30L	79685	green
240070_at	TIGIT	201633	green
240413_at	PYHIN1	149628	green
240862_at	RASGRP4	115727	green
241791_at	TTN	7273	green
241819_at	TNFSF8	944	green

241871_at	CAMK4	814	green
242268_at	CELF2	10659	green
242388_x_at	TAGAP	117289	green
242641_at	COL6A6	131873	green
242916_at	CNTRL	11064	green
242945_at	FAM20A	54757	green
243099_at	NFAM1	150372	green
243601_at	LOC285957	285957	green
243602_at	MGC40069	348035	green
243968_x_at	FCRL1	115350	green
244251_at	LCP2	3937	green
244313_at	CR1	1378	green
244413_at	CLECL1	160365	green
244578_at	LCP2	3937	green
244598_at	LCP2	3937	green
266_s_at	CD24	100133941	green
32128_at	CCL18 /// LOC101060271	6362 /// 101060271	green
33197_at	MYO7A	4647	green
35974_at	LRMP	4033	green
37145_at	GNLY	10578	green
38149_at	ARHGAP25	9938	green
38487_at	STAB1	23166	green
41577_at	PPP1R16B	26051	green
44673_at	SIGLEC1	6614	green
44790_s_at	KIAA0226L	80183	green
